# Complementary metal-oxide-semiconductor (CMOS) time of evaporation measurement system for binary chemical monitoring

**DOI:** 10.1038/s41598-026-35322-x

**Published:** 2026-01-23

**Authors:** Ebrahim Ghafar-Zadeh, Saghi Forouhi, Hamed Osouli Tabrizi, Abbas Panahi, Yasaman Tahernezhad, Azadeh Amrollahi Biyouki

**Affiliations:** 1 Biologically Inspired Sensors and Actuators Laboratory (BioSA Lab), Department of Electrical Engineering and Computer Science, Lassonde School of Engineering, York University, Toronto, ON M3J1P3 Canada; 2 Division of Electronics and Computer Engineering (ELDA), Department of Electrical Engineering (ISY), Linköping University, 581 83 Linköping, Sweden

**Keywords:** CMOS capacitive sensor, Binary liquid mixtures, Time-of-evaporation, Dielectric sensing, LOESS modeling, Chemical analysis, Portable diagnostics, Electrical and electronic engineering, Engineering

## Abstract

Accurate, real-time analysis of binary liquid mixtures is essential in chemical sensing, especially for miniaturized, low-cost applications. We present a complementary metal-oxide-semiconductor (CMOS)-based platform—ITEMS (Integrated Time-of-Evaporation Measurement System)—designed to monitor binary mixtures via high-resolution capacitive sensing of evaporation dynamics. ITEMS employs an integrated capacitive sensor to detect time-resolved dielectric changes during droplet evaporation under controlled temperatures. By extracting features such as intermediate evaporation time (Δt₂), total evaporation time (ToE), and capacitance variation (ΔCap), ITEMS provides multidimensional insights into solvent composition. We validated the system across ethanol–water, methanol–water, and methanol–ethanol mixtures, with concentrations from 0 to 100% and temperatures between 25 °C and 60 °C. Our analysis reveals that evaporation time and dielectric response exhibit nonlinear dependencies on solvent concentration, particularly at elevated temperatures. Comparative modeling using linear regression and LOESS confirms LOESS’s superiority in capturing these trends, yielding lower Root Mean Square Error (RMSE) values across all datasets. The CMOS integration enables compact packaging, low sample volume requirements (< 1 μL), and direct digital interfacing via a microcontroller and graphical user interface (GUI). These results establish ITEMS as a robust, scalable platform for high-sensitivity, real-time chemical composition monitoring in environmental, biomedical, and industrial applications.

**Supplementary Information:**

The online version contains supplementary material available at 10.1038/s41598-026-35322-x.

## Introduction

**Fig. 1:**
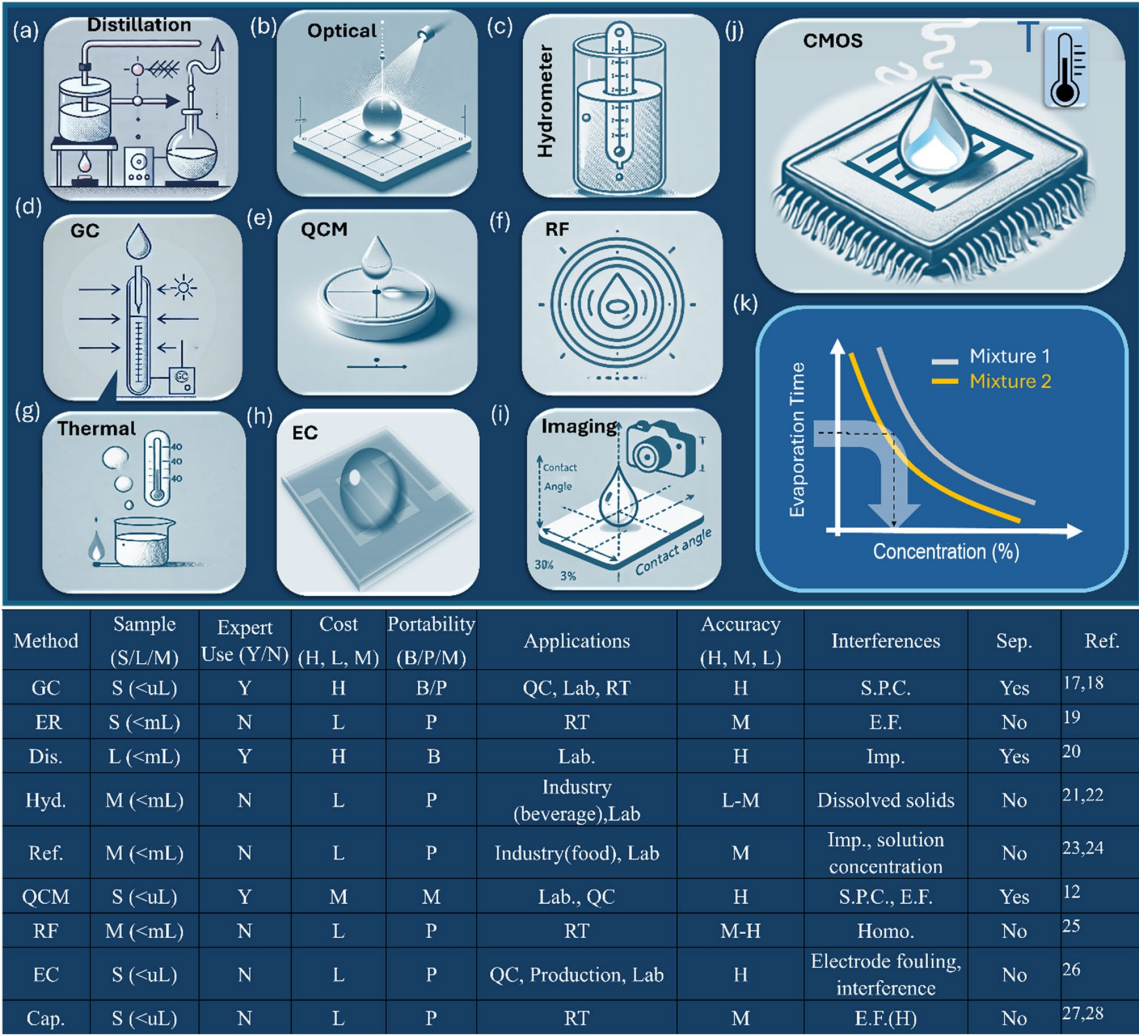
Comparative overview of various techniques for analyzing binary liquid mixtures based on evaporation dynamics and physical interaction mechanisms. Panels (**a**–**j**) illustrate the core principles of each method, including gas chromatography (GC), thermal analysis, distillation, hydrometry (Hyd.), spectroscopy (Spectro.), quartz crystal microbalance (QCM), radio frequency (RF), electrochemical (EC), imaging-based contact angle analysis, and CMOS-based capacitive sensing (Cap.). Panel (**k**) schematically shows the inverse relationship between evaporation time and solute concentration for two representative mixtures. Panel (**l**) summarizes the methods by key criteria including sample volume, expertise required, cost, portability, application context, accuracy, dominant error sources, and core sensing mechanism. CMOS capacitive sensing (**j**) offers notable advantages such as small sample size, high sensitivity, low cost, real-time response, and minimal need for separation, making it well-suited for compact and portable evaporation monitoring platforms.

The precise determination of alcohol concentration in aqueous mixtures is essential across numerous industries, including pharmaceuticals, food and beverage production, fuel cell, environmental monitoring, and chemical research^1–4^. Over the years, a wide range of analytical methods has been developed, each offering distinct advantages and facing specific limitations related to accuracy, cost, scalability, and operational simplicity, as illustrated in Fig. 1. Traditional methods such as distillation are known for their high precision, yet they are energy-intensive and unsuitable for real-time or high-throughput applications. In contrast, modern sensor-based techniques—including gas chromatography (GC), spectroscopy, thermal analysis, and advanced electrochemical systems—offer improved sensitivity and faster response times, though they often lack portability and may be affected by environmental conditions^3,5,6^.

Distillation remains one of the most accurate methods, but is impractical for field use due to its complexity and resource demands^7,8^. Density-based techniques like hydrometry are widely accessible and cost-effective but are prone to errors from impurities and temperature fluctuations. More sophisticated methods, such as GC and spectroscopic techniques (IR, UV–Vis, Raman), provide high sensitivity for analyzing complex mixtures but require specialized equipment and trained personnel. Similarly, thermal techniques, quartz crystal microbalances (QCM), and radio frequency (RF) sensing have shown promise for high-sensitivity and real-time detection, yet their deployment is hindered by the need for careful calibration and vulnerability to environmental interferences^9–13^.

Figure 1 summarizes the comparative performance of these approaches across key parameters: measurement accuracy (high (H), medium (M), low (L)), operational ease (expert-dependent vs. user-friendly), sample volume requirements (small (S), medium (M), large (L)), instrument cost (low (L), medium (M), high (L)), and portability (bulky (B), desktop (D), or portable (P)). These methods are employed in contexts ranging from industrial quality control (QC) and real-time (RT) monitoring in manufacturing to laboratory research and academic instruction. One established method for evaluating droplet behavior based on thermal properties is Thermogravimetric Analysis (TGA). TGA is a thermal analysis technique that measures the mass loss of a substance under controlled heating, enabling direct determination of evaporation time by identifying the onset and endpoint of volatile component loss^14–16^. It is particularly effective for studying both single solvents and binary mixtures such as water–ethanol or water–methanol. While TGA typically tracks mass loss, our approach employs a capacitive sensor to detect the presence of the droplet and empirically determine the Time of Evaporation (ToE). TGA curves (mass vs. time or temperature) reveal critical thermal characteristics, including evaporation rate, phase transitions, and residual mass. These curves can be further analyzed using computational models to extract thermodynamic parameters such as the heat of vaporization (ΔHvap), often through kinetic models like the Arrhenius equation or zero-order kinetics, as well as the Clausius–Clapeyron relationship.

TGA is widely used in materials science, pharmaceuticals, and environmental studies for characterizing solvent evaporation and drying behavior^17,18^. However, given the computational complexity of these multiparameter models (see Supplementary Information (SI), Section: Analytical Modeling of Binary Droplet Evaporation, Eqs. (7) to (17), Figs. S21 and S22), we adopt an empirical approach in this study by developing a practical measurement setup based on capacitive sensing.

The table also categorizes the fundamental measurement mechanisms underpinning each method, including differences in volatility and boiling point, buoyancy, light absorption and scattering, piezoelectric frequency shifts, and dielectric variation across frequency domains, as depicted in Fig. 1. However, these techniques are susceptible to several sources of error. Contamination during sample preparation can affect GC and QCM accuracy, while ambient temperature and humidity may impact thermal, electrochemical, and capacitive systems. Additionally, impurities can degrade the precision of distillation and spectroscopic methods, and concentration inhomogeneities can influence RF sensor readings.

Studies have also examined the evaporation rate (ER) of alcohols—such as *n*-butanol—from aqueous mixtures. Morikawa et al. employed a custom air tunnel and manometer-based system to facilitate and quantify evaporation^19^. Dilling et al. analyzed the ER of chloromethanes, ethanes, ethylenes, propane, and propylenes using a hollow fiber-mass spectrometric approach^20^. Despite these advancements, accurate measurement of ER in aqueous solutions under varying conditions remains challenging. Recent efforts have focused on clarifying the physicochemical parameters influencing evaporation and on enhancing measurement techniques^21^. For example, Combe et al. used optical microscopy to measure droplet diameters and infer ER^21^, while Misyura et al. demonstrated the application of thermal imaging to assess evaporation dynamics in water and salt-containing solutions^22^. Although droplet size change has become a common proxy for evaporation, the integration of chemical or biological sensing based on ER or total ToE remains relatively underexplored.

Based on our knowledge, due to the practical challenge of precisely measuring the evaporation time of low concentration droplets, there have been limited studies reported in this area, especially for applications such as chemical and biological sensing.

To help overcome these challenges, this work utilized two structures of CMOS capacitive sensors for the measurement of evaporation time in low concentration of droplet solutions and the analysis of binary mixture concentrations. The alcohol shows a lower boiling point and higher volatility than water, which causes faster evaporation compared to water. The evaporation of water-alcohol mixtures’ droplets with different concentrations of alcohol has been widely studied^23–27^.

CMOS biosensors perform as transducers that convert biological information into electronic signals, combining the advantages of integrated circuit technology with the sensitivity of biological detection^28^. As the demand for efficient diagnostic tools increases, researchers have developed various CMOS biosensor designs tailored for a wide range of applications, making them a versatile platform for point-of-care testing, environmental monitoring, and biomedical applications^29–31^. Certainly, the field of CMOS biosensors has seen a multitude of techniques proposed for droplet analysis, reflecting the ongoing advancements in microelectronics and biotechnology.

CMOS biosensors can be categorized based on their detection mechanisms and the types of target analytes. For instance, optical CMOS biosensors, employing light-based techniques to quantify fluorescence, absorbance changes, or evanescent field in biological samples, enabling the detection of specific biomolecules at very low concentrations^32–35^. Notably, in optical fluorescence-based sensors, the information is created by the reactions between the fluorescence label and target and transduction into optical signals. In addition to optical sensors, magnetic CMOS sensors use magnetic micro- and nanoparticles as labels to detect target analytes by utilizing variations in magnetic fields^36–38^. Labeling for optical and magnetic sensors is often expensive and time-consuming, and it can also impact the natural habitat of bio-particles.

In contrast, electrochemical sensing techniques are regarded as real-time measurement, label-free, and low complexity methods that can detect biochemical changes at the electrode-solution interface^39–41^. These techniques are highly compatible with standard CMOS technology, allowing the top metal layer of CMOS to serve as electrodes without requiring additional post-processing or external components. The term “electrochemical measurement technique” encompasses various methods, including potentiometry^42^, impedimetry^43,44^, amperometry^44–46^, conductometry^47,48^, and capacitance measurement^49–54^.

In the realm of electrochemical sensing techniques, capacitive sensors directly measure the capacitance and bypassing the need to derive it from impedance measurements^55^. Capacitive sensing, leveraging the inherent capacitance changes induced by droplets, has been a prominent approach, offering high sensitivity and accuracy. For example, in the work by Isgor et al*.*^56^, a microfluidic system incorporating capacitive sensors for detecting the content of droplets is proposed. The concept revolves around the change in the dielectric constant of the droplet content influencing the capacitive signal. Mohammadi et al*.*^57^, introduced a differential ring-oscillator sensing platform for nanodroplet detection. The sensing mechanism described in the article relies on the dielectric constant variations of an interdigitated capacitor that is integrated into a ring-oscillator structure.

**Fig. 2:**
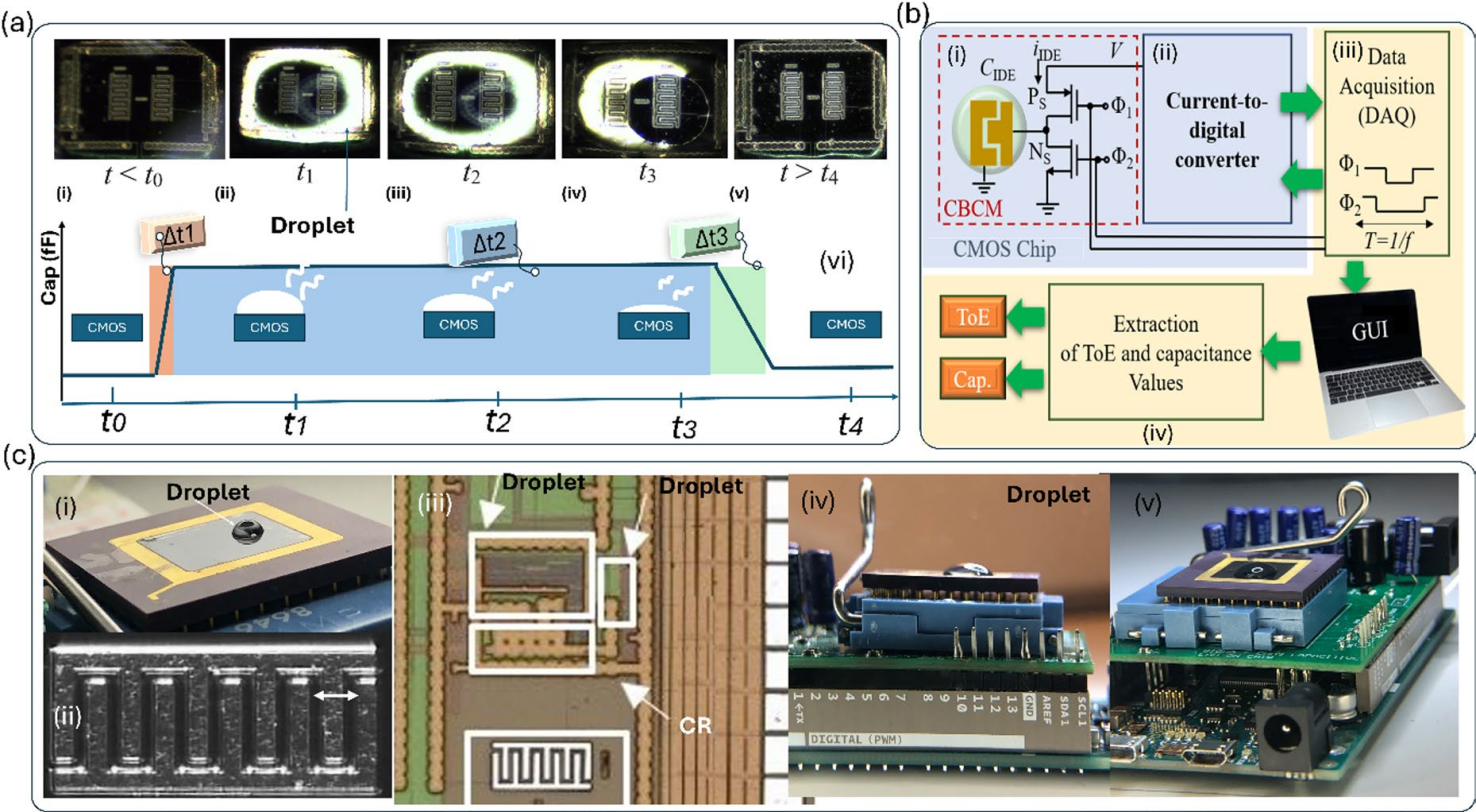
CMOS-based capacitive sensing platform for droplet evaporation monitoring: (**a**) time-lapse images of a droplet evaporating over IDEs, with corresponding schematic representation of evaporation phases (Δt₁, Δt₂, Δt₃) across different sensor regions, (**b**) The system diagram illustrates signal processing from the (i) CBCM through a (ii) CCO and (iii) DAQ, enabling extraction of (iv) ToE and capacitance values displayed via a GUI, (**c**) experimental setup, including (I) the droplet on the sensor surface, (ii) microphotograph of IDE, (iii) microchip layout, (iv) chip packaging, and integration onto a (v) PCB with microcontroller and USB interface for computer connectivity.

One of the popular topology of capacitive sensor is name as charge-based capacitance measurement (CBCM) that is shown Fig. 2b, which presents a compelling solution with its advantageous blend of high accuracy and low complexity, rendering it particularly well-suited for lab-on-chip (LoC) applications^58^. Within our group’s extensive research portfolio, we have unveiled a series of core-CBCM capacitive sensors explicitly crafted for monitoring a spectrum of liquid samples characterized by diverse dielectric constants, hydrogel^59^, encompassing water^60^, ethanol^61^, methanol^60–62^, propanol^60^, dichloromethane^60,62^, and acetone^60,62^.

## Proposed method

This section presents the proposed measurement method and the implemented CMOS interface circuit.

### ToE measurement

The evaporation of a droplet on interdigitated electrodes (IDEs) induces capacitance changes on the order of femtofarads (fF). The IDEs, measuring 220 µm × 110 µm, are integrated with the underlying readout circuitry and fabricated using 350 nm CMOS technology (AMS process). As illustrated in Fig. 2a, a droplet is placed on top of the IDEs located on the CMOS chip surface. The resulting capacitance variations are precisely measured by a CMOS interface circuit, generating a clear capacitive signal as shown in Fig. 2b. The sensor exhibits three distinct operational states: (i) when no droplet is present, the capacitance is at its minimum value (C_min_); (ii) when the droplet height exceeds the sensor’s screening length (SL), the capacitance reaches its maximum value (C_max_); and (iii) when the droplet height falls below SL, the capacitance returns to C_min_. Initially, during phase I, the system maintains C_min_. Upon droplet application, the capacitance increases over a time interval Δt₁, transitioning into phase II, where it stabilizes at C_max_ during Δt₂ as long as the droplet’s thickness (τ) remains above SL. As the droplet evaporates and τ drops below SL, the capacitance decreases over Δt₃, completing the cycle by returning to C_min_. The overall duration of the capacitance transition correlates with the droplet’s evaporation time, enabling high-resolution temporal monitoring of microdroplet behavior. The presence of different liquids with varying ToE results in distinct capacitive signal profiles, as shown in Fig. 2c. For example, a higher concentration of alcohols such as methanol in water leads to shorter transient durations—specifically, reduced Δt₁, Δt₂, and Δt₃—due to faster evaporation rates. In other words, a correlation is expected between alcohol concentration and the duration of transient phases, providing a potential means for distinguishing liquid composition based on capacitive response. Variations in sample properties inherently lead to changes in their dielectric characteristics, which in turn affect the capacitance of the IDEs and are captured by the CMOS interface circuit. In other words, the capacitive sensor is capable of detecting both dielectric property variations and evaporation dynamics (ToE). This dual functionality positions the system as a dual-mode sensing device, capable of providing complementary information for material identification and characterization. To further elucidate the role of SL, COMSOL simulations were conducted, as detailed in the SI (Section: Numerical Simulations, Figs. S18 and S19).

### Interface circuit

The structure of the CMOS capacitive sensor for droplet sensing is illustrated in Fig. 2b. The sensor integrates a charge-based capacitance measurement (CBCM) module that transforms the differential capacitance (C_Sen_–C_Ref_) into a current signal. This current is then fed into a current-controlled oscillator (CCO), which converts it into a frequency signal. A counter digitizes the frequency, resulting in a digital output linearly proportional to the capacitance difference. To interface the chip with a computer, a data acquisition system (DAQ) is employed, and a graphical user interface (GUI) is implemented to monitor evaporation time by analyzing the time-dependent capacitance. The reference capacitance, C_Ref_, is realized through a programmable capacitor bank consisting of eight capacitors ranging from 10 fF to 1.27 pF, with 10 fF resolution. This configuration compensates for residual capacitance introduced by sample remnants on the sensor surface. As shown in Fig. 2c, the sensor is integrated on-chip with another circuitry. Figure 2c(i) depicts the chip encapsulated in epoxy, with bonding pads and wires protected, except for a cavity that allows direct droplet placement for sensing demonstrations. The chip supports serial input and output; the serial output transmits digitized capacitance values, while the serial input is used to control the reference capacitor bank, allowing fine-tuned sensor calibration. A microcontroller (µC) is programmed to interface with the CMOS chip, mounted in a socket on a printed circuit board (PCB), as seen in Fig. 2c(v). This PCB includes a voltage regulator and level shifters to match the operating voltages of the chip and microcontroller. The µC communicates with a computer via USB, enabling real-time readout through the GUI. Further details of the CMOS interface circuit are provided in the SI (Section: CMOS Interface Circuit, Fig. S20, and Eqs. (1) to (6)).

## Results

The droplet electroporation profiles, transient phase analysis, and optimization process are discussed in this section. Also, the details of statistical data analysis are provided in SI (Section: Statistical Analysis, Figs. S1 to S17 and Tables S1 to S6).

### Evaporation profile mapping across binary mixtures and thermal conditions

**Fig. 3:**
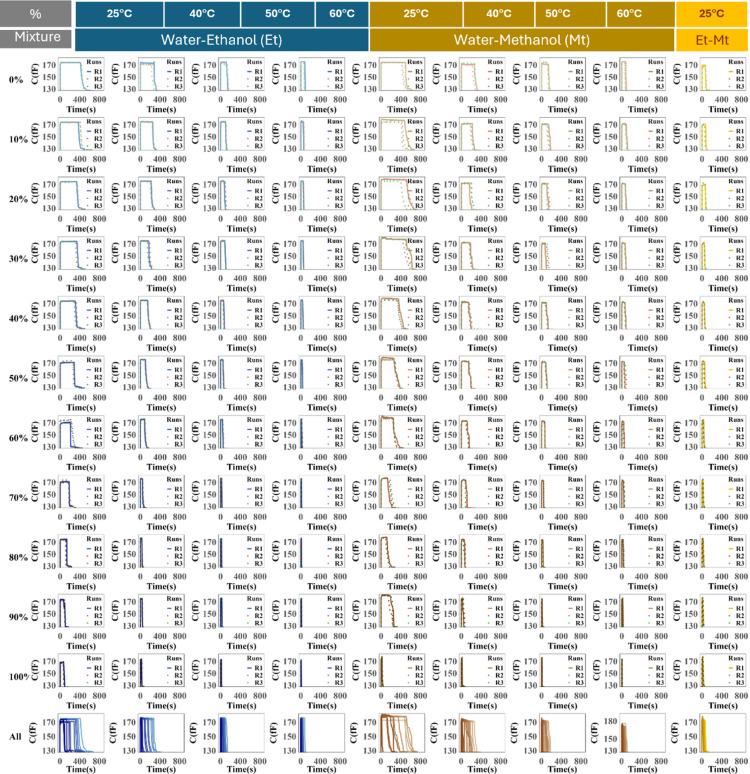
Evaporation profiles of binary liquid mixtures measured using an IDE sensor at varying concentrations and temperatures. Each row corresponds to a specific mixture concentration (0% to 100%), while each column represents a different temperature (25 °C, 40 °C, 50 °C, and 60 °C) for three mixture types: water–ethanol (blue), water–methanol (orange), and ethanol–methanol (yellow). For each condition, 1 μL of the mixture was deposited on the IDE surface, and the sensor response was recorded over time. The decay in signals reflects the change in dielectric properties due to evaporation. Each plot displays three replicates to assess repeatability. The bottom row (“All”) shows the combined or average response across all concentrations for each condition.

After preparing binary mixtures of water–methanol, water–ethanol, and methanol–ethanol with concentrations ranging from 0 to 100%, we conducted experiments by introducing 1 μL of each mixture onto the IDE. The sensor platform was then placed in an incubator set to different temperatures: room temperature, 40 °C, 50 °C, and 60 °C. For each concentration and binary combination, the experiment was repeated three times to ensure reproducibility, as illustrated in the figure below. As shown in Fig. 3, increasing temperature accelerates both the rate and sharpness of capacitance changes, particularly at higher solvent concentrations, underscoring the strong influence of thermal effects on system dynamics. The final row in each column displays the first experimental run (R1) under each condition, serving as a baseline reference for comparative analysis.

Figure 3 provides a comprehensive visualization of time-dependent capacitance behavior across different binary mixtures, concentration levels (0% to 100%), and temperatures (25 °C, 40 °C, 50 °C, and 60 °C). The y-axis represents capacitance, while the x-axis shows time. Each vertical block of four columns corresponds to a specific mixture type: water–ethanol (W-Et), water–methanol (W-Mt), and ethanol–methanol (Et-Mt), with each column within a block reflecting a distinct temperature. The systematic structure facilitates direct observation of how each parameter influences sensor response.

For water–ethanol mixtures, located in the first four columns, capacitance profiles show clear concentration- and temperature-dependent trends. At 25 °C, the response begins with a stable phase, followed by a gradual rise. As ethanol concentration increases, the transition from stability to rapid capacitance increase becomes more abrupt, suggesting stronger evaporation-driven interactions. At 40 °C and 50 °C, this transition occurs sooner, with steeper slopes, indicating enhanced solvent volatility and interaction. At 60 °C, these effects are magnified, especially for high ethanol concentrations, where the capacitance shift is almost instantaneous.

For water–methanol mixtures (columns five to eight), a similar but slightly moderated behavior is observed. At 25 °C, transitions are slower and more gradual, indicating weaker initial interaction compared to ethanol–water systems. As temperature rises to 40 °C and beyond, the rate of capacitance change increases, with higher methanol concentrations producing sharper transitions. At 60 °C, capacitance profiles become steep and rapid, especially in high methanol cases, again emphasizing the thermal enhancement of droplet evaporation and dielectric change.

The final column shows ethanol–methanol mixtures at 25 °C. These mixtures exhibit more gradual and balanced capacitance changes compared to water-based systems. Initial stability gives way to a slow transition, with only moderate sharpness observed even at high ethanol concentrations. This behavior suggests less volatile interaction between ethanol and methanol at ambient temperature relative to water–solvent systems.

Overall, the data in Fig. 3 clearly demonstrate that capacitance evolution is highly dependent on the mixture type, solvent concentration, and ambient temperature. Ethanol–water mixtures display the most rapid and pronounced capacitance shifts, followed by methanol–water, while methanol–ethanol systems show the most gradual changes. Temperature acts as a key amplifier, enhancing evaporation rates and dielectric shifts. The inclusion of first-run (R1) data in the last row of each column provides a consistent reference point for evaluating repeatability and baseline behavior across conditions.

**Fig. 4:**
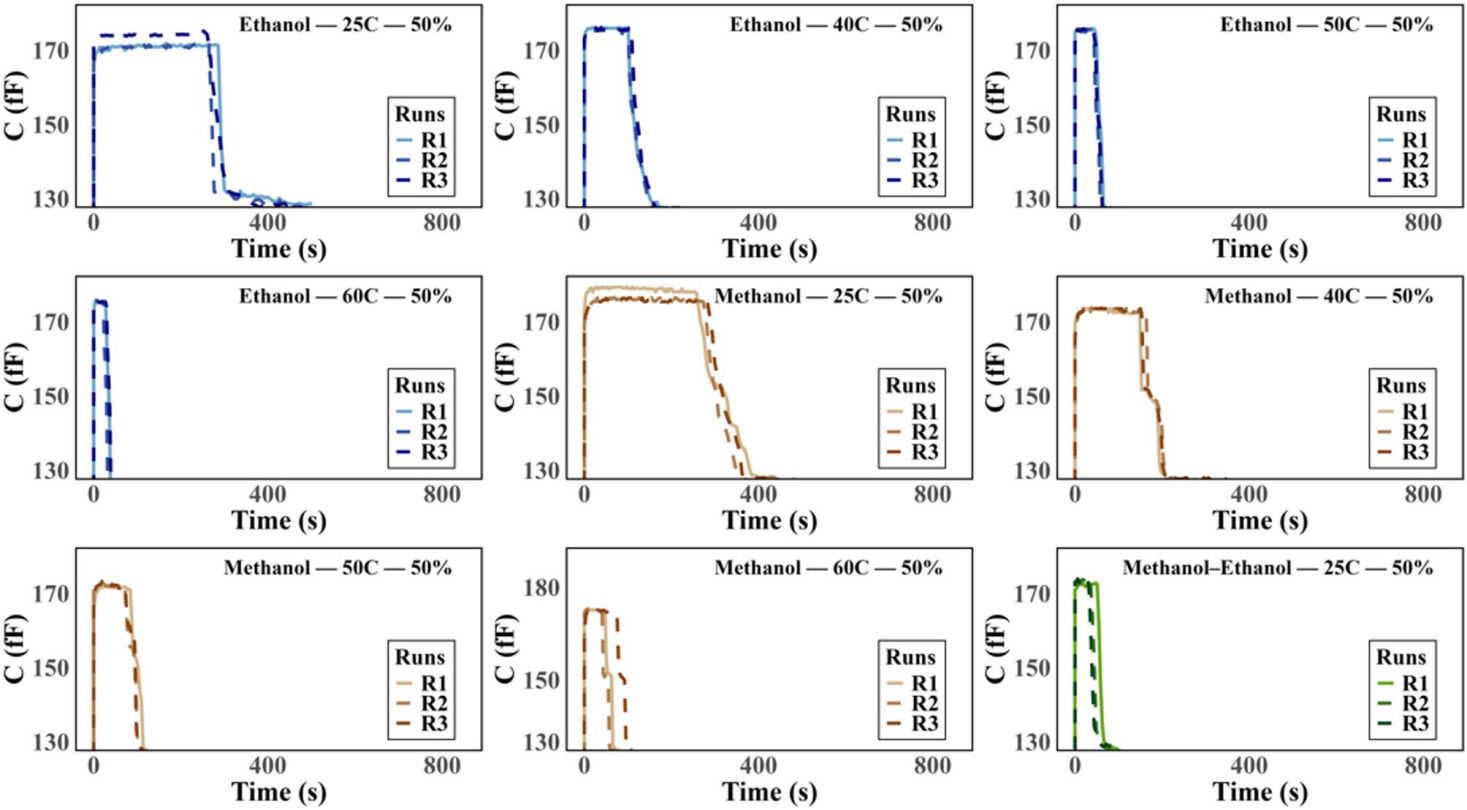
The time-aligned capacitance curves for ethanol–water, methanol–water, and methanol–ethanol mixtures measured between 25 °C and 60 °C, each with a solvent concentration of 50% in the binary mixtures.

For clarity, one row from Fig. 3 is highlighted in Fig. 4, which presents the time-aligned capacitance curves for ethanol–water, methanol–water, and methanol–ethanol mixtures measured between 25 °C and 60 °C, each at a 50% concentration in the respective binary mixtures. Each panel includes three repeated runs (R1–R3) to demonstrate the consistency of the responses. In all mixtures, the capacitance first rises sharply, remains briefly steady, and then drops as evaporation completes. With increasing temperature, this process occurs faster, shortening the steady-state period. The close overlap of R1–R3 confirms reproducibility, while differences between mixtures and temperatures highlight the sensor’s sensitivity to composition and thermal effects. These trends are consistent with the numerical results presented in SI, where Δt₂, ToE, and ΔCap decrease with temperature and vary with solvent type.

### Transient phase analysis and model fitting of evaporation dynamics in binary solvent systems

**Fig. 5:**
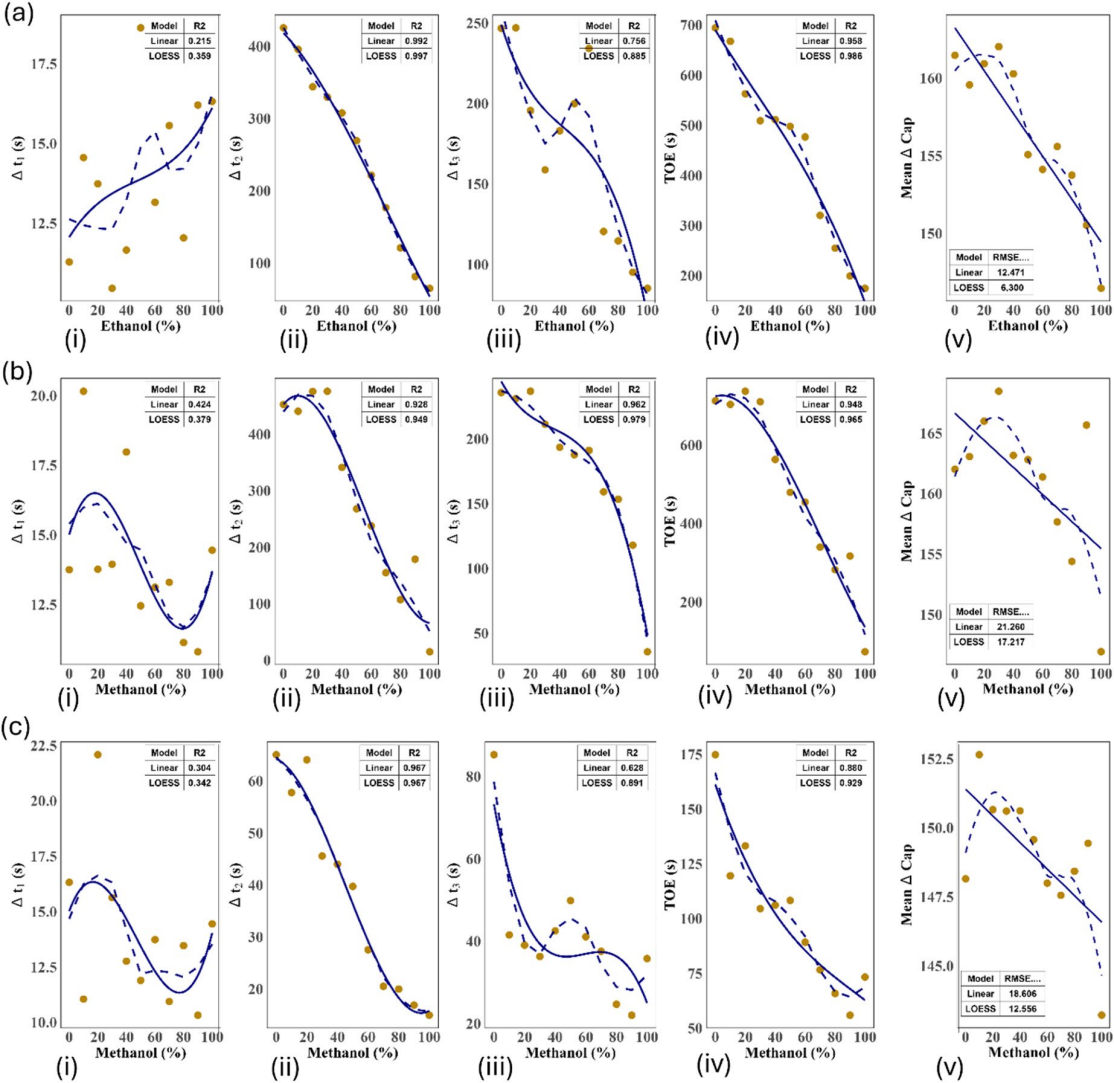
Quantitative analysis of evaporation behavior for three binary solvent systems—(**a**) ethanol–water, (**b**) methanol–water, and (**c**) methanol–ethanol—across a range of solvent concentrations (0–100%). Each column displays a specific evaporation-related parameter plotted against solvent concentration: (i) initial evaporation interval (Δt₁), (ii) intermediate evaporation interval (Δt₂), (iii) total evaporation time (ToE), (iv) final evaporation interval (Δt₃) and (v) mean capacitance change (ΔCap). Experimental data (orange dots) are fitted using both linear regression (dashed blue) and LOESS smoothing (solid blue).

Figure 5 of ethanol–water, methanol–water, and methanol–ethanol systems at 25 °C across four evaporation phases—Δt₁, Δt₂, Δt₃, and ToE—alongside mean capacitance change (ΔCap). Linear regression (dashed blue) and LOESS smoothing (solid blue) models are applied to each dataset. The LOESS model consistently provides superior fitting accuracy, particularly in capturing non-linear trends in Δt₁ and ΔCap. Evaporation time generally decreases with increasing alcohol concentration, reflecting enhanced volatility, while dielectric changes correlate with shifts in mixture composition. Insets the report R2 or RMSE values to compare model performance.

The ethanol–water system (row (a)) demonstrates distinct concentration-dependent behaviors across all time zones. For Δt₁ (a-i), both models detect an upward trend in early-phase evaporation time with rising ethanol content, but LOESS captures subtle inflection points more effectively (R2: LOESS > Linear). In Δt₂ (a-ii), evaporation duration decreases steadily with increasing ethanol concentration, a trend captured equally by both models, though LOESS maintains a slight advantage. For ToE (a-iii) and Δt₃ (a-iv), the decline is more pronounced, reflecting ethanol’s higher volatility, with LOESS offering a smoother and more consistent fit. The dielectric response (a-v) shows an initial drop in ΔCap followed by stabilization, indicating concentration-induced dielectric shifts; LOESS again models this behavior more accurately, as shown by lower RMSE.

The methanol–water system (row (b)) displays similar but less pronounced behavior. Δt₁ (b-i) lacks a clear monotonic trend, and both models yield lower R2 values, reflecting variability in early-stage evaporation. In contrast, Δt₂ (b-ii) and ToE (b-iii) exhibit strong negative trends, with LOESS achieving high R2 values, highlighting its ability to capture non-linearity in phase transition timing. Δt₃ (b-iv) follows a consistent downward trajectory, and LOESS again tracks the curve more accurately. Capacitance change (b-v) decreases with increasing methanol concentration, where the LOESS fit better reflects the non-uniform response compared to the linear model.

In the methanol–ethanol system (row (c)), evaporation behavior becomes more complex. Δt₁ (c-i) presents irregular trends with poor model fits (low R2), suggesting that early-phase dynamics in this system are highly variable. However, Δt₂ (c-ii) and ToE (c-iii) show stronger, monotonic decreases in response to concentration, where LOESS outperforms linear fitting, capturing inflection zones and curvature. Δt₃ (c-iv) continues this pattern, reinforcing the trend. The capacitance change (c-v) exhibits a subtle but nonlinear decline, once again modeled more effectively by LOESS with reduced RMSE.

In summary, across all systems and measurement phases, the LOESS model consistently provides superior fitting, particularly where non-linearity is evident. This is especially notable in Δt₁ and ΔCap, where linear models fail to capture inflection behavior. The ethanol–water system shows the clearest overall trends, with LOESS performing exceptionally well in Δt₂ and ToE. For the methanol–water system, LOESS excels in modeling mid-to-late phase evaporation times (Δt₂ and ToE), while the methanol–ethanol system benefits from LOESS’s robustness in handling more complex transitions. These findings underscore the importance of non-linear modeling in accurately analyzing evaporation dynamics and dielectric changes in mixed-solvent systems.

### Temperature optimization for linearity and dynamic range in binary solvent evaporation sensing

**Fig. 6:**
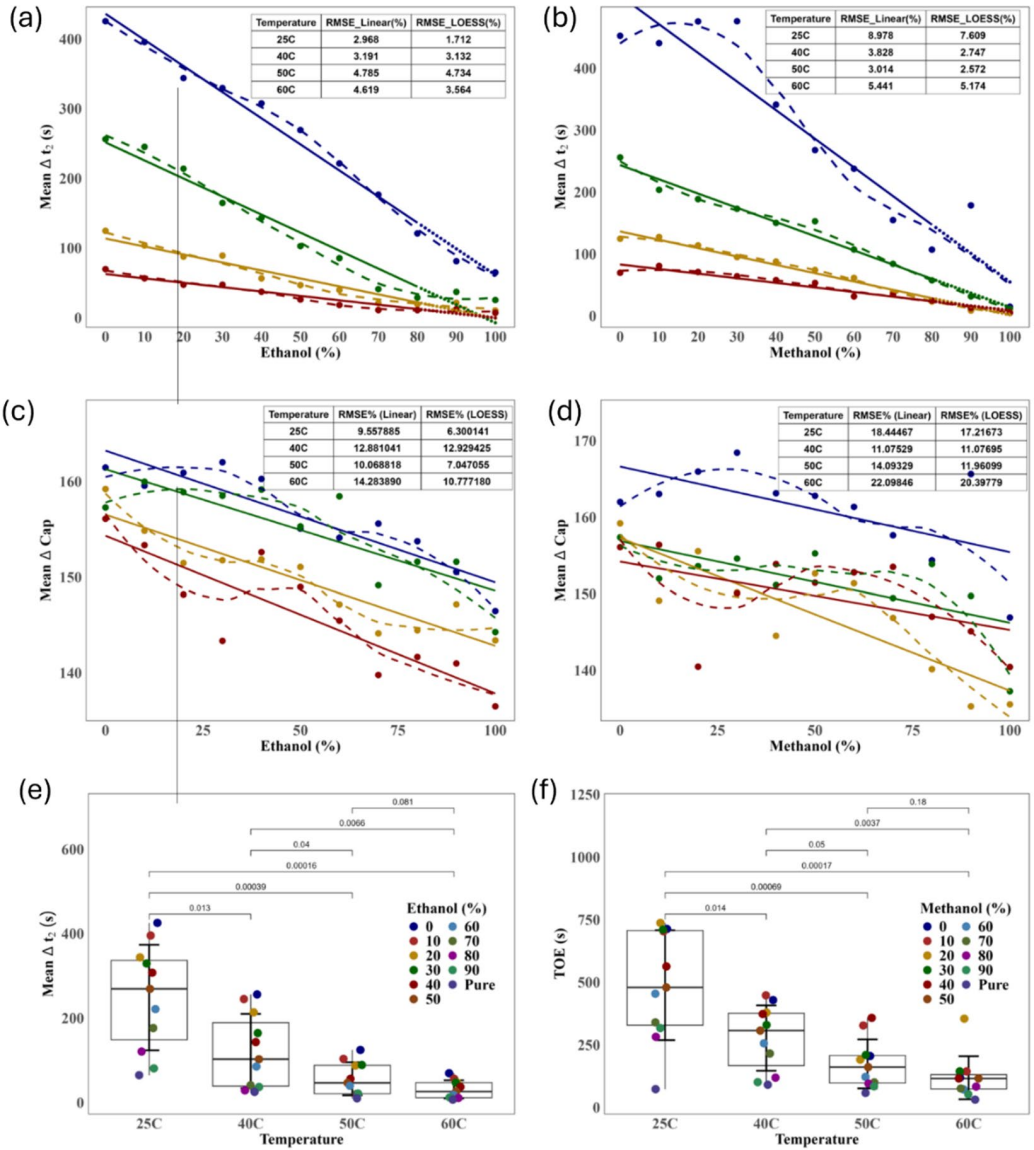
Quantitative analysis of solvent evaporation dynamics and dielectric response for ethanol–water and methanol–water mixtures across varying concentrations and temperatures; (**a**, **b**) Mean intermediate evaporation time (Δt₂) versus ethanol (**a**) and methanol (**b**) concentrations at four temperatures: 25 °C (blue), 40 °C (green), 50 °C (orange), and 60 °C (red). LOESS (solid lines) and linear regression (dashed lines) models are fitted to the experimental data points. Insets show that LOESS consistently yields lower RMSE values, particularly at lower concentrations and higher temperatures; (**c**, **d**) Mean capacitance change (ΔCap) as a function of ethanol (**c**) and methanol (**d**) concentration, again showing improved fit quality with LOESS. Capacitance values generally decrease with rising alcohol content and temperature, reflecting dielectric property shifts during evaporation; (**e**, **f**) Boxplots showing temperature-dependent trends: (**e**) Δt₂ for ethanol–water mixtures and (**f**) ToE for methanol–water mixtures, across selected concentrations. Statistical comparisons (annotated *p*-values) confirm that temperature significantly impacts both evaporation rate and dielectric behavior.

Figures 6a and 5f provide a detailed analysis of the evaporation behavior (Δt₂) and dielectric response (ΔCap) of ethanol–water and methanol–water binary mixtures across varying concentrations and temperatures. The top row of the figure illustrates how the intermediate evaporation time (Δt₂), corresponding to the plateau phase in the capacitance signal, is affected by both solvent composition and thermal conditions. In ethanol–water systems, Δt₂ consistently decreases with increasing ethanol concentration, particularly at elevated temperatures (50 °C and 60 °C), where the decline becomes steeper. This trend reflects the increased volatility and faster transition through the evaporation phases in ethanol-rich mixtures. Methanol–water mixtures exhibit a similar downward trend in Δt₂ with increasing methanol concentration, but the slope is gentler, suggesting a less aggressive evaporation behavior compared to ethanol under the same thermal conditions.

Comparative model performance highlights the strength of the LOESS approach. At all temperature points, the LOESS model achieves substantially lower RMSE values than the linear model, especially in high-concentration regimes where the relationship between Δt₂ and solvent composition deviates from linearity. This demonstrates the importance of applying non-parametric modeling when dealing with complex, thermally sensitive fluid dynamics.

In the middle row, the dielectric response of the mixtures, expressed as mean capacitance change (ΔCap), further reinforces the concentration- and temperature-dependent behavior. For ethanol–water mixtures, ΔCap decreases progressively as ethanol concentration increases. This reduction in capacitance is likely resulting from the lower dielectric constant of ethanol compared to water, as well as from accelerated evaporation rates at higher alcohol levels and temperatures. The effect is more pronounced at 50 °C and 60 °C, suggesting that increased thermal energy amplifies the dielectric sensitivity of the system. Methanol–water mixtures follow the same general pattern, though the slope of capacitance decline is again milder compared to ethanol–water, consistent with methanol’s intermediate volatility and dielectric properties.

The model comparison across all capacitance data further confirms the superiority of LOESS in capturing non-linear interactions. RMSE values for LOESS are consistently lower than those for linear regression, particularly at the extremes of the concentration range and under higher thermal conditions. This suggests that the underlying physical processes governing both evaporation and dielectric shift are inherently non-linear, making LOESS a more appropriate model for capturing subtle variations that linear models cannot represent.

Finally, the boxplots in the bottom row (Fig. 6e, f) statistically validate the observed trends. For ethanol–water mixtures, Δt₂ significantly decreases with increasing temperature across most concentrations, with several comparisons showing highly significant *p*-values (*p* < 0.001). A similar pattern is observed for methanol–water mixtures in ToE, confirming that temperature acts as a dominant factor in accelerating evaporation dynamics.

Collectively, these findings emphasize the dual role of concentration and temperature in shaping both the kinetic and dielectric behavior of binary liquid mixtures. The results also underline the importance of non-linear modeling techniques, such as LOESS, for accurately representing evaporation dynamics in capacitive sensing platforms. These insights are crucial for optimizing sensor performance in practical applications where temperature fluctuations and mixture composition vary in real-time conditions. Further data analysis can be found in the SI, under the Sections “Statistical Analysis” and “Extra Statistical and Artificial Intelligence Analyses”.

## Conclusion

This study presents ITEMS, a CMOS-based capacitive sensing platform capable of extracting time-resolved evaporation dynamics and dielectric responses for accurate characterization of binary liquid mixtures. By analyzing key temporal parameters such as Δt₂ and ToE, along with capacitance changes, the system reliably distinguishes between solvent compositions across a range of temperatures and concentrations. The superior performance of LOESS modeling further highlights the non-linear nature of solvent interactions and supports the system’s high sensitivity and analytical robustness. With its compact form factor, low power consumption, and minimal sample requirements, ITEMS offers a scalable and portable solution for real-time chemical monitoring. Future work will focus on extending the platform for multi-analyte detection, integration with microfluidic channels, and deployment in environmental and biomedical sensing applications where continuous, in situ monitoring is critical.

From a statistical perspective, future work will focus on improving data analysis and verification. We plan to test new methods for predicting solvent concentration more accurately and automatically, with each instrument version evaluated through clear statistical checks, including comparison of predicted versus measured values, calculation of remaining error, and repeated tests to ensure consistency over time.

We will also examine how temperature, repeated use, and different solvent mixtures affect measurement stability. Long-term robustness under diverse environmental conditions will be assessed, sensor materials will be optimized for sensitivity and selectivity, and potential applications beyond the current scope will be explored. For example, in biomedical contexts, this method could monitor the evaporation of small volumes of biological fluids, such as saliva or sweat, offering insights for non-invasive diagnostics. Miniaturization and integration of on-chip transceivers will enhance portability for use in remote laboratories, field sites, clinical settings, or wearable/implantable sensors.

Future efforts may include implementing algorithms for real-time concentration detection and exploring wireless or cloud connectivity. By combining these developments with rigorous statistical validation, the platform is expected to become a versatile, high-performance tool capable of providing accurate, reproducible, and practical measurements in real-world applications.

## Methods

### Chip fabrication and packaging

The sensor chip was implemented by 0.35 µm CMOS technology and packaged by commercial CPGA85, as seen in Fig. 2c(i). The bonding wires were encapsulated by a UV-cured epoxy resin to avoid direct contact with the liquid. The electrodes were implemented on the topmost metal layers of the technology (metal 4). The employed chip includes two 220 × 110 µm^2^-IDEs passivated by SiO_2_ (see Fig. 2c(ii)) connected to two independent readout circuits^12^.

The sensing electrodes are protected by a thermally grown SiO₂ passivation layer that completely isolates the metal layers from direct contact with the liquid sample. SiO₂ is chemically inert to the tested compounds (water, ethanol, and methanol) and effectively prevents any surface reactions or corrosion. Under our experimental conditions (1 μL liquid droplets, ambient to 60 °C, short contact times), SiO₂ remains chemically stable and does not undergo covalent reaction with methanol or ethanol.

According to well-established studies on amorphous silica surface chemistry^63–65^, alcohol molecules primarily interact with silica through physical adsorption or hydrogen bonding to surface silanol groups rather than by consuming or chemically transforming the oxide. It should be noted that decomposition or stronger surface chemistry of alcohols on silica has been reported only under special conditions, such as metal-impurity-containing, or activated surfaces, and ultrahigh vacuum or low-temperature experiments^66^, which differ entirely from our passivated, packaged CMOS chip configuration.

During our experiments, no chemical degradation or performance drift was observed, even after multiple consecutive measurements on the same chip. The capacitance profiles remained consistent across three repeated trials for each condition (as shown in Fig. 3), confirming the stability and reusability of the passivated surface. This SiO₂ layer is explicitly designed to ensure long-term sensor reliability and to maintain dielectric consistency during droplet evaporation.

### Sample preparation and experimental methodology

In this study, the proposed capacitive ToE sensing method was tested using ethanol and methanol. Different concentrations of the two types of alcohol and water were mixed to assess the accuracy of the proposed concept. The experiments involved introducing a specific volume of the sample containing x% of liquid 2 and (100-x)% of liquid 1 to the sensor, where x ranges from 0 to 100 with increments of 10. The experiments were performed using water–ethanol (W-Et), water–methanol (W-Mt), and ethanol-methanol (Et-Mt) mixtures, which were prepared using a pre-calibrated micropipette.

Each experiment was independently repeated three times (denoted as R1, R2, and R3) to ensure reproducibility. For every mixture concentration and temperature, a new 1 µL droplet was deposited on the IDE and recorded until full evaporation. After completion of one measurement, the surface was cleaned and the next repetition was performed under identical environmental conditions. Since both water and alcohol are highly volatile, no extensive cleaning step was required between measurements, as the previous sample evaporated completely before introducing the next mixture, minimizing any risk of cross-contamination. Nevertheless, the sensor was briefly rinsed with isopropyl alcohol between measurements to remove any residual sample and further prevent cross-contamination.

### Statistical data analysis

Statistical analysis was conducted to evaluate the trends and behavior of ethanol–water, methanol–water, and methanol-ethanol mixtures across various temperatures and concentrations. The analysis utilized both linear regression and LOESS models to capture linear and non-linear patterns in the data. The LOESS model, originally introduced by^67^ is a non-parametric regression technique that fits a smooth curve to data by locally weighting neighboring points within a defined span. This approach makes LOESS particularly effective for identifying and modeling non-linear relationships, especially in datasets with complex variability. RMSE values were calculated to assess the accuracy of these models, with LOESS consistently outperforming the linear model, especially in cases of non-linear trends. The comparison of Δt_2_ (time interval during the flat cap phase), capacitance, and total ToE across different mixtures and temperatures provided insights into the dynamic interactions within these systems. The statistical computation and visualization were implemented **R version 4.4.1**, using widely accepted packages. To validate the statistical assumptions for comparing temperatures, normality and equality of variance tests were performed. The Shapiro–Wilk test^68^, implemented using the nortest package in R, confirmed that the data followed a normal distribution for all temperatures, as indicated by non-significant *p*-values. However, the equality of variance tests^69^, conducted using the Bartlett test from the stats package, revealed significant non-equality of variances for both ethanol–water and methanol–water mixtures (*p* < 0.001*p* < 0.001*p* < 0.001). These findings indicated the need for statistical methods that account for unequal variances when computing *p*-values. To evaluate the significance of differences between temperatures, Welch’s t-test^70^ was applied using the stats package in R. Welch’s t-test is specifically designed to handle datasets with unequal variances, making it suitable for this analysis. The *p*-values generated from Welch’s t-test provided robust comparisons between the temperature groups for both ethanol–water and methanol–water mixtures. These tests ensured that the comparisons were statistically valid and reliable, reflecting the true impact of temperature on Δt2, capacitance, and ToE. The **ggplot2** package was employed for generating detailed plots, and the caret package was used for calculating RMSE and evaluating model performance. For data manipulation and preparation, the **dplyr** package was utilized, allowing efficient filtering, grouping, and transformation of large datasets. Additional packages like **readxl** facilitated the import of experimental data directly from Excel files. The use of LOESS modeling, accessible through the **geom_smooth** function in ggplot2, allowed for precise depiction of non-linear behavior, particularly at higher temperatures and solvent concentrations. This analysis demonstrated the robust capabilities of R for handling complex datasets and applying rigorous statistical techniques. The results underscore the importance of using flexible models like LOESS for systems exhibiting non-linear interactions. The findings contribute valuable insights into the effects of temperature and concentration on chemical mixtures, supporting the reliability of the proposed methods for evaluating such dynamic systems. These techniques align with previously published methodologies in the field and expand on their application through improved modeling and data visualization.

## Supplementary Information

Below is the link to the electronic supplementary material.

Supplementary Material 1

Supplementary Material 2

Supplementary Material 3

## Data Availability

The results of our research will be available upon request and sent to the corresponding authors.
