# Supplementary material for: Complementary metal-oxide-semiconductor (CMOS) time of evaporation measurement system for binary chemical monitoring
Source: Sci Rep. 2026 Jan 23;16:5542. doi: 10.1038/s41598-026-35322-x (PMC12887047; doi:10.1038/s41598-026-35322-x)
Supplement: Supplementary file 1 — Supplementary Material 1 [file 41598_2026_35322_MOESM1_ESM.docx]

**Complementary Metal-Oxide-Semiconductor (CMOS) Time of Evaporation Measurement System for Binary Chemical Monitoring (supplementary)**

Ebrahim Ghafar-Zadeh^1*^, Saghi Forouhi^2*^, Hamed Osouli Tabrizi^1^, Abbas Panahi^1^, Yasaman Tahernezhad^1^, Azadeh Amrollahi Biyouki^1^

^1^Biologically Inspired Sensors and Actuators Laboratory (BioSA Lab), Department of Electrical Engineering and Computer Science, Lassonde School of Engineering, York University, Toronto, ON, M3J1P3, Canada

^2^Division of Electronics and Computer Engineering (ELDA), Department of Electrical Engineering (ISY), Linköping University, 581 83 Linköping, Sweden

*Corresponding author: Author (e-mail: [egz@yorku.ca](mailto:egz@yorku.ca), [saghi.forouhi@liu.se](mailto:saghi.forouhi@liu.se))

# Statistical Analysis

## Water_Ethanol Analysis

### Dynamic range

Based on the collected data shown in Figure 3, Table S1 presents the dynamic ranges for both capacitance (cap) and time across different temperatures (25°C, 40°C, 50°C, and 60°C), calculated as the percentage change between the maximum and minimum values relative to the mean value. The dynamic range for capacitance shows a slight decrease, from 4.98% at 25°C to 3.27% at 60°C, indicating that fluctuations in the sensor’s capacitance diminish slightly as the temperature increases. Conversely, the dynamic range for time remains significantly higher than that for capacitance, with values ranging from 325.70% at 25°C to 379.17% at 60°C. This high variability in time reflects greater inconsistency in the sensor's transitions, especially at higher temperatures, with the largest variation observed at 60°C. In conclusion, while the dynamic range for capacitance decreases slightly with increasing temperature, the dynamic range for time of evaporation (ToE) reveals a substantial and increasing variability. This suggests that the sensor’s timing becomes more erratic as temperature rises, particularly at 60°C, where the greatest time variability is observed. The formulas for dynamic range demonstrate how temperature affects both parameters (cap and ToE), showing that the sensor’s performance becomes increasingly inconsistent in terms of timing as temperature increases.

**Table S1.** Dynamic ranges for both cap and time at different temperatures

| *Temperature* | *25C* | *40C* | *50C* | *60C* |
| --- | --- | --- | --- | --- |
| *Dynamic range for cap* | 4.98% | 3.28% | 3.72% | 3.27% |
| *Dynamic range for time* | 325.70% | 342.48% | 316.27% | 379.17% |

### ToE-Condition plot

Figure S1 shows the behavior o${\Delta t}_{1}$(the time from the start to the flat cap) across different water-ethanol mixtures at various temperatures (25°C, 40°C, 50°C, and 60°C). At 25°C, the system exhibits moderate variability, with some fluctuations and notable error bars, especially at higher ethanol concentrations. At 40°C and 50°C, the system becomes more erratic, with large peaks and substantial variability, particularly around 50% ethanol. In contrast, at 60°C, the system is more stable, with smaller error bars and more consistent${\Delta t}_{1}$values across ethanol concentrations, indicating improved predictability at higher temperatures.


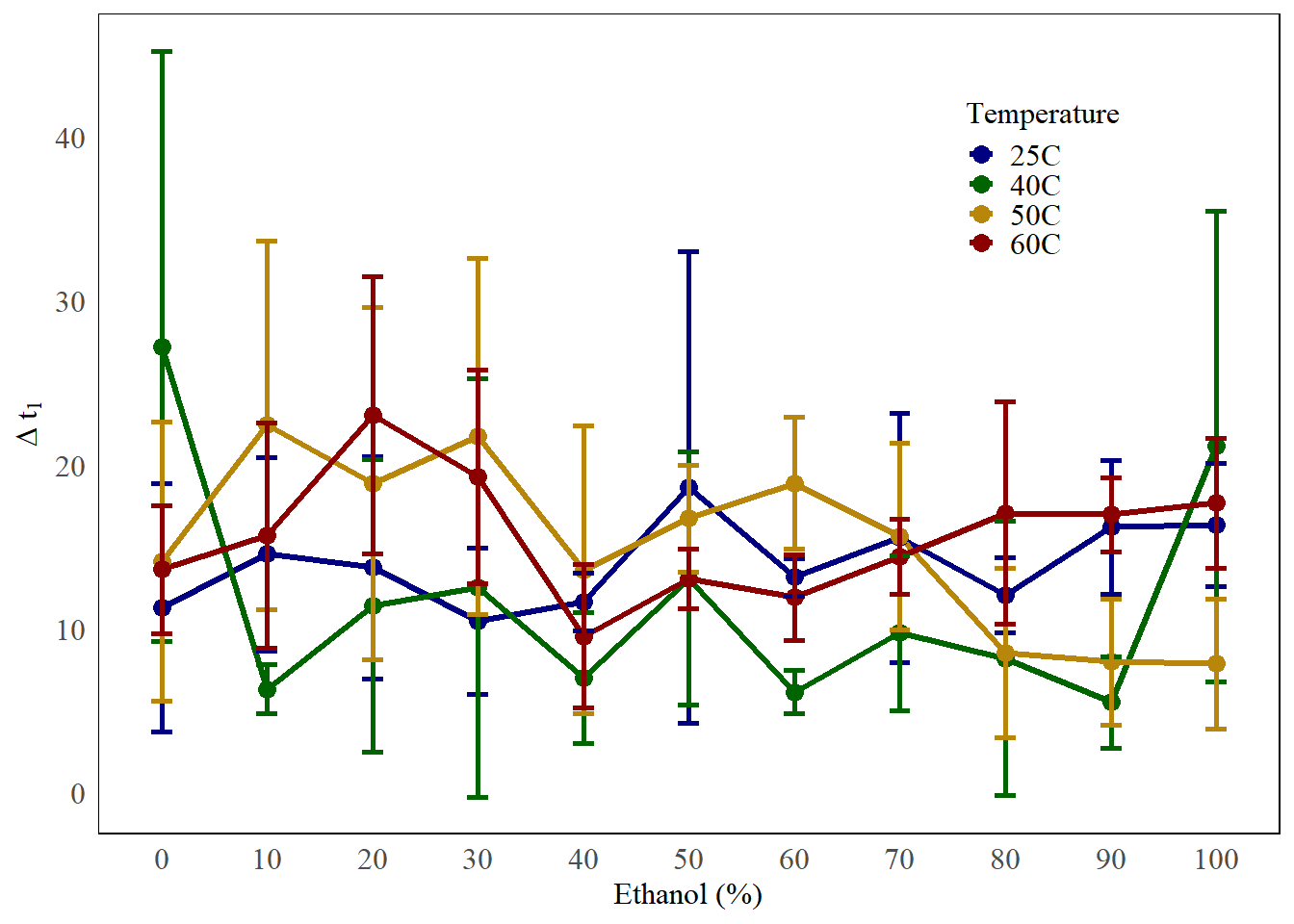


**Figure S1.** Initial evaporation phase duration (Δt₁) as a function of ethanol concentration in ethanol–water mixtures at four temperatures: 25 °C (blue), 40 °C (green), 50 °C (orange), and 60 °C (red). Δt₁ corresponds to the short, early stage immediately after droplet placement, capturing the sensor's response to initial spreading and early evaporation onset. Error bars represent standard deviation across replicate measurements.

The following plot (Figure S2) examines the behavior of ${\Delta t}_{2}$, the flat time period, across a range of water-ethanol. This plot shows the behavior of ${\Delta t}_{2}$​ (the time during the flat cap phase) across different ethanol concentrations and temperatures (25°C, 40°C, 50°C, and 60°C). As temperature increases, ${\Delta t}_{2}$ decreases consistently across all ethanol concentrations. At 25°C, ${\Delta t}_{2}$​ starts high, around 400 units, and steadily decreases as ethanol concentration increases, with significant error bars indicating variability. At 40°C, the trend is similar but ${\Delta t}_{2}$values are lower, around 200 units, and show less variability. The 50°C and 60°C curves follow the same pattern but at much lower values, suggesting more stability, especially at 60°C where the error bars are smaller and ${\Delta t}_{2}$​ remains under 100 units. Overall, the plot demonstrates that as temperature increases, the flat cap time $({\Delta t}_{2})$ decreases, and the system becomes more stable, particularly at higher temperatures.


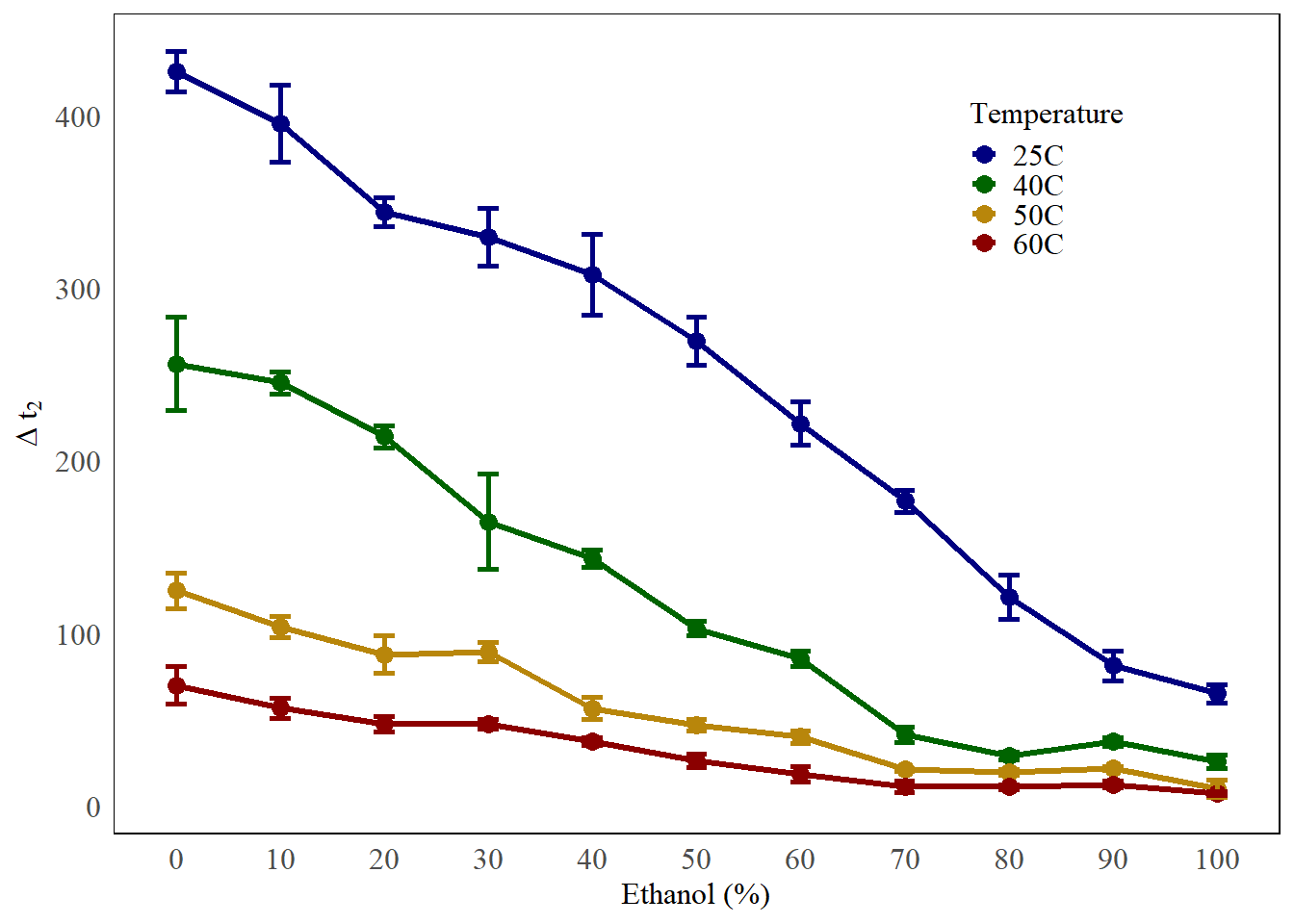


**Figure S2.** Intermediate evaporation phase duration (Δt₂) as a function of ethanol concentration in ethanol–water mixtures at four temperatures: 25 °C (blue), 40 °C (green), 50 °C (orange), and 60 °C (red). Standard deviation from multiple replicates is represented by the error bars.

Figure S3 shows the behavior of ${\Delta t}_{3}$ (the time from the flat cap to the drop) across various ethanol concentrations at different temperatures (25°C, 40°C, 50°C, and 60°C). At 25°C, there is significant fluctuation with high variability, indicated by large error bars. At 40°C, the system becomes more stable, but some variability is still present, particularly at low and high ethanol concentrations. At 50°C, ${\Delta t}_{3}$decreases with increasing ethanol concentration, though a spike at 30% ethanol introduces variability. At 60°C, the system is generally stable with low ${\Delta t}_{3}$values, except for a peak at 30% ethanol, where variability briefly increases. Overall, higher temperatures result in more consistent and stable ${\Delta t}_{3}$behavior, with fewer fluctuations compared to lower temperatures.


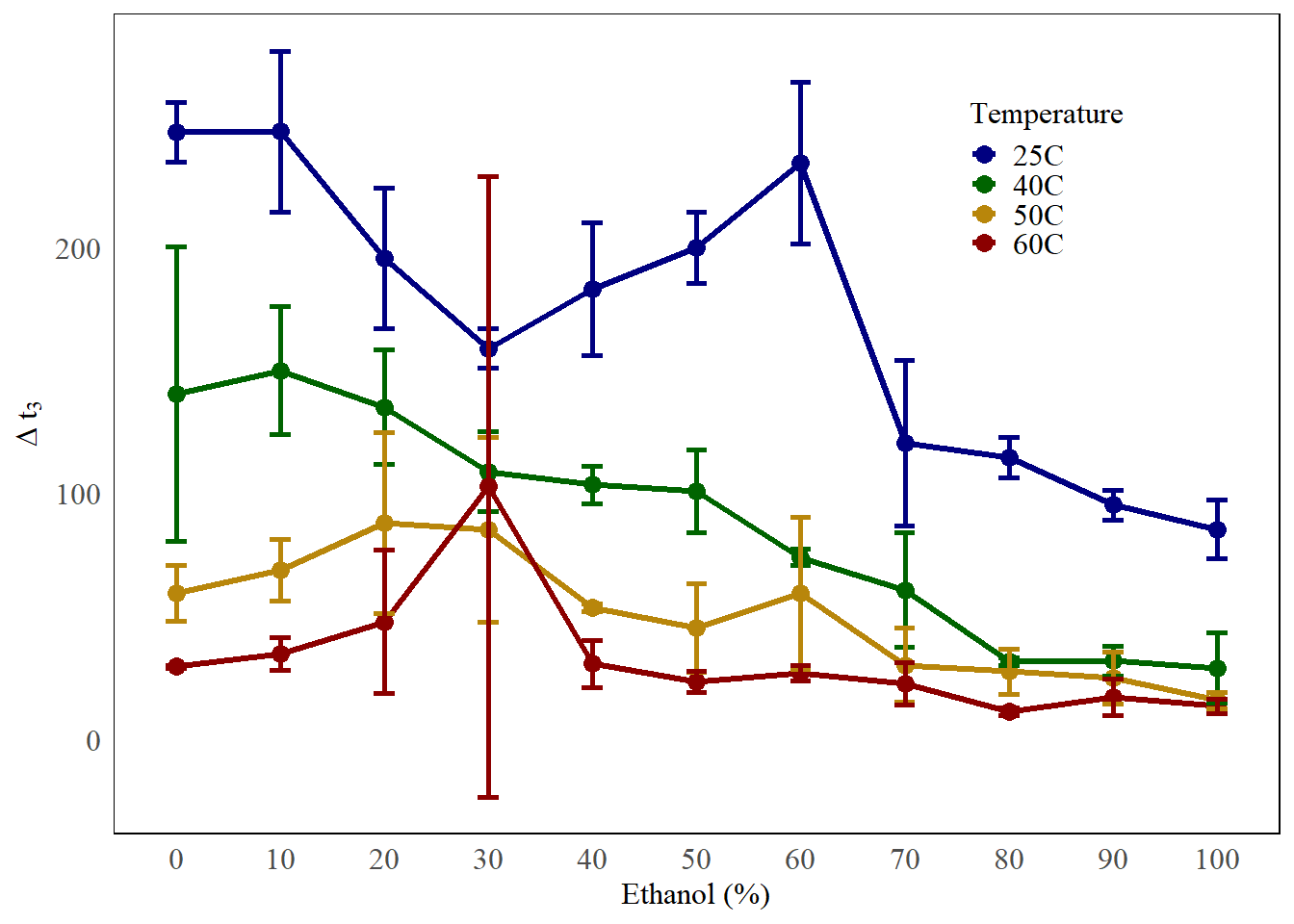


**Figure S3.** Time duration of the final evaporation phase (Δt₃) as a function of ethanol concentration in ethanol–water mixtures at four temperatures: 25 °C (blue), 40 °C (green), 50 °C (orange), and 60 °C (red). Data points represent mean Δt₃ values with standard deviation error bars from replicate measurements.

Figure S4 demonstrates ToE across ethanol concentrations at different temperatures (25°C, 40°C, 50°C, and 60°C). At 25°C, ToE is the highest, starting above 600 units and decreasing steadily as ethanol concentration increases. At 40°C, ToE is lower, starting around 400 units, but follows a similar downward trend. At 50°C and 60°C, ToE is significantly shorter, remaining around 200 units, with a noticeable spike in variability at 30% ethanol. Overall, higher temperatures lead to shorter and more stable ToEs, with variability decreasing as temperature increases.


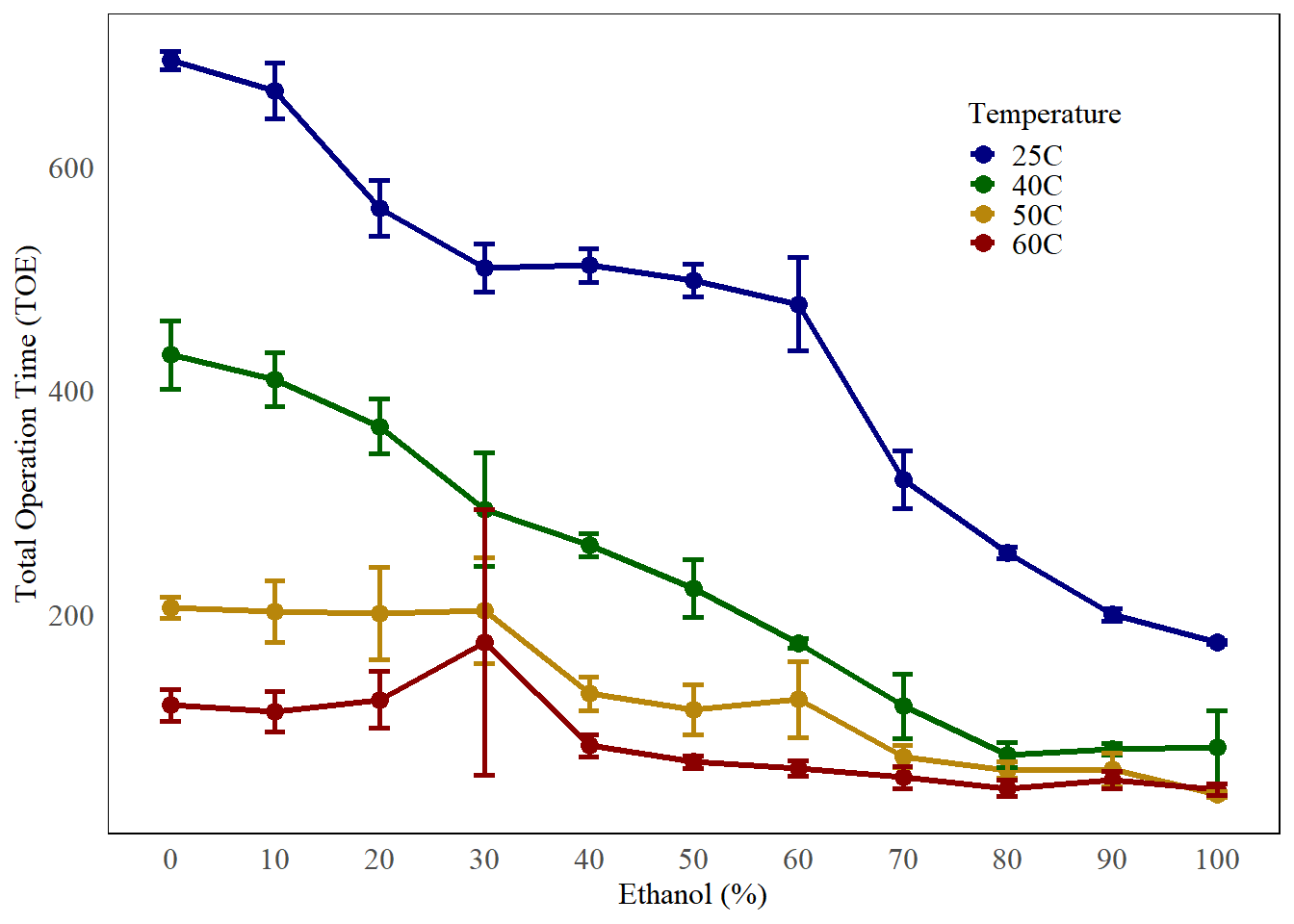


**Figure S4.** ToE as a function of ethanol concentration for ethanol–water mixtures at four temperatures: 25 °C (blue), 40 °C (green), 50 °C (orange), and 60 °C (red). Each data point represents the mean ToE across three replicates, with error bars indicating standard deviation.

### Capacitance-condition plot

The bar plot shown in Figure S5 presents the mean $\Delta$cap values at each ethanol concentration for all four temperatures, accompanied by error bars representing variability. The results indicate that $\Delta$cap remains relatively stable across temperatures, with values ranging between 100 and 160 units. However, the presence of large error bars suggests considerable variability in the capacitance response, particularly at higher ethanol concentrations. Despite this variability, there is no clear pattern of significant changes in $\Delta$cap across temperatures, implying that temperature alone does not drastically alter capacitance, and other factors may contribute to these fluctuations.


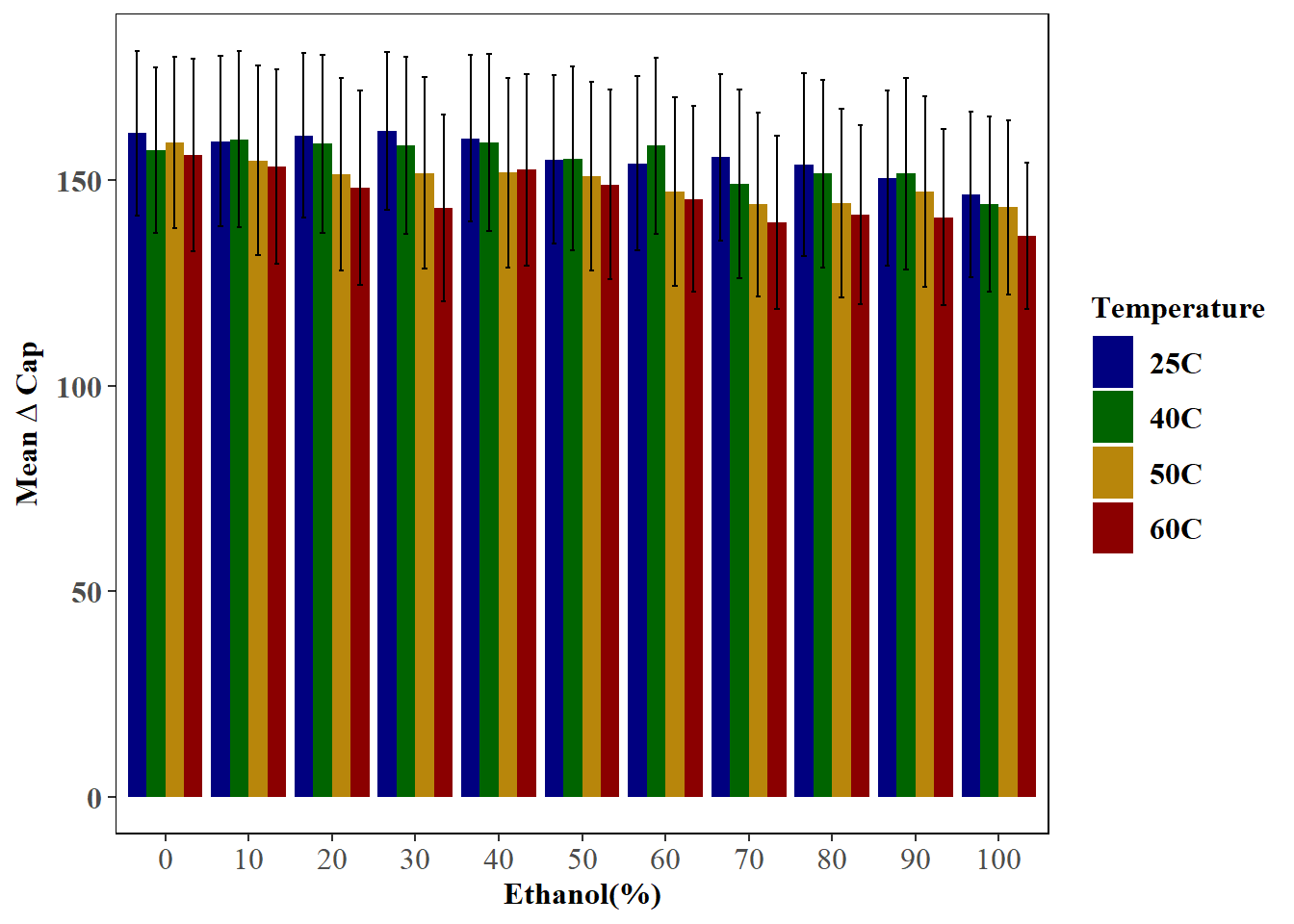


**Figure S5.** Mean change in capacitance (Δcap) as a function of ethanol concentration in ethanol–water mixtures at four temperatures: 25 °C (blue), 40 °C (green), 50 °C (orange), and 60 °C (red). Δcap reflects the average capacitive signal variation during evaporation and serves as a proxy for dielectric property changes in the droplet.

## Water_Methanol Analysis

### Dynamic range

Table S2 offers critical insights into how temperature influences the dynamic range of both capacitance and time within the system. Upon examining the dynamic range for capacitance, the values remain relatively consistent across all temperatures, with minor fluctuations between 4.43% and 5.36%. At 25°C, the dynamic range is 4.64%, which increases slightly to 5.36% at 40°C. This suggests that the system's response in terms of capacitance variation remains relatively stable across these temperatures. At 50°C, the dynamic range for capacitance shows a slight reduction to 4.83%, and at 60°C, it further decreases to 4.43%. These small changes indicate that while the system remains stable across different methanol concentrations, the sensitivity to these changes slightly decreases as the temperature rises. However, this reduction is marginal and does not suggest any dramatic shifts in system behavior with respect to capacitance. In contrast, the dynamic range for time presents a much more pronounced variability as temperature changes. At 25°C, the system exhibits a large dynamic range for time, reaching 306.01%, indicating that the system maintains a stable flat capacitance for an extended period before transitioning into a decline.

**Table S2.** Dynamic ranges for both cap and time at different temperatures

| *Temperature* | *25C* | *40C* | *50C* | *60C* |
| --- | --- | --- | --- | --- |
| *Dynamic range for cap* | 4.64% | 5.36% | 4.83% | 4.43% |
| *Dynamic range for time* | 306.01% | 335.206% | 309.76% | 294.717% |

As the temperature rises to 40°C, the ToE dynamic range increases slightly to 335.206%, demonstrating that the system can sustain its flat capacitance even longer at this temperature. However, as the temperature increases further to 50°C and 60°C, the dynamic range for time begins to narrow, dropping to 309.76% at 50°C and further decreasing to 294.717% at 60°C. These reductions indicate that, at higher temperatures, the system transitions more rapidly out of the stable phase, resulting in shorter periods of flat capacitance. Overall, the data shows that while the dynamic range for capacitance remains fairly consistent across different temperatures, the system’s ability to maintain stable capacitance over time is significantly impacted by temperature. At higher temperatures, particularly at 60°C, the system reacts more quickly, with shorter periods of stability in the flat capacitance phase. This behavior aligns with previous observations of quicker transitions and reduced flat time zones at elevated temperatures, reflecting the system's tendency to respond more uniformly and with less variability as the temperature increases.

### ToE-Condition plot

The plot of ${\Delta t}_{1}$ (the time interval from the start to the sharpness time) for the methanol-water mixture across varying methanol concentrations and temperatures (25°C, 40°C, 50°C, and 60°C) shown in Figure S6 demonstrates substantial variability, particularly at higher methanol concentrations and temperatures. Unlike the ToE, the ${\Delta t}_{1}$values fluctuate significantly, with prominent peaks and wide error bars, especially at 50°C and 60°C, suggesting greater instability and inconsistency in system response during the initial phase of operation at these temperatures. At lower temperatures (25°C and 40°C), the ${\Delta t}_{1}$values are generally more stable, showing lower variability and tighter error bars, indicating more consistent performance at these temperatures. The variations seen at higher temperatures and concentrations suggest that the system’s response is more erratic under these conditions, which could reflect complex interactions between the methanol concentration and the sensor's ability to detect sharp transitions in capacitance. Overall, the data highlight a clear temperature-dependent behavior, where increasing temperatures lead to greater variability in the system's early operational phase.


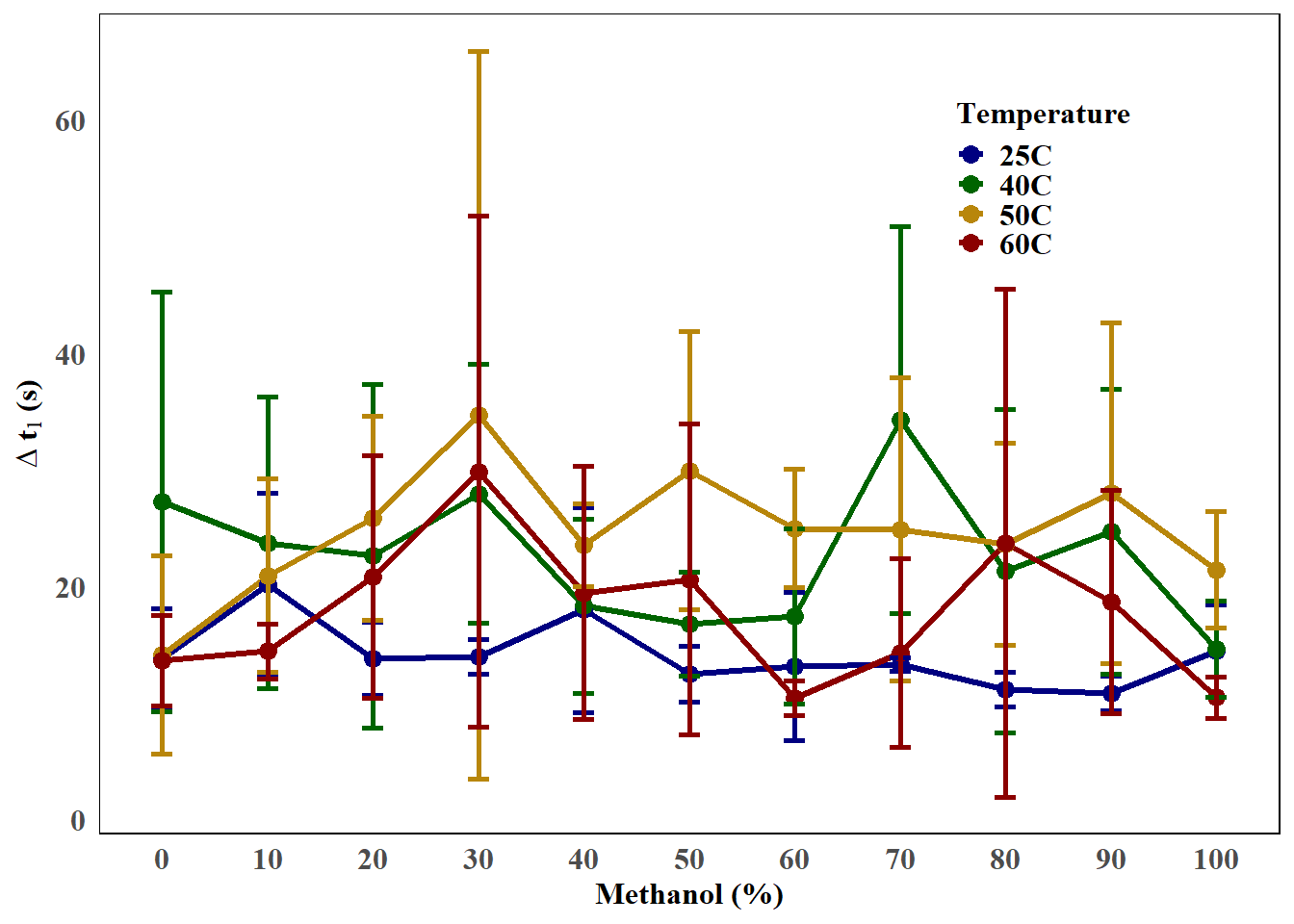


**Figure S6.** Initial evaporation phase duration (Δt₁) as a function of methanol concentration in methanol–water mixtures at four temperatures: 25 °C (blue), 40 °C (green), 50 °C (orange), and 60 °C (red). Error bars represent standard deviation across replicate measurements.

Figure S7 is the plot for ${\Delta t}_{2}$(the time from sharpness time to the end of the flat cap period) as a function of methanol concentration across different temperatures (25°C, 40°C, 50°C, and 60°C) provides insight into how the system's operational duration changes based on the methanol percentage and temperature. At lower temperatures, especially 25°C, ${\Delta t}_{2}$remains relatively high, with a notable decrease as the methanol concentration increases. This suggests that at 25°C, the system's operational phase after sharpness is prolonged, particularly at lower methanol concentrations, but it declines sharply as methanol increases beyond 70%. In contrast, at higher temperatures (40°C, 50°C, and 60°C), the ${\Delta t}_{2}$values are consistently lower across the methanol concentrations. This indicates a faster completion of the system’s operational cycle after the sharpness time as temperature increases. The trend is more pronounced at 60°C, where ${\Delta t}_{2}$is significantly shorter and shows less variability with methanol concentration. The overall decrease in ${\Delta t}_{2}$with rising temperature reflects faster system response at elevated temperatures, possibly due to enhanced fluid dynamics or sensor interactions at those conditions. The error bars, particularly at 25°C, are wider, indicating greater variability in the system's behavior under lower temperatures, while higher temperatures exhibit more consistency, especially at methanol concentrations above 50%. This graph highlights the influence of temperature on the operational duration after the sharpness event, with lower temperatures resulting in prolonged operation, especially at lower methanol concentrations, while higher temperatures streamline this phase.


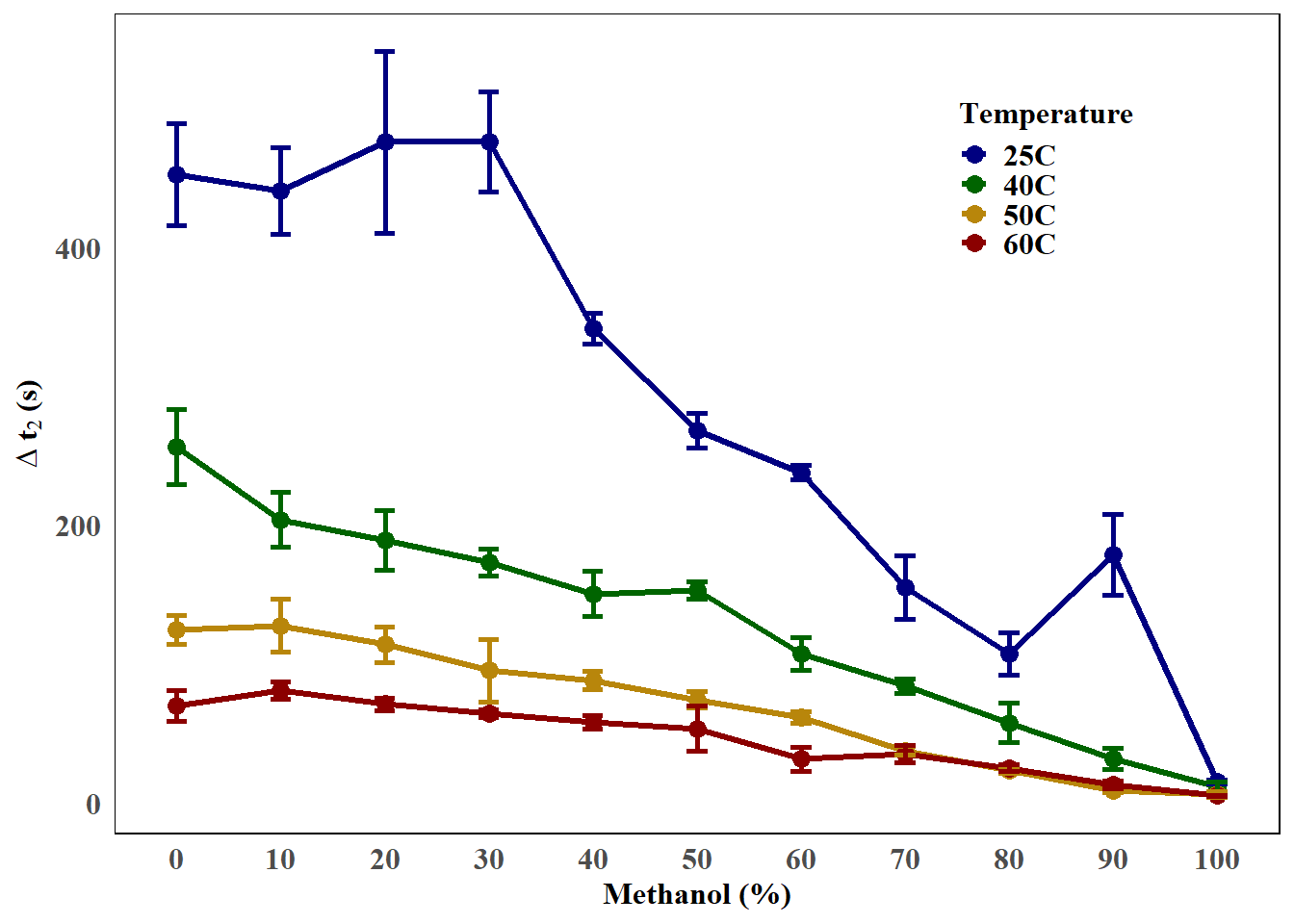


**Figure S7.** Intermediate evaporation phase duration (Δt₂) as a function of methanol concentration in methanol–water mixtures at four temperatures: 25 °C (blue), 40 °C (green), 50 °C (orange), and 60 °C (red). Standard deviation from multiple replicates is represented by the error bars.

Figure S8 shows${\Delta t}_{3}$ (the time interval from the end of the flat cap to the end of the operation) as a function of methanol concentration across different temperatures (25°C, 40°C, 50°C, and 60°C). The behavior of ${\Delta t}_{3}$seems to be more variable compared to ${\Delta t}_{2}.$There is no consistent trend across temperatures, and the fluctuations appear more pronounced, particularly at 20% methanol concentration, where all temperature levels exhibit larger variations and error bars. At 25°C, ${\Delta t}_{3}$shows relatively stable values across most methanol concentrations, although with higher variability at certain points (e.g., 30% and 50% methanol). At 40°C, the values of ${\Delta t}_{3}$ also remain moderately consistent, although there is some fluctuation. At higher temperatures, specifically 50°C and 60°C, the values generally decrease after 30% methanol, indicating a shorter duration for the final phase of operation at elevated temperatures. The large error bars at certain concentrations (e.g., 20% methanol at 60°C) suggest that the system's behavior becomes more unpredictable under specific methanol concentrations, particularly at higher temperatures. This could indicate that the final operational phase is more sensitive to variations in temperature and methanol concentration, making it challenging to establish a clear trend in ${\Delta t}_{2}$ under these conditions.


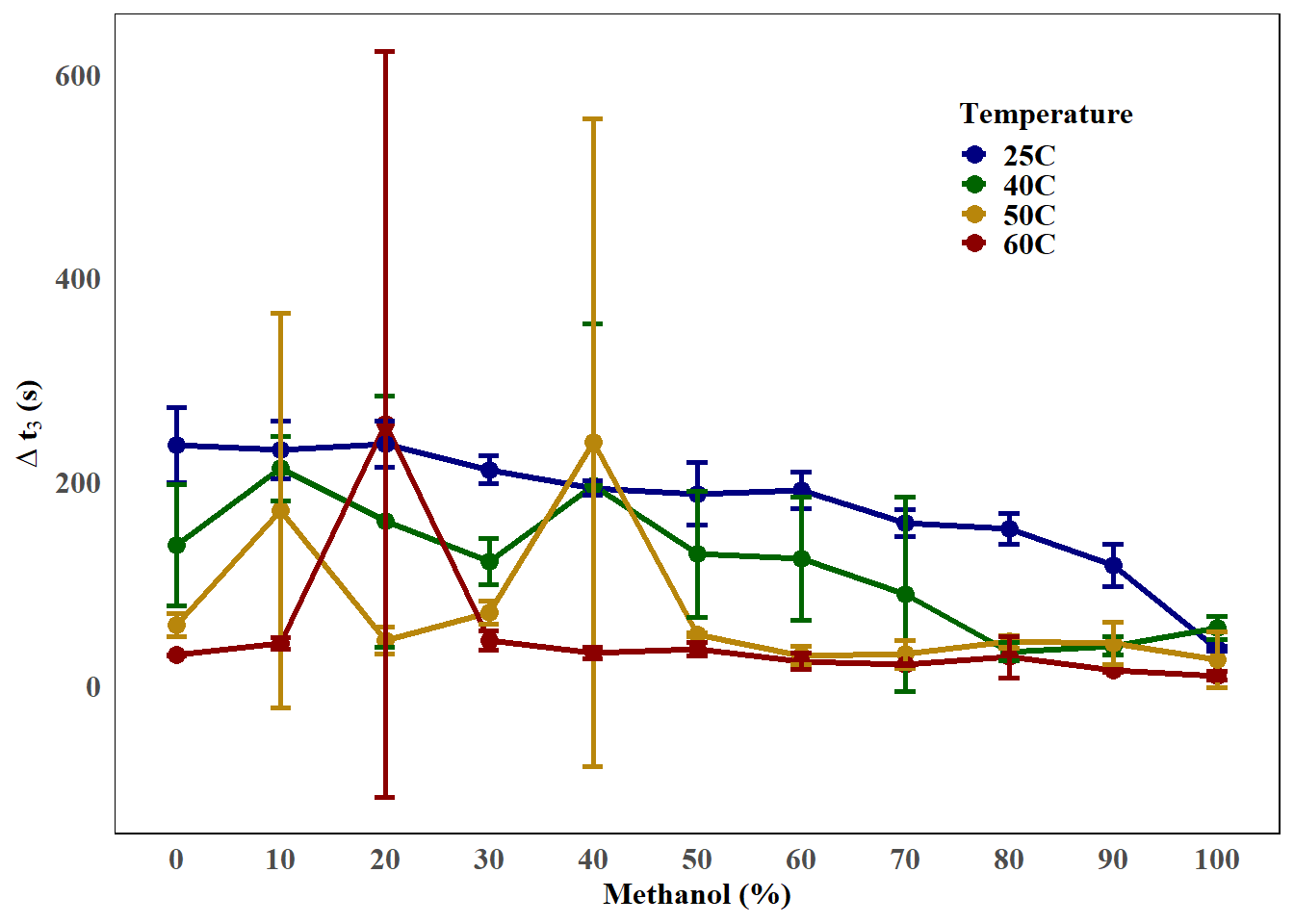


**Figure S8.** Final evaporation phase duration (Δt₃) as a function of methanol concentration in methanol–water mixtures at four temperatures: 25 °C (blue), 40 °C (green), 50 °C (orange), and 60 °C (red). Δt₃ reflects the final decay phase in the evaporation profile, typically associated with the last stages of liquid film thinning and complete solvent removal.

The analysis of the ToE for methanol-water mixtures across different methanol concentrations and temperatures (25°C, 40°C, 50°C, and 60°C) reveals distinct patterns of system behavior (Figure S9). The plot indicates that ToE remains relatively high at lower methanol concentrations, particularly at 25°C, where ToE values start near 800 seconds. As methanol concentration increases, ToE generally declines, with the sharpest decreases observed for higher temperatures (50°C and 60°C), which maintain lower ToE values throughout. At intermediate methanol concentrations, the plot shows notable variability, especially for 50°C, where significant spikes in ToE occur, suggesting irregular system behavior or transitions in sensor response. These results demonstrate that higher temperatures tend to reduce ToE overall, reflecting more efficient operation, while the impact of methanol concentration is more complex, particularly at intermediate levels where system responses appear less stable, as indicated by larger error bars. This suggests the presence of temperature-dependent dynamics in the methanol-water mixture's performance.


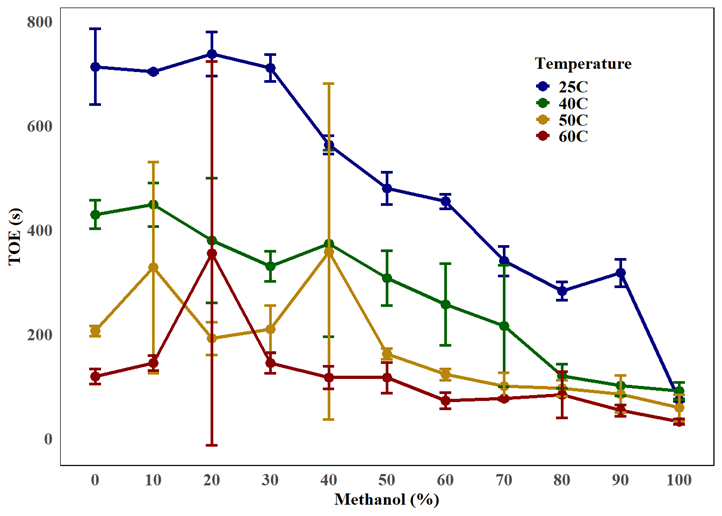


**Figure S9.** ToE as a function of methanol concentration in methanol–water mixtures at four temperatures: 25 °C (blue), 40 °C (green), 50 °C (orange), and 60 °C (red). ToE represents the full duration from droplet deposition to complete evaporation as captured by the capacitive sensor.

### Capacitance-Condition plot

In the bar plot depicted in Figure S10, the mean $\Delta$cap values are presented for each temperature condition, with error bars reflecting variability across replicates. The values remain relatively stable, ranging from 100 to 160 units, despite increasing methanol concentration. Notably, larger error bars at higher methanol concentrations suggest significant variability in capacitance response, though no clear trend emerges to suggest that temperature drastically alters $\Delta$cap. This stability across temperatures may indicate that temperature is not the primary factor driving fluctuations in capacitance behavior, and further investigation into other system variables may be warranted to explain the observed variance.


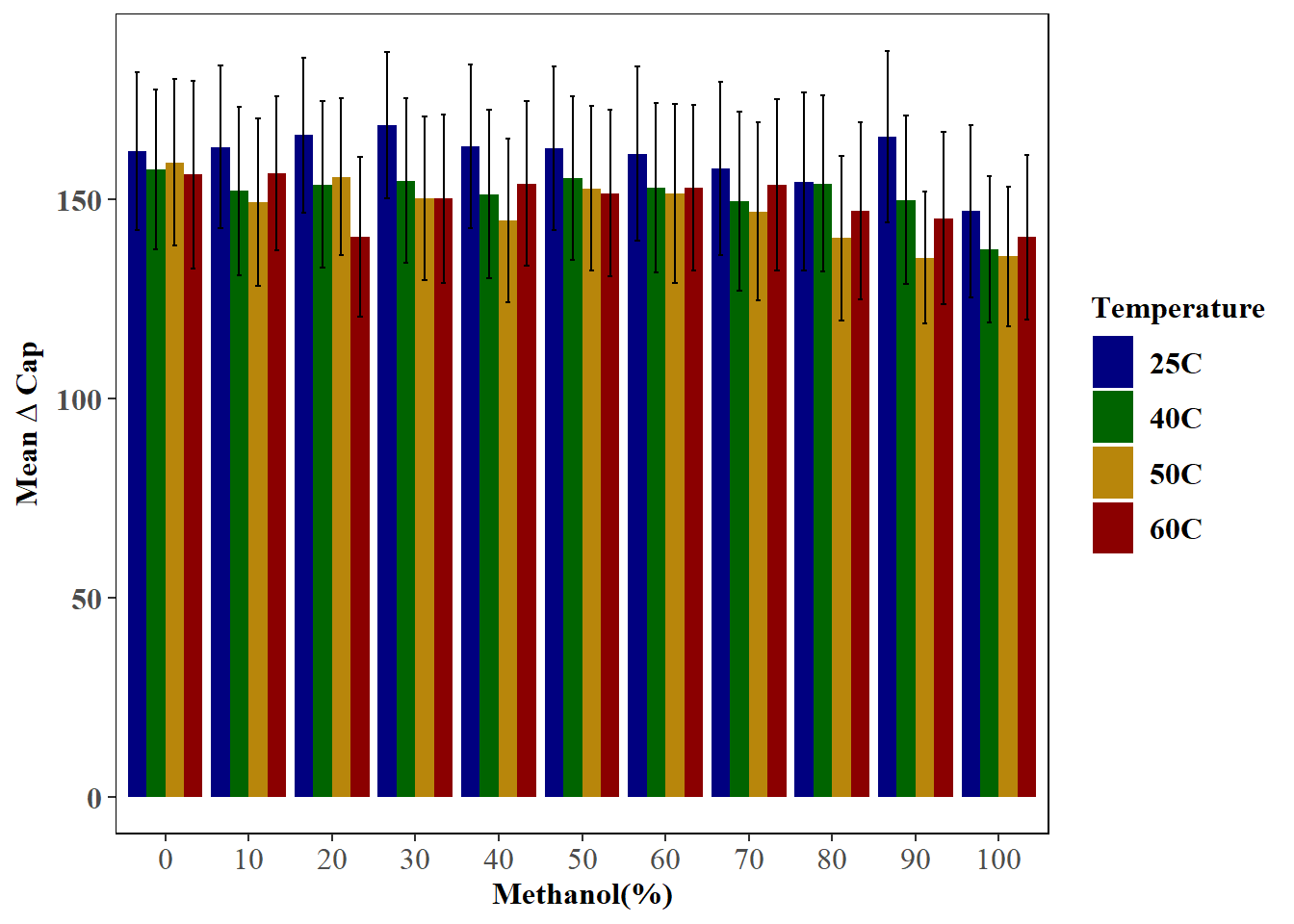


**Figure S10.** Mean change in capacitance (Δcap) as a function of methanol concentration in methanol–water mixtures at four temperatures: 25 °C (blue), 40 °C (green), 50 °C (orange), and 60 °C (red). Δcap reflects the average capacitive signal variation during evaporation and serves as a proxy for dielectric property changes in the droplet.

## Methanol_Ethanol Analysis

### Normality and equality variance test

The results in Table S3 show normality assumptions for the temperature 25C.

**Table S3.** Shapiro_normality test

| Temperature | t-statistics | p-value |
| --- | --- | --- |
| 25C | 0.89797 | 0.1746 |

This boxplot in Figure S11 presents the distribution of ${\Delta t}_{2}$ (time from the flat region to the end) for ethanol-methanol mixtures at 25°C, with individual data points corresponding to varying methanol concentrations (0% to 100%). The interquartile range (IQR) represents the central 50% of the data, while the whiskers display the spread of the data outside this range. The plot indicates significant variability in ${\Delta t}_{2}$ values across different concentrations, with several outliers present, particularly at 0% and 100% methanol. These outliers suggest distinct behavior in the system at these concentrations, potentially reflecting differences in the interaction between ethanol and methanol at varying proportions. The relatively wide spread of the data points and the length of the whiskers highlight the complexity of the relationship between methanol concentration and${\Delta t}_{2}$. Notably, there is no clear linear trend between methanol content and ${\Delta t}_{2}$, implying that the system’s response time after the flat region may be influenced by non-linear interactions within the mixture. Further investigation into these outliers and the factors driving the variability in ${\Delta t}_{2}$ across concentrations would provide deeper insights into the behavior of the ethanol-methanol system at this temperature.

**
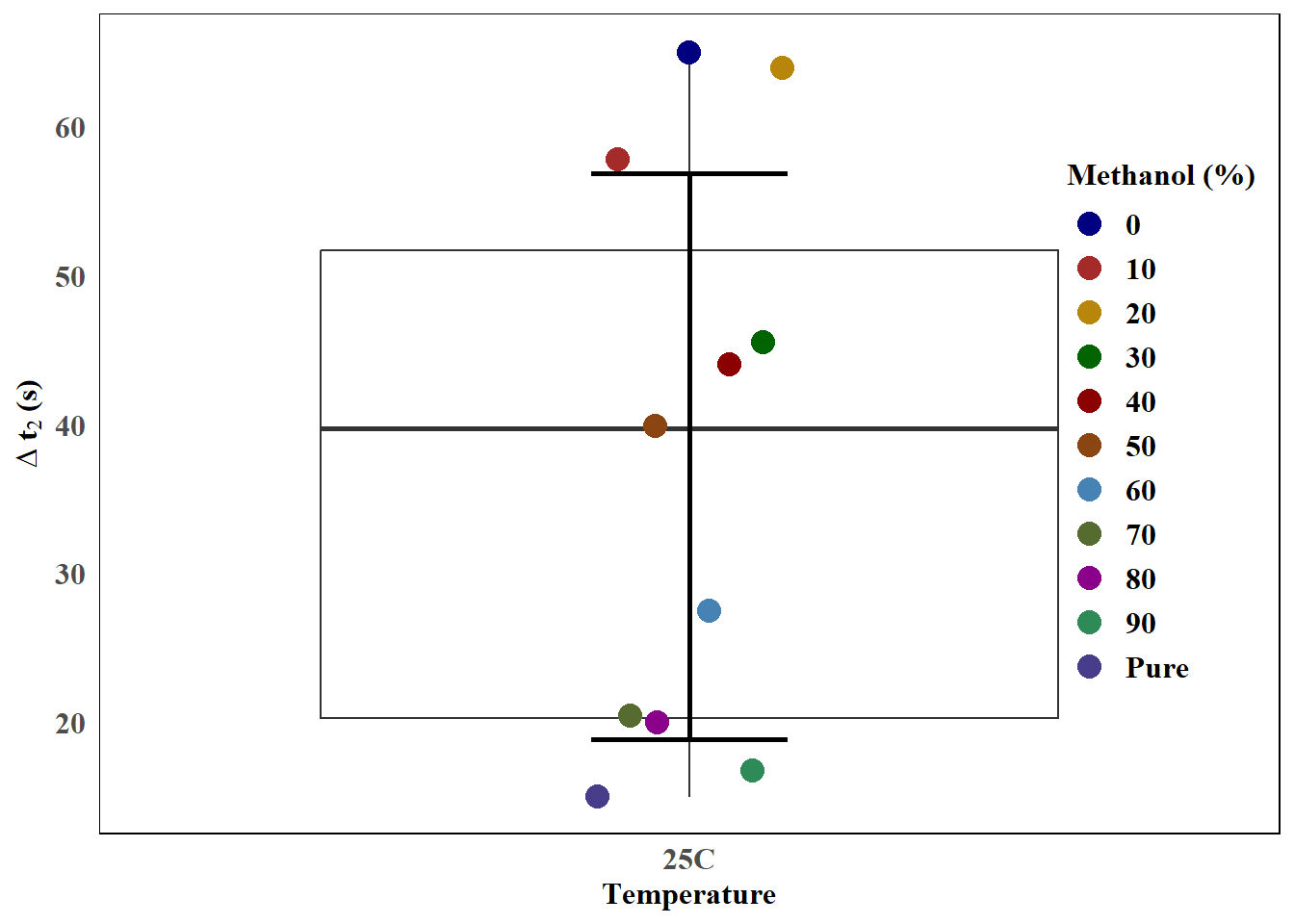
**

**Figure S11.** Scatter distribution of intermediate evaporation phase durations (Δt₂) at 25 °C for ethanol–methanol mixtures with varying methanol concentrations. Each colored dot represents a specific methanol percentage, as indicated in the legend. The boxplot overlay shows the median (central horizontal line), interquartile range (box), and minimum/maximum thresholds (whiskers), highlighting the spread and central tendency of Δt₂ values.

### Cap-Conditions plot

Figure S12 shows a bar plot of the mean $\Delta$cap across methanol concentrations ranging from 0% to 100%, with error bars indicating the variability in sensor readings. The results reveal that the mean capacitance remains relatively stable across all methanol concentrations, with slight variations observed. This stability suggests that the sensor’s capacitance response is not significantly affected by the concentration of methanol in the mixture at 25°C. The error bars show a consistent range of variability, indicating that while the sensor’s readings fluctuate slightly, they remain consistent across different concentrations.


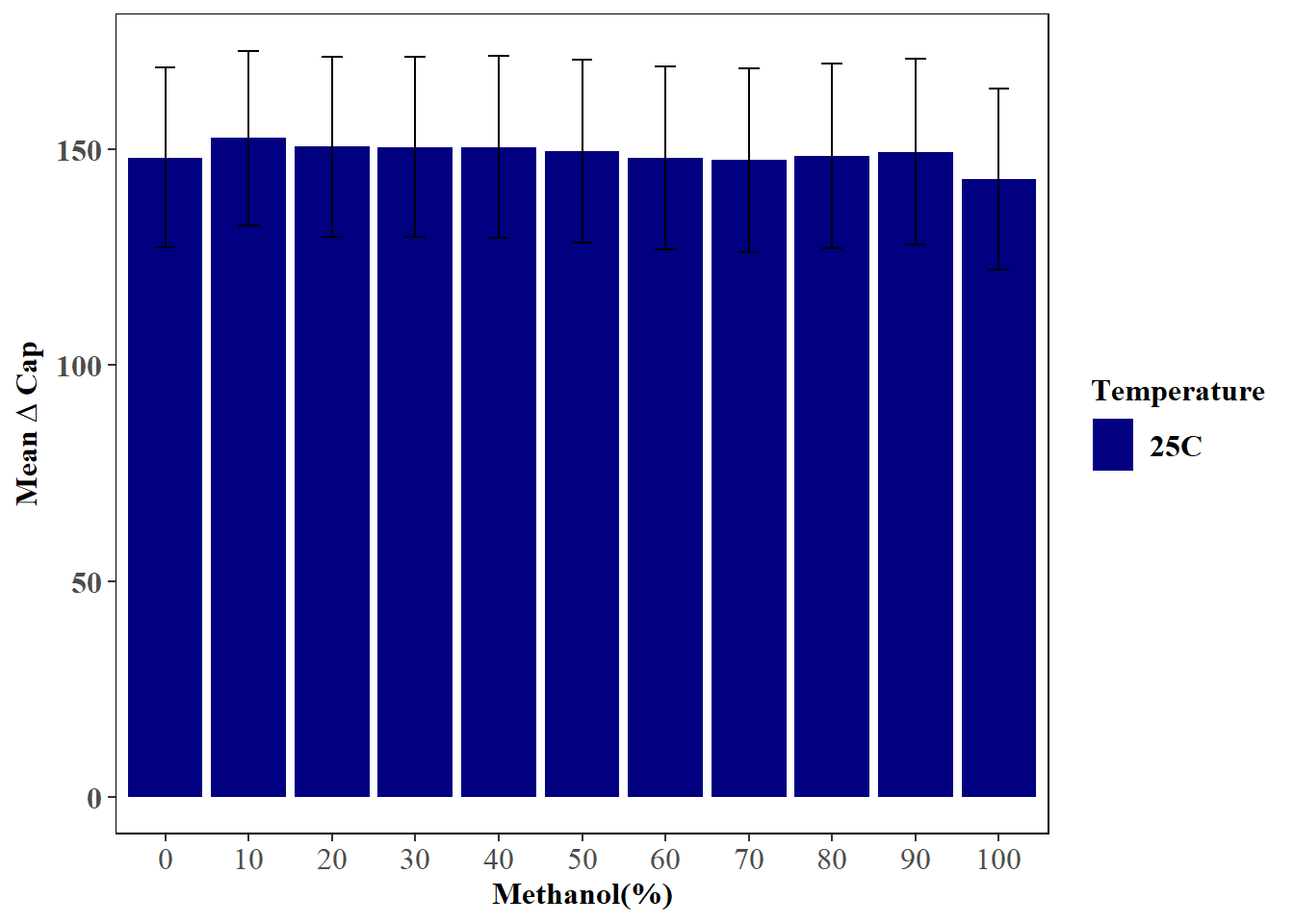


**Figure S12.** Mean change in capacitance (Δcap) at 25 °C as a function of methanol concentration in ethanol–methanol mixtures. Each bar represents the average Δcap measured during the evaporation process for a given concentration, with error bars indicating standard deviation across replicate measurements.

## Comparing three mixture

Figure S13 (a and b) present complementary visual analyses of the time parameter ${\Delta t}_{2}$ across various temperatures and mixture types. (A) Figure S13a is a box plot that provides a comparative analysis of ${\Delta t}_{2}$for three distinct mixture types (ethanol-water, methanol-water, and methanol-ethanol) across four temperatures (25°C, 40°C, 50°C, and 60°C). Each box plot summarizes the distribution of ${\Delta t}_{2}$values for each mixture type at the respective temperatures, while the overlaid points represent individual data points corresponding to varying alcohol concentrations (ranging from 0% to 100%). The gradient of the points, from navy blue (0% concentration) to gold (100% concentration), emphasizes the influence of alcohol concentration on ${\Delta t}_{2}$values. The plot reveals notable differences across both temperatures and mixtures. At 25°C, the ethanol-water and methanol-water mixtures exhibit much higher ${\Delta t}_{2}$values than at higher temperatures, especially at higher concentrations. In contrast, the methanol-ethanol mixture consistently demonstrates lower ${\Delta t}_{2}$values across all temperatures, indicating a distinct kinetic behavior compared to the other two mixtures. (B) Figure S13b is a scatter plot that focuses on the relationship between the percentage of alcohol concentration and ${\Delta t}_{2}$values for each mixture type, specifically at 25°C. The solid lines represent linear regression fits for the ethanol-water (E-W), methanol-water (M-W), and methanol-ethanol (M-E) mixtures.

| 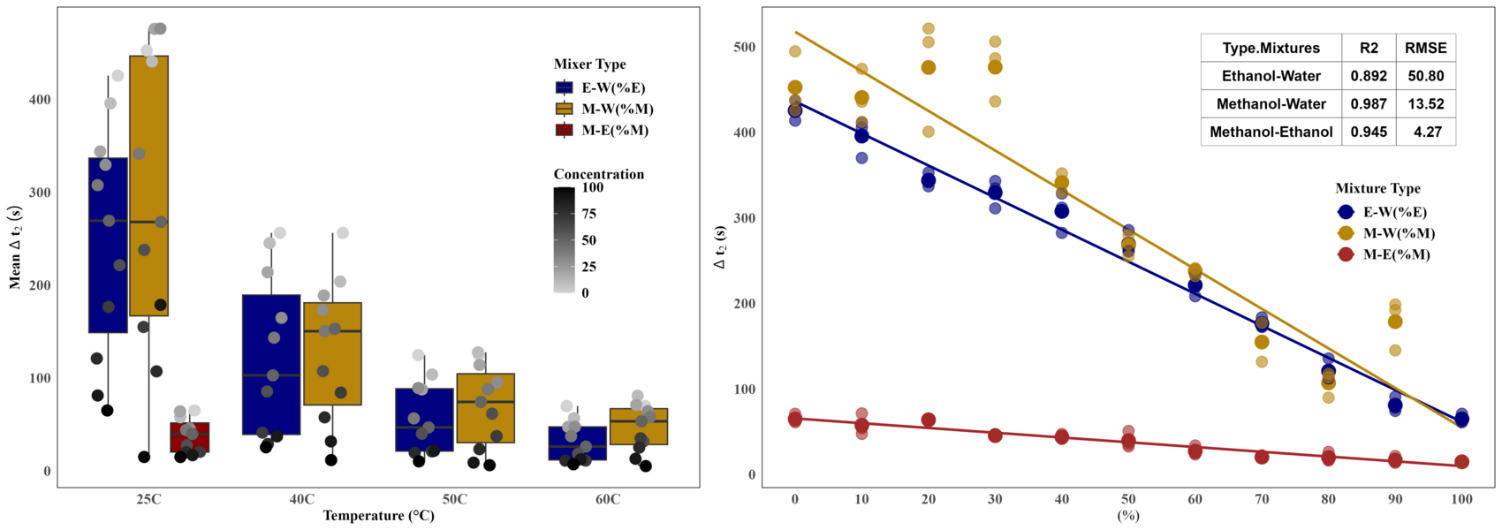 | |
| --- | --- |
| (a) | (b) |

**Figure S13.** (a) Boxplot of mean intermediate evaporation phase duration (Δt₂) across four temperatures (25 °C, 40 °C, 50 °C, 60 °C) for three binary mixture types: Ethanol–Water (E–W, blue), Methanol–Water (M–W, orange), and Methanol–Ethanol (M–E, red), (b) Linear regression analysis of Δt₂ versus solvent concentration for each mixture type. Ethanol–water and methanol–water mixtures show strong negative correlations with increasing concentration, as reflected in high R² values (0.892 and 0.987, respectively), while methanol–ethanol exhibits minimal variation. RMSE values indicate the best model fit for methanol–ethanol, followed by methanol–water.

The smaller data points in the plot indicate individual ${\Delta t}_{2}$values for three selected runs within each mixture type, while the larger points denote the mean ${\Delta t}_{2}$values. The slope of the regression lines demonstrates a strong negative correlation between alcohol concentration and ${\Delta t}_{2}$ for ethanol-water and methanol-water mixtures, while the methanol-ethanol mixture shows a much flatter relationship, indicating minimal sensitivity to changes in concentration. Additionally, the table embedded within the image presents the $R^{2}$and RMSE values for each mixture, highlighting the high goodness-of-fit for the methanol-water ($R^{2}$= 0.987) and methanol-ethanol ($R^{2}$ = 0.945) mixtures, with relatively low RMSE values, suggesting strong predictive accuracy. In contrast, the ethanol-water mixture exhibits a lower $R^{2}$ (0.892) and higher RMSE (50.80), indicating more variability and less predictive accuracy in this mixture at 25°C. In summary, the statistical analyses in these images demonstrate that methanol-water mixtures exhibit the strongest relationship between alcohol concentration and ${\Delta t}_{2}$across all temperatures. The ethanol-water mixture shows higher variability, particularly at lower temperatures, while the methanol-ethanol mixture consistently demonstrates low ${\Delta t}_{2}$values with minimal sensitivity to concentration changes.

Table S4 provides a comprehensive analysis of the performance metrics for three different mixtures—Water-Ethanol (W-E), Water-Methanol (W-M), and Ethanol-Methanol (E-M)—across four temperature conditions (25°C, 40°C, 50°C, and 60°C). The metrics include $R^{2}$, RMSE values for both ${\Delta t}_{2}$ and $\Delta$cap, and dynamic percentages for these parameters. Each metric offers insights into the behavior of the mixtures as they interact with ethanol concentrations at varying temperatures. The $R^{2}$ values, available only for the 25°C condition, suggest that the Water-Methanol mixture has the strongest linear relationship between ethanol concentration and $\Delta_{t2}$, with an $R^{2}$value of 0.275. This implies that the model explains a greater portion of the variability in ${\Delta t}_{2}$for this mixture at 25°C. In contrast, the Ethanol-Methanol mixture has the weakest relationship, with an $R^{2}$ of 0.177, indicating less predictability in the system's response to ethanol concentration under this condition. The RMSE values for ${\Delta t}_{2}$reveal further distinctions among the mixtures. Water-Ethanol generally has higher RMSE values across all temperatures, suggesting that it exhibits more variability in ${\Delta t}_{2},$particularly at higher temperatures, such as 60°C. Conversely, Water-Methanol consistently shows lower RMSE values, indicating a more stable and predictable response. This is especially notable at 50°C, where the RMSE for ${\Delta t}_{2}$is as low as 4.847, further supporting the stability of this mixture under varying ethanol concentrations. When examining the RMSE for $\Delta$cap, a similar pattern emerges. At 25°C, the Water-Ethanol mixture displays the lowest RMSE for $\Delta$cap (12.471), reflecting a stable capacitance response at this temperature. However, as temperatures increase, especially at 60°C, the Water-Methanol mixture shows a significant rise in RMSE, reaching 29.396, indicating a greater degree of variability in the $\Delta$cap response. This highlights that while Water-Methanol may have a predictable ${\Delta t}_{2}$, its $\Delta$cap response becomes more erratic at higher temperatures. The dynamic range of ${\Delta t}_{2},$which reflects the percentage change between the lowest and highest values, is particularly pronounced for the Water-Ethanol mixture at 60°C, where it reaches 379.17%. This suggests a high sensitivity to ethanol concentration at this temperature, in contrast to the Water-Methanol mixture, which shows consistently lower dynamic ranges across all temperatures, indicating a more uniform system response. The dynamic range of $\Delta$cap, while smaller, follows a similar trend, with the highest variation observed for the Ethanol-Methanol mixture at 25°C (7.089%). In conclusion, the analysis reveals that the Water-Methanol mixture offers the most stability and predictability in terms of both ${\Delta t}_{2}$and $\Delta$cap, particularly at moderate temperatures like 50°C. In contrast, the Water-Ethanol mixture is more sensitive to changes in ethanol concentration, especially at higher temperatures, where it exhibits large dynamic ranges but also higher variability, as evidenced by the RMSE values. The Ethanol-Methanol mixture presents a mixed behavior, with moderate dynamic responses but higher variability in $\Delta$cap at elevated temperatures. These findings suggest that the choice of mixture and operating temperature can significantly influence the system’s behavior in response to ethanol concentration.

**Table S4.** Comprehensive Analysis Of The Performance Metrics

| Tempreture | Parameters | W-E | W-M | E-M |
| --- | --- | --- | --- | --- |
| 25C | $R^{2}$ | 0.208 | 0.275 | 0.177 |
|  | RMSE$\Delta_{t2}$ | 3.75 | 11.02 | 8.927 |
|  | RMSE$\Delta$cap | 12.471 | 21.26 | 18.606 |
|  | Dynamic$\Delta_{t2}$(%) | 325.76 | 306.01 | 300.69 |
|  | Dynamic$\Delta$cap %) | 4.98 | 4.64 | 7.089 |
| 40C |  | | | |
|  | RMSE$\Delta_{t2}$ | 7.94 | 4.188 |  |
|  | RMSE$\Delta$cap | 17.299 | 18.686 |  |
|  | Dynamic$\Delta_{t2}$(%) | 342.48 | 335.206 |  |
|  | Dynamic$\Delta$cap (%) | 3.28 | 5.36 |  |
| 50C |  | | | |
|  | RMSE$\Delta_{t2}$ | 7.84 | 4.847 |  |
|  | RMSE$\Delta$cap | 11.329 | 16.426 |  |
|  | Dynamic$\Delta_{t2}$(%) | 316.27 | 309.76 |  |
|  | Dynamic$\Delta$cap (%) | 3.72 | 4.83 |  |
| 60C |  | | | |
|  | RMSE$\Delta_{t2}$ | 8.248 | 7.848 |  |
|  | RMSE$\Delta$cap | 14.958 | 29.396 |  |
|  | Dynamic$\Delta_{t2}(\%)$ | 379.17 | 294.79 |  |
|  | Dynamic$\Delta$cap (%) | 3.27 | 4.43 |  |

## Boxplots showing temperature-dependent trends

The results in Table S5 and Table S6 confirm the assumptions underlying the statistical analysis for both ethanol-water and methanol-water mixtures across all temperatures (25°C, 40°C, 50°C, and 60°C). The Shapiro-Wilk normality tests showed no significant deviations from normality, as indicated by the p-values across all temperatures, confirming that the data for both mixtures are normally distributed. However, the equality of variance tests revealed significant non-equality of variances for both mixtures, with p-values below 0.05. This result indicates that the variance across temperature groups is not constant, which is a critical consideration for further statistical comparisons.

**Table S5.** Shapiro_normality test and equality variance test for ethanol-water mixture in different temperature

| \| Temperature \| t-statistics \| p-value \| \| --- \| --- \| --- \| \| 25C \| 0.9449 \| 0.5798 \| \| 40C \| 0.8918 \| 0.1468 \| \| 50C \| 0.91224 \| 0.2592 \| \| 60C \| 0.90547 \| 0.2153 \| | \| DF \| p_value \| \| --- \| --- \| \| 3 \| 9.208e-05 \| \| 40 \|  \| |
| --- | --- | --- | --- | --- | --- | --- | --- | --- | --- | --- | --- | --- | --- | --- | --- | --- | --- | --- | --- | --- | --- | --- |

**Table S6.** Shapiro_normality test and equality variance test for methanol-water mixture in different temperatures

| \| Temperature \| t-statistics \| p-value \| \| --- \| --- \| --- \| \| 25C \| 0.91985 \| 0.3174 \| \| 40C \| 0.97058 \| 0.8923 \| \| 50C \| 0.92321 \| 0.3463 \| \| 60C \| 0.94217 \| 0.5463 \| | \| DF \| p_value \| \| --- \| --- \| \| 3 \| 9.67e-06 \| \| 40 \|  \| |
| --- | --- | --- | --- | --- | --- | --- | --- | --- | --- | --- | --- | --- | --- | --- | --- | --- | --- | --- | --- | --- | --- | --- |

Given the non-equal variances across temperature groups, Welch’s t-test was used to compute the p-values for the comparisons displayed in the boxplots in Figure 6. Welch’s t-test is particularly suitable in this context as it accounts for unequal variances, ensuring the reliability of the statistical comparisons.

The bottom row of Figure 6 presents boxplots summarizing the distribution of total ToE for ethanol-water and methanol-water mixtures across temperatures. For ethanol-water mixtures, ToE decreases significantly with increasing temperature, reflecting faster system dynamics at higher temperatures. The p-values above the boxplots confirm statistically significant differences between temperature groups, such as the comparison between 25°C and 50°C (p=0.00039), demonstrating the substantial reduction in ToE at higher temperatures. Similarly, for methanol-water mixtures, ToE also decreases with rising temperature, though the reduction is less pronounced. Significant differences are observed between specific temperature pairs, such as 25°C vs. 50°C (p=0.0017) and 25°C vs. 60°C (p=0.000006), highlighting the temperature-dependent effects on methanol-water systems. The combination of normality, non-equal variance assumptions, and the use of Welch’s t-test allows for robust and reliable comparisons between temperature groups. These findings highlight the pronounced sensitivity of ethanol-water mixtures to temperature changes, with methanol-water mixtures showing more moderate but still statistically significant effects.

Overall, the LOESS model performs better than the linear model for both ethanol and methanol mixtures, as it captures the non-linear trends more accurately. Ethanol-water mixtures show stronger interactions with the sensor system, reflected in the steeper changes in Δt_2_ and capacitance compared to methanol-water mixtures. The boxplots further emphasize the temperature-dependent effects on total ToE, with statistically significant differences observed between temperature groups. These findings highlight the importance of considering temperature and concentration effects when analyzing such systems, with LOESS being the preferred model for achieving accurate results.

## Extra Statistical and Artificial Intelligence Analyses

We tested several additional modelling approaches to see whether the observed data trends could be described more precisely. In addition to the simple linear and LOESS fits used in the original version, we compared five methods: Linear, LOESS, Neural Network (NN), Generalized Additive Model (GAM), and Support Vector Regression (SVR). The updated results are shown in Figure S14.

Across all solvent systems and measurements, the LOESS, GAM, and SVR models provided smoother and more realistic curves that follow the experimental points closely, especially in the mid-range solvent mixtures where the data change most rapidly. These models confirmed the same nonlinear behavior discussed in the paper, but with better accuracy and less sensitivity to noise. In contrast, the Neural Network model did not perform as well because the dataset is relatively small; such models usually require many more observations to capture the pattern reliably.

Overall, these comparisons show that the main findings of the study remain valid and that the nonlinear trends we reported are genuine features of the system, not artifacts of the fitting method.

| 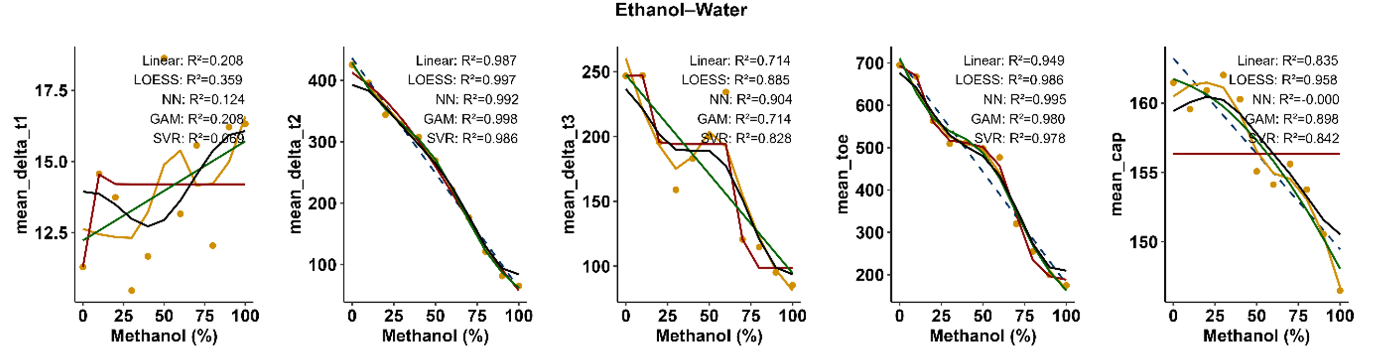 | | | | |
| --- | --- | --- | --- | --- |
| (i) | (ii) | (iii) | (iv) | (v) |
| (a) | | | | |
| 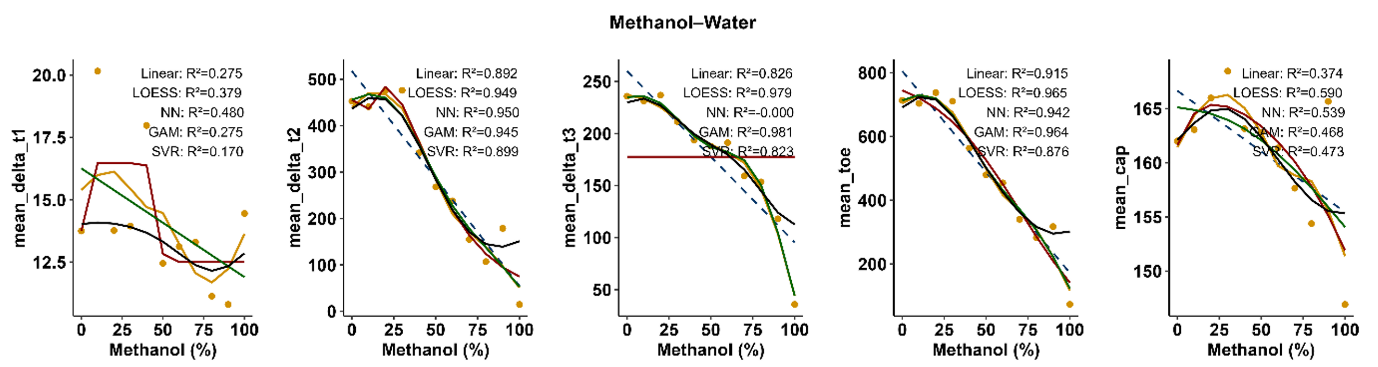 | | | | |
| (i) | (ii) | (iii) | (iv) | (v) |
| (b) | | | | |
| 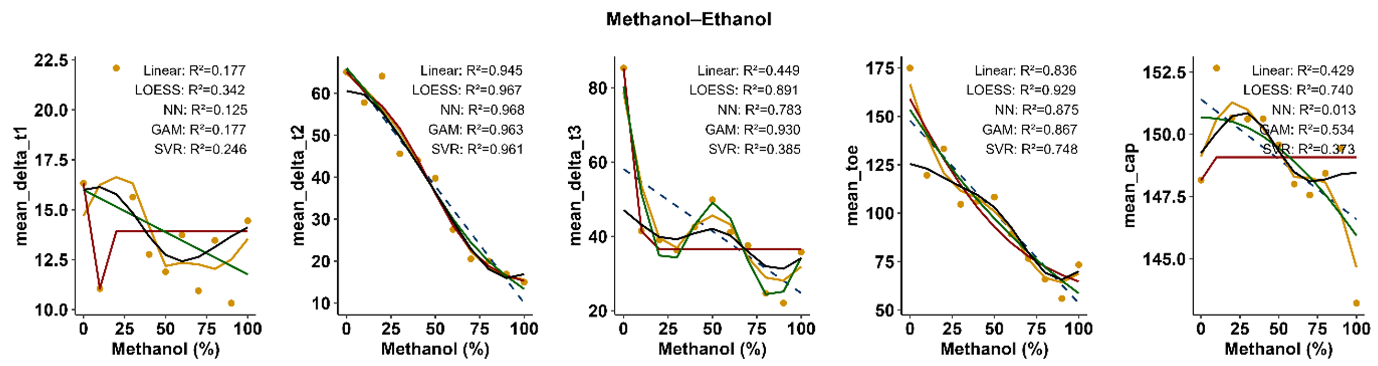 | | | | |
| (i) | (ii) | (iii) | (iv) | (v) |
| (c) | | | | |

**Figure S14.** Extra ML and statistical analysis of evaporation behavior for three binary solvent systems—(a) ethanol–water, (b) methanol–water, and (c) methanol–ethanol—across a range of solvent concentrations (0–100%). Each column displays a specific evaporation-related parameter plotted against solvent concentration: (i) initial evaporation interval (Δt₁), (ii) intermediate evaporation interval (Δt₂), (iii) total evaporation time (ToE), (iv) final evaporation interval (Δt₃) and (v) mean capacitance change (ΔCap). Experimental data (orange dots) are fitted using both linear regression (dashed blue) and LOESS smoothing (solid blue).

In another study, we developed and evaluated machine learning models to predict experimental outcomes under varying thermal and compositional conditions. The data set comprised measurements collected at four different temperatures (25 °C, 40 °C, 50 °C, and 60 °C), each tested through three repeated experimental runs. The experiments covered a full range of alcohol purities (0–100%) for water-ethanol, water-methanol and ethanol-methanol mixtures.

To capture the relationship between the measured features and target variables, two separate regression models were trained for methanol and ethanol. Several regression algorithms were initially considered, including linear regression, support vector regression, decision tree regression, and polynomial regression. Among these, the polynomial regression model of degree three demonstrated superior predictive performance across all evaluation metrics and was therefore selected as the final model.

Due to the limited number of available samples, a stratified data selection strategy was applied to ensure balanced representation across the experimental space. Specifically, two samples from each temperature–purity combination were used for model training, while the remaining data were reserved for model evaluation. This procedure yielded a total of 44 samples for testing, allowing for comprehensive coverage of the experimental conditions.

Feature engineering focused on transforming raw time-interval data (Δt₁–Δt₃, ToE, ΔCap) into compact descriptors such as their sum, mean, and variance, together with temperature. These derived features improved generalization and reduced noise compared with the direct use of raw intervals.

Figures S15 to S17 illustrate the modeling performance for ethanol and methanol concentrations in water–ethanol water–methanol mixtures and ethanol-methanol mixtures. Model accuracy was assessed using the R² and the RMSE, metrics that evaluate predictive generalization rather than curve-fitting quality. Training and testing were repeated multiple times to account for variability. For water-methanol, the model achieved an average R^2^ = 0.932 $\pm$ 0.014 and RMSE = 0.814 $\pm$ 0.087, while for water-ethanol, the performance reached R^2^ = 0.941 $\pm$0.022 and RMSE = 0.225 $\pm$ 0.144. For methanol-ethanol, R^2^=0.765$\pm$0.093 and RMSE=1.497$\pm$0.326. These results confirm that the polynomial model effectively captures the non-linear dependencies identified experimentally.

**
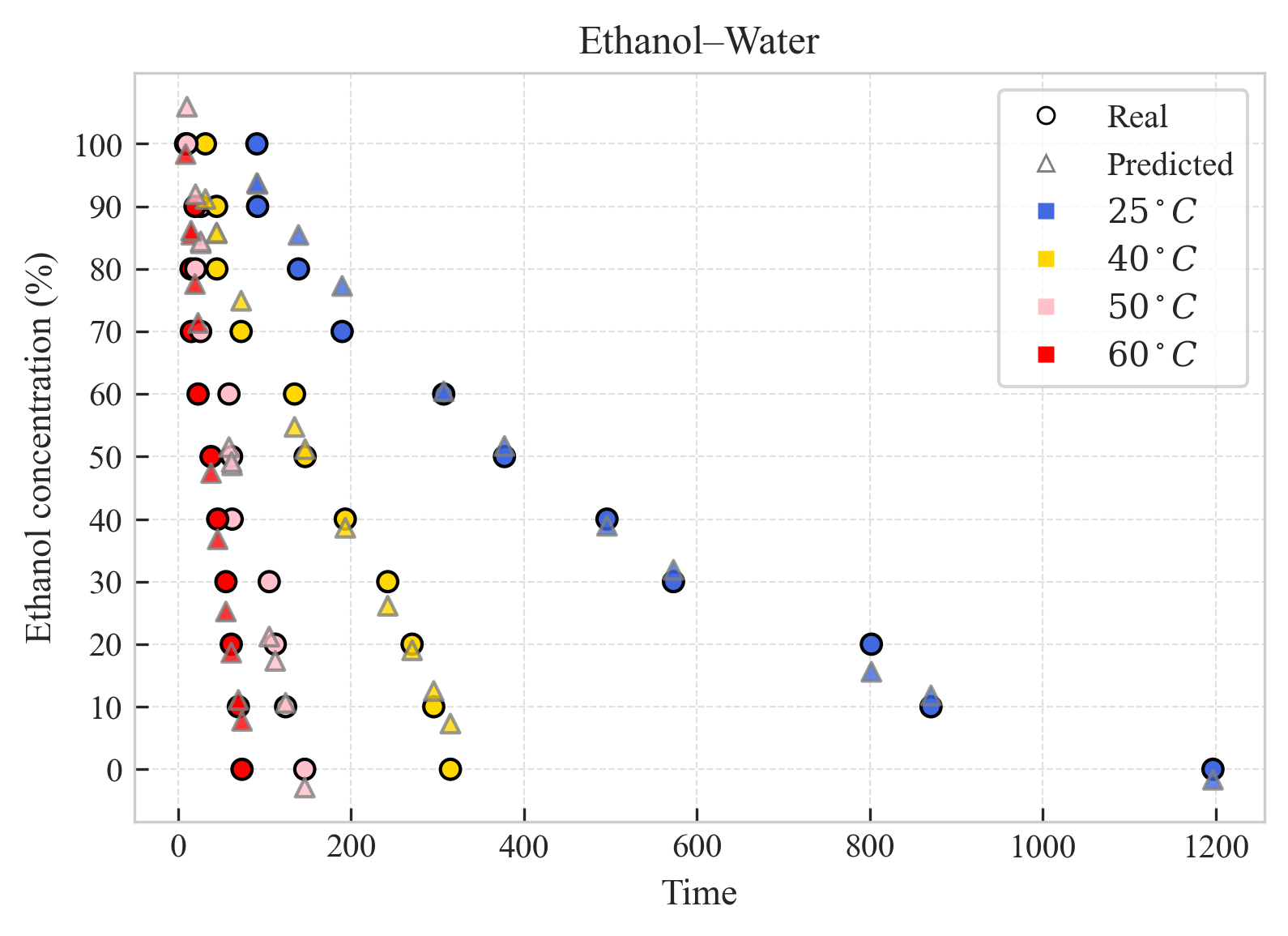
**

**Figure S15.** Predictive modeling of ethanol concentration in water-ethanol mixtures.

**
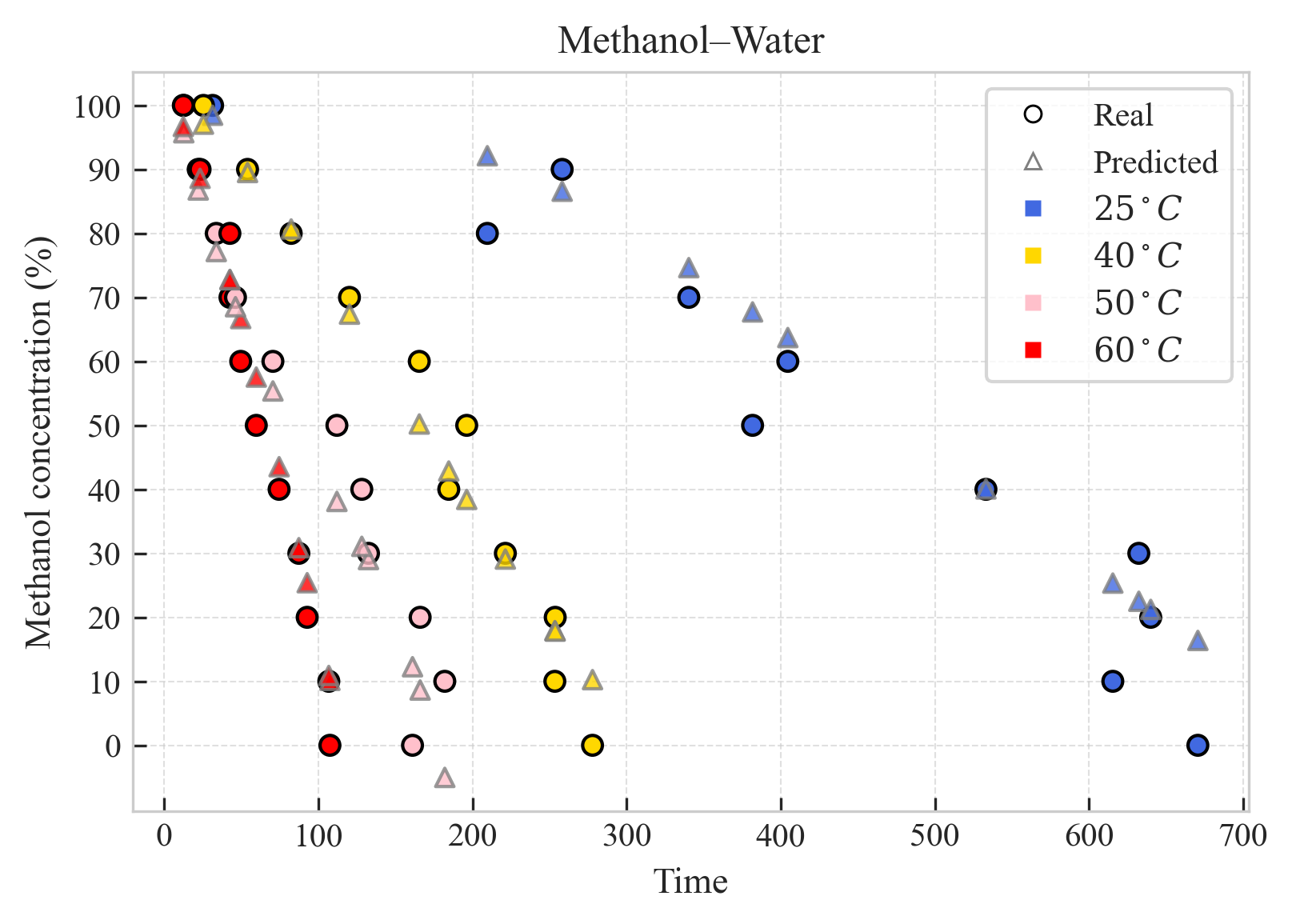
**

**Figure S16.** Predictive modeling of methanol concentration in water-methanol mixtures.

**
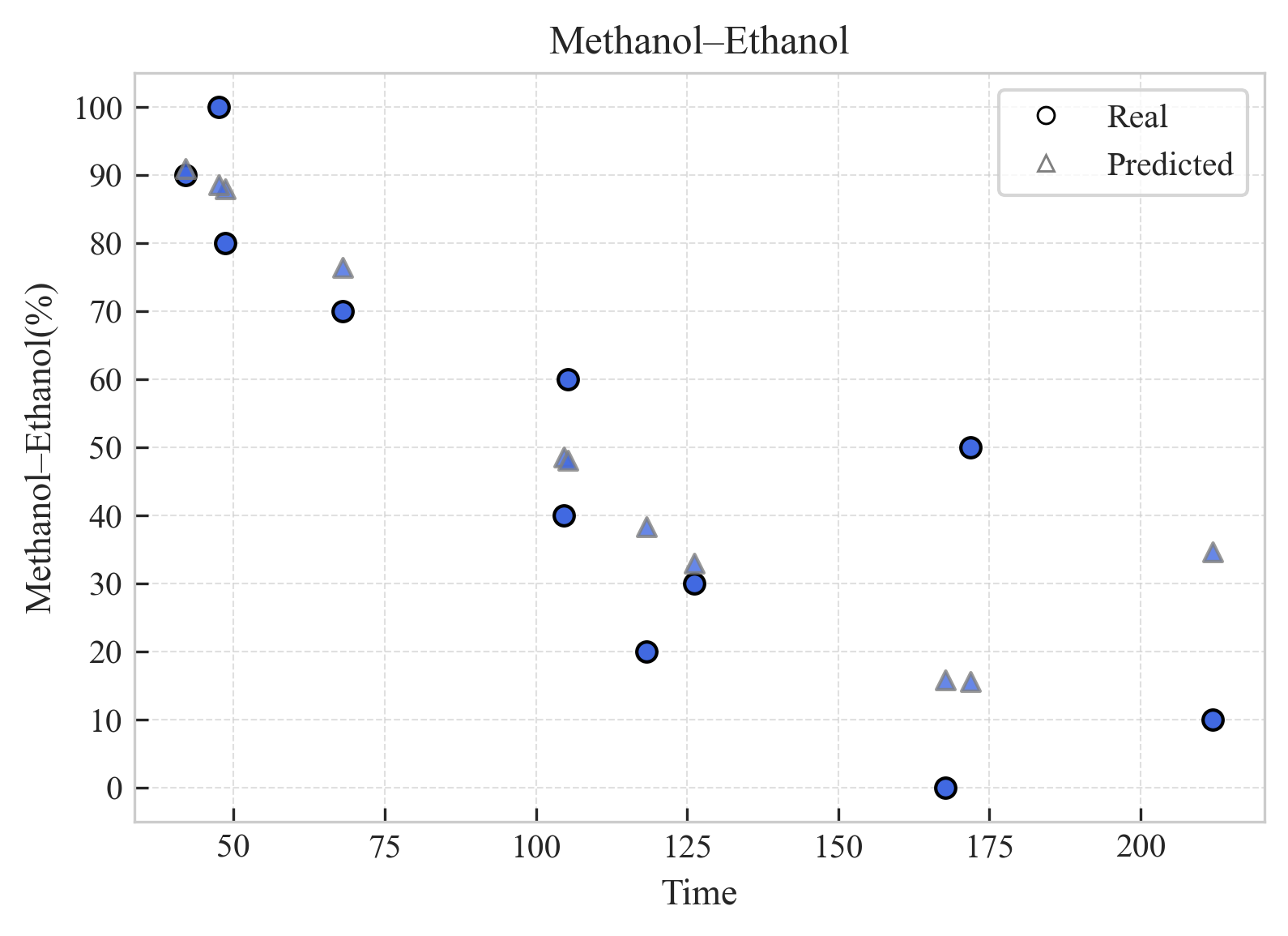
**

**Figure S17.** Predictive modeling of ethanol concentration in ethanol-methanol mixtures.

# Numerical Simulations

A COMSOL Multiphysics simulation was conducted to model the static electrical response of a CMOS capacitive sensor in both air and liquid conditions, to understand the resulting capacitance and electric fields around the sensor. The sensor was initially designed in a CAD environment within COMSOL and then incorporated into the electrostatic module, with specific size and physical boundary conditions replicating the experimental setup of the fabricated sensor. The simulation consists of an interdigitated electrode on the substrate of SiO_2_, and the electrodes are exposed to an air environment. To solve the electrostatic Maxwell equations with Finite Element Method (FEM) approximations. The domain required for the simulation of the single electrode and the meshed structure is shown in Figure S18. The sensor and the air box have meshed with a triangular meshing structure with a minimum mesh size of 1 µm and a maximum of 20 µm with a growth rate of 1.2 to give the mesh-independent result. In Figure S18, the mesh size is presented in 3D to show how small the mesh is around the micro-sized electrodes and the growth rate to the medium box. The simulated electrode size has been adapted to the fabricated sensor to be 20 µm, 5 µm, and 0.9 µm in length, width, and thickness, respectively.

Figure S18 shows that the sensor has been operated by applying a voltage of 2.7 V to one electrode while grounding the other terminal, resulting in a capacitance of 4.885 fF based on the biasing. However, the COMSOL simulation predicts a capacitance with a certain degree of mismatch compared to the experimental data due to non-idealities, such as the effect of material properties (e.g., Si_3_N_4_ and SiO_2_ layers) on the fabricated sensor and the inherent limitation of FEM method. As illustrated in Figure S18, an electric field is present within a certain distance from the sensor surface, which can be modulated by new material on the sensor. When a liquid is placed on the sensor surface, due to the change in the dielectric of the medium of the capacitive sensor, the resultant capacitance changes; however, there should be a threshold for the thickness of the materials beyond which the sensor’s response is saturated. The streamlines shown in Figure S18 evidence of this expectation of how far the sensor's screening length is.

| 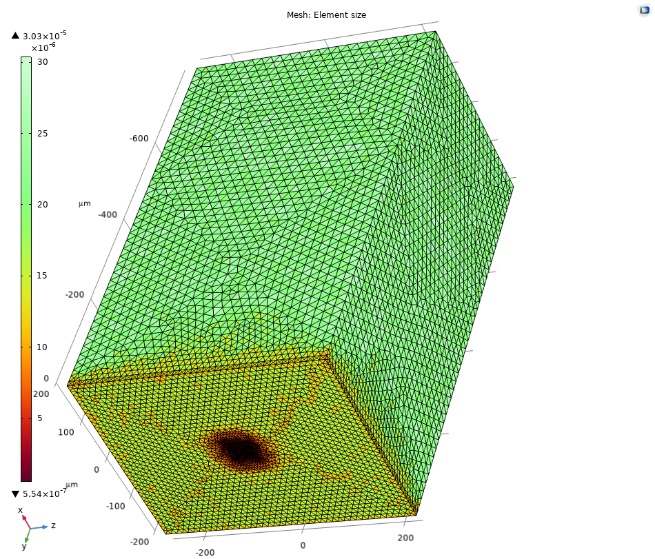 | 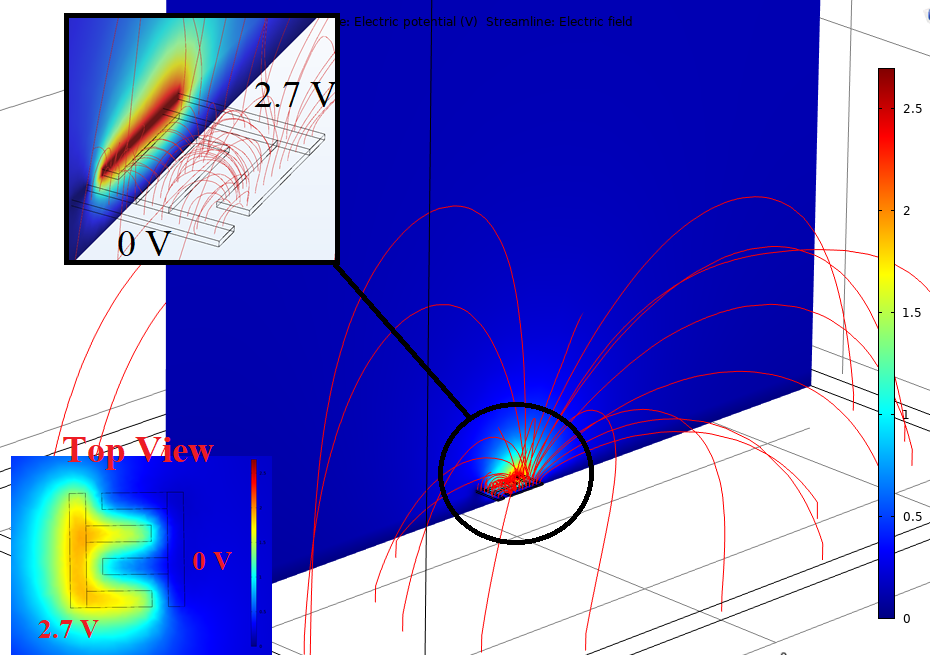 |
| --- | --- |

**Figure S18.** The simulated interdigital CMSO sensor. (a): the meshed structure of the sensor in a box of air. (b): the middle-slice plan counter of electric potential around the sensor when a bias of 2.7 V is applied.

To further demonstrate this concept and verify the experimental pattern of liquid evaporation on the sensor, we modeled the decrement in the thickness of a liquid on the sensor. We reduced the length from 500 µm to 0 to see the screening length. The sensor’s response pattern was derived to estimate the CMOS sensor’s capacitance response saturation region. The simulation results showed that the sensor gets saturated when the thickness of the liquid is beyond a specific limit. In this context, we call it “capacitance screen length” (CSL), which refers to the extent existence of a dielectric on the CMOS capacitance sensor can be detected. Based on Figure S19, the simulation results clearly demonstrate that with liquid's evaporation, the capacitance decreases to its dry mode capacitance base level. The evaporation pattern is explicitly modeled as a reduction in the thickness of a 0.5 mm^2^ vertical fluid element on top of the CMOS sensor. According to Figure S19. the sensor response gets saturated for a liquid droplet with a thickness of more than ~ 100 µm. In this region, the sensor response is primarily modulated by the dielectric constant of liquid (or any material) on the sensing site. This concept has been clearly shown in the results provided in Figure S19, as in the saturation region with an increase in the ethanol concentration, the capacitance decreases while the liquid still exists on the CMOS sensor. The experiments have validated this, showing a unique CMOS capacitive sensor sensing modality with promises in many life sciences applications.

According to Figure S19, the relative dielectric of water decreases with the addition of ethanol. The relation presented in Figure S19b has been derived based on the Lorentz-Lorenz relation for ethanol-water mixture. The resultant dielectric (relative permittivity) was utilized in the COMSOL simulation. The resultant capacitance versus liquid thickness and relative permittivity effects were analyzed to demonstrate the sensor operation as seen in Figure S19. The total capacitance decreases in all ranges of the liquid thickness on top of the sensor both in saturation mode and in the range of CSL. To better understand the CSL threshold thickness, the derivative of capacitance versus liquid thickness was plotted against the liquid thickness to assess the onset of the saturation, after which the dielectric variation of liquid influences the sensor’s response. Figure S19c shows that the threshold for water and all ethanol concentrations can be considered between ~ 30 µm to 40 µm. This number helps a lot in the prediction of the level of liquid thickness down to the 30-40 µm range with the evaporation-on-CMOS technique.

To better understand this concept, the electrical potential has been calculated along a line, as shown in Figure S19d. According to the results (Figure S19d), the decaying pattern of electrical potential further consolidates the CSL for the CMOS sensor, which shows different sensitivity to the existence of a dielectric in relation to distance from the sensor surface.

| 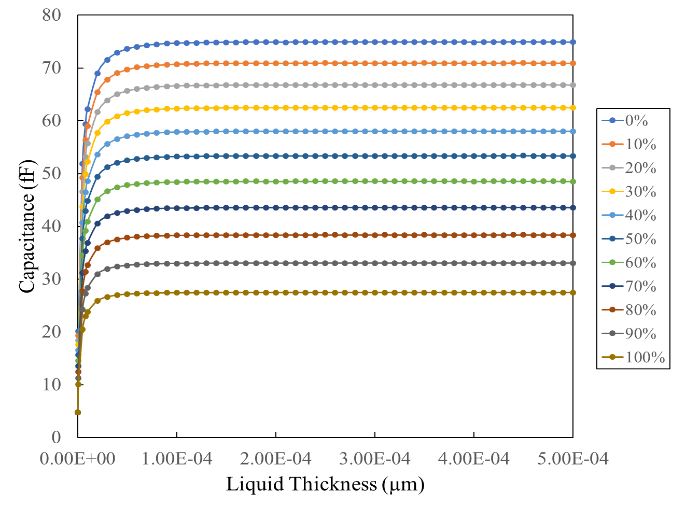 | 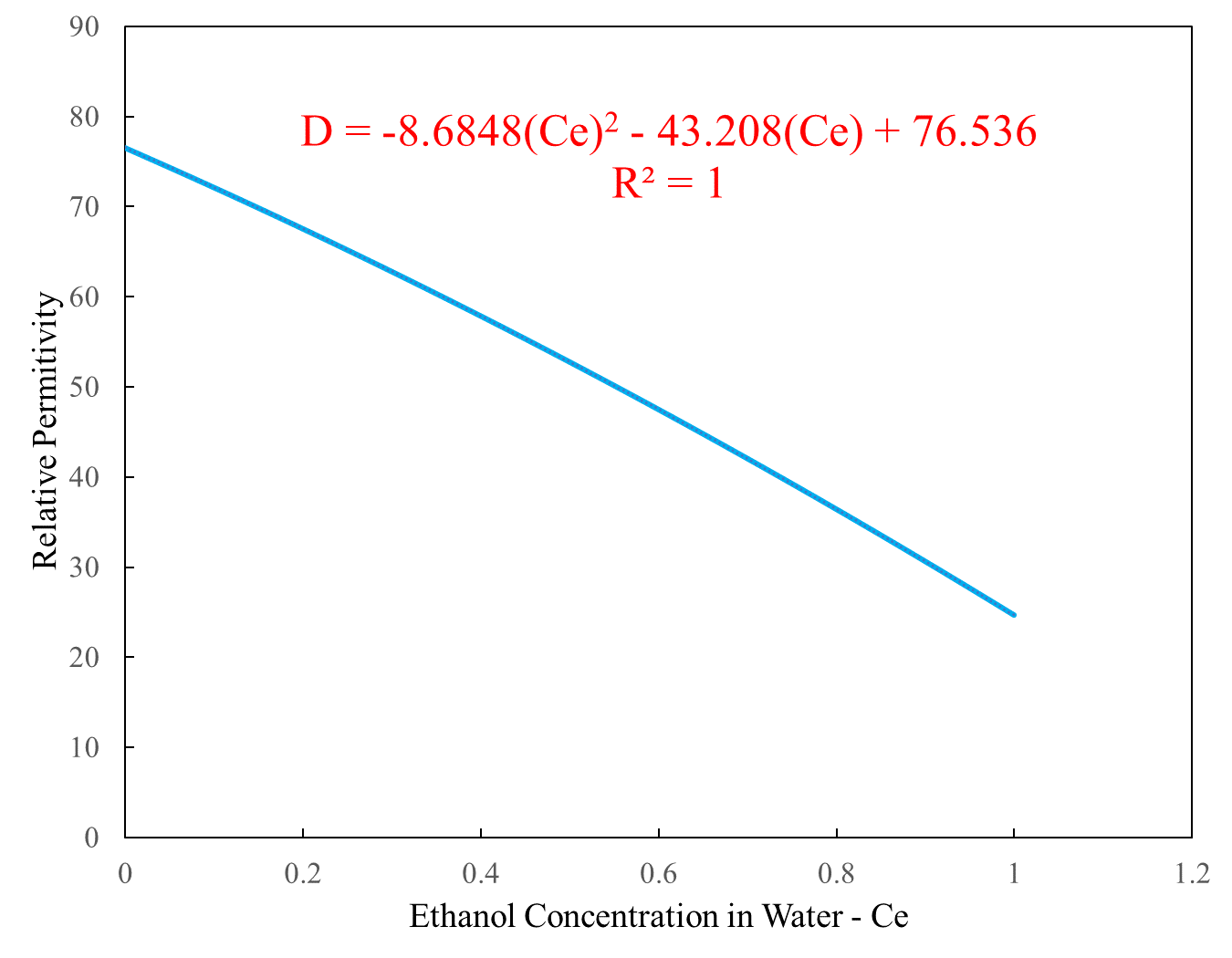 |
| --- | --- |
| 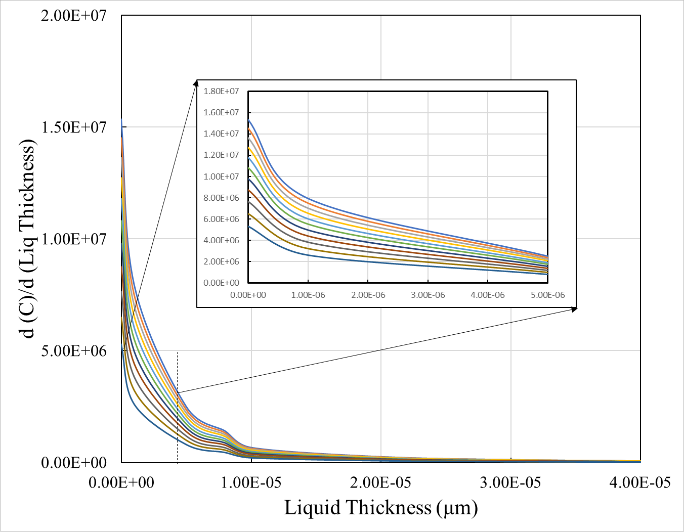 | 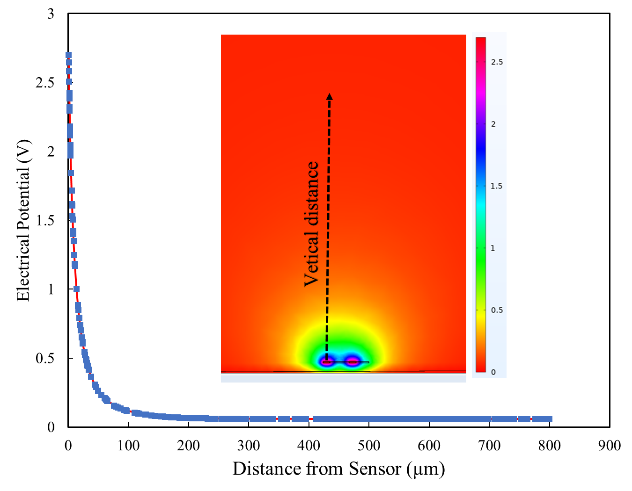 |

**Figure S19.** (a): The CMOS response to the water-ethanol liquid droplet on the sensor for different concentrations of ethanol in solution. The liquid thickness represents the evaporation of liquid explicitly. (b): present the relative permittivity (dielectric) of a water-ethanol mixture based on an empirical model used in COMSOL simulation.

# CMOS Interface Circuit

The designed and implemented chip for capacitive sensing relies on a charge-based capacitance measurement (CBCM) as shown in Figure S20. The system shown in this figure implements a fully integrated current-mode core-CBCM circuit designed for high-resolution capacitive sensing. The architecture converts the difference between a sensing capacitance (*C*_IDE_) and a reference capacitance (*C*_R_) into a digital output. The structure comprises five main functional blocks: (1) a differential core-CBCM capacitance-to-current converter, (2) a programmable capacitor bank for *C*_R_, (3) an opamp-based current interface circuit, (4) a current-controlled oscillator (CCO), and (5) a counter-serializer for digital readout. The principle of operation relies on controlled charging and discharging of capacitive branches, followed by precise current processing and frequency encoding.

**Figure S20.** A current-mode core-CBCM circuit designed for high-resolution capacitive sensing: (i) the CBCM core for capacitance-to-current conversion, (ii) Applied clock pulses and output current waveforms of the CBCM core, (iii) a programmable capacitor bank for C_R_, (iv) an opamp-based current interface circuit, (v) a CCO, and (vi) a counter-serializer for digital readout.

At the heart of the circuit is the CBCM core, as shown in Figure S16 (i), which utilizes two pseudo-inverter branches to control the charge and discharge cycles of the sensing and reference capacitors, *C*_IDE_ and *C*_R_, respectively. These pseudo-inverters are composed of pull-up and pull-down transistors that are driven by non-overlapping clock phases Φ_1_ and Φ_2_, ensuring controlled switching without short-circuit currents (Figure S16 (ii)). When the clock pulses transition, the capacitors are charged to a known voltage level and subsequently discharged through active switching. This process results in sharp transient exponential currents through each capacitor branch, denoted as *i*_S_(*t*, *C*_IDE_) and *i*_R_(*t*, *C*_R_), which are directly related to the capacitance values.

Mathematically, the instantaneous currents flowing through the sensing and reference branches are governed by the following expressions:

| $i_{S}\left( t \right)=C_{IDE}\frac{dv_{s}(t)}{dt}$ | (1) |
| --- | --- |
| $i_{R}\left( t \right)=C_{R}\frac{dv_{R}(t)}{dt}$ | (2) |

Here, *v*_S_(t) and *v*_R_(t) are the instantaneous voltages across *C*_IDE_ and *C*_R_, respectively. These currents are sharp and time-limited, resembling exponential spikes that repeat every switching period *T*_s_​. By averaging these currents over a complete clock cycle, the average values *I*_S_ ​ and *I*_R_​ are derived:

| $I_{S}=\frac{1}{T_{s}}\int_{0}^{T_{s}} C_{\mathrm{IDE}}\frac{dv_{s}\left( t \right)}{dt}dt$ | |  |
| --- | --- | --- |
|  | $=\frac{C_{\mathrm{IDE}}}{T_{s}}\int_{0}^{V_{\mathrm{dd}}-V_{Th}} dv_{s}$ |  |
|  | $=\frac{{C_{\mathrm{IDE}}(V}_{\mathrm{dd}}-V_{Th})}{T_{s}}$ | (3) |
| $I_{R}=\frac{1}{T_{s}}\int_{0}^{T_{s}} C_{R}\frac{dv_{R}\left( t \right)}{dt}dt$ | |  |
|  | $=\frac{C_{R}}{T_{s}}\int_{0}^{V_{\mathrm{dd}}-V_{Th}} dv_{s}$ |  |
|  | $=\frac{{C_{R}(V}_{\mathrm{dd}}-V_{Th})}{T_{s}}$ | (4) |

In these expressions, *V*_dd_​ is the supply voltage, and *V*_Th_ ​ is the threshold voltage of the transistors used in the current mirrors, P_4_ and P_3_, connected to the pull-up switches of the CBCM core. It can be easily shown that the difference between the two averaged currents is proportional to the capacitance difference Δ*C*=*C*_IDE_−*C*_R_ ​:

| $I_{S}-I_{R}=f_{S}\left( V_{\mathrm{dd}}-V_{\mathrm{Th}} \right).\Delta C$ | (5) |
| --- | --- |

where *f*_s_=1/*T*_s_ ​ is the clocking frequency. This is the principle of core-CBCM circuits. In the circuit shown in Figure S20, first we generate the instantaneous currents, *i*_S_(*t*, *C*_IDE_) and *i*_R_(*t*, *C*_R_), using the CBCM core (Figure S20 (i)), then amplify and subtract them using the curernt mirrors to generate the intantaneous differential current, *i*_out_. This differential current is then processed in the current interface circuit shown in Figure S20 (iv), which incorporates a feedback path comprising N_5_, N_3_, and an OpAmp to enforce the drain-source voltage of N_3_ (*V*_D_) to track the drain-source voltage of N_4_ (*V*_C_), regardless of the value of *C*_IDE_.

The amplified differential current, *i*′_out_, is then fed into a CCO as depicted in Figure S20 (v). The CCO translates the amplitude of the input current into an oscillation frequency, such that:

| $f_{\mathrm{CCO}}\left( t, C_{\mathrm{IDE}}{,C}_{R} \right)=A_{\mathrm{CCO}}.{i'}_{\mathrm{out}}\left( t, C_{\mathrm{IDE}}{,C}_{R} \right)$ | (6) |
| --- | --- |

Here, *A*_CCO_ ​ is the gain of the CCO, and the output frequency *f*_CCO_ directly reflects the capacitance difference Δ*C*.

Note that, in CBCM method, the average of the current is proportional to the capacitance. So, we need to integrate the instantaneous current or equivalently the current-modulated frequency. To average this frequency in the digital domain, the output pulses of the CCO are counted by the counter-serializer block (Figure S20 (vi)). Over a defined integration time *T*_int_, the number of pulses *N*_count_ is proportional to the integral (or average) of the frequency. This final digital count is serialized and read out as the sensor’s output, representing the magnitude of Δ*C*. Since the system is inherently differential, it offers resilience against common-mode disturbances such as temperature drift, supply variation, and substrate noise.

The bank of capacitors shown in Figure S20 (iii), implemented in the reference path in place of a single fixed *C*_R_​, enables on-chip programmability and calibration through dynamic sweeping of the reference capacitance. This is especially valuable in biosensing applications, where dynamic range and calibration-free operation are critical. The ability to periodically sweep *C*_R_ enables both static and time-variant offset capacitance effects (e.g., sedimentation, fouling) to be compensated or visualized, generating a two-dimensional signature that reveals the presence of both analyte-induced signals and background artifacts.

Hence, the employed system performs fully integrated capacitance-to-digital conversion in a compact and power-efficient fashion. The use of current-mode readout, differential architecture, programmable reference capacitance, and frequency-domain digitization enhances the sensor’s resolution, dynamic range, and robustness against non-idealities.

# Analytical Modeling of Binary Droplet Evaporation

To model the evaporation of binary droplets under varying temperature and pressure, thermodynamic equations of state are combined with mass and heat transfer models. However, detailed changes in thermodynamic conditions within and around the droplet are rarely addressed in the literature. Due to the complexity of binary mixtures, each droplet composition requires a tailored analysis based on its unique thermophysical properties and component interactions. These models involve complex assumptions and boundary conditions and often face challenges due to nonlinear behavior and multicomponent interactions. To better understand the process, a representative binary droplet is analyzed using governing equations for mass and heat transfer in both gas and liquid phases, capturing key transport phenomena and gradients.

Experimental validation is essential to verify and refine these theoretical models, providing empirical data, highlighting model limitations, and ensuring alignment with physical reality for practical applications. The general equations used for the analytical modeling of a binary droplet undergoing evaporation are presented below. The axisymmetric model, formulated in spherical coordinates, assumes spherical symmetry and thermodynamic equilibrium at the vapor–liquid interface. Effects of gravity and gas solubility in the liquid are neglected ^1-3^.


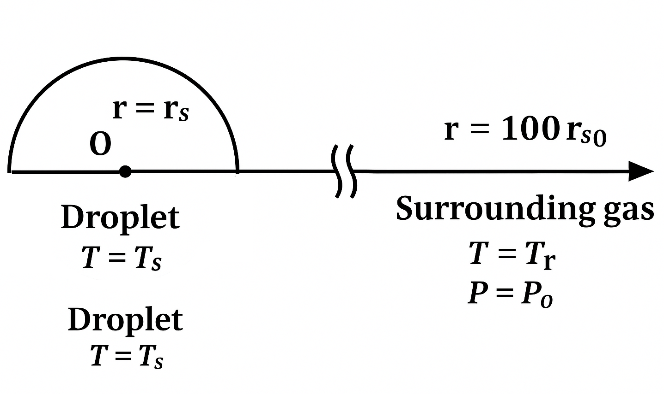


**Figure S21.** Schematic of 2-component droplet and environment.

The governing equations in the gas and liquid phase are expressed as follows:

- Species Conservation:

| $\frac{\partial\left( \rho Y_{i} \right)}{\partial t}+\left( \frac{1}{r^{2}} \right)\frac{\partial\left( r^{2} \rho uY_{i} \right)}{\partial r}=\left( \frac{1}{r^{2}} \right)\frac{\partial\left( r^{2}\rho D_{i}\frac{\partial Y_{i}}{\partial r} \right)}{\partial r}$ | (7) |
| --- | --- |
| $\frac{\partial\left( \rho X_{i} \right)}{\partial t}=\left( \frac{1}{r^{2}} \right)\frac{\partial\left( r^{2}\rho D_{i}\frac{\partial X_{i}}{\partial r} \right)}{\partial r}$ | (8) |

- Momentum Conservation:

| $\frac{\partial\left( \rho u \right)}{\partial t}+\left( \frac{1}{r^{2}} \right)\frac{\partial\left( r^{2} \rho u^{2} \right)}{\partial r}=-\frac{\partial p}{\partial r}+\left( \frac{1}{r^{2}} \right)\frac{\partial\left( r^{2}\mu\frac{\partial u}{\partial r} \right)}{\partial r}$ | (9) |
| --- | --- |

- Energy Conservation:

| $\frac{\partial\left( \rho h \right)}{\partial t}+\left( \frac{1}{r^{2}} \right)\frac{\partial\left( r^{2} \rho u h \right)}{\partial r}=\left( \frac{1}{r^{2}} \right)\frac{\partial\left( r^{2}k\frac{T}{\partial r} \right)}{\partial r}$ | (10) |
| --- | --- |
| $\frac{\partial\left( \rho Cp T \right)}{\partial t}=\left( \frac{1}{r^{2}} \right)\frac{\partial\left( r^{2}k\frac{T}{\partial r} \right)}{\partial r}$ | (11) |

Where, ρ: density, X_i_, Y_i_: mass fraction of species I in liquid and gas phase, u: radial velocity, D_i_: diffusion coefficient, μ: dynamic viscosity, h: specific enthalpy, k: thermal conductivity, T: temperature. The thermophysical properties are calculated using the Peng-Robinson equation of state. For liquid phase equations, internal motion is neglected in the liquid phase; only diffusion and heat conduction are considered. Boundary and initial conditions are: at the droplet center (r = 0), ∂T/∂r = 0, ∂Yi/∂r = 0, at the interface (r = R), mass flux continuity: ρ_g_ D_g_ (∂Y_i_/∂r)g = ρ_l_ D_l_ (∂Y_i_/∂r)_l_, heat flux continuity: kg (∂T/∂r)g = k_l_ (∂T/∂r)_l_  , thermodynamic equilibrium: $f_{i}^{g}=f_{i}^{l}$ , evaporation rate: ṁ = ρ D_i_ (∂Y/∂r)|r=R, radius change: dR/dt = -ṁ/ρ.

The numerical solution algorithm begins with initializing the surface temperature and the mass flux at the droplet interface. Following this, the relevant thermophysical properties and fugacities of all components are computed using appropriate equations of state. These values are then used to solve the governing equations for both the gas and liquid phases in spherical coordinates ^4-6^. After solving, the algorithm checks for convergence in the interfacial values, such as temperature and species composition. If the convergence criteria are met, the droplet radius is updated based on the calculated evaporation rate. The simulation then advances to the next time step, and the process is repeated iteratively.

At the gas-liquid interface, thermodynamic equilibrium is assumed. This equilibrium is governed by the equality of fugacities of each component in both phases. For a multicomponent system, this condition is expressed as:

| $f_{i}^{g}=f_{i}^{l}$ | (12) |
| --- | --- |

Where, $f_{i}^{g}$: Fugacity of component i in the gas phase and $f_{i}^{l}$: Fugacity of component i in the liquid phase. The fugacity of each component is determined using the Peng–Robinson equation of state (PR-EoS). The fugacity in each phase is given by:

| $f_{i}^{g}=\varphi_{i}^{g}x_{i}^{g}P$ | (13) |
| --- | --- |
| $f_{i}^{l}=\varphi_{i}^{l}x_{i}^{l}P$ | (14) |

Where $\varphi_{i}^{g}\varphi_{i}^{l}$ : fugacity coefficients for i species in gas and liquid phases (from PR-EoS) and $x_{i}^{g}x_{i}^{l}$ : mole fraction of component i in each phase and P: Total pressure. By equating the two fugacities, we obtain:

| $\varphi_{i}^{g}x_{i}^{g}P=\varphi_{i}^{l}x_{i}^{l}P$ | (15) |
| --- | --- |

This relation is solved iteratively to obtain the interfacial composition for both phases. After computing the evaporation rate, the gas velocity at the droplet surface is calculated using:

| $u_{g}\left( r \right)=\dot{\frac{m}{\left( \rho_{g}\times A \right)}}$ | (16) |
| --- | --- |

Where, u_g_(R): gas velocity at the droplet surface, ṁ: total evaporation rate, ṁ = Σ ṁ_i_, where ṁ_i_ is the evaporation rate of species I, ρ_g_: gas density at the surface and A: surface area of the droplet. To compute the droplet radius at any time step, the following equation is used:

| $\frac{dR\left( t \right)}{dt}=\frac{-\dot{m}}{4\pi R^{2}\rho_{lm}}+\frac{R\left( t \right)}{3\rho_{lm}}\frac{d\rho_{lm}}{dx}$ | (17) |
| --- | --- |

Where, dR/dt: time derivative of droplet radius, ṁ: evaporation rate, ρ_lm_: Liquid density, R: droplet radius, dρ_l_/dt: rate of change of liquid density. The first term on the right-hand side represents the effect of evaporation in reducing the droplet radius. Accordingly, a droplet diameter–time curve is constructed to evaluate the duration required for complete droplet evaporation. The second term accounts for the effect of density variation, which includes thermal expansion.

**Figure S22.** Drop volume evolution with time for pure substances (water and ethanol) and mixtures for three different concentrations (25% ethanol, 50% ethanol, 75% ethanol) ^7^.

To validate the results of our numerical model, we compared our findings with the experimental data reported by Sefiane et al. (2003) ^7^, who investigated the evaporation dynamics of water–ethanol binary droplets on PTFE substrates. Their study identified a characteristic three-stage evaporation behavior, which closely aligns with the trends observed in our simulations, particularly the dependence of contact angle, volume, and base width evolution on ethanol concentration. The qualitative agreement between the experimental results and our computational predictions further supports the reliability of our model in capturing key aspects of binary droplet evaporation under ambient conditions ^7-9^.

# References

1 Arabkhalaj, A., Azimi, A., Ghassemi, H. & Markadeh, R. S. A fully transient approach on evaporation of multi-component droplets. *Applied Thermal Engineering* **125**, 584–595 (2017).

2 Zhu, G.-S., Reitz, R. D. & Aggarwal, S. K. Gas-phase unsteadiness and its influence on droplet vaporization in sub-and super-critical environments. *International Journal of Heat and Mass Transfer* **44**, 3081–3093 (2001).

3 Reid, R., Prausnitz, J. & Poling, B. *The properties of gases and liquids*. 4 edn, (McGraw-Hill, 1987).

4 Sazhin, S. *et al.* Radiative heating of semi-transparent diesel fuel droplets. *J. Heat Transfer* **126**, 105–109 (2004).

5 Abramzon, B. & Sazhin, S. Droplet vaporization model in the presence of thermal radiation. *International Journal of Heat and Mass Transfer* **48**, 1868–1873 (2005).

6 Poling, B. E., Prausnitz, J. M. & O'connell, J. P. *The properties of gases and liquids*. 5 edn, (Mcgraw-hill, New York, 2001).

7 Sefiane, K., Tadrist, L. & Douglas, M. Experimental study of evaporating water–ethanol mixture sessile drop: influence of concentration. *International journal of heat and mass transfer* **46**, 4527–4534 (2003).

8 Riazi, M. *Characterization and properties of petroleum fractions*. 1 edn, Vol. 50 (ASTM international, 2005).

9 Yaws, C. L. *Handbook of thermodynamic diagrams: volume and enthalpy diagrams for major organic chemicals and hydrocarbons*. Vol. 2 (Elsrvier, Texas, 1996).
