# Supplementary material for: Complementary metal-oxide-semiconductor (CMOS) time of evaporation measurement system for binary chemical monitoring
Source: Sci Rep. 2026 Jan 23;16:5542. doi: 10.1038/s41598-026-35322-x (PMC12887047; doi:10.1038/s41598-026-35322-x)
Supplement: Supplementary file 2 — Supplementary Material 2 [file 41598_2026_35322_MOESM2_ESM.html]

Complementary Metal-Oxide-Semiconductor (CMOS) Time of Evaporation Measurement System for Binary Chemical Monitoring


# Complementary Metal-Oxide-Semiconductor (CMOS) Time of Evaporation Measurement System for Binary Chemical Monitoring

#### **Ebrahim Ghafar-Zadeh¹\***, **Saghi Forouhi²\***, Hamed Osouli Tabrizi¹, Abbas Panahi¹, Yasaman Tahernezhad¹, Azadeh Amrollahi Biyouki¹ ¹ Biologically Inspired Sensors and Actuators Laboratory (BioSA Lab), Department of Electrical Engineering and Computer Science, Lassonde School of Engineering, York University, Toronto, ON, M3J 1P3, Canada ² Division of Electronics and Computer Engineering (ELDA), Department of Electrical Engineering (ISY), Linköping University, 581 83 Linköping, Sweden

# 1. All combinations

## 1.1 Figure3

```
# Ethanol
#_________________________
rm(list=ls())

#_______________________
# Libraries
#_______________________
library(nortest)
library(writexl)
library(tidyverse)
library(openxlsx)
library(dplyr)
library(Matrix)
library(lme4)
library(ggplot2)
library(car)
library(extrafont)
library(stringr)
library(gridExtra)
library(combinat)
library(tidyr)
library(cowplot)
library(ggpubr)
library(rlang)
library(broom)
library(purrr)
library(magick)
library(ggpmisc)  # For adding equation and R² to plot
# Import fonts
# font_import(prompt = FALSE)
loadfonts(device = "win")

get_sharpness_time <- function(data) {
  # Loop through the data until the cap value increases significantly
  for (i in 2:nrow(data)) {
    # Check if there's a notable increase in the Cap value after a steady range
    if (data$Cap[i] > 160) {
      return(data$Time[i])
    }
  }
  
  # Return NA if no sharpness time is found
  return(NA)
}

calculate_max_time_for_run <- function(data, df2) {
  # Extract the first selected run from df2
  selected_run <- str_split(df2$combination[1], ", ")[[1]][1]
  
  # Process the data to calculate cumulative time for the selected run
  selected_data <- data %>%
    mutate(Condition = str_replace(Condition, "-run", "_run")) %>%
    filter(Condition == paste0(df2$condition[1], "_", selected_run)) %>%
    group_by(Condition) %>%
    mutate(Cumulative_Time = cumsum(Time)) %>%
    ungroup()
  
  # Return the maximum cumulative time for the selected run
  return(max(selected_data$Cumulative_Time))
}


process_selected_data <- function(data, temp_label, df, df2, max_time_templabel) {
  
  # Group and calculate cumulative time
  data <- data %>%
    group_by(Condition) %>%
    mutate(Cumulative_Time = cumsum(Time)) %>%
    ungroup() %>%
    mutate(Time = Cumulative_Time) %>%
    select(-Cumulative_Time)
  
  # Replace "-run" with "_run" in the Condition column to ensure uniformity
  data <- data %>%
    mutate(Condition = str_replace(Condition, "-run", "_run"))
  
  # Extract Run_Num as a numeric value from Condition
  data <- data %>%
    mutate(Run_Num = as.numeric(str_extract(Condition, "\\d+$")))
  
  # Ensure df2 and df have consistent column names
  df$condition <- factor(df$condition)
  
  # Initialize a list to store plots
  plot_list <- list()
  
  # Define condition mapping for Pure Water (0) and Pure Ethanol (100)
  condition_map <- c("Pure Water" = 0, "Pure Ethanol" = 100)
  for (i in 1:99) {
    condition_map[paste0(i, "% Ethanol")] <- i
  }
  
  # Define gradient colors for R1, R2, R3
  gradient_colors_R1 <- colorRampPalette(c("#6CA6CD", "#000080"))(101)  # Light blue to navy blue
  gradient_colors_R2 <- colorRampPalette(c("#E396A5", "#8B0000"))(101)  # Light red to dark red
  gradient_colors_R3 <- colorRampPalette(c("#78D978", "#006400"))(101)  # Light green to dark green
  
  # Define fixed colors for R1, R2, and R3 legend (e.g., midpoints or other meaningful colors)
  fixed_colors <- c(
    "R1" = gradient_colors_R1[51],  # Midpoint of R1 gradient (50% Ethanol)
    "R2" = gradient_colors_R2[51],  # Midpoint of R2 gradient (50% Ethanol)
    "R3" = gradient_colors_R3[51]   # Midpoint of R3 gradient (50% Ethanol)
  )
  
  # Loop over each condition to align data and create plots
  for (condition in unique(df2$condition)) {
    # Get the corresponding rows for the current condition from df2
    df2_subset <- df2 %>% filter(condition == !!condition)
    
    # Initialize a list to store the aligned data for each run
    condition_aligned_data <- list()
    selected_runs <- unlist(strsplit(as.character(df2_subset$combination[1]), ", "))
    
    # Get the condition percentage from the mapping
    condition_percentage <- condition_map[condition]
    
    # Assign colors and linetypes based on the run order
    run_linetypes <- c("solid", "dotted", "dotted")
    
    # Iterate through the selected runs
    for (i in seq_along(selected_runs)) {
      run <- selected_runs[i]
      filtered_data <- data %>% filter(Condition == paste(condition, run, sep = "_"))
      
      # Get the sharpness time and align the data
      sharpness_time <- get_sharpness_time(filtered_data)
      data_run <- filtered_data %>%
        mutate(Time_aligned = Time - sharpness_time) %>%
        mutate(Time_aligned = pmax(Time_aligned, 0)) %>%  # Set negative values to 0
        mutate(Cap_aligned = Cap)  # Retain Cap values
      
      # Assign gradient color based on the condition percentage
      gradient_color <- switch(i,
                               gradient_colors_R1[condition_percentage + 1],
                               gradient_colors_R2[condition_percentage + 1],
                               gradient_colors_R3[condition_percentage + 1]
      )
      
      condition_aligned_data[[i]] <- data_run %>%
        mutate(Run = paste0("R", i), Run_Color = gradient_color, Run_Linetype = run_linetypes[i])
    }
    
    # Combine all aligned data for this condition
    aligned_data_combined <- bind_rows(condition_aligned_data)
    
    # Filter data based on max time
    aligned_data_combined <- aligned_data_combined %>%
      filter(Time_aligned <= max_time_templabel)
    
    # Create the plot
    plot <- aligned_data_combined %>%
      ggplot(aes(x = Time_aligned, y = Cap_aligned, group = Run, color = Run_Color, linetype = Run_Linetype)) +
      geom_line(size = 1.2) +
      scale_color_identity(
        guide = guide_legend(override.aes = list(color = fixed_colors, linetype = run_linetypes)),
        labels = names(fixed_colors)
      ) +
      scale_linetype_identity() +
      labs(
        x = "Time(s)",
        y = "C(fF)",
        color = "Runs",
        linetype = "Runs"
      ) +
      theme_minimal() +
      theme(
        axis.text = element_text(size = 20, family = "Times New Roman", face = "bold"),
        axis.text.x = element_text(margin = margin(t = 10)),
        axis.title.x = element_text(size = 20, family = "Times New Roman", face = "bold", margin = margin(t = 0, r = 40, b = 0, l = 10)),
        axis.title.y = element_text(size = 20, family = "Times New Roman", face = "bold", margin = margin(t = 0, r = 40, b = 0, l = 10)),
        legend.text = element_text(size = 15, family = "Times New Roman", face = "bold"),
        legend.title = element_text(size = 15, family = "Times New Roman", face = "bold"),
        legend.position = c(0.7, 0.5), 
        legend.direction = "vertical",
        legend.spacing.y = unit(0.002, 'cm'),
        legend.key.size = unit(0.8, 'lines'),
        panel.background = element_blank(),
        plot.background = element_blank(),
        panel.grid.major = element_blank(),
        panel.grid.minor = element_blank(),
        panel.border = element_rect(color = "black", fill = NA)
      ) +
      scale_x_continuous(
        limits = c(0, 850),
        breaks = seq(0, 800, by = 400)) +
      coord_cartesian(ylim = c(130, 180)) +
      scale_y_continuous(breaks = c(130, 150, 170))
    
    plot_list[[condition]] <- plot
  }
  
  # Combine the plots into a grid
  combined_plot <- cowplot::plot_grid(
    plotlist = lapply(plot_list, function(p) {
      p 
    }),
    nrow = 11,
    ncol = 1,
    align = "v"
  )
  
  # Save the combined plot
  ggsave(
    filename = paste0("Combined_Plot_E", temp_label, ".png"), 
    plot = combined_plot, 
    height = 20, 
    width = 2.5
  )
}


data_25C <- read.xlsx("reshape_Water-Ethanol.xlsx", sheet = "25C", colNames = TRUE)
data_40C <- read.xlsx("reshape_Water-Ethanol.xlsx", sheet = "40C", colNames = TRUE)
data_50C <- read.xlsx("reshape_Water-Ethanol.xlsx", sheet = "50C", colNames = TRUE)
data_60C <- read.xlsx("reshape_Water-Ethanol.xlsx", sheet = "60C", colNames = TRUE)


df_25C <- read.xlsx("timeline_results__ethanol_water25C.xlsx", colNames = TRUE)
df_40C <- read.xlsx("timeline_results__ethanol_water40C.xlsx", colNames = TRUE)
df2_40C <- read.xlsx("mean_sd_combinations_and_selected_runs_ethanol_water40C.xlsx", sheet = "Best_Combinations", colNames = TRUE)
df2_25C <- read.xlsx("mean_sd_combinations_and_selected_runs_ethanol_water25C.xlsx", sheet = "Best_Combinations", colNames = TRUE)
df_50C <- read.xlsx("timeline_results__ethanol_water50C.xlsx", colNames = TRUE)
df2_50C <- read.xlsx("mean_sd_combinations_and_selected_runs_ethanol_water50C.xlsx", sheet = "Best_Combinations", colNames = TRUE)

df_60C <- read.xlsx("timeline_results__ethanol_water60C.xlsx", colNames = TRUE)
df2_60C <- read.xlsx("mean_sd_combinations_and_selected_runs_ethanol_water60C.xlsx", sheet = "Best_Combinations", colNames = TRUE)


# Calculate the maximum time for each temperature's selected run

max_time_25C <- calculate_max_time_for_run(data_25C, df2_25C)
max_time_40C <- calculate_max_time_for_run(data_40C, df2_40C)
max_time_50C <- calculate_max_time_for_run(data_50C, df2_50C)
max_time_60C <- calculate_max_time_for_run(data_60C, df2_60C)


# Apply the process_data function to each temperature
process_selected_data(data_25C, "25C", df_25C, df2_25C, max_time_25C)
process_selected_data(data_40C, "40C", df_40C, df2_40C, max_time_40C)
process_selected_data(data_50C, "50C", df_50C, df2_50C, max_time_50C)
process_selected_data(data_60C, "60C", df_60C, df2_60C, max_time_60C)

############selected curves#############
############selected curves#############
# Function to calculate the maximum cumulative time for the selected run of a temperature
calculate_max_time_for_run <- function(data, df2) {
  # Extract the first selected run from df2
  selected_run <- str_split(df2$combination[1], ", ")[[1]][1]
  
  # Process the data to calculate cumulative time for the selected run
  selected_data <- data %>%
    mutate(Condition = str_replace(Condition, "-run", "_run")) %>%
    filter(Condition == paste0(df2$condition[1], "_", selected_run)) %>%
    group_by(Condition) %>%
    mutate(Cumulative_Time = cumsum(Time)) %>%
    ungroup()
  
  # Return the maximum cumulative time for the selected run
  return(max(selected_data$Cumulative_Time))
}

# Calculate the maximum time for each temperature's selected run
max_time_25C <- calculate_max_time_for_run(data_25C, df2_25C)
max_time_40C <- calculate_max_time_for_run(data_40C, df2_40C)
max_time_50C <- calculate_max_time_for_run(data_50C, df2_50C)
max_time_60C <- calculate_max_time_for_run(data_60C, df2_60C)

# Function to process and visualize data for each temperature using the specific max time
process_selected_data <- function(data, temp_label, df, df2, max_time_templabel) {
  
  data <- data %>%
    group_by(Condition) %>%
    mutate(Cumulative_Time = cumsum(Time)) %>%
    ungroup() %>%
    mutate(Time = Cumulative_Time) %>%
    select(-Cumulative_Time) %>%
    mutate(Condition = str_replace(Condition, "-run", "_run"))
  
  # Ensure 'df' is structured properly
  df$condition <- factor(df$condition)
  
  # Preparing to store selected data
  selected_data <- data.frame()
  
  # Create ethanol labels for each condition depending on the temperature
  if (temp_label == "25C") {
    ethanol_labels <- c(
      "Pure Water_run10" = "0",
      "10% Ethanol_run5" = "10",
      "20% Ethanol_run4" = "20",
      "30% Ethanol_run5" = "30",
      "40% Ethanol_run5" = "40",
      "50% Ethanol_run1" = "50",
      "60% Ethanol_run2" = "60",
      "70% Ethanol_run2" = "70",
      "80% Ethanol_run1" = "80",
      "90% Ethanol_run3" = "90",
      "Pure Ethanol_run3" = "100"
    )
  } else {
    ethanol_labels <- c(
      "Pure Water_run1" = "0",
      "10% Ethanol_run1" = "10",
      "20% Ethanol_run1" = "20",
      "30% Ethanol_run1" = "30",
      "40% Ethanol_run1" = "40",
      "50% Ethanol_run1" = "50",
      "60% Ethanol_run1" = "60",
      "70% Ethanol_run1" = "70",
      "80% Ethanol_run1" = "80",
      "90% Ethanol_run1" = "90",
      "Pure Ethanol_run1" = "100"
    )
  }
  
  # Process each condition from df2
  unique_conditions <- unique(df2$condition)
  
  for (condition in unique_conditions) {
    df2_subset <- df2 %>% filter(condition == !!condition)
    selected_runs <- unlist(strsplit(as.character(df2_subset$combination[1]), ", "))
    run <- selected_runs[1]  # Ensure 'run' exists in our data columns
    
    filtered_data <- data %>%
      filter(Condition == paste(condition, run, sep = "_"))
    
    # Get the sharpness time for aligning data
    sharpness_time <- get_sharpness_time(filtered_data)
    
    data_run <- filtered_data %>%
      mutate(Time_aligned = pmax(Time - sharpness_time, 0)) %>%
      mutate(Cap_aligned = Cap)
    
    selected_data <- bind_rows(selected_data, data_run %>% select(Time_aligned, Cap_aligned, Condition))
  }
  
  # Determine a common starting value, e.g., the median of the first capacitance values of each condition
  common_start_value <- median(selected_data$Cap_aligned[selected_data$Time_aligned == 0])
  
  # Function to adjust the capacitance values so that all start from the common start value
  adjust_capacitance <- function(data, common_value) {
    data %>%
      group_by(Condition) %>%
      mutate(adjustment = common_value - first(Cap_aligned)) %>%
      ungroup() %>%
      mutate(Cap_aligned = Cap_aligned + adjustment)
  }
  
  # Apply the adjustment function
  adjusted_data <- adjust_capacitance(selected_data, common_start_value)
  
  # Truncate data at the max time for the current temperature
  adjusted_data <- adjusted_data %>%
    filter(Time_aligned <= max_time_templabel)
  
  # Update the Condition column to reflect ethanol percentages
  adjusted_data <- adjusted_data %>%
    mutate(Condition = ethanol_labels[Condition])
  
  adjusted_data$Condition <- factor(adjusted_data$Condition, levels = c("0", "10", "20", "30", "40", "50", "60", "70", "80", "90", "100"))
  # Generate gradient colors from light blue to navy blue for 11 conditions (0% to 100%)
  color_gradient <- colorRampPalette(c("#6CA6CD", "navyblue"))(11)
  # Plot the adjusted data with custom color and legend settings
  selected_plot <- ggplot(adjusted_data, aes(x = Time_aligned, y = Cap_aligned, color = Condition)) +
    geom_line(size = 1) +
    labs(
      x = "Time(s)",
      y = "C(fF)",
      color = paste("E_W(%),", temp_label)) +  # Adding temp_label to the legend title
    theme_minimal() +
    theme(
      axis.text = element_text(size = 20, family = "Times New Roman", face = "bold"),
      axis.text.x = element_text(margin = margin(t = 10)),  # Add margin to x-axis text
      axis.title.x = element_text(size = 20, family = "Times New Roman", face = "bold", margin = margin(t = 0, r = 10, b = 0, l = 5)),
      axis.title.y = element_text(size = 20, family = "Times New Roman", face = "bold",  margin = margin(t = 0, r = 10, b = 0, l = 5)),
      legend.text = element_text(size = 12, family = "Times New Roman", face = "bold"),
      legend.title = element_text(size = 12, family = "Times New Roman", face = "bold"),
      legend.position = "none", 
      legend.direction = "vertical",
      legend.spacing.y = unit(0.002, 'cm'),
      legend.key.size = unit(0.8, 'lines'),
      panel.background = element_blank(),
      plot.background = element_blank(),
      panel.grid.major = element_blank(),
      panel.grid.minor = element_blank(),
      panel.border = element_rect(color = "black", fill = NA)
    ) +
    # Apply scale_color_manual with dynamically generated gradient colors
    scale_color_manual(
      values = color_gradient
    )+
    scale_x_continuous(
      limits = c(0, 850),  # Slightly extend the limit
      breaks = seq(0, 800, by = 400)) +
    
    coord_cartesian(ylim = c(130, 180)) +
    scale_y_continuous(breaks = c(130, 150, 170))
  
  # Save the plot
  ggsave(filename = paste0("Selected_Curves_E", temp_label, ".png"), plot = selected_plot, height = 2, width = 2.5)
}

# Apply the process_selected_data function to the data of each temperature using its specific max time
process_selected_data(data_25C, "25C", df_25C, df2_25C, max_time_25C)
process_selected_data(data_40C, "40C", df_40C, df2_40C, max_time_40C)
process_selected_data(data_50C, "50C", df_50C, df2_50C, max_time_50C)
process_selected_data(data_60C, "60C", df_60C, df2_60C, max_time_60C)

############

combine_and_align <- function(upper_image_path, lower_image_path, output_path) {
  # Load images
  upper_image <- image_read(upper_image_path)
  lower_image <- image_read(lower_image_path)
  
  # Trim the images to calculate the content width
  trimmed_upper_image <- image_trim(upper_image)
  trimmed_lower_image <- image_trim(lower_image)
  
  # Get the width of the trimmed images
  trimmed_upper_width <- image_info(trimmed_upper_image)$width
  trimmed_lower_width <- image_info(trimmed_lower_image)$width
  
  # Calculate the left padding (difference between original width and trimmed width)
  left_padding_upper <- image_info(upper_image)$width - trimmed_upper_width
  left_padding_lower <- image_info(lower_image)$width - trimmed_lower_width
  
  # Determine the maximum width for alignment
  aligned_width <- max(image_info(upper_image)$width, image_info(lower_image)$width)
  
  # Adjust both images with consistent left padding and total width
  aligned_upper_image <- image_extent(upper_image, paste0(aligned_width, "x"), gravity = "west")
  aligned_lower_image <- image_extent(lower_image, paste0(aligned_width, "x"), gravity = "west")
  
  # Stack images vertically
  combined_image <- image_append(c(aligned_upper_image, aligned_lower_image), stack = TRUE)
  
  # Save the final aligned image
  image_write(combined_image, path = output_path)
}

# Use the function with your specific files
combine_and_align("Combined_Plot_E25C.png", "Selected_Curves_E25C.png", "Combined_Plot_E25C.png")
combine_and_align("Combined_Plot_E40C.png", "Selected_Curves_E40C.png", "Combined_Plot_E40C.png")
combine_and_align("Combined_Plot_E50C.png", "Selected_Curves_E50C.png", "Combined_Plot_E50C.png")
combine_and_align("Combined_Plot_E60C.png", "Selected_Curves_E60C.png", "Combined_Plot_E60C.png")


#__________________________
# Methanol
#_________________________
rm(list=ls())

#_______________________
# Libraries
#_______________________
library(nortest)
library(writexl)
library(tidyverse)
library(openxlsx)
library(dplyr)
library(Matrix)
library(lme4)
library(ggplot2)
library(car)
library(extrafont)
library(stringr)
library(gridExtra)
library(combinat)
library(tidyr)
library(cowplot)
library(ggpubr)
library(rlang)
library(broom)
library(purrr)
library(magick)
library(ggpmisc)  # For adding equation and R² to plot
# Import fonts
# font_import(prompt = FALSE)
loadfonts(device = "win")

get_sharpness_time <- function(data) {
  # Loop through the data until the cap value increases significantly
  for (i in 2:nrow(data)) {
    # Check if there's a notable increase in the Cap value after a steady range
    if (data$Cap[i] > 160) {
      return(data$Time[i])
    }
  }
  
  # Return NA if no sharpness time is found
  return(NA)
}

calculate_max_time_for_run <- function(data, df2) {
  # Extract the first selected run from df2
  selected_run <- str_split(df2$combination[1], ", ")[[1]][1]
  
  # Process the data to calculate cumulative time for the selected run
  selected_data <- data %>%
    mutate(Condition = str_replace(Condition, "-run", "_run")) %>%
    filter(Condition == paste0(df2$condition[1], "_", selected_run)) %>%
    group_by(Condition) %>%
    mutate(Cumulative_Time = cumsum(Time)) %>%
    ungroup()
  
  # Return the maximum cumulative time for the selected run
  return(max(selected_data$Cumulative_Time))
}

process_selected_data <- function(data, temp_label, df, df2, max_time_templabel) {
  
  # Group and calculate cumulative time
  data <- data %>%
    group_by(Condition) %>%
    mutate(Cumulative_Time = cumsum(Time)) %>%
    ungroup() %>%
    mutate(Time = Cumulative_Time) %>%
    select(-Cumulative_Time)
  
  # Replace "-run" with "_run" in the Condition column to ensure uniformity
  data <- data %>%
    mutate(Condition = str_replace(Condition, "-run", "_run"))
  
  # Extract Run_Num as a numeric value from Condition
  data <- data %>%
    mutate(Run_Num = as.numeric(str_extract(Condition, "\\d+$")))
  
  # Ensure df2 and df have consistent column names
  df$condition <- factor(df$condition)
  
  # Initialize a list to store plots
  plot_list <- list()
  
  # Define condition mapping for Pure Water (0) and Pure Methanol (100)
  condition_map <- c("Pure Water" = 0, "Pure Methanol" = 100)
  for (i in 1:99) {
    condition_map[paste0(i, "% Methanol")] <- i
  }
  
  # Define gradient colors for R1, R2, R3
  gradient_colors_R1 <- colorRampPalette(c("#D2B48C", "#8B4513"))(101)  # Light blue to navy blue
  gradient_colors_R2 <- colorRampPalette(c("#E396A5", "#8B0000"))(101)  # Light red to dark red
  gradient_colors_R3 <- colorRampPalette(c("#78D978", "#006400"))(101)  # Light green to dark green
  
  # Define fixed colors for R1, R2, and R3 legend (e.g., midpoints or other meaningful colors)
  fixed_colors <- c(
    "R1" = gradient_colors_R1[51],  # Midpoint of R1 gradient (50% Methanol)
    "R2" = gradient_colors_R2[51],  # Midpoint of R2 gradient (50% Methanol)
    "R3" = gradient_colors_R3[51]   # Midpoint of R3 gradient (50% Methanol)
  )
  
  # Loop over each condition to align data and create plots
  for (condition in unique(df2$condition)) {
    # Get the corresponding rows for the current condition from df2
    df2_subset <- df2 %>% filter(condition == !!condition)
    
    # Initialize a list to store the aligned data for each run
    condition_aligned_data <- list()
    selected_runs <- unlist(strsplit(as.character(df2_subset$combination[1]), ", "))
    
    # Get the condition percentage from the mapping
    condition_percentage <- condition_map[condition]
    
    # Assign colors and linetypes based on the run order
    run_linetypes <- c("solid", "dotted", "dotted")
    
    # Iterate through the selected runs
    for (i in seq_along(selected_runs)) {
      run <- selected_runs[i]
      filtered_data <- data %>% filter(Condition == paste(condition, run, sep = "_"))
      
      # Get the sharpness time and align the data
      sharpness_time <- get_sharpness_time(filtered_data)
      data_run <- filtered_data %>%
        mutate(Time_aligned = Time - sharpness_time) %>%
        mutate(Time_aligned = pmax(Time_aligned, 0)) %>%  # Set negative values to 0
        mutate(Cap_aligned = Cap)  # Retain Cap values
      
      # Assign gradient color based on the condition percentage
      gradient_color <- switch(i,
                               gradient_colors_R1[condition_percentage + 1],
                               gradient_colors_R2[condition_percentage + 1],
                               gradient_colors_R3[condition_percentage + 1]
      )
      
      condition_aligned_data[[i]] <- data_run %>%
        mutate(Run = paste0("R", i), Run_Color = gradient_color, Run_Linetype = run_linetypes[i])
    }
    
    # Combine all aligned data for this condition
    aligned_data_combined <- bind_rows(condition_aligned_data)
    
    # Filter data based on max time
    aligned_data_combined <- aligned_data_combined %>%
      filter(Time_aligned <= max_time_templabel)
    
    # Create the plot
    plot <- aligned_data_combined %>%
      ggplot(aes(x = Time_aligned, y = Cap_aligned, group = Run, color = Run_Color, linetype = Run_Linetype)) +
      geom_line(size = 1.2) +
      scale_color_identity(
        guide = guide_legend(override.aes = list(color = fixed_colors, linetype = run_linetypes)),
        labels = names(fixed_colors)
      ) +
      scale_linetype_identity() +
      labs(
        x = "Time(s)",
        y = "C(fF)",
        color = "Runs",
        linetype = "Runs"
      ) +
      theme_minimal() +
      theme(
        axis.text = element_text(size = 20, family = "Times New Roman", face = "bold"),
        axis.text.x = element_text(margin = margin(t = 10)),
        axis.title.x = element_text(size = 20, family = "Times New Roman", face = "bold", margin = margin(t = 0, r = 40, b = 0, l = 10)),
        axis.title.y = element_text(size = 20, family = "Times New Roman", face = "bold", margin = margin(t = 0, r = 40, b = 0, l = 10)),
        legend.text = element_text(size = 15, family = "Times New Roman", face = "bold"),
        legend.title = element_text(size = 15, family = "Times New Roman", face = "bold"),
        legend.position = c(0.7, 0.5), 
        legend.direction = "vertical",
        legend.spacing.y = unit(0.002, 'cm'),
        legend.key.size = unit(0.8, 'lines'),
        panel.background = element_blank(),
        plot.background = element_blank(),
        panel.grid.major = element_blank(),
        panel.grid.minor = element_blank(),
        panel.border = element_rect(color = "black", fill = NA)
      ) +
      scale_x_continuous(
        limits = c(0, 850),
        breaks = seq(0, 800, by = 400)) +
      coord_cartesian(ylim = c(130, 180)) +
      scale_y_continuous(breaks = c(130, 150, 170))
    
    plot_list[[condition]] <- plot
  }
  
  # Combine the plots into a grid
  combined_plot <- cowplot::plot_grid(
    plotlist = lapply(plot_list, function(p) {
      p 
    }),
    nrow = 11,
    ncol = 1,
    align = "v"
  )
  
  # Save the combined plot
  ggsave(
    filename = paste0("Combined_Plot_M", temp_label, ".png"), 
    plot = combined_plot, 
    height = 20, 
    width = 2.5
  )
}


data_25C <- read.xlsx("reshape_Water-Methanol.xlsx", sheet = "25C", colNames = TRUE)
data_40C <- read.xlsx("reshape_Water-Methanol.xlsx", sheet = "40C", colNames = TRUE)
data_50C <- read.xlsx("reshape_Water-Methanol.xlsx", sheet = "50C", colNames = TRUE)
data_60C <- read.xlsx("reshape_Water-Methanol.xlsx", sheet = "60C", colNames = TRUE)


df_25C <- read.xlsx("timeline_results__Methanol_water25C.xlsx", colNames = TRUE)
df_40C <- read.xlsx("timeline_results__Methanol_water40C.xlsx", colNames = TRUE)
df2_40C <- read.xlsx("mean_sd_combinations_and_selected_runs_Methanol_water40C.xlsx", sheet = "Best_Combinations", colNames = TRUE)
df2_25C <- read.xlsx("mean_sd_combinations_and_selected_runs_Methanol_water25C.xlsx", sheet = "Best_Combinations", colNames = TRUE)
df_50C <- read.xlsx("timeline_results__Methanol_water50C.xlsx", colNames = TRUE)
df2_50C <- read.xlsx("mean_sd_combinations_and_selected_runs_Methanol_water50C.xlsx", sheet = "Best_Combinations", colNames = TRUE)

df_60C <- read.xlsx("timeline_results__Methanol_water60C.xlsx", colNames = TRUE)
df2_60C <- read.xlsx("mean_sd_combinations_and_selected_runs_Methanol_water60C.xlsx", sheet = "Best_Combinations", colNames = TRUE)


# Calculate the maximum time for each temperature's selected run

max_time_25C <- calculate_max_time_for_run(data_25C, df2_25C)
max_time_40C <- calculate_max_time_for_run(data_40C, df2_40C)
max_time_50C <- calculate_max_time_for_run(data_50C, df2_50C)
max_time_60C <- calculate_max_time_for_run(data_60C, df2_60C)


# Apply the process_data function to each temperature
process_selected_data(data_25C, "25C", df_25C, df2_25C, max_time_25C)
process_selected_data(data_40C, "40C", df_40C, df2_40C, max_time_40C)
process_selected_data(data_50C, "50C", df_50C, df2_50C, max_time_50C)
process_selected_data(data_60C, "60C", df_60C, df2_60C, max_time_60C)


############selected curves#############
# Function to calculate the maximum cumulative time for the selected run of a temperature
calculate_max_time_for_run <- function(data, df2) {
  # Extract the first selected run from df2
  selected_run <- str_split(df2$combination[1], ", ")[[1]][1]
  
  # Process the data to calculate cumulative time for the selected run
  selected_data <- data %>%
    mutate(Condition = str_replace(Condition, "-run", "_run")) %>%
    filter(Condition == paste0(df2$condition[1], "_", selected_run)) %>%
    group_by(Condition) %>%
    mutate(Cumulative_Time = cumsum(Time)) %>%
    ungroup()
  
  # Return the maximum cumulative time for the selected run
  return(max(selected_data$Cumulative_Time))
}

# Calculate the maximum time for each temperature's selected run
max_time_25C <- calculate_max_time_for_run(data_25C, df2_25C)
max_time_40C <- calculate_max_time_for_run(data_40C, df2_40C)
max_time_50C <- calculate_max_time_for_run(data_50C, df2_50C)
max_time_60C <- calculate_max_time_for_run(data_60C, df2_60C)

# Function to process and visualize data for each temperature using the specific max time
process_selected_data <- function(data, temp_label, df, df2, max_time_templabel) {
  
  data <- data %>%
    group_by(Condition) %>%
    mutate(Cumulative_Time = cumsum(Time)) %>%
    ungroup() %>%
    mutate(Time = Cumulative_Time) %>%
    select(-Cumulative_Time) %>%
    mutate(Condition = str_replace(Condition, "-run", "_run"))
  
  # Ensure 'df' is structured properly
  df$condition <- factor(df$condition)
  
  # Preparing to store selected data
  selected_data <- data.frame()
  
  if (temp_label == "25C") {
    Methanol_labels <- c(
      "Pure Water_run7" = "0",
      "10% Methanol_run3" = "10",
      "20% Methanol_run2" = "20",
      "30% Methanol_run1" = "30",
      "40% Methanol_run2" = "40",
      "50% Methanol_run1" = "50",
      "60% Methanol_run3" = "60",
      "70% Methanol_run4" = "70",
      "80% Methanol_run4" = "80",
      "90% Methanol_run1" = "90",
      "Pure Methanol_run1" = "100"
    )
  } else if (temp_label == "40C") {
    Methanol_labels <- c(
      "Pure Water_run2" = "0",
      "10% Methanol_run1" = "10",
      "20% Methanol_run1" = "20",
      "30% Methanol_run1" = "30",
      "40% Methanol_run1" = "40",
      "50% Methanol_run1" = "50",
      "60% Methanol_run1" = "60",
      "70% Methanol_run1" = "70",
      "80% Methanol_run1" = "80",
      "90% Methanol_run1" = "90",
      "Pure Methanol_run1" = "100"
    )
  } else if (temp_label == "60C") {
    Methanol_labels <- c(
      "Pure Water_run4" = "0",
      "10% Methanol_run1" = "10",
      "20% Methanol_run1" = "20",
      "30% Methanol_run1" = "30",
      "40% Methanol_run1" = "40",
      "50% Methanol_run1" = "50",
      "60% Methanol_run1" = "60",
      "70% Methanol_run1" = "70",
      "80% Methanol_run1" = "80",
      "90% Methanol_run1" = "90",
      "Pure Methanol_run1" = "100"
    )
  } else {
    Methanol_labels <- c(
      "Pure Water_run1" = "0",
      "10% Methanol_run1" = "10",
      "20% Methanol_run1" = "20",
      "30% Methanol_run1" = "30",
      "40% Methanol_run1" = "40",
      "50% Methanol_run1" = "50",
      "60% Methanol_run1" = "60",
      "70% Methanol_run1" = "70",
      "80% Methanol_run1" = "80",
      "90% Methanol_run1" = "90",
      "Pure Methanol_run1" = "100"
    )
  }
  
  # Process each condition from df2
  unique_conditions <- unique(df2$condition)
  
  for (condition in unique_conditions) {
    df2_subset <- df2 %>% filter(condition == !!condition)
    selected_runs <- unlist(strsplit(as.character(df2_subset$combination[1]), ", "))
    run <- selected_runs[1]  # Ensure 'run' exists in our data columns
    
    filtered_data <- data %>%
      filter(Condition == paste(condition, run, sep = "_"))
    
    filtered_data <- filtered_data %>%
      filter(!is.na(Cap))
    
    # Get the sharpness time for aligning data
    sharpness_time <- get_sharpness_time(filtered_data)
    
    data_run <- filtered_data %>%
      mutate(Time_aligned = pmax(Time - sharpness_time, 0)) %>%
      mutate(Cap_aligned = Cap)
    
    selected_data <- bind_rows(selected_data, data_run %>% select(Time_aligned, Cap_aligned, Condition))
  }
  
  # Determine a common starting value, e.g., the median of the first capacitance values of each condition
  common_start_value <- median(selected_data$Cap_aligned[selected_data$Time_aligned == 0])
  
  # Function to adjust the capacitance values so that all start from the common start value
  adjust_capacitance <- function(data, common_value) {
    data %>%
      group_by(Condition) %>%
      mutate(adjustment = common_value - first(Cap_aligned)) %>%
      ungroup() %>%
      mutate(Cap_aligned = Cap_aligned + adjustment)
  }
  
  # Apply the adjustment function
  adjusted_data <- adjust_capacitance(selected_data, common_start_value)
  
  # Truncate data at the max time for the current temperature
  adjusted_data <- adjusted_data %>%
    filter(Time_aligned <= max_time_templabel)
  
  # Update the Condition column to reflect ethanol percentages
  adjusted_data <- adjusted_data %>%
    mutate(Condition = Methanol_labels[Condition])
  
  adjusted_data$Condition <- factor(adjusted_data$Condition, levels = c("0", "10", "20", "30", "40", "50", "60", "70", "80", "90", "100"))
  # Generate gradient colors from light blue to navy blue for 11 conditions (0% to 100%)
  color_gradient <- colorRampPalette(c("#D2B48C", "#8B4513"))(11)
  # Plot the adjusted data with custom color and legend settings
  selected_plot <- ggplot(adjusted_data, aes(x = Time_aligned, y = Cap_aligned, color = Condition)) +
    geom_line(size = 1) +
    labs(
      x = "Time(s)",
      y = "C(fF)",
      color = paste("M_W(%),", temp_label)) +  # Adding temp_label to the legend title
    theme_minimal() +
    theme(
      axis.text = element_text(size = 20, family = "Times New Roman", face = "bold"),
      axis.text.x = element_text(margin = margin(t = 10)),  # Add margin to x-axis text
      axis.title.x = element_text(size = 20, family = "Times New Roman", face = "bold", margin = margin(t = 0, r = 10, b = 0, l = 5)),
      axis.title.y = element_text(size = 20, family = "Times New Roman", face = "bold",  margin = margin(t = 0, r = 10, b = 0, l = 5)),
      legend.text = element_text(size = 12, family = "Times New Roman", face = "bold"),
      legend.title = element_text(size = 12, family = "Times New Roman", face = "bold"),
      legend.position = "none", 
      legend.direction = "vertical",
      legend.spacing.y = unit(0.002, 'cm'),
      legend.key.size = unit(0.8, 'lines'),
      panel.background = element_blank(),
      plot.background = element_blank(),
      panel.grid.major = element_blank(),
      panel.grid.minor = element_blank(),
      panel.border = element_rect(color = "black", fill = NA)
    ) +
    # Apply scale_color_manual with dynamically generated gradient colors
    scale_color_manual(
      values = color_gradient
    )+
    scale_x_continuous(
      limits = c(0, 850),  # Slightly extend the limit
      breaks = seq(0, 800, by = 400)) +
    
    coord_cartesian(
      ylim = if (temp_label == "60C") c(130, 190) else c(130, 180)
    ) +
    scale_y_continuous(
      breaks = if (temp_label == "60C") c(130, 150, 180) else c(130, 150, 170)
    )
  
  # Save the plot
  ggsave(filename = paste0("Selected_Curves_M", temp_label, ".png"), plot = selected_plot, height = 2, width = 2.5)
}

# Apply the process_selected_data function to the data of each temperature using its specific max time
process_selected_data(data_25C, "25C", df_25C, df2_25C, max_time_25C)
process_selected_data(data_40C, "40C", df_40C, df2_40C, max_time_40C)
process_selected_data(data_50C, "50C", df_50C, df2_50C, max_time_50C)
process_selected_data(data_60C, "60C", df_60C, df2_60C, max_time_60C)

############
combine_and_align <- function(upper_image_path, lower_image_path, output_path) {
  # Load images
  upper_image <- image_read(upper_image_path)
  lower_image <- image_read(lower_image_path)
  
  # Trim the images to calculate the content width
  trimmed_upper_image <- image_trim(upper_image)
  trimmed_lower_image <- image_trim(lower_image)
  
  # Get the width of the trimmed images
  trimmed_upper_width <- image_info(trimmed_upper_image)$width
  trimmed_lower_width <- image_info(trimmed_lower_image)$width
  
  # Calculate the left padding (difference between original width and trimmed width)
  left_padding_upper <- image_info(upper_image)$width - trimmed_upper_width
  left_padding_lower <- image_info(lower_image)$width - trimmed_lower_width
  
  # Determine the maximum width for alignment
  aligned_width <- max(image_info(upper_image)$width, image_info(lower_image)$width)
  
  # Adjust both images with consistent left padding and total width
  aligned_upper_image <- image_extent(upper_image, paste0(aligned_width, "x"), gravity = "west")
  aligned_lower_image <- image_extent(lower_image, paste0(aligned_width, "x"), gravity = "west")
  
  # Stack images vertically
  combined_image <- image_append(c(aligned_upper_image, aligned_lower_image), stack = TRUE)
  
  # Save the final aligned image
  image_write(combined_image, path = output_path)
}

# Use the function with your specific files
combine_and_align("Combined_Plot_M25C.png", "Selected_Curves_M25C.png", "Combined_Plot_M25C.png")
combine_and_align("Combined_Plot_M40C.png", "Selected_Curves_M40C.png", "Combined_Plot_M40C.png")
combine_and_align("Combined_Plot_M50C.png", "Selected_Curves_M50C.png", "Combined_Plot_M50C.png")
combine_and_align("Combined_Plot_M60C.png", "Selected_Curves_M60C.png", "Combined_Plot_M60C.png")


############__________________________
# Ethanol_Methanol
#_________________________
############################################
#_________________________
rm(list=ls())

#_______________________
# Libraries
#_______________________
library(nortest)
library(writexl)
library(tidyverse)
library(openxlsx)
library(dplyr)
library(Matrix)
library(lme4)
library(ggplot2)
library(car)
library(extrafont)
library(stringr)
library(gridExtra)
library(combinat)
library(tidyr)
library(cowplot)
library(ggpubr)
library(rlang)
library(broom)
library(purrr)
library(magick)
library(ggpmisc)  # For adding equation and R² to plot
# Import fonts
# font_import(prompt = FALSE)
loadfonts(device = "win")

get_sharpness_time <- function(data) {
  # Loop through the data until the cap value increases significantly
  for (i in 2:nrow(data)) {
    # Check if there's a notable increase in the Cap value after a steady range
    if (data$Cap[i] > 160) {
      return(data$Time[i])
    }
  }
  
  # Return NA if no sharpness time is found
  return(NA)
}


data_25C <- read.xlsx("reshape_Methanol-Ethanol.xlsx", sheet = "25C", colNames = TRUE)


df_25C <- read.xlsx("timeline_results__Methanol_Ethanol25C.xlsx", colNames = TRUE)
df2_25C <- read.xlsx("mean_sd_combinations_and_selected_runs_methanol_ethanol25C.xlsx", sheet = "Best_Combinations", colNames = TRUE)


calculate_max_time_for_run <- function(data, df2) {
  # Extract the first selected run from df2
  selected_run <- str_split(df2$combination[1], ", ")[[1]][1]
  
  # Process the data to calculate cumulative time for the selected run
  selected_data <- data %>%
    mutate(Condition = str_replace(Condition, "-run", "_run")) %>%
    filter(Condition == paste0(df2$condition[1], "_", selected_run)) %>%
    group_by(Condition) %>%
    mutate(Cumulative_Time = cumsum(Time)) %>%
    ungroup()
  
  # Return the maximum cumulative time for the selected run
  return(max(selected_data$Cumulative_Time))
}

# Calculate the maximum time for each temperature's selected run

max_time_25C <- calculate_max_time_for_run(data_25C, df2_25C)


process_selected_data <- function(data, temp_label, df, df2, max_time_templabel) {
  
  # Group and calculate cumulative time
  data <- data %>%
    group_by(Condition) %>%
    mutate(Cumulative_Time = cumsum(Time)) %>%
    ungroup() %>%
    mutate(Time = Cumulative_Time) %>%
    select(-Cumulative_Time)
  
  # Replace "-run" with "_run" in the Condition column to ensure uniformity
  data <- data %>%
    mutate(Condition = str_replace(Condition, "-run", "_run"))
  
  # Extract Run_Num as a numeric value from Condition
  data <- data %>%
    mutate(Run_Num = as.numeric(str_extract(Condition, "\\d+$")))
  
  # Ensure df2 and df have consistent column names
  df$condition <- factor(df$condition)
  
  # Initialize a list to store plots
  plot_list <- list()
  
  # Define condition mapping for Pure Water (0) and Pure Methanol (100)
  condition_map <- c("Pure Methanol" = 100)
  for (i in 0:99) {
    condition_map[paste0(i, "% Methanol")] <- i
  }
  
  # Define gradient colors for R1, R2, R3
  gradient_colors_R1 <- colorRampPalette(c("#66a61e", "#00441b"))(101)  # Light blue to navy blue
  gradient_colors_R2 <- colorRampPalette(c("#E396A5", "#8B0000"))(101)  # Light red to dark red
  gradient_colors_R3 <- colorRampPalette(c("#78D978", "#006400"))(101)  # Light green to dark green
  
  # Define fixed colors for R1, R2, and R3 legend (e.g., midpoints or other meaningful colors)
  fixed_colors <- c(
    "R1" = gradient_colors_R1[51],  # Midpoint of R1 gradient (50% Methanol)
    "R2" = gradient_colors_R2[51],  # Midpoint of R2 gradient (50% Methanol)
    "R3" = gradient_colors_R3[51]   # Midpoint of R3 gradient (50% Methanol)
  )
  
  # Loop over each condition to align data and create plots
  for (condition in unique(df2$condition)) {
    # Get the corresponding rows for the current condition from df2
    df2_subset <- df2 %>% filter(condition == !!condition)
    
    # Initialize a list to store the aligned data for each run
    condition_aligned_data <- list()
    selected_runs <- unlist(strsplit(as.character(df2_subset$combination[1]), ", "))
    
    # Get the condition percentage from the mapping
    condition_percentage <- condition_map[condition]
    
    # Assign colors and linetypes based on the run order
    run_linetypes <- c("solid", "dotted", "dotted")
    
    # Iterate through the selected runs
    for (i in seq_along(selected_runs)) {
      run <- selected_runs[i]
      filtered_data <- data %>% filter(Condition == paste(condition, run, sep = "_"))
      
      # Get the sharpness time and align the data
      sharpness_time <- get_sharpness_time(filtered_data)
      data_run <- filtered_data %>%
        mutate(Time_aligned = Time - sharpness_time) %>%
        mutate(Time_aligned = pmax(Time_aligned, 0)) %>%  # Set negative values to 0
        mutate(Cap_aligned = Cap)  # Retain Cap values
      
      # Assign gradient color based on the condition percentage
      gradient_color <- switch(i,
                               gradient_colors_R1[condition_percentage + 1],
                               gradient_colors_R2[condition_percentage + 1],
                               gradient_colors_R3[condition_percentage + 1]
      )
      
      condition_aligned_data[[i]] <- data_run %>%
        mutate(Run = paste0("R", i), Run_Color = gradient_color, Run_Linetype = run_linetypes[i])
    }
    
    # Combine all aligned data for this condition
    aligned_data_combined <- bind_rows(condition_aligned_data)
    
    # Filter data based on max time
    aligned_data_combined <- aligned_data_combined %>%
      filter(Time_aligned <= max_time_templabel)
    
    # Create the plot
    plot <- aligned_data_combined %>%
      ggplot(aes(x = Time_aligned, y = Cap_aligned, group = Run, color = Run_Color, linetype = Run_Linetype)) +
      geom_line(size = 1.2) +
      scale_color_identity(
        guide = guide_legend(override.aes = list(color = fixed_colors, linetype = run_linetypes)),
        labels = names(fixed_colors)
      ) +
      scale_linetype_identity() +
      labs(
        x = "Time(s)",
        y = "C(fF)",
        color = "Runs",
        linetype = "Runs"
      ) +
      theme_minimal() +
      theme(
        axis.text = element_text(size = 20, family = "Times New Roman", face = "bold"),
        axis.text.x = element_text(margin = margin(t = 10)),
        axis.title.x = element_text(size = 20, family = "Times New Roman", face = "bold", margin = margin(t = 0, r = 40, b = 0, l = 10)),
        axis.title.y = element_text(size = 20, family = "Times New Roman", face = "bold", margin = margin(t = 0, r = 40, b = 0, l = 10)),
        legend.text = element_text(size = 15, family = "Times New Roman", face = "bold"),
        legend.title = element_text(size = 15, family = "Times New Roman", face = "bold"),
        legend.position = c(0.7, 0.5), 
        legend.direction = "vertical",
        legend.spacing.y = unit(0.002, 'cm'),
        legend.key.size = unit(0.8, 'lines'),
        panel.background = element_blank(),
        plot.background = element_blank(),
        panel.grid.major = element_blank(),
        panel.grid.minor = element_blank(),
        panel.border = element_rect(color = "black", fill = NA)
      ) +
      scale_x_continuous(
        limits = c(0, 850),
        breaks = seq(0, 800, by = 400)) +
      coord_cartesian(ylim = c(130, 180)) +
      scale_y_continuous(breaks = c(130, 150, 170))
    
    plot_list[[condition]] <- plot
  }
  
  # Combine the plots into a grid
  combined_plot <- cowplot::plot_grid(
    plotlist = lapply(plot_list, function(p) {
      p 
    }),
    nrow = 11,
    ncol = 1,
    align = "v"
  )
  
  # Save the combined plot
  ggsave(
    filename = paste0("Combined_Plot_EM", temp_label, ".png"), 
    plot = combined_plot, 
    height = 20, 
    width = 2.5
  )
}


# Apply the process_data function to each temperature
process_selected_data(data_25C, "25C", df_25C, df2_25C, max_time_25C)


####################################################

# Function to calculate the maximum cumulative time for the selected run of a temperature
calculate_max_time_for_run <- function(data, df2) {
  # Extract the first selected run from df2
  selected_run <- str_split(df2$combination[1], ", ")[[1]][1]
  
  # Process the data to calculate cumulative time for the selected run
  selected_data <- data %>%
    mutate(Condition = str_replace(Condition, "-run", "_run")) %>%
    filter(Condition == paste0(df2$condition[1], "_", selected_run)) %>%
    group_by(Condition) %>%
    mutate(Cumulative_Time = cumsum(Time)) %>%
    ungroup()
  
  # Return the maximum cumulative time for the selected run
  return(max(selected_data$Cumulative_Time))
}

# Calculate the maximum time for each temperature's selected run
max_time_25C <- calculate_max_time_for_run(data_25C, df2_25C)


# Function to process and visualize data for each temperature using the specific max time
process_selected_data <- function(data, temp_label, df, df2, max_time_templabel) {
  
  data <- data %>%
    group_by(Condition) %>%
    mutate(Cumulative_Time = cumsum(Time)) %>%
    ungroup() %>%
    mutate(Time = Cumulative_Time) %>%
    select(-Cumulative_Time) %>%
    mutate(Condition = str_replace(Condition, "-run", "_run"))
  
  # Ensure 'df' is structured properly
  df$condition <- factor(df$condition)
  
  # Preparing to store selected data
  selected_data <- data.frame()
  
  # Create ethanol labels for the legend specific to the 25°C condition
  Methanol_labels <- c(
    "0% Methanol_run5" = "0",
    "10% Methanol_run4" = "10",
    "20% Methanol_run10" = "20",
    "30% Methanol_run5" = "30",
    "40% Methanol_run4" = "40",
    "50% Methanol_run6" = "50",
    "60% Methanol_run5" = "60",
    "70% Methanol_run4" = "70",
    "80% Methanol_run2" = "80",
    "90% Methanol_run3" = "90",
    "Pure Methanol_run1" = "100"
  )
  
  # Process each condition from df2
  unique_conditions <- unique(df2$condition)
  
  for (condition in unique_conditions) {
    df2_subset <- df2 %>% filter(condition == !!condition)
    selected_runs <- unlist(strsplit(as.character(df2_subset$combination[1]), ", "))
    run <- selected_runs[1]  # Ensure 'run' exists in our data columns
    
    filtered_data <- data %>%
      filter(Condition == paste(condition, run, sep = "_"))
    
    filtered_data <- filtered_data %>%
      filter(!is.na(Cap))
    
    # Get the sharpness time for aligning data
    sharpness_time <- get_sharpness_time(filtered_data)
    
    data_run <- filtered_data %>%
      mutate(Time_aligned = pmax(Time - sharpness_time, 0)) %>%
      mutate(Cap_aligned = Cap)
    
    selected_data <- bind_rows(selected_data, data_run %>% select(Time_aligned, Cap_aligned, Condition))
  }
  
  # Determine a common starting value, e.g., the median of the first capacitance values of each condition
  common_start_value <- median(selected_data$Cap_aligned[selected_data$Time_aligned == 0])
  
  # Function to adjust the capacitance values so that all start from the common start value
  adjust_capacitance <- function(data, common_value) {
    data %>%
      group_by(Condition) %>%
      mutate(adjustment = common_value - first(Cap_aligned)) %>%
      ungroup() %>%
      mutate(Cap_aligned = Cap_aligned + adjustment)
  }
  
  # Apply the adjustment function
  adjusted_data <- adjust_capacitance(selected_data, common_start_value)
  
  # Truncate data at the max time for the current temperature
  adjusted_data <- adjusted_data %>%
    filter(Time_aligned <= max_time_templabel)
  
  # Update the Condition column to reflect ethanol percentages
  adjusted_data <- adjusted_data %>%
    mutate(Condition = Methanol_labels[Condition])
  
  adjusted_data$Condition <- factor(adjusted_data$Condition, levels = c("0", "10", "20", "30", "40", "50", "60", "70", "80", "90", "100"))
  # Generate gradient colors from light blue to navy blue for 11 conditions (0% to 100%)
  color_gradient <- colorRampPalette(c("#66a61e", "#00441b"))(11)
  # Plot the adjusted data with custom color and legend settings
  selected_plot <- ggplot(adjusted_data, aes(x = Time_aligned, y = Cap_aligned, color = Condition)) +
    geom_line(size = 1) +
    labs(
      x = "Time(s)",
      y = "C(fF)",
      color = paste("M_W(%),", temp_label)) +  # Adding temp_label to the legend title
    theme_minimal() +
    theme(
      axis.text = element_text(size = 20, family = "Times New Roman", face = "bold"),
      axis.text.x = element_text(margin = margin(t = 10)),  # Add margin to x-axis text
      axis.title.x = element_text(size = 20, family = "Times New Roman", face = "bold", margin = margin(t = 0, r = 10, b = 0, l = 5)),
      axis.title.y = element_text(size = 20, family = "Times New Roman", face = "bold",  margin = margin(t = 0, r = 10, b = 0, l = 5)),
      legend.text = element_text(size = 12, family = "Times New Roman", face = "bold"),
      legend.title = element_text(size = 12, family = "Times New Roman", face = "bold"),
      legend.position = "none", 
      legend.direction = "vertical",
      legend.spacing.y = unit(0.002, 'cm'),
      legend.key.size = unit(0.8, 'lines'),
      panel.background = element_blank(),
      plot.background = element_blank(),
      panel.grid.major = element_blank(),
      panel.grid.minor = element_blank(),
      panel.border = element_rect(color = "black", fill = NA)
    ) +
    # Apply scale_color_manual with dynamically generated gradient colors
    scale_color_manual(
      values = color_gradient
    )+
    scale_x_continuous(
      limits = c(0, 850),  # Slightly extend the limit
      breaks = seq(0, 800, by = 400)) +
    
    coord_cartesian(
      ylim = if (temp_label == "60C") c(130, 190) else c(130, 180)
    ) +
    scale_y_continuous(
      breaks = if (temp_label == "60C") c(130, 150, 180) else c(130, 150, 170)
    )
  
  # Save the plot
  ggsave(filename = paste0("Selected_Curves_EM", temp_label, ".png"), plot = selected_plot, height = 2, width = 2.5)
}

# Apply the process_selected_data function to the data of each temperature using its specific max time
process_selected_data(data_25C, "25C", df_25C, df2_25C, max_time_25C)

############
combine_and_align <- function(upper_image_path, lower_image_path, output_path) {
  # Load images
  upper_image <- image_read(upper_image_path)
  lower_image <- image_read(lower_image_path)
  
  # Trim the images to calculate the content width
  trimmed_upper_image <- image_trim(upper_image)
  trimmed_lower_image <- image_trim(lower_image)
  
  # Get the width of the trimmed images
  trimmed_upper_width <- image_info(trimmed_upper_image)$width
  trimmed_lower_width <- image_info(trimmed_lower_image)$width
  
  # Calculate the left padding (difference between original width and trimmed width)
  left_padding_upper <- image_info(upper_image)$width - trimmed_upper_width
  left_padding_lower <- image_info(lower_image)$width - trimmed_lower_width
  
  # Determine the maximum width for alignment
  aligned_width <- max(image_info(upper_image)$width, image_info(lower_image)$width)
  
  # Adjust both images with consistent left padding and total width
  aligned_upper_image <- image_extent(upper_image, paste0(aligned_width, "x"), gravity = "west")
  aligned_lower_image <- image_extent(lower_image, paste0(aligned_width, "x"), gravity = "west")
  
  # Stack images vertically
  combined_image <- image_append(c(aligned_upper_image, aligned_lower_image), stack = TRUE)
  
  # Save the final aligned image
  image_write(combined_image, path = output_path)
}

# Use the function with your specific files
combine_and_align("Combined_Plot_EM25C.png", "Selected_Curves_EM25C.png", "Combined_Plot_EM25C.png")


#__________________________
#arrange
#______________________________
library(magick)

# Define images with scaling and trimming
images <- list(
  image_scale(image_trim(image_read("Combined_Plot_E25C.png")), "2000x3000"),
  image_scale(image_trim(image_read("Combined_Plot_E40C.png")), "2000x3000"),
  image_scale(image_trim(image_read("Combined_Plot_E50C.png")), "2000x3000"),
  image_scale(image_trim(image_read("Combined_Plot_E60C.png")), "2000x3000"),
  image_scale(image_trim(image_read("Combined_Plot_M25C.png")), "2000x3000"),
  image_scale(image_trim(image_read("Combined_Plot_M40C.png")), "2000x3000"),
  image_scale(image_trim(image_read("Combined_Plot_M50C.png")), "2000x3000"),
  image_scale(image_trim(image_read("Combined_Plot_M60C.png")), "2000x3000"),
  image_scale(image_trim(image_read("Combined_Plot_EM25C.png")), "2000x3000")
)

# Define a transparent spacer (e.g., 50 pixels wide, same height as images)
spacer <- image_blank(width = 15, height = 3000, color = "none")

# Interleave images and spacers
images_with_spacers <- do.call(c, lapply(images, function(img) list(img, spacer)))

# Remove the last spacer (not needed at the end)
images_with_spacers <- images_with_spacers[-length(images_with_spacers)]

# Combine images horizontally
combined_image <- Reduce(function(x, y) image_append(c(x, y), stack = FALSE), images_with_spacers)

# Save the combined image
image_write(combined_image, "Combined_All_Plots.png")

image <- image_read("Combined_All_Plots.png")

# Define the labels
labels <- c("0%", "10%", "20%", "30%", "40%", "50%", "60%", "70%", "80%", "90%", "100%", "All")

# Create a blank label image with the same height as the combined image
label_width <- 95  # Width for the text area
label_height <- image_info(combined_image)$height
label_image <- image_blank(width = label_width, height = label_height, color = "none")

image_height <- image_info(image)$height
image_width <- image_info(image)$width

row_height <- image_height / length(labels)


# Annotate each label in the corresponding position
label_spacing <- label_height / length(labels)
for (i in seq_along(labels)) {
  label_y <- (i - 1) * row_height + row_height / 2  # Center align each label
  label_image <- image_annotate(
    label_image,
    text = labels[i],
    size = 40,  # Adjust font size
    gravity = "northwest",
    location = paste0("+10+", round(label_y - 55)),  # Adjust for fine-tuning vertical centering
    color = "black",
    font = "Times New Roman",  # Font name
    weight = 700  # Add this line for bold text (700 is equivalent to bold weight)
  )
}


# Combine the label image and the combined image
final_image <- image_append(c(label_image, combined_image), stack = FALSE)

# Save the final image
image_write(final_image, "Combined_All_Plots_Labeled.png")


  knitr::include_graphics("Combined_All_Plots_Labeled.png")
```

## 1.2 Figure4

```
#undrastand best selected run

library(openxlsx)
library(stringr)
library(dplyr)

files <- list.files(pattern = "^mean_sd_combinations_and_selected_runs_.*\\.xlsx$")
for (f in files) {
  df2 <- read.xlsx(f, sheet = "Best_Combinations")
  if (!"condition" %in% names(df2) | !"combination" %in% names(df2)) next
  df50 <- df2 %>% filter(grepl("^50%", condition))
  if (nrow(df50) > 0) {
    cat("\n", f, "\n",
        " → Condition:", df50$condition[1],
        "\n → Selected run(s):", df50$combination[1], "\n")
  }
}
```

```
## 
##  mean_sd_combinations_and_selected_runs_25C.xlsx 
##   → Condition: 50% Methanol 
##  → Selected run(s): run4, run6, run9 
## 
##  mean_sd_combinations_and_selected_runs_40C.xlsx 
##   → Condition: 50% Methanol 
##  → Selected run(s): run1, run2, run3 
## 
##  mean_sd_combinations_and_selected_runs_50C.xlsx 
##   → Condition: 50% Methanol 
##  → Selected run(s): run1, run2, run3 
## 
##  mean_sd_combinations_and_selected_runs_60C.xlsx 
##   → Condition: 50% Methanol 
##  → Selected run(s): run1, run2, run3 
## 
##  mean_sd_combinations_and_selected_runs_ethanol_water25C.xlsx 
##   → Condition: 50% Ethanol 
##  → Selected run(s): run1, run3, run6 
## 
##  mean_sd_combinations_and_selected_runs_ethanol_water40C.xlsx 
##   → Condition: 50% Ethanol 
##  → Selected run(s): run1, run2, run3 
## 
##  mean_sd_combinations_and_selected_runs_ethanol_water50C.xlsx 
##   → Condition: 50% Ethanol 
##  → Selected run(s): run1, run2, run3 
## 
##  mean_sd_combinations_and_selected_runs_ethanol_water60C.xlsx 
##   → Condition: 50% Ethanol 
##  → Selected run(s): run1, run2, run3 
## 
##  mean_sd_combinations_and_selected_runs_methanol_ethanol25C.xlsx 
##   → Condition: 50% Methanol 
##  → Selected run(s): run6, run7, run8 
## 
##  mean_sd_combinations_and_selected_runs_Methanol_water25C.xlsx 
##   → Condition: 50% Methanol 
##  → Selected run(s): run1, run4, run5 
## 
##  mean_sd_combinations_and_selected_runs_methanol_water40C.xlsx 
##   → Condition: 50% Methanol 
##  → Selected run(s): run1, run2, run3 
## 
##  mean_sd_combinations_and_selected_runs_methanol_water50C.xlsx 
##   → Condition: 50% Methanol 
##  → Selected run(s): run1, run2, run3 
## 
##  mean_sd_combinations_and_selected_runs_methanol_water60C.xlsx 
##   → Condition: 50% Methanol 
##  → Selected run(s): run1, run2, run3 
## 
##  mean_sd_combinations_and_selected_runs_methanol_waterRoom temperature.xlsx 
##   → Condition: 50% Methanol 
##  → Selected run(s): run1, run4, run5 
## 
##  mean_sd_combinations_and_selected_runs_Room temperature.xlsx 
##   → Condition: 50% Methanol 
##  → Selected run(s): run1, run4, run5
```

```
# ============================================================
# 0️⃣ Setup
# ============================================================
rm(list = ls())

library(openxlsx)
library(tidyverse)
library(ggplot2)
library(patchwork)
library(stringr)
library(cowplot)
library(magick)

# ============================================================
# 1️⃣ Helper: sharpness time finder
# ============================================================
get_sharpness_time <- function(data) {
  for (i in 2:nrow(data)) {
    if (data$Cap[i] > 160) {
      return(data$Time[i])
    }
  }
  return(NA)
}

# ============================================================
# 2️⃣ Function to make single temperature plot for given solvent
# ============================================================
make_panel_50pct <- function(data_path, df2_path, solvent, temp_label, color1, color2) {
  
  # ---- Load data ----
  data <- read.xlsx(data_path, sheet = temp_label)
  df2  <- read.xlsx(df2_path, sheet = "Best_Combinations")
  
  # ---- Select 50% condition ----
  df2 <- df2 %>% filter(grepl("50%", condition))
  
  # ---- Extract selected runs ----
  selected_runs <- unlist(strsplit(as.character(df2$combination[1]), ", "))
  
  # ---- Clean & prepare ----
  data <- data %>%
    mutate(Condition = str_replace(Condition, "-run", "_run")) %>%
    group_by(Condition) %>%
    mutate(Cumulative_Time = cumsum(Time)) %>%
    ungroup() %>%
    mutate(Time = Cumulative_Time) %>%
    select(-Cumulative_Time)
  
  # ---- Keep only 50% condition ----
  cond_50 <- df2$condition[1]
  data_50 <- data %>% filter(grepl(cond_50, Condition))
  
  # ---- Align runs ----
  aligned <- list()
  run_linetypes <- c("solid", "dotted", "dotted")
  
  for (i in seq_along(selected_runs)) {
    run <- selected_runs[i]
    filtered_data <- data_50 %>% filter(Condition == paste(cond_50, run, sep = "_"))
    
    sharpness_time <- get_sharpness_time(filtered_data)
    dat <- filtered_data %>%
      mutate(Time_aligned = pmax(Time - sharpness_time, 0),
             Cap_aligned = Cap,
             Run = paste0("R", i),
             Run_Linetype = run_linetypes[i])
    aligned[[i]] <- dat
  }
  
  aligned <- bind_rows(aligned)
  
  # ---- Set color palette ----
  gradient_colors <- colorRampPalette(c(color1, color2))(3)
  fixed_colors <- c("R1" = gradient_colors[1],
                    "R2" = gradient_colors[2],
                    "R3" = gradient_colors[3])
  
  # ---- Define limits ----
  ylim_use <- if (solvent == "Methanol" & temp_label == "60C") c(130, 190) else c(130, 180)
  ybreaks  <- if (solvent == "Methanol" & temp_label == "60C") c(130, 150, 180) else c(130, 150, 170)
  
  # ---- Plot ----
  # ---- Plot ----
  p <- aligned %>%
    ggplot(aes(
      x = Time_aligned, y = Cap_aligned,
      group = Run,
      color = Run, linetype = Run, shape = Run
    )) +
    
    # Lines + overlaid points for better visual distinction
    geom_line(size = 1.4) +
    geom_point(size = 3.8, stroke = 1.1) +
    
    # Manual styling for each Run
    scale_color_manual(
      values = fixed_colors,
      name = "Runs",
      labels = c("R1", "R2", "R3")
    ) +
    scale_linetype_manual(
      values = c("solid", "dashed", "dashed"),
      name = "Runs",
      labels = c("R1", "R2", "R3")
    ) +
    scale_shape_manual(
      values = c(NA, NA, NA),   # NA = none, 15 = square, 16 = circle
      name = "Runs",
      labels = c("R1", "R2", "R3")
    ) +
    
    labs(
      x = "Time (s)",
      y = "C (fF)"
    ) +
    theme_minimal(base_family = "Times New Roman") +
    theme(
      axis.text = element_text(size = 22, face = "bold"),
      axis.title = element_text(size = 24, face = "bold"),
      legend.text = element_text(size = 20, face = "bold"),
      legend.title = element_text(size = 20, face = "bold"),
      legend.position = c(0.85, 0.3),      # 🔹 legend inside upper-right
      legend.background = element_rect(fill = "white", color = "black"),
      legend.key.size = unit(1.2, "lines"),
      plot.title = element_text(size = 24, face = "bold", hjust = 0.5),
      panel.border = element_rect(color = "black", fill = NA, linewidth = 1.3),
      panel.grid = element_blank(),
      aspect.ratio = 1/2
    ) +
    scale_x_continuous(limits = c(0, 850), breaks = seq(0, 800, 400)) +
    coord_cartesian(ylim = ylim_use) +
    scale_y_continuous(breaks = ybreaks) +
    # ---- Title moved inside top-right corner ----
  annotate(
    "text",
    x = 0.97 * 850, 
    y = ylim_use[2] - 0.02 * diff(ylim_use),
    label = paste0(solvent, " — ", temp_label, " — 50%"),
    hjust = 1, vjust = 1,
    size = 6.5, fontface = "bold", family = "Times New Roman"
  )
  
  return(p)
}

# ============================================================
# 3️⃣ Generate all 9 plots (50% condition)
# ============================================================

plots <- list(
  make_panel_50pct("reshape_Water-Ethanol.xlsx", "mean_sd_combinations_and_selected_runs_ethanol_water25C.xlsx",
                   "Ethanol", "25C", "#6CA6CD", "#000080"),
  make_panel_50pct("reshape_Water-Ethanol.xlsx", "mean_sd_combinations_and_selected_runs_ethanol_water40C.xlsx",
                   "Ethanol", "40C", "#6CA6CD", "#000080"),
  make_panel_50pct("reshape_Water-Ethanol.xlsx", "mean_sd_combinations_and_selected_runs_ethanol_water50C.xlsx",
                   "Ethanol", "50C", "#6CA6CD", "#000080"),
  make_panel_50pct("reshape_Water-Ethanol.xlsx", "mean_sd_combinations_and_selected_runs_ethanol_water60C.xlsx",
                   "Ethanol", "60C", "#6CA6CD", "#000080"),
  make_panel_50pct("reshape_Water-Methanol.xlsx", "mean_sd_combinations_and_selected_runs_methanol_water25C.xlsx",
                   "Methanol", "25C", "#D2B48C", "#8B4513"),
  make_panel_50pct("reshape_Water-Methanol.xlsx", "mean_sd_combinations_and_selected_runs_methanol_water40C.xlsx",
                   "Methanol", "40C", "#D2B48C", "#8B4513"),
  make_panel_50pct("reshape_Water-Methanol.xlsx", "mean_sd_combinations_and_selected_runs_methanol_water50C.xlsx",
                   "Methanol", "50C", "#D2B48C", "#8B4513"),
  make_panel_50pct("reshape_Water-Methanol.xlsx", "mean_sd_combinations_and_selected_runs_methanol_water60C.xlsx",
                   "Methanol", "60C", "#D2B48C", "#8B4513"),
  make_panel_50pct("reshape_Methanol-Ethanol.xlsx", "mean_sd_combinations_and_selected_runs_methanol_ethanol25C.xlsx",
                   "Methanol–Ethanol", "25C", "#66a61e", "#00441b")
)

# ============================================================
# 4️⃣ Combine all plots (3×3 grid)
# ============================================================
final_plot <- wrap_plots(
  plots,
  ncol = 3,
  byrow = TRUE,
  guides = "keep"   # 🔹 each plot keeps its own legend
)

#ggsave(
  #"Combined_50pct_3x3_internal_legends.png",
 # final_plot,
 # width = 18, height = 15, dpi = 400
#)

knitr::include_graphics("Combined_50pct_3x3_internal_legends.png")
```

## 1.3 Figure5

```
rm(list=ls())

#_______________________
# Libraries
#_______________________
library(nortest)
library(writexl)
library(tidyverse)
library(openxlsx)
library(dplyr)
library(Matrix)
library(lme4)
library(ggplot2)
library(car)
library(extrafont)
library(stringr)
library(gridExtra)
library(combinat)
library(tidyr)
library(cowplot)
library(ggpubr)
library(rlang)
library(broom)
library(purrr)
library(ggpmisc)  # For adding equation and R² to plot

# Import fonts
# font_import(prompt = FALSE)
loadfonts(device = "win")

#_______________________
# Function Definitions
#_______________________
get_sharpness_time <- function(data) {
  # Loop through the data until the cap value increases significantly
  for (i in 2:nrow(data)) {
    # Check if there's a notable increase in the Cap value after a steady range
    if (data$Cap[i] > 160) {
      return(data$Time[i])
    }
  }
  
  # Return NA if no sharpness time is found
  return(NA)
}


# Function to extract timeline points and calculate delta times
get_timeline_points <- function(data, condition, run_name) {
  # Filter data for the specified condition and run
  # Group and calculate cumulative time
  data <- data %>%
    group_by(Condition) %>%
    mutate(Cumulative_Time = cumsum(Time)) %>%
    ungroup() %>%
    mutate(Time = Cumulative_Time) %>%
    select(-Cumulative_Time)
  
  # Replace "-run" with "_run" in the Condition column to ensure uniformity
  data <- data %>%
    mutate(Condition = str_replace(Condition, "-run", "_run"))
  
  filtered_data <- data %>% filter(Condition == paste(condition, run_name, sep = "_"))
  
  # Get the sharpness time (steepest capacitance transition)
  sharpness_time <- get_sharpness_time(filtered_data)
  
  # Filter data based on the sharpness time
  data_before_sharpness <- filtered_data %>%
    filter(Time < sharpness_time)
  
  
  # Filter data based on the sharpness time
  data_after_sharpness <- filtered_data %>%
    filter(Time >= sharpness_time)
  
  # Define timeline points based on the sharpness
  max_cap_value <- max(data_after_sharpness$Cap, na.rm = TRUE)
  lower_bound <- max_cap_value - 5
  upper_bound <- max_cap_value + 5
  
  cap_range_data <- data_after_sharpness %>%
    filter(Cap >= lower_bound & Cap <= upper_bound)
  
  first_flat_time <- cap_range_data$Time[1]
  last_flat_time <- cap_range_data$Time[nrow(cap_range_data)]
  
  data_after_last_time <- data_after_sharpness %>%
    filter(Time > last_flat_time)
  
  delta_t1 <- max(data_before_sharpness$Time) - min(data_before_sharpness$Time)
  delta_t2 <- max(cap_range_data$Time) - min(cap_range_data$Time)
  delta_t3 <- max(data_after_last_time$Time) - min(data_after_last_time$Time)
  
  TOE <- max(filtered_data$Time)-min(filtered_data$Time)
  
  return(list(
    before_flat = data_before_sharpness,
    flat = cap_range_data,
    after_flat = data_after_last_time,
    delta_t1 = delta_t1,
    delta_t2 = delta_t2,
    delta_t3 = delta_t3,
    TOE = TOE
  ))
}

# Function to calculate Mean and SD of delta_t1, delta_t3, and TOE for each combination of three runs
calculate_mean_sd_combinations <- function(data) {
  condition <- unique(data$condition)
  
  # Generate all possible combinations of three runs
  run_combinations <- combn(data$run_name, 3, simplify = FALSE)
  
  # Calculate Mean and SD of delta_t1, delta_t3, and TOE for each combination
  mean_delta_t1 <- sapply(run_combinations, function(runs) {
    selected_delta_t1 <- data %>% filter(run_name %in% runs) %>% pull(delta_t1)
    mean(selected_delta_t1)
  })
  
  sd_delta_t1 <- sapply(run_combinations, function(runs) {
    selected_delta_t1 <- data %>% filter(run_name %in% runs) %>% pull(delta_t1)
    sd(selected_delta_t1)
  })
  
  mean_delta_t2 <- sapply(run_combinations, function(runs) {
    selected_delta_t2 <- data %>% filter(run_name %in% runs) %>% pull(delta_t2)
    mean(selected_delta_t2)
  })
  
  sd_delta_t2 <- sapply(run_combinations, function(runs) {
    selected_delta_t2 <- data %>% filter(run_name %in% runs) %>% pull(delta_t2)
    sd(selected_delta_t2)
  })
  
  mean_delta_t3 <- sapply(run_combinations, function(runs) {
    selected_delta_t3 <- data %>% filter(run_name %in% runs) %>% pull(delta_t3)
    mean(selected_delta_t3)
  })
  
  sd_delta_t3 <- sapply(run_combinations, function(runs) {
    selected_delta_t3 <- data %>% filter(run_name %in% runs) %>% pull(delta_t3)
    sd(selected_delta_t3)
  })
  
  mean_toe <- sapply(run_combinations, function(runs) {
    selected_toes <- data %>% filter(run_name %in% runs) %>% pull(TOE)
    mean(selected_toes)
  })
  
  sd_toe <- sapply(run_combinations, function(runs) {
    selected_toes <- data %>% filter(run_name %in% runs) %>% pull(TOE)
    sd(selected_toes)
  })
  
  # Create a data frame with the combinations, their Mean and SD for delta_t1, delta_t3, and TOE
  mean_sd_table <- data.frame(
    condition = condition,
    combination = sapply(run_combinations, paste, collapse = ", "),
    mean_delta_t1 = mean_delta_t1,
    sd_delta_t1 = sd_delta_t1,
    mean_delta_t2 = mean_delta_t2,
    sd_delta_t2 = sd_delta_t2,
    mean_delta_t3 = mean_delta_t3,
    sd_delta_t3 = sd_delta_t3,
    mean_toe = mean_toe,
    sd_toe = sd_toe
  )
  
  # Return the Mean and SD table
  return(mean_sd_table)
}

# Function to calculate mean Cap for the selected runs
calculate_mean_cap <- function(data, condition, combination) {
  # Extract the runs from the combination column
  selected_runs <- str_split(combination, ", ") %>% unlist()
  
  # Filter the data for the specific condition and selected runs
  filtered_data <- data %>%
    filter(str_detect(Condition, paste0(condition, "_(", paste0(selected_runs, collapse = "|"), ")")))
  
  # Calculate the mean Cap
  mean_cap <- mean(filtered_data$Cap, na.rm = TRUE)
  sd_cap <- sd(filtered_data$Cap, na.rm = TRUE)
  return(list(mean_cap = mean_cap, sd_cap = sd_cap))
}

# Function to Process Data for a Given Temperature
process_data <- function(data, temperature) {
  
  # Replace "-run" with "_run" in the Condition column to ensure uniformity
  data <- data %>%
    mutate(Condition = str_replace(Condition, "-run", "_run"))
  
  unique_conditions <- unique(data$Condition)
  
  split_conditions <- data.frame(
    condition = sapply(strsplit(unique_conditions, "_"), `[`, 1),
    run_name = sapply(strsplit(unique_conditions, "_"), `[`, 2)
  )
  
  
  results <- split_conditions %>%
    mutate(timeline_points = map2(condition, run_name, ~get_timeline_points(data, .x, .y)))
  
  final_results <- results %>%
    rowwise() %>%
    mutate(
      before_flat = list(timeline_points$before_flat),
      flat = list(timeline_points$flat),
      after_flat = list(timeline_points$after_flat),
      delta_t1 = timeline_points$delta_t1,
      delta_t2 = timeline_points$delta_t2,
      delta_t3 = timeline_points$delta_t3,
      TOE = timeline_points$TOE
    ) %>%
    select(-timeline_points) %>%
    ungroup()
  
  final_results <- final_results %>%
    mutate(
      before_flat = sapply(before_flat, function(x) toString(x$Time)),
      flat = sapply(flat, function(x) toString(x$Time)),
      after_flat = sapply(after_flat, function(x) toString(x$Time))
    )
  
  # Save the final results as an Excel file
  write_xlsx(final_results, paste0("timeline_results__ethanol_water", temperature, ".xlsx"))
  mean_sd_combinations_table <- final_results %>%
    group_by(condition) %>%
    do(calculate_mean_sd_combinations(.)) %>%
    ungroup()
  
  # Find the combination with the smallest combined SD for delta_t1, delta_t2, delta_t3, and TOE
  best_combinations_table <- mean_sd_combinations_table %>%
    group_by(condition) %>%
    mutate(total_sd = sd_delta_t1 + sd_delta_t2 + sd_delta_t3 + sd_toe) %>%
    slice_min(total_sd) %>%
    ungroup()
  
  # Apply the function to calculate mean Cap for each row of best_combinations_table
  best_combinations_table <- best_combinations_table %>%
    rowwise() %>%
    mutate(
      mean_cap = calculate_mean_cap(data, as.character(condition), as.character(combination))$mean_cap,
      sd_cap = calculate_mean_cap(data, as.character(condition), as.character(combination))$sd_cap
    ) %>%
    ungroup()
  
  # Define the correct order of conditions
  condition_order <- c(
    "Pure Water", "10% Ethanol", "20% Ethanol", "30% Ethanol",
    "40% Ethanol", "50% Ethanol", "60% Ethanol", "70% Ethanol",
    "80% Ethanol", "90% Ethanol", "Pure Ethanol"
  )
  
  # Convert the condition column to a factor with the specified levels
  best_combinations_table$condition <- factor(best_combinations_table$condition, levels = condition_order)
  
  # Arrange the data frame based on the factor levels
  best_combinations_table <- best_combinations_table %>%
    arrange(condition)
  
  # Save the tables to Excel
  write_xlsx(list(
    Mean_SD_Combinations = mean_sd_combinations_table,
    Best_Combinations = best_combinations_table
  ), paste0("mean_sd_combinations_and_selected_runs_ethanol_water", temperature, ".xlsx"))
  
  return(best_combinations_table)
}
# Process for each temperature
data_25C <- read.xlsx("reshape_Water-Ethanol.xlsx", sheet = "25C", colNames = TRUE)
result_25C <- process_data(data_25C, "25C")

data_40C <- read.xlsx("reshape_Water-Ethanol.xlsx", sheet = "40C", colNames = TRUE)
result_40C <- process_data(data_40C, "40C")


data_50C <- read.xlsx("reshape_Water-Ethanol.xlsx", sheet = "50C", colNames = TRUE)
result_50C <- process_data(data_50C, "50C")


data_60C <- read.xlsx("reshape_Water-Ethanol.xlsx", sheet = "60C", colNames = TRUE)
result_60C <- process_data(data_60C, "60C")


##################boxplot###################

combined_results <- bind_rows(
  result_25C %>% mutate(temperature = "25C"),
  result_40C %>% mutate(temperature = "40C"),
  result_50C %>% mutate(temperature = "50C"),
  result_60C %>% mutate(temperature = "60C")
)

condition_order <- c(
  "Pure Water", "10% Ethanol", "20% Ethanol", "30% Ethanol",
  "40% Ethanol", "50% Ethanol", "60% Ethanol", "70% Ethanol",
  "80% Ethanol", "90% Ethanol", "Pure Ethanol"
)

# Replace 'Pure Water' with '0' and remove 'Ethanol' and '%' from other labels
clean_condition_labels <- gsub("Pure Water", "0", gsub(" Ethanol|%", "", condition_order))

# Create the plot with summarized data and customizations
combined_results <- combined_results %>%
  mutate(Condition_Num = as.numeric(factor(condition, levels = condition_order)))

# Step 1: Summarize the data to calculate the overall mean and SD for each temperature
temperature_summary <- combined_results %>%
  group_by(temperature) %>%
  summarize(
    overall_mean_toe = mean(mean_delta_t2),
    overall_sd_toe = sd(mean_delta_t2),
    .groups = 'drop'
  )

# Step 2: Create the plot with the summarized data
p <- ggplot(combined_results, aes(x = factor(temperature, levels = c("25C", "40C", "50C", "60C")), y = mean_delta_t2)) +
  geom_boxplot(outlier.shape = NA) +
  geom_errorbar(data = temperature_summary, 
                aes(x = temperature, ymin = overall_mean_toe - overall_sd_toe, ymax = overall_mean_toe + overall_sd_toe), 
                width = 0.2, linewidth = 1, color = "black", inherit.aes = FALSE) +  # Single error bar for each temperature
  geom_jitter(aes(color = factor(Condition_Num)), size = 4, width = 0.1) +
  
  # Use dark colors for each condition
  scale_color_manual(
    values = c("navyblue", "brown", "#B8860B", "darkgreen", "darkred", "#8B4513", "#4682B4", "#556B2F", "#8B008B", "#2E8B57", "#483D8B"),
    breaks = seq_along(condition_order),
    labels = clean_condition_labels,  # Updated labels with '0' for 'Pure Water' and cleaned others
    guide = guide_legend(ncol = 2)  # Make legend two columns
  ) +
  
  # Update axis labels and legend title
  labs(
    x = "Temperature",
    y = "TOE (s)",
    color = "Ethanol (%)"
  ) +
  
  # Customize the theme
  theme_minimal() +
  theme(
    axis.text= element_text(size = 18, family = "Times New Roman",face="bold"),
    axis.title = element_text(size = 18, family = "Times New Roman",face="bold"),
    legend.text = element_text(size = 18, family = "Times New Roman",face="bold"),
    legend.title = element_text(size = 18, family = "Times New Roman",face="bold"),
    legend.position = c(0.85, 0.5), 
    legend.direction = "vertical",
    panel.background = element_blank(),
    plot.background = element_blank(),
    panel.grid.major = element_blank(),
    panel.grid.minor = element_blank(),
    panel.border = element_rect(color = "black", fill = NA)
  )

# Add statistical comparisons using the Wilcoxon test
p <- p + stat_compare_means(comparisons = list(
  c("25C", "40C"), c("25C", "50C"),
  c("25C", "60C"), c("40C", "50C"),
  c("40C", "60C"), c("50C", "60C")
), 
method = "t.test",  method.args = list(var.equal = FALSE), size = 3.5, family = "Times New Roman")


ggsave("BOXPLOT_t2_E.png", plot = p, width =8, height = 6)

##############RMSE_t2###########


# Combine results for all temperatures
combined_results <- bind_rows(
  result_25C %>% mutate(temperature = "25C"),
  result_40C %>% mutate(temperature = "40C"),
  result_50C %>% mutate(temperature = "50C"),
  result_60C %>% mutate(temperature = "60C")
)

# Define condition order for the factor
condition_order <- c(
  "Pure Water", "10% Ethanol", "20% Ethanol", "30% Ethanol",
  "40% Ethanol", "50% Ethanol", "60% Ethanol", "70% Ethanol",
  "80% Ethanol", "90% Ethanol", "Pure Ethanol"
)
combined_results$condition <- factor(combined_results$condition, levels = condition_order)

# Assign to temp_data_t2
temp_data_t2 <- combined_results

# Define custom colors for each temperature
custom_colors <- c("25C" = "navyblue", "40C" = "darkgreen", "50C" = "#B8860B", "60C" = "darkred")

# Add numeric_condition for easier modeling and plotting
temp_data_t2 <- temp_data_t2 %>%
  mutate(
    numeric_condition = case_when(
      condition == "Pure Water" ~ 0,
      condition == "Pure Ethanol" ~ 100,
      TRUE ~ as.numeric(gsub("[^0-9]", "", as.character(condition)))
    )
  )

# Ensure a continuous range of numeric_condition
temp_data_t2 <- temp_data_t2 %>%
  arrange(temperature, numeric_condition) %>%
  group_by(temperature) %>%
  complete(numeric_condition = full_seq(numeric_condition, 1)) %>%
  ungroup()

# Fit a single linear model after completing missing rows
lm_model <- lm(mean_delta_t2 ~ numeric_condition * as.factor(temperature), 
               data = temp_data_t2 %>% filter(!is.na(mean_delta_t2)))

# Add linear predicted values
temp_data_t2 <- temp_data_t2 %>%
  mutate(predicted_values = predict(lm_model, newdata = temp_data_t2))

# Fit LOESS model per temperature
temp_data_t2 <- temp_data_t2 %>%
  group_by(temperature) %>%
  mutate(
    loess_fit = list(loess(mean_delta_t2 ~ numeric_condition, data = cur_data(), span = 0.75)),
    predicted_loess = predict(loess_fit[[1]], newdata = cur_data())
  ) %>%
  ungroup()

# Find min and max values for plotting
min_value_t2 <- min(temp_data_t2$mean_delta_t2, na.rm = TRUE)
max_value_t2 <- max(temp_data_t2$mean_delta_t2, na.rm = TRUE)

# Calculate accuracy metrics for linear model (using a cubic polynomial model for evaluation)
temp_models_t2 <- temp_data_t2 %>%
  filter(!is.na(mean_delta_t2)) %>%
  group_by(temperature) %>%
  nest() %>%
  mutate(
    model = map(data, ~ lm(mean_delta_t2 ~ poly(numeric_condition, 3), data = .x)),
    r_squared = map_dbl(model, ~ glance(.x)$r.squared),
    rmse = map_dbl(model, ~ sqrt(mean(residuals(.x)^2))),
    mae = map_dbl(model, ~ mean(abs(residuals(.x)))),
    rmse_percentage = map_dbl(data, ~ {
      preds <- predict(lm(mean_delta_t2 ~ poly(numeric_condition, 3), data = .x))
      range_y <- max(.x$mean_delta_t2) - min(.x$mean_delta_t2)
      rmse_val <- sqrt(mean((.x$mean_delta_t2 - preds)^2))
      (rmse_val / range_y) * 100
    })
  )

# Calculate accuracy metrics for LOESS model
temp_models_t2_loess <- temp_data_t2 %>%
  filter(!is.na(mean_delta_t2)) %>%
  group_by(temperature) %>%
  summarize(
    r_squared_loess = {
      loess_resid = mean_delta_t2 - predicted_loess
      1 - (sum(loess_resid^2) / sum((mean_delta_t2 - mean(mean_delta_t2))^2))
    },
    rmse_loess = {
      loess_resid = mean_delta_t2 - predicted_loess
      sqrt(mean(loess_resid^2))
    },
    mae_loess = {
      loess_resid = mean_delta_t2 - predicted_loess
      mean(abs(loess_resid))
    },
    rmse_percentage_loess = {
      loess_resid = mean_delta_t2 - predicted_loess
      range_y <- max(mean_delta_t2) - min(mean_delta_t2)
      (sqrt(mean(loess_resid^2)) / range_y) * 100
    }
  )

# Combine accuracy metrics
accuracy_combined <- temp_models_t2 %>%
  select(temperature, rmse_percentage) %>%
  left_join(
    temp_models_t2_loess %>% select(temperature, rmse_percentage_loess),
    by = "temperature"
  )

# Combine accuracy metrics
accuracy_combined <- temp_models_t2 %>%
  select(temperature, rmse_percentage) %>%
  left_join(
    temp_models_t2_loess %>% select(temperature, rmse_percentage_loess),
    by = "temperature"
  )

# Prepare a table with 4 rows and 2 columns for annotation
my_table <- data.frame(
  Temperature = accuracy_combined$temperature,
  `RMSE (%) (Linear)` = round(accuracy_combined$rmse_percentage, 3),
  `RMSE (%) (LOESS)` = round(accuracy_combined$rmse_percentage_loess, 3)
)

colnames(my_table) <- c("Temperature", "RMSE_Linear(%)", "RMSE_LOESS(%)")


tt <- ttheme_minimal(
  core = list(
    fg_params = list(
      fontface = c("bold", "bold", "bold", "bold"),  # Bold text for all rows
      col = c("navyblue", "darkgreen", "#B8860B", "darkred")  # Row colors
    ),
    bg_params = list(col = "black", lwd = 1)
  ),
  colhead = list(
    fg_params = list(fontface = "bold", col = "black"),  # Black title
    bg_params = list(col = "black", lwd = 1)
  )
)

# Apply the custom theme to your table
table_grob <- tableGrob(my_table, rows = NULL, theme = tt)


# Split data into segments for numeric_condition <= 80 and > 80
temp_data_t2$segment <- ifelse(temp_data_t2$numeric_condition > 80, "above_80", "below_80")

p_t2 <- ggplot(temp_data_t2, aes(x = numeric_condition, y = mean_delta_t2, color = as.factor(temperature))) +
  geom_point(size = 3, na.rm = TRUE) +  # Points for original data
  # LOESS dashed line
  geom_line(aes(y = predicted_loess, group = temperature, linetype = "LOESS"), size = 1) +
  # Linear solid line for numeric_condition <= 80
  geom_line(
    data = temp_data_t2 %>% filter(segment == "below_80"),
    aes(y = predicted_values, group = temperature, linetype = "Linear (under 80%)"),
    size = 1.8
  ) +
  # Linear dotted line for numeric_condition > 80
  geom_line(
    data = temp_data_t2 %>% filter(segment == "above_80"),
    aes(y = predicted_values, group = temperature, linetype = "Linear (80%-100%)"),
    size = 1.2
  ) +
  labs(
    x = "Ethanol (%)",
    y = expression(bold("Mean"~Delta~t[2]~"(s)")),
    linetype = "Line Type"
  ) +
  theme_minimal() +
  theme(
    axis.text.x = element_text(size = 18, family = "Times New Roman", face = "bold"),
    axis.text.y = element_text(size = 18, family = "Times New Roman", face = "bold"),
    axis.title = element_text(size = 18, family = "Times New Roman", face = "bold"),
    legend.text = element_text(size = 15, family = "Times New Roman", face = "bold"),
    legend.title = element_text(size = 15, family = "Times New Roman", face = "bold"),
    legend.key.height = unit(1.5, "lines"),
    legend.position = c(0.83, 0.55),
    panel.background = element_blank(),
    plot.background = element_blank(),
    panel.grid.major = element_blank(),
    panel.grid.minor = element_blank(),
    panel.border = element_rect(color = "black", fill = NA)
  ) +
  scale_color_manual(values = custom_colors, guide = "none") +  # Remove color legend
  scale_linetype_manual(
    values = c(
      "Linear (under 80%)" = "solid",
      "Linear (80%-100%)" = "dotted",
      "LOESS" = "dashed"
    ),
    breaks = c("Linear (under 80%)", "Linear (80%-100%)", "LOESS")  # Set the order explicitly
  ) +
  coord_cartesian(ylim = c(min_value_t2, max_value_t2)) +
  scale_x_continuous(breaks = seq(0, 100, 10), labels = as.character(seq(0, 100, 10))) +
  # Add the table annotation
  annotation_custom(
    grob = table_grob,
    xmin = 50, xmax = 90,   # Adjust as needed for positioning
    ymin = max_value_t2 - 100,
    ymax = max_value_t2 
  )

# Save the plot
ggsave("RMSE_t2_E.png", plot = p_t2, width = 8, height = 6)
##############RMSE_cap###########
# Define condition order for proper factor levels
condition_order <- c(
  "Pure Water", "10% Ethanol", "20% Ethanol", "30% Ethanol",
  "40% Ethanol", "50% Ethanol", "60% Ethanol", "70% Ethanol",
  "80% Ethanol", "90% Ethanol", "Pure Ethanol"
)

# Combine results for all temperatures
all_results <- bind_rows(
  result_25C %>% mutate(Temperature = "25C"),
  result_40C %>% mutate(Temperature = "40C"),
  result_50C %>% mutate(Temperature = "50C"),
  result_60C %>% mutate(Temperature = "60C")
)

# Convert 'condition' to factor with specified levels
all_results$condition <- factor(all_results$condition, levels = condition_order)

# Summarize data by condition and temperature
all_results_summarized <- all_results %>%
  group_by(condition, Temperature) %>%
  summarize(
    mean_cap = mean(mean_cap),
    sd_cap = mean(sd_cap),
    .groups = 'drop'
  )

# Add numeric_condition for easier modeling
all_results_summarized <- all_results_summarized %>%
  mutate(numeric_condition = as.numeric(gsub("[^0-9]", "", as.character(condition))),
         numeric_condition = ifelse(is.na(numeric_condition), 
                                    ifelse(condition == "Pure Water", 0, 100), numeric_condition))

# Fit models for mean_cap using both Linear and LOESS
temp_models_cap <- all_results_summarized %>%
  group_by(Temperature) %>%
  nest() %>%
  mutate(
    # Fit Linear model
    linear_model = map(data, ~ lm(mean_cap ~ poly(numeric_condition, 3), data = .x)),
    # Fit LOESS model
    loess_model = map(data, ~ loess(mean_cap ~ numeric_condition, data = .x, span = 0.75)),
    # Calculate predictions for Linear and LOESS
    linear_predictions = map(linear_model, ~ augment(.x)),
    loess_predictions = map(loess_model, ~ data.frame(.x$fitted)),
    # Calculate accuracy metrics
    r_squared_linear = map_dbl(linear_model, ~ glance(.x)$r.squared),
    rmse_linear = map_dbl(linear_model, ~ sqrt(mean(residuals(.x)^2))),
    rmse_loess = map_dbl(loess_model, ~ sqrt(mean((.x$residuals)^2))),
    mae_linear = map_dbl(linear_model, ~ mean(abs(residuals(.x))))
  ) %>%
  mutate(
    range_y = map_dbl(data, ~ max(.x$mean_cap) - min(.x$mean_cap)),  # Range of the observed values
    rmse_percentage_linear = (rmse_linear / range_y) * 100,
    rmse_percentage_loess = (rmse_loess / range_y) * 100
  )

# Print accuracy metrics for each temperature
temp_models_cap %>%
  select(Temperature, r_squared_linear, rmse_percentage_linear, rmse_percentage_loess)
```

```
## # A tibble: 4 × 4
## # Groups:   Temperature [4]
##   Temperature r_squared_linear rmse_percentage_linear rmse_percentage_loess
##   <chr>                  <dbl>                  <dbl>                 <dbl>
## 1 25C                    0.903                   9.56                  6.30
## 2 40C                    0.826                  12.9                  12.9 
## 3 50C                    0.885                  10.1                   7.05
## 4 60C                    0.781                  14.3                  10.8
```

```
# Find min and max values for the plot
min_value_cap <- min(all_results_summarized$mean_cap, na.rm = TRUE)
max_value_cap <- max(all_results_summarized$mean_cap, na.rm = TRUE) + 5  # Add buffer to max for annotations

# Prepare a table with RMSE for both Linear and LOESS
accuracy_combined <- temp_models_cap %>%
  select(
    Temperature, 
    `RMSE% (Linear)` = rmse_percentage_linear, 
    `RMSE% (LOESS)` = rmse_percentage_loess
  )

# Define custom theme for table
tt <- ttheme_minimal(
  core = list(
    fg_params = list(
      fontface = c("bold", "bold", "bold", "bold"),  # Bold text for all rows
      col = c("navyblue", "darkgreen", "#B8860B", "darkred")  # Row colors
    ),
    bg_params = list(col = "black", lwd = 1)
  ),
  colhead = list(
    fg_params = list(fontface = "bold", col = "black"),  # Black title
    bg_params = list(col = "black", lwd = 1)
  )
)

# Create table grob
table_grob <- tableGrob(accuracy_combined, rows = NULL, theme = tt)

# Add a separate legend for line types
p <- ggplot(all_results_summarized, aes(x = numeric_condition, y = mean_cap, group = Temperature)) +
  geom_point(aes(color = Temperature), size = 3, show.legend = FALSE) +  # Points for data, no legend
  geom_smooth(aes(linetype = "Linear", color = Temperature), method = "lm", se = FALSE, size = 1) +  # Linear model
  geom_smooth(aes(linetype = "LOESS", color = Temperature), method = "loess", se = FALSE, size = 1.8) +  # LOESS model
  labs(x = "Ethanol (%)", y = expression(bold(Mean~Delta~Cap~(fF))), linetype = "Model") +
  theme_minimal() +
  theme(
    axis.text.x = element_text(size = 18, family = "Times New Roman", face = "bold"),
    axis.text.y = element_text(size = 18, family = "Times New Roman", face = "bold"),
    axis.title = element_text(size = 18, family = "Times New Roman", face = "bold"),
    legend.text = element_text(size = 18, family = "Times New Roman", face = "bold"),
    legend.title = element_text(size = 18, family = "Times New Roman", face = "bold"),
    panel.background = element_blank(),
    plot.background = element_blank(),
    panel.grid.major = element_blank(),
    panel.grid.minor = element_blank(),
    panel.border = element_rect(color = "black", fill = NA),
    legend.position = c(0.1, 0.15)  # Adjust legend position as needed
  ) +
  coord_cartesian(ylim = c(min_value_cap, max_value_cap)) +
  scale_color_manual(values = c("25C" = "navyblue", "40C" = "darkgreen", "50C" = "#B8860B", "60C" = "darkred"), guide = "none") +
  scale_linetype_manual(
    values = c("Linear" = "solid", "LOESS" = "dashed"),
    breaks = c("Linear", "LOESS")  # Order of the legend
  ) +
  guides(
    linetype = guide_legend(override.aes = list(color = "black"))  # Black line symbols in legend
  ) +
  # Add table annotation
  annotation_custom(
    grob = table_grob,
    xmin = 55, xmax = 90,   # Adjust as needed for positioning
    ymin = max_value_cap - 7,
    ymax = max_value_cap
  )

# Save the plot
ggsave("RMSE_cap_E.png", plot = p, width = 8, height = 6)
#_______________________Metanol_______________________-
process_data <- function(data, temperature) {
  
  # Replace "-run" with "_run" in the Condition column to ensure uniformity
  data <- data %>%
    mutate(Condition = str_replace(Condition, "-run", "_run"))
  
  unique_conditions <- unique(data$Condition)
  
  split_conditions <- data.frame(
    condition = sapply(strsplit(unique_conditions, "_"), `[`, 1),
    run_name = sapply(strsplit(unique_conditions, "_"), `[`, 2)
  )
  
  
  results <- split_conditions %>%
    mutate(timeline_points = map2(condition, run_name, ~get_timeline_points(data, .x, .y)))
  
  final_results <- results %>%
    rowwise() %>%
    mutate(
      before_flat = list(timeline_points$before_flat),
      flat = list(timeline_points$flat),
      after_flat = list(timeline_points$after_flat),
      delta_t1 = timeline_points$delta_t1,
      delta_t2 = timeline_points$delta_t2,
      delta_t3 = timeline_points$delta_t3,
      TOE = timeline_points$TOE
    ) %>%
    select(-timeline_points) %>%
    ungroup()
  
  final_results <- final_results %>%
    mutate(
      before_flat = sapply(before_flat, function(x) toString(x$Time)),
      flat = sapply(flat, function(x) toString(x$Time)),
      after_flat = sapply(after_flat, function(x) toString(x$Time))
    )
  
  # Save the final results as an Excel file
  write_xlsx(final_results, paste0("timeline_results__Methanol_water", temperature, ".xlsx"))
  mean_sd_combinations_table <- final_results %>%
    group_by(condition) %>%
    do(calculate_mean_sd_combinations(.)) %>%
    ungroup()
  
  # Find the combination with the smallest combined SD for delta_t1, delta_t2, delta_t3, and TOE
  best_combinations_table <- mean_sd_combinations_table %>%
    group_by(condition) %>%
    mutate(total_sd = sd_delta_t1 + sd_delta_t2 + sd_delta_t3 + sd_toe) %>%
    slice_min(total_sd) %>%
    ungroup()
  
  # Apply the function to calculate mean Cap for each row of best_combinations_table
  best_combinations_table <- best_combinations_table %>%
    rowwise() %>%
    mutate(
      mean_cap = calculate_mean_cap(data, as.character(condition), as.character(combination))$mean_cap,
      sd_cap = calculate_mean_cap(data, as.character(condition), as.character(combination))$sd_cap
    ) %>%
    ungroup()
  
  # Define the correct order of conditions
  condition_order <- c(
    "Pure Water", "10% Methanol", "20% Methanol", "30% Methanol",
    "40% Methanol", "50% Methanol", "60% Methanol", "70% Methanol",
    "80% Methanol", "90% Methanol", "Pure Methanol"
  )
  
  # Convert the condition column to a factor with the specified levels
  best_combinations_table$condition <- factor(best_combinations_table$condition, levels = condition_order)
  
  # Arrange the data frame based on the factor levels
  best_combinations_table <- best_combinations_table %>%
    arrange(condition)
  
  # Save the tables to Excel
  write_xlsx(list(
    Mean_SD_Combinations = mean_sd_combinations_table,
    Best_Combinations = best_combinations_table
  ), paste0("mean_sd_combinations_and_selected_runs_Methanol_water", temperature, ".xlsx"))
  
  return(best_combinations_table)
}

# Process for each temperature
data_25C <- read.xlsx("reshape_Water-Methanol.xlsx", sheet = "25C", colNames = TRUE)
result_25C <- process_data(data_25C, "25C")

data_40C <- read.xlsx("reshape_Water-Methanol.xlsx", sheet = "40C", colNames = TRUE)
result_40C <- process_data(data_40C, "40C")


data_50C <- read.xlsx("reshape_Water-Methanol.xlsx", sheet = "50C", colNames = TRUE)
result_50C <- process_data(data_50C, "50C")


data_60C <- read.xlsx("reshape_Water-Methanol.xlsx", sheet = "60C", colNames = TRUE)
result_60C <- process_data(data_60C, "60C")


#############boxplot##############
combined_results <- bind_rows(
  result_25C %>% mutate(temperature = "25C"),
  result_40C %>% mutate(temperature = "40C"),
  result_50C %>% mutate(temperature = "50C"),
  result_60C %>% mutate(temperature = "60C")
)

condition_order <- c(
  "Pure Water", "10% Methanol", "20% Methanol", "30% Methanol",
  "40% Methanol", "50% Methanol", "60% Methanol", "70% Methanol",
  "80% Methanol", "90% Methanol", "Pure Methanol"
)

# Replace 'Pure Water' with '0' and remove 'Methanol' and '%' from other labels
clean_condition_labels <- gsub("Pure Water", "0", gsub(" Methanol|%", "", condition_order))

# Create the plot with summarized data and customizations
combined_results <- combined_results %>%
  mutate(Condition_Num = as.numeric(factor(condition, levels = condition_order)))

# Step 1: Summarize the data to calculate the overall mean and SD for each temperature
temperature_summary <- combined_results %>%
  group_by(temperature) %>%
  summarize(
    overall_mean_toe = mean(mean_toe),
    overall_sd_toe = sd(mean_toe),
    .groups = 'drop'
  )

# Step 2: Create the plot with the summarized data
p <- ggplot(combined_results, aes(x = factor(temperature, levels = c("25C", "40C", "50C", "60C")), y = mean_toe)) +
  geom_boxplot(outlier.shape = NA) +
  geom_errorbar(data = temperature_summary, 
                aes(x = temperature, ymin = overall_mean_toe - overall_sd_toe, ymax = overall_mean_toe + overall_sd_toe), 
                width = 0.2, linewidth = 1, color = "black", inherit.aes = FALSE) +  # Single error bar for each temperature
  geom_jitter(aes(color = factor(Condition_Num)), size = 4, width = 0.1) +
  
  # Use dark colors for each condition
  scale_color_manual(
    values = c("navyblue", "brown", "#B8860B", "darkgreen", "darkred", "#8B4513", "#4682B4", "#556B2F", "#8B008B", "#2E8B57", "#483D8B"),
    breaks = seq_along(condition_order),
    labels = clean_condition_labels,  # Updated labels with '0' for 'Pure Water' and cleaned others
    guide = guide_legend(ncol = 2)  # Make legend two columns
  ) +
  
  # Update axis labels and legend title
  labs(
    x = "Temperature",
    y = "TOE (s)",
    color = "Methanol (%)"
  ) +
  
  # Customize the theme
  theme_minimal() +
  theme(
    axis.text= element_text(size = 18, family = "Times New Roman",face="bold"),
    axis.title = element_text(size = 18, family = "Times New Roman",face="bold"),
    legend.text = element_text(size = 18, family = "Times New Roman",face="bold"),
    legend.title = element_text(size = 18, family = "Times New Roman",face="bold"),
    legend.position = c(0.85, 0.5), 
    legend.direction = "vertical",
    panel.background = element_blank(),
    plot.background = element_blank(),
    panel.grid.major = element_blank(),
    panel.grid.minor = element_blank(),
    panel.border = element_rect(color = "black", fill = NA)
  )

# Add statistical comparisons using the Wilcoxon test
p <- p + stat_compare_means(comparisons = list(
  c("25C", "40C"), c("25C", "50C"),
  c("25C", "60C"), c("40C", "50C"),
  c("40C", "60C"), c("50C", "60C")
), 
method = "t.test",  method.args = list(var.equal = FALSE), size = 3.5, family = "Times New Roman",fontface = "bold")

ggsave("boxplot_M.png", plot = p, width =8, height = 6)

#############RMSEt2#################
# Combine results for all temperatures
combined_results <- bind_rows(
  result_25C %>% mutate(temperature = "25C"),
  result_40C %>% mutate(temperature = "40C"),
  result_50C %>% mutate(temperature = "50C"),
  result_60C %>% mutate(temperature = "60C")
)

# Define condition order for the factor
condition_order <- c(
  "Pure Water", "10% Methanol", "20% Methanol", "30% Methanol",
  "40% Methanol", "50% Methanol", "60% Methanol", "70% Methanol",
  "80% Methanol", "90% Methanol", "Pure Methanol"
)
combined_results$condition <- factor(combined_results$condition, levels = condition_order)

# Assign to temp_data_t2
temp_data_t2 <- combined_results

# Define custom colors for each temperature
custom_colors <- c("25C" = "navyblue", "40C" = "darkgreen", "50C" = "#B8860B", "60C" = "darkred")

# Add numeric_condition for easier modeling and plotting
temp_data_t2 <- temp_data_t2 %>%
  mutate(
    numeric_condition = case_when(
      condition == "Pure Water" ~ 0,
      condition == "Pure Methanol" ~ 100,
      TRUE ~ as.numeric(gsub("[^0-9]", "", as.character(condition)))
    )
  )

# Ensure a continuous range of numeric_condition
temp_data_t2 <- temp_data_t2 %>%
  arrange(temperature, numeric_condition) %>%
  group_by(temperature) %>%
  complete(numeric_condition = full_seq(numeric_condition, 1)) %>%
  ungroup()

# Fit a single linear model after completing missing rows
lm_model <- lm(mean_delta_t2 ~ numeric_condition * as.factor(temperature), 
               data = temp_data_t2 %>% filter(!is.na(mean_delta_t2)))

# Add linear predicted values
temp_data_t2 <- temp_data_t2 %>%
  mutate(predicted_values = predict(lm_model, newdata = temp_data_t2))

# Fit LOESS model per temperature
temp_data_t2 <- temp_data_t2 %>%
  group_by(temperature) %>%
  mutate(
    loess_fit = list(loess(mean_delta_t2 ~ numeric_condition, data = cur_data(), span = 0.75)),
    predicted_loess = predict(loess_fit[[1]], newdata = cur_data())
  ) %>%
  ungroup()

# Find min and max values for plotting
min_value_t2 <- min(temp_data_t2$mean_delta_t2, na.rm = TRUE)
max_value_t2 <- max(temp_data_t2$mean_delta_t2, na.rm = TRUE)

# Calculate accuracy metrics for linear model (using a cubic polynomial model for evaluation)
temp_models_t2 <- temp_data_t2 %>%
  filter(!is.na(mean_delta_t2)) %>%
  group_by(temperature) %>%
  nest() %>%
  mutate(
    model = map(data, ~ lm(mean_delta_t2 ~ poly(numeric_condition, 3), data = .x)),
    r_squared = map_dbl(model, ~ glance(.x)$r.squared),
    rmse = map_dbl(model, ~ sqrt(mean(residuals(.x)^2))),
    mae = map_dbl(model, ~ mean(abs(residuals(.x)))),
    rmse_percentage = map_dbl(data, ~ {
      preds <- predict(lm(mean_delta_t2 ~ poly(numeric_condition, 3), data = .x))
      range_y <- max(.x$mean_delta_t2) - min(.x$mean_delta_t2)
      rmse_val <- sqrt(mean((.x$mean_delta_t2 - preds)^2))
      (rmse_val / range_y) * 100
    })
  )

# Calculate accuracy metrics for LOESS model
temp_models_t2_loess <- temp_data_t2 %>%
  filter(!is.na(mean_delta_t2)) %>%
  group_by(temperature) %>%
  summarize(
    r_squared_loess = {
      loess_resid = mean_delta_t2 - predicted_loess
      1 - (sum(loess_resid^2) / sum((mean_delta_t2 - mean(mean_delta_t2))^2))
    },
    rmse_loess = {
      loess_resid = mean_delta_t2 - predicted_loess
      sqrt(mean(loess_resid^2))
    },
    mae_loess = {
      loess_resid = mean_delta_t2 - predicted_loess
      mean(abs(loess_resid))
    },
    rmse_percentage_loess = {
      loess_resid = mean_delta_t2 - predicted_loess
      range_y <- max(mean_delta_t2) - min(mean_delta_t2)
      (sqrt(mean(loess_resid^2)) / range_y) * 100
    }
  )

# Combine accuracy metrics
accuracy_combined <- temp_models_t2 %>%
  select(temperature, rmse_percentage) %>%
  left_join(
    temp_models_t2_loess %>% select(temperature, rmse_percentage_loess),
    by = "temperature"
  )

# Combine accuracy metrics
accuracy_combined <- temp_models_t2 %>%
  select(temperature, rmse_percentage) %>%
  left_join(
    temp_models_t2_loess %>% select(temperature, rmse_percentage_loess),
    by = "temperature"
  )

# Prepare a table with 4 rows and 2 columns for annotation
my_table <- data.frame(
  Temperature = accuracy_combined$temperature,
  `RMSE (%) (Linear)` = round(accuracy_combined$rmse_percentage, 3),
  `RMSE (%) (LOESS)` = round(accuracy_combined$rmse_percentage_loess, 3)
)

colnames(my_table) <- c("Temperature", "RMSE_Linear(%)", "RMSE_LOESS(%)")


tt <- ttheme_minimal(
  core = list(
    fg_params = list(
      fontface = c("bold", "bold", "bold", "bold"),  # Bold text for all rows
      col = c("navyblue", "darkgreen", "#B8860B", "darkred")  # Row colors
    ),
    bg_params = list(col = "black", lwd = 1)
  ),
  colhead = list(
    fg_params = list(fontface = "bold", col = "black"),  # Black title
    bg_params = list(col = "black", lwd = 1)
  )
)

# Apply the custom theme to your table
table_grob <- tableGrob(my_table, rows = NULL, theme = tt)


# Split data into segments for numeric_condition <= 80 and > 80
temp_data_t2$segment <- ifelse(temp_data_t2$numeric_condition > 80, "above_80", "below_80")

p_t2 <- ggplot(temp_data_t2, aes(x = numeric_condition, y = mean_delta_t2, color = as.factor(temperature))) +
  geom_point(size = 3, na.rm = TRUE) +  # Points for original data
  # LOESS dashed line
  geom_line(aes(y = predicted_loess, group = temperature, linetype = "LOESS"), size = 1) +
  # Linear solid line for numeric_condition <= 80
  geom_line(
    data = temp_data_t2 %>% filter(segment == "below_80"),
    aes(y = predicted_values, group = temperature, linetype = "Linear (under 80%)"),
    size = 1.8
  ) +
  # Linear dotted line for numeric_condition > 80
  geom_line(
    data = temp_data_t2 %>% filter(segment == "above_80"),
    aes(y = predicted_values, group = temperature, linetype = "Linear (80%-100%)"),
    size = 1.2
  ) +
  labs(
    x = "Methanol (%)",
    y = expression(bold("Mean"~Delta~t[2]~"(s)")),
    linetype = "Line Type"
  ) +
  theme_minimal() +
  theme(
    axis.text.x = element_text(size = 18, family = "Times New Roman", face = "bold"),
    axis.text.y = element_text(size = 18, family = "Times New Roman", face = "bold"),
    axis.title = element_text(size = 18, family = "Times New Roman", face = "bold"),
    legend.text = element_text(size = 15, family = "Times New Roman", face = "bold"),
    legend.title = element_text(size = 15, family = "Times New Roman", face = "bold"),
    legend.key.height = unit(1.5, "lines"),
    legend.position = c(0.83, 0.55),
    panel.background = element_blank(),
    plot.background = element_blank(),
    panel.grid.major = element_blank(),
    panel.grid.minor = element_blank(),
    panel.border = element_rect(color = "black", fill = NA)
  ) +
  scale_color_manual(values = custom_colors, guide = "none") +  # Remove color legend
  scale_linetype_manual(
    values = c(
      "Linear (under 80%)" = "solid",
      "Linear (80%-100%)" = "dotted",
      "LOESS" = "dashed"
    ),
    breaks = c("Linear (under 80%)", "Linear (80%-100%)", "LOESS")  # Set the order explicitly
  ) +
  coord_cartesian(ylim = c(min_value_t2, max_value_t2)) +
  scale_x_continuous(breaks = seq(0, 100, 10), labels = as.character(seq(0, 100, 10))) +
  # Add the table annotation
  annotation_custom(
    grob = table_grob,
    xmin = 50, xmax = 90,   # Adjust as needed for positioning
    ymin = max_value_t2 - 100,
    ymax = max_value_t2 
  )


ggsave("RMSE_t2_M.png", plot = p_t2, width =8, height = 6)


############rmse_cap#############
###################
condition_order <- c(
  "Pure Water", "10% Methanol", "20% Methanol", "30% Methanol",
  "40% Methanol", "50% Methanol", "60% Methanol", "70% Methanol",
  "80% Methanol", "90% Methanol", "Pure Methanol"
)


# Combine results for all temperatures
all_results <- bind_rows(
  result_25C %>% mutate(Temperature = "25C"),
  result_40C %>% mutate(Temperature = "40C"),
  result_50C %>% mutate(Temperature = "50C"),
  result_60C %>% mutate(Temperature = "60C")
)

# Convert 'condition' to factor with specified levels
all_results$condition <- factor(all_results$condition, levels = condition_order)

# Summarize data by condition and temperature
all_results_summarized <- all_results %>%
  group_by(condition, Temperature) %>%
  summarize(
    mean_cap = mean(mean_cap),
    sd_cap = mean(sd_cap),
    .groups = 'drop'
  )

# Add numeric_condition for easier modeling
all_results_summarized <- all_results_summarized %>%
  mutate(numeric_condition = as.numeric(gsub("[^0-9]", "", as.character(condition))),
         numeric_condition = ifelse(is.na(numeric_condition), 
                                    ifelse(condition == "Pure Water", 0, 100), numeric_condition))

# Fit models for mean_cap using both Linear and LOESS
temp_models_cap <- all_results_summarized %>%
  group_by(Temperature) %>%
  nest() %>%
  mutate(
    # Fit Linear model
    linear_model = map(data, ~ lm(mean_cap ~ poly(numeric_condition, 3), data = .x)),
    # Fit LOESS model
    loess_model = map(data, ~ loess(mean_cap ~ numeric_condition, data = .x, span = 0.75)),
    # Calculate predictions for Linear and LOESS
    linear_predictions = map(linear_model, ~ augment(.x)),
    loess_predictions = map(loess_model, ~ data.frame(.x$fitted)),
    # Calculate accuracy metrics
    r_squared_linear = map_dbl(linear_model, ~ glance(.x)$r.squared),
    rmse_linear = map_dbl(linear_model, ~ sqrt(mean(residuals(.x)^2))),
    rmse_loess = map_dbl(loess_model, ~ sqrt(mean((.x$residuals)^2))),
    mae_linear = map_dbl(linear_model, ~ mean(abs(residuals(.x))))
  ) %>%
  mutate(
    range_y = map_dbl(data, ~ max(.x$mean_cap) - min(.x$mean_cap)),  # Range of the observed values
    rmse_percentage_linear = (rmse_linear / range_y) * 100,
    rmse_percentage_loess = (rmse_loess / range_y) * 100
  )

# Print accuracy metrics for each temperature
temp_models_cap %>%
  select(Temperature, r_squared_linear, rmse_percentage_linear, rmse_percentage_loess)
```

```
## # A tibble: 4 × 4
## # Groups:   Temperature [4]
##   Temperature r_squared_linear rmse_percentage_linear rmse_percentage_loess
##   <chr>                  <dbl>                  <dbl>                 <dbl>
## 1 25C                    0.529                   18.4                  17.2
## 2 40C                    0.805                   11.1                  11.1
## 3 50C                    0.794                   14.1                  12.0
## 4 60C                    0.585                   22.1                  20.4
```

```
# Find min and max values for the plot
min_value_cap <- min(all_results_summarized$mean_cap, na.rm = TRUE)
max_value_cap <- max(all_results_summarized$mean_cap, na.rm = TRUE) + 5  # Add buffer to max for annotations

# Prepare a table with RMSE for both Linear and LOESS
accuracy_combined <- temp_models_cap %>%
  select(
    Temperature, 
    `RMSE% (Linear)` = rmse_percentage_linear, 
    `RMSE% (LOESS)` = rmse_percentage_loess
  )

# Define custom theme for table
tt <- ttheme_minimal(
  core = list(
    fg_params = list(
      fontface = c("bold", "bold", "bold", "bold"),  # Bold text for all rows
      col = c("navyblue", "darkgreen", "#B8860B", "darkred")  # Row colors
    ),
    bg_params = list(col = "black", lwd = 1)
  ),
  colhead = list(
    fg_params = list(fontface = "bold", col = "black"),  # Black title
    bg_params = list(col = "black", lwd = 1)
  )
)

# Create table grob
table_grob <- tableGrob(accuracy_combined, rows = NULL, theme = tt)

# Add a separate legend for line types
p <- ggplot(all_results_summarized, aes(x = numeric_condition, y = mean_cap, group = Temperature)) +
  geom_point(aes(color = Temperature), size = 3, show.legend = FALSE) +  # Points for data, no legend
  geom_smooth(aes(linetype = "Linear", color = Temperature), method = "lm", se = FALSE, size = 1) +  # Linear model
  geom_smooth(aes(linetype = "LOESS", color = Temperature), method = "loess", se = FALSE, size = 1.8) +  # LOESS model
  labs(x = "Methanol (%)", y = expression(bold(Mean~Delta~Cap~(fF))), linetype = "Model") +
  theme_minimal() +
  theme(
    axis.text.x = element_text(size = 18, family = "Times New Roman", face = "bold"),
    axis.text.y = element_text(size = 18, family = "Times New Roman", face = "bold"),
    axis.title = element_text(size = 18, family = "Times New Roman", face = "bold"),
    legend.text = element_text(size = 18, family = "Times New Roman", face = "bold"),
    legend.title = element_text(size = 18, family = "Times New Roman", face = "bold"),
    panel.background = element_blank(),
    plot.background = element_blank(),
    panel.grid.major = element_blank(),
    panel.grid.minor = element_blank(),
    panel.border = element_rect(color = "black", fill = NA),
    legend.position = c(0.1, 0.15)  # Adjust legend position as needed
  ) +
  coord_cartesian(ylim = c(min_value_cap, max_value_cap)) +
  scale_color_manual(values = c("25C" = "navyblue", "40C" = "darkgreen", "50C" = "#B8860B", "60C" = "darkred"), guide = "none") +
  scale_linetype_manual(
    values = c("Linear" = "solid", "LOESS" = "dashed"),
    breaks = c("Linear", "LOESS")  # Order of the legend
  ) +
  guides(
    linetype = guide_legend(override.aes = list(color = "black"))  # Black line symbols in legend
  ) +
  # Add table annotation
  annotation_custom(
    grob = table_grob,
    xmin = 55, xmax = 90,   # Adjust as needed for positioning
    ymin = max_value_cap - 7,
    ymax = max_value_cap
  )

ggsave("RMSE_cap_M.png", plot = p, width =8, height = 6)


#_______________________-arrange
#___________________________________
library(magick)

# Load and resize the images
RMSE_t2_E <- image_read("RMSE_t2_E.png") %>% image_background("white") %>% image_resize("2000x600")  # Make RMSE_t2 bigger
RMSE_cap_E <- image_read("RMSE_cap_E.png") %>% image_background("white") %>% image_resize("800x600")  # Shrink RMSE_cap

RMSE_t2_M <- image_read("RMSE_t2_M.png") %>% image_background("white") %>% image_resize("2000x600")  # Make RMSE_t2 bigger
RMSE_cap_M <- image_read("RMSE_cap_M.png") %>% image_background("white") %>% image_resize("800x600")  # Shrink RMSE_cap to 


boxplot_Ethanol <- image_read("BOXPLOT_t2_E.png") %>% image_background("white") %>% image_resize("2000x600")  # Make RMSE_t2 bigger
boxplot_Methanol<- image_read("boxplot_M.png") %>% image_background("white") %>% image_resize("800x600")  # Shrink RMSE_cap to 

# Arrange the first row:  RMSE_cap_E, RMSE_t2_E
# Create horizontal spacer
horizontal_spacer <- image_blank(width = 50, height = image_info(RMSE_cap_E)$height, color = "white")  # 50px wide blank space


row1 <- image_append(c(RMSE_t2_E, horizontal_spacer, RMSE_t2_M))

row2 <- image_append(c(RMSE_cap_E, horizontal_spacer, RMSE_cap_M))

row3 <- image_append(c(boxplot_Ethanol, horizontal_spacer, boxplot_Methanol))

# Create vertical spacer for alignment
vertical_spacer <- image_blank(width = image_info(row1)$width, height = 50, color = "white")  # 200px height blank space

# Stack the rows vertically with spacers between them
final_image <- image_append(c(row1, vertical_spacer, row2, vertical_spacer, row3), stack = TRUE)

# Save the final combined image
image_write(final_image, "combined_plot1.png")
knitr::include_graphics("combined_plot1.png")
```

## 1.4 Figure6

```
rm(list=ls())

#_______________________
# Libraries
#_______________________
library(nortest)
library(writexl)
library(tidyverse)
library(openxlsx)
library(dplyr)
library(Matrix)
library(lme4)
library(ggplot2)
library(car)
library(extrafont)
library(stringr)
library(gridExtra)
library(combinat)
library(tidyr)
library(cowplot)
library(ggpubr)
library(rlang)
library(broom)
library(purrr)
library(ggpmisc)  # For adding equation and R² to plot

# Import fonts
# font_import(prompt = FALSE)
loadfonts(device = "win")

#_______________________
# Function Definitions
#_______________________
get_sharpness_time <- function(data) {
  # Loop through the data until the cap value increases significantly
  for (i in 2:nrow(data)) {
    # Check if there's a notable increase in the Cap value after a steady range
    if (data$Cap[i] > 160) {
      return(data$Time[i])
    }
  }
  
  # Return NA if no sharpness time is found
  return(NA)
}


# Function to extract timeline points and calculate delta times
get_timeline_points <- function(data, condition, run_name) {
  # Filter data for the specified condition and run
  # Group and calculate cumulative time
  data <- data %>%
    group_by(Condition) %>%
    mutate(Cumulative_Time = cumsum(Time)) %>%
    ungroup() %>%
    mutate(Time = Cumulative_Time) %>%
    select(-Cumulative_Time)
  
  # Replace "-run" with "_run" in the Condition column to ensure uniformity
  data <- data %>%
    mutate(Condition = str_replace(Condition, "-run", "_run"))
  
  filtered_data <- data %>% filter(Condition == paste(condition, run_name, sep = "_"))
  
  # Get the sharpness time (steepest capacitance transition)
  sharpness_time <- get_sharpness_time(filtered_data)
  
  # Filter data based on the sharpness time
  data_before_sharpness <- filtered_data %>%
    filter(Time < sharpness_time)
  
  
  # Filter data based on the sharpness time
  data_after_sharpness <- filtered_data %>%
    filter(Time >= sharpness_time)
  
  # Define timeline points based on the sharpness
  max_cap_value <- max(data_after_sharpness$Cap, na.rm = TRUE)
  lower_bound <- max_cap_value - 5
  upper_bound <- max_cap_value + 5
  
  cap_range_data <- data_after_sharpness %>%
    filter(Cap >= lower_bound & Cap <= upper_bound)
  
  first_flat_time <- cap_range_data$Time[1]
  last_flat_time <- cap_range_data$Time[nrow(cap_range_data)]
  
  data_after_last_time <- data_after_sharpness %>%
    filter(Time > last_flat_time)
  
  delta_t1 <- max(data_before_sharpness$Time) - min(data_before_sharpness$Time)
  delta_t2 <- max(cap_range_data$Time) - min(cap_range_data$Time)
  delta_t3 <- max(data_after_last_time$Time) - min(data_after_last_time$Time)
  
  TOE <- max(filtered_data$Time)-min(filtered_data$Time)
  
  return(list(
    before_flat = data_before_sharpness,
    flat = cap_range_data,
    after_flat = data_after_last_time,
    delta_t1 = delta_t1,
    delta_t2 = delta_t2,
    delta_t3 = delta_t3,
    TOE = TOE
  ))
}

# Function to calculate Mean and SD of delta_t1, delta_t3, and TOE for each combination of three runs
calculate_mean_sd_combinations <- function(data) {
  condition <- unique(data$condition)
  
  # Generate all possible combinations of three runs
  run_combinations <- combn(data$run_name, 3, simplify = FALSE)
  
  # Calculate Mean and SD of delta_t1, delta_t3, and TOE for each combination
  mean_delta_t1 <- sapply(run_combinations, function(runs) {
    selected_delta_t1 <- data %>% filter(run_name %in% runs) %>% pull(delta_t1)
    mean(selected_delta_t1)
  })
  
  sd_delta_t1 <- sapply(run_combinations, function(runs) {
    selected_delta_t1 <- data %>% filter(run_name %in% runs) %>% pull(delta_t1)
    sd(selected_delta_t1)
  })
  
  mean_delta_t2 <- sapply(run_combinations, function(runs) {
    selected_delta_t2 <- data %>% filter(run_name %in% runs) %>% pull(delta_t2)
    mean(selected_delta_t2)
  })
  
  sd_delta_t2 <- sapply(run_combinations, function(runs) {
    selected_delta_t2 <- data %>% filter(run_name %in% runs) %>% pull(delta_t2)
    sd(selected_delta_t2)
  })
  
  mean_delta_t3 <- sapply(run_combinations, function(runs) {
    selected_delta_t3 <- data %>% filter(run_name %in% runs) %>% pull(delta_t3)
    mean(selected_delta_t3)
  })
  
  sd_delta_t3 <- sapply(run_combinations, function(runs) {
    selected_delta_t3 <- data %>% filter(run_name %in% runs) %>% pull(delta_t3)
    sd(selected_delta_t3)
  })
  
  mean_toe <- sapply(run_combinations, function(runs) {
    selected_toes <- data %>% filter(run_name %in% runs) %>% pull(TOE)
    mean(selected_toes)
  })
  
  sd_toe <- sapply(run_combinations, function(runs) {
    selected_toes <- data %>% filter(run_name %in% runs) %>% pull(TOE)
    sd(selected_toes)
  })
  
  # Create a data frame with the combinations, their Mean and SD for delta_t1, delta_t3, and TOE
  mean_sd_table <- data.frame(
    condition = condition,
    combination = sapply(run_combinations, paste, collapse = ", "),
    mean_delta_t1 = mean_delta_t1,
    sd_delta_t1 = sd_delta_t1,
    mean_delta_t2 = mean_delta_t2,
    sd_delta_t2 = sd_delta_t2,
    mean_delta_t3 = mean_delta_t3,
    sd_delta_t3 = sd_delta_t3,
    mean_toe = mean_toe,
    sd_toe = sd_toe
  )
  
  # Return the Mean and SD table
  return(mean_sd_table)
}

# Function to calculate mean Cap for the selected runs
calculate_mean_cap <- function(data, condition, combination) {
  # Extract the runs from the combination column
  selected_runs <- str_split(combination, ", ") %>% unlist()
  
  # Filter the data for the specific condition and selected runs
  filtered_data <- data %>%
    filter(str_detect(Condition, paste0(condition, "_(", paste0(selected_runs, collapse = "|"), ")")))
  
  # Calculate the mean Cap
  mean_cap <- mean(filtered_data$Cap, na.rm = TRUE)
  sd_cap <- sd(filtered_data$Cap, na.rm = TRUE)
  return(list(mean_cap = mean_cap, sd_cap = sd_cap))
}

# Function to Process Data for a Given Temperature
process_data <- function(data, temperature) {
  
  # Replace "-run" with "_run" in the Condition column to ensure uniformity
  data <- data %>%
    mutate(Condition = str_replace(Condition, "-run", "_run"))
  
  unique_conditions <- unique(data$Condition)
  
  split_conditions <- data.frame(
    condition = sapply(strsplit(unique_conditions, "_"), `[`, 1),
    run_name = sapply(strsplit(unique_conditions, "_"), `[`, 2)
  )
  
  
  results <- split_conditions %>%
    mutate(timeline_points = map2(condition, run_name, ~get_timeline_points(data, .x, .y)))
  
  final_results <- results %>%
    rowwise() %>%
    mutate(
      before_flat = list(timeline_points$before_flat),
      flat = list(timeline_points$flat),
      after_flat = list(timeline_points$after_flat),
      delta_t1 = timeline_points$delta_t1,
      delta_t2 = timeline_points$delta_t2,
      delta_t3 = timeline_points$delta_t3,
      TOE = timeline_points$TOE
    ) %>%
    select(-timeline_points) %>%
    ungroup()
  
  final_results <- final_results %>%
    mutate(
      before_flat = sapply(before_flat, function(x) toString(x$Time)),
      flat = sapply(flat, function(x) toString(x$Time)),
      after_flat = sapply(after_flat, function(x) toString(x$Time))
    )
  
  # Save the final results as an Excel file
  write_xlsx(final_results, paste0("timeline_results__ethanol_water", temperature, ".xlsx"))
  mean_sd_combinations_table <- final_results %>%
    group_by(condition) %>%
    do(calculate_mean_sd_combinations(.)) %>%
    ungroup()
  
  # Find the combination with the smallest combined SD for delta_t1, delta_t2, delta_t3, and TOE
  best_combinations_table <- mean_sd_combinations_table %>%
    group_by(condition) %>%
    mutate(total_sd = sd_delta_t1 + sd_delta_t2 + sd_delta_t3 + sd_toe) %>%
    slice_min(total_sd) %>%
    ungroup()
  
  # Apply the function to calculate mean Cap for each row of best_combinations_table
  best_combinations_table <- best_combinations_table %>%
    rowwise() %>%
    mutate(
      mean_cap = calculate_mean_cap(data, as.character(condition), as.character(combination))$mean_cap,
      sd_cap = calculate_mean_cap(data, as.character(condition), as.character(combination))$sd_cap
    ) %>%
    ungroup()
  
  # Define the correct order of conditions
  condition_order <- c(
    "Pure Water", "10% Ethanol", "20% Ethanol", "30% Ethanol",
    "40% Ethanol", "50% Ethanol", "60% Ethanol", "70% Ethanol",
    "80% Ethanol", "90% Ethanol", "Pure Ethanol"
  )
  
  # Convert the condition column to a factor with the specified levels
  best_combinations_table$condition <- factor(best_combinations_table$condition, levels = condition_order)
  
  # Arrange the data frame based on the factor levels
  best_combinations_table <- best_combinations_table %>%
    arrange(condition)
  
  # Save the tables to Excel
  write_xlsx(list(
    Mean_SD_Combinations = mean_sd_combinations_table,
    Best_Combinations = best_combinations_table
  ), paste0("mean_sd_combinations_and_selected_runs_ethanol_water", temperature, ".xlsx"))
  
  return(best_combinations_table)
}
# Process for each temperature
data_25C <- read.xlsx("reshape_Water-Ethanol.xlsx", sheet = "25C", colNames = TRUE)
result_25C <- process_data(data_25C, "25C")
##########R2################
condition_order <- c(
  "Pure Water", "10% Ethanol", "20% Ethanol", "30% Ethanol",
  "40% Ethanol", "50% Ethanol", "60% Ethanol", "70% Ethanol",
  "80% Ethanol", "90% Ethanol", "Pure Ethanol"
)

combined_results <- bind_rows(
  result_25C %>% mutate(temperature = "25C")
)

plot_cubic_models_25C <- function(data) {
  # Filter data for 25C temperature
  data_25C <- data %>% filter(temperature == "25C")
  
  # Function to calculate accuracy metrics (R², RMSE, etc.)
  calculate_metrics <- function(model, data, y_var) {
    predictions <- predict(model, data)
    residuals <- data[[y_var]] - predictions
    r_squared <- 1 - sum(residuals^2) / sum((data[[y_var]] - mean(data[[y_var]]))^2)  # Manually calculate R²
    rmse <- sqrt(mean(residuals^2))
    mae <- mean(abs(residuals))
    range_y <- max(data[[y_var]]) - min(data[[y_var]])
    rmse_percentage <- (rmse / range_y) * 100
    
    return(list(r_squared = r_squared, rmse = rmse, mae = mae, rmse_percentage = rmse_percentage))
  }
  
  # Fit cubic and LOESS models and calculate metrics for each Delta
  lm_delta_t1 <- lm(mean_delta_t1 ~ as.numeric(condition), data = data_25C)
  loess_delta_t1 <- loess(mean_delta_t1 ~ as.numeric(condition), data = data_25C, span = 0.75)
  metrics_delta_t1_lm <- calculate_metrics(lm_delta_t1, data_25C, "mean_delta_t1")
  metrics_delta_t1_loess <- calculate_metrics(loess_delta_t1, data_25C, "mean_delta_t1")
  
  lm_delta_t2 <- lm(mean_delta_t2 ~ as.numeric(condition), data = data_25C)
  loess_delta_t2 <- loess(mean_delta_t2 ~ as.numeric(condition), data = data_25C, span = 0.75)
  metrics_delta_t2_lm <- calculate_metrics(lm_delta_t2, data_25C, "mean_delta_t2")
  metrics_delta_t2_loess <- calculate_metrics(loess_delta_t2, data_25C, "mean_delta_t2")
  
  lm_delta_t3 <- lm(mean_delta_t3 ~ as.numeric(condition), data = data_25C)
  loess_delta_t3 <- loess(mean_delta_t3 ~ as.numeric(condition), data = data_25C, span = 0.75)
  metrics_delta_t3_lm <- calculate_metrics(lm_delta_t3, data_25C, "mean_delta_t3")
  metrics_delta_t3_loess <- calculate_metrics(loess_delta_t3, data_25C, "mean_delta_t3")
  
  lm_toe <- lm(mean_toe ~ as.numeric(condition), data = data_25C)
  loess_toe <- loess(mean_toe ~ as.numeric(condition), data = data_25C, span = 0.75)
  metrics_toe_lm <- calculate_metrics(lm_toe, data_25C, "mean_toe")
  metrics_toe_loess <- calculate_metrics(loess_toe, data_25C, "mean_toe")
  
  # Function to create plots with LOESS and linear model
  create_combined_plot <- function(data, y_var, y_label, lm_model, loess_model, metrics_lm, metrics_loess, color) {
    min_value <- min(data[[y_var]], na.rm = TRUE)
    max_value <- max(data[[y_var]], na.rm = TRUE)
    annotation_x <- max(as.numeric(data$condition)) - 1  # Adjust annotation position
    
    values <- if (y_var == "mean_delta_t1") {
      list(
        max_value = max_value - (max_value - min_value) * 0.1,  # Default for mean_delta_t1
        legend = c(0.2, 0.8)  # Legend position for mean_delta_t1
      )
    } else {
      list(
        max_value = max_value - (max_value - min_value) * 0.05,  # Move up slightly for other conditions
        legend = c(0.2, 0.25)  # Legend position for other conditions
      )
    }
    
    # Access the calculated values
    max_value <- values$max_value
    legend_position <- values$legend
    
    
    # Table for R² metrics
    table_data <- data.frame(
      Model = c("Linear", "LOESS"),
      R2 = c(round(metrics_lm$r_squared, 3), round(metrics_loess$r_squared, 3))
    )
    
    table_grob <- tableGrob(
      table_data,
      rows = NULL,
      theme = ttheme_minimal(
        core = list(bg_params = list(col = "black", lwd = 1),
                    fg_params = list(fontface = "bold", fontsize = 12)),
        colhead = list(bg_params = list(col = "black", lwd = 1),
                       fg_params = list(fontface = "bold", fontsize = 12))
      )
    )
    ggplot(data, aes(x = as.numeric(condition), y = !!sym(y_var))) +
      # Points for "25C"
      geom_point(aes(color = "25C"), size = 4, show.legend = TRUE) +
      
      # Linear model (solid line)
      # Linear model (solid line)
      geom_smooth(method = "lm", formula = y ~ x, se = FALSE,
                  aes(linetype = "Linear"), color = color, size = 1) +
      
      # LOESS model (dashed line)
      geom_line(aes(y = predict(loess_model, data), linetype = "LOESS"),
                color = color, size = 1.8) +
      
      # Manual legends and scales
      labs(
        x = "Ethanol (%)",
        y = y_label,
        color = "Condition",  # Legends for points
        linetype = "Model"    # Legends for lines
      ) +
      scale_linetype_manual(values = c("Linear" = "solid", "LOESS" = "dashed")) +
      scale_color_manual(values = c("25C" = "#B8860B")) +
      
      # Fix the legend guides
      guides(
        color = guide_legend(override.aes = list(linetype = "blank", shape = 16, size = 4)),  # Ensure points are circles
        linetype = guide_legend(override.aes = list(color = color, size = c(0.8, 1.5)))              # Ensure lines are lines
      ) +
      
      # Adjust the theme and legend
      theme_minimal() +
      theme(
        axis.text.x = element_text(size = 20, family = "Times New Roman", face = "bold"),
        axis.text.y = element_text(size = 20, family = "Times New Roman", face = "bold"),
        axis.title = element_text(size = 20, family = "Times New Roman", face = "bold"),
        legend.text = element_text(size = 15, family = "Times New Roman", face = "bold"),
        legend.title =  element_text(size = 15, family = "Times New Roman", face = "bold"),
        legend.position = legend_position, 
        legend.direction = "vertical",
        legend.spacing.y = unit(0.002, 'cm'),
        #legend.key.size = unit(0.8, 'lines'),
        panel.background = element_blank(),
        plot.background = element_blank(),
        panel.grid.major = element_blank(),
        panel.grid.minor = element_blank(),
        panel.border = element_rect(color = "black", fill = NA)
      )+
      annotation_custom(
        table_grob,
        xmin = max(as.numeric(data$condition)) - 3,  # Adjust to position in top-right corner
        xmax = max(as.numeric(data$condition))-1.5,      # Align to the right edge
        ymin = max_value - (max_value - min_value) * 0.1,  # Slightly below the top
        ymax = max_value                              # Align to the top edge
      ) 
    
    
  }
  
  # Combined plots for each metric
  p1 <- create_combined_plot(data_25C, "mean_delta_t1", expression(bold(Delta~t[1]~"(s)")), lm_delta_t1, loess_delta_t1, metrics_delta_t1_lm, metrics_delta_t1_loess, "navyblue")
  p2 <- create_combined_plot(data_25C, "mean_delta_t2", expression(bold(Delta~t[2]~"(s)")), lm_delta_t2, loess_delta_t2, metrics_delta_t2_lm, metrics_delta_t2_loess, "navyblue")
  p3 <- create_combined_plot(data_25C, "mean_delta_t3", expression(bold(Delta~t[3]~"(s)")), lm_delta_t3, loess_delta_t3, metrics_delta_t3_lm, metrics_delta_t3_loess, "navyblue")
  p4 <- create_combined_plot(data_25C, "mean_toe", "TOE (s)", lm_toe, loess_toe, metrics_toe_lm, metrics_toe_loess, "navyblue")
  
  plot_grid <- arrangeGrob(p1, p2, p3, p4, ncol = 4)
  ggsave("R2E.png", plot = plot_grid, width = 16, height = 6)
}


combined_results$condition <- factor(combined_results$condition, levels = condition_order)


plot_cubic_models_25C(combined_results)
##############RMSE_cap###########
condition_order <- c(
  "Pure Water", "10% Ethanol", "20% Ethanol", "30% Ethanol",
  "40% Ethanol", "50% Ethanol", "60% Ethanol", "70% Ethanol",
  "80% Ethanol", "90% Ethanol", "Pure Ethanol"
)

all_results <- bind_rows(
  result_25C %>% mutate(Temperature = "25C")
)


# Now convert the 'Condition' column to a factor with the specified levels
all_results$condition <- factor(all_results$condition, levels = condition_order)
all_results_summarized <- all_results %>%
  group_by(condition, Temperature) %>%
  summarize(
    mean_cap = mean(mean_cap),
    sd_cap = mean(sd_cap),
    .groups = 'drop'  # Ensures we drop the grouping after summarizing
  )


# Fit separate models for 25C and 40C using cubic polynomial fit for mean_delta__cap
temp_models_cap <- all_results_summarized %>%
  group_by(Temperature) %>%
  nest() %>%
  mutate(model = map(data, ~ lm(mean_cap ~ as.numeric(condition), data = .x)))  # Linear model

# Calculate accuracy metrics for mean_delta__cap
temp_models_cap <- temp_models_cap %>%
  mutate(
    predictions = map(model, ~ augment(.x)),  # Get predictions
    r_squared = map_dbl(model, ~ glance(.x)$r.squared),  # R-squared
    range_y = map_dbl(data, ~ max(.x$mean_cap) - min(.x$mean_cap)),  # Range of the observed values
    rmse = map_dbl(model, ~ sqrt(mean(residuals(.x)^2))),  # Root Mean Squared Error
    mae = map_dbl(model, ~ mean(abs(residuals(.x))))  # Mean Absolute Error
  ) %>%
  mutate(
    rmse_percentage = (rmse / range_y) * 100,  # RMSE as percentage of the range
    mae_percentage = (mae / range_y) * 100  # MAE as percentage of the range
  )

# Print accuracy metrics for each Temperature for mean_cap
temp_models_cap %>%
  select(Temperature, r_squared, rmse, mae, rmse_percentage, mae_percentage)
```

```
## # A tibble: 1 × 6
## # Groups:   Temperature [1]
##   Temperature r_squared  rmse   mae rmse_percentage mae_percentage
##   <chr>           <dbl> <dbl> <dbl>           <dbl>          <dbl>
## 1 25C             0.835  1.94  1.72            12.5           11.1
```

```
min_value_cap <- min(all_results_summarized$mean_cap, na.rm = TRUE)
max_value_cap <- max(all_results_summarized$mean_cap, na.rm = TRUE)

max_value_cap = max_value_cap +5

custom_colors <- c("25C" = "navyblue")

# Add LOESS model and compare RMSE with a table
p <- ggplot(all_results_summarized, aes(x = condition, y = mean_cap, group = Temperature)) +
  # Points with a fixed color (#B8860B) and a legend
  geom_point(aes(color = "25C"), size = 4, show.legend = TRUE) +
  
  # Linear fit with solid navy blue line
  geom_smooth(aes(linetype = "Linear"), method = "lm", se = FALSE, size = 1, color = "navyblue") +
  
  # LOESS fit with dashed navy blue line
  geom_smooth(aes(linetype = "LOESS"), method = "loess", se = FALSE, size = 1.8, color = "navyblue") +
  
  labs(
    x = "Ethanol (%)",
    y = expression(bold(Mean~Delta~Cap~(fF))),
    color = "Condition",  # Title for points legend
    linetype = "Model"    # Title for lines legend
  ) +
  
  # Adjust the legend aesthetics
  guides(
    color = guide_legend(
      override.aes = list(
        shape = 16,       # Circle shape for points
        size = 4,         # Size of points
        color = "#B8860B" # Fixed color for points
      )
    ),
    linetype = guide_legend(
      override.aes = list(
        color = "navyblue",    # Navy blue legend lines
        size = c(0.8, 1.5)     # Thin solid line and thicker dashed line
      )
    )
  ) +
  
  # Apply custom scales for linetypes
  scale_linetype_manual(values = c("Linear" = "solid", "LOESS" = "dashed")) +
  
  # Apply custom scales for the points legend
  scale_color_manual(values = c("25C" = "#B8860B")) +
  
  # Customize x-axis labels
  scale_x_discrete(
    limits = condition_order,
    labels = c("0", "", "20", "", "40", "", "60", "", "80", "", "100")  # Show labels every 20
  ) +
  
  # Apply theme modifications
  theme_minimal() +
  theme(
    axis.text.x = element_text(size = 20, family = "Times New Roman", face = "bold"),
    axis.text.y = element_text(size = 20, family = "Times New Roman", face = "bold"),
    axis.title = element_text(size = 20, family = "Times New Roman", face = "bold"),
    legend.text = element_text(size = 15, family = "Times New Roman", face = "bold"),
    legend.title = element_text(size = 15, family = "Times New Roman", face = "bold"),
    legend.position = c(0.75, 0.8), 
    panel.background = element_blank(),
    plot.background = element_blank(),
    panel.grid.major = element_blank(),
    panel.grid.minor = element_blank(),
    panel.border = element_rect(color = "black", fill = NA)
  )

# Fit LOESS model
loess_model <- loess(mean_cap ~ as.numeric(condition), data = all_results_summarized, span = 0.75)

# Calculate RMSE for LOESS model
loess_predictions <- predict(loess_model, newdata = all_results_summarized)
loess_residuals <- all_results_summarized$mean_cap - loess_predictions
loess_rmse <- sqrt(mean(loess_residuals^2))
loess_range <- max(all_results_summarized$mean_cap) - min(all_results_summarized$mean_cap)
loess_rmse_percentage <- (loess_rmse / loess_range) * 100

# Extract RMSE for linear model
lm_rmse_percentage <- temp_models_cap$rmse_percentage[temp_models_cap$Temperature == "25C"]

# Create RMSE comparison table
rmse_comparison <- data.frame(
  Model = c("Linear", "LOESS"),
  RMSE = c(paste0(round(lm_rmse_percentage, 3), "%"), paste0(round(loess_rmse_percentage, 3), "%"))
)
#colnames(rmse_comparison) <- c("Model", "RMSE (%)")

# Create table grob
table_grob <- tableGrob(
  rmse_comparison,
  rows = NULL,
  theme = ttheme_minimal(
    core = list(bg_params = list(col = "black", lwd = 1),
                fg_params = list(fontface = "bold", fontsize = 12)),
    colhead = list(bg_params = list(col = "black", lwd = 1),
                   fg_params = list(fontface = "bold", fontsize = 12))
  )
)


# Annotate table in the plot
p <- p +
  annotation_custom(
    grob = table_grob,
    xmin =3,  # Position table near the right edge
    xmax = 4,  # Slightly left of the rightmost edge
    ymin = 145,  # Slightly below the top
    ymax =150                                      # Align to the top edge
  )


# Save the updated plot
ggsave("RMSE_cap_E.png", plot = p, width = 4, height = 6)

#_______________________Metanol_______________________-
# Function to Process Data for a Given Temperature
process_data <- function(data, temperature) {
  
  # Replace "-run" with "_run" in the Condition column to ensure uniformity
  data <- data %>%
    mutate(Condition = str_replace(Condition, "-run", "_run"))
  
  unique_conditions <- unique(data$Condition)
  
  split_conditions <- data.frame(
    condition = sapply(strsplit(unique_conditions, "_"), `[`, 1),
    run_name = sapply(strsplit(unique_conditions, "_"), `[`, 2)
  )
  
  
  results <- split_conditions %>%
    mutate(timeline_points = map2(condition, run_name, ~get_timeline_points(data, .x, .y)))
  
  final_results <- results %>%
    rowwise() %>%
    mutate(
      before_flat = list(timeline_points$before_flat),
      flat = list(timeline_points$flat),
      after_flat = list(timeline_points$after_flat),
      delta_t1 = timeline_points$delta_t1,
      delta_t2 = timeline_points$delta_t2,
      delta_t3 = timeline_points$delta_t3,
      TOE = timeline_points$TOE
    ) %>%
    select(-timeline_points) %>%
    ungroup()
  
  final_results <- final_results %>%
    mutate(
      before_flat = sapply(before_flat, function(x) toString(x$Time)),
      flat = sapply(flat, function(x) toString(x$Time)),
      after_flat = sapply(after_flat, function(x) toString(x$Time))
    )
  
  # Save the final results as an Excel file
  write_xlsx(final_results, paste0("timeline_results__Methanol_water", temperature, ".xlsx"))
  mean_sd_combinations_table <- final_results %>%
    group_by(condition) %>%
    do(calculate_mean_sd_combinations(.)) %>%
    ungroup()
  
  # Find the combination with the smallest combined SD for delta_t1, delta_t2, delta_t3, and TOE
  best_combinations_table <- mean_sd_combinations_table %>%
    group_by(condition) %>%
    mutate(total_sd = sd_delta_t1 + sd_delta_t2 + sd_delta_t3 + sd_toe) %>%
    slice_min(total_sd) %>%
    ungroup()
  
  # Apply the function to calculate mean Cap for each row of best_combinations_table
  best_combinations_table <- best_combinations_table %>%
    rowwise() %>%
    mutate(
      mean_cap = calculate_mean_cap(data, as.character(condition), as.character(combination))$mean_cap,
      sd_cap = calculate_mean_cap(data, as.character(condition), as.character(combination))$sd_cap
    ) %>%
    ungroup()
  
  # Define the correct order of conditions
  condition_order <- c(
    "Pure Water", "10% Methanol", "20% Methanol", "30% Methanol",
    "40% Methanol", "50% Methanol", "60% Methanol", "70% Methanol",
    "80% Methanol", "90% Methanol", "Pure Methanol"
  )
  
  # Convert the condition column to a factor with the specified levels
  best_combinations_table$condition <- factor(best_combinations_table$condition, levels = condition_order)
  
  # Arrange the data frame based on the factor levels
  best_combinations_table <- best_combinations_table %>%
    arrange(condition)
  
  # Save the tables to Excel
  write_xlsx(list(
    Mean_SD_Combinations = mean_sd_combinations_table,
    Best_Combinations = best_combinations_table
  ), paste0("mean_sd_combinations_and_selected_runs_Methanol_water", temperature, ".xlsx"))
  
  return(best_combinations_table)
}
# Process for each temperature
data_25C <- read.xlsx("reshape_Water-Methanol.xlsx", sheet = "25C", colNames = TRUE)
result_25C <- process_data(data_25C, "25C")
##########R2################
condition_order <- c(
  "Pure Water", "10% Methanol", "20% Methanol", "30% Methanol",
  "40% Methanol", "50% Methanol", "60% Methanol", "70% Methanol",
  "80% Methanol", "90% Methanol", "Pure Methanol"
)

combined_results <- bind_rows(
  result_25C %>% mutate(temperature = "25C")
)

plot_cubic_models_25C <- function(data) {
  # Filter data for 25C temperature
  data_25C <- data %>% filter(temperature == "25C")
  
  # Function to calculate accuracy metrics (R², RMSE, etc.)
  calculate_metrics <- function(model, data, y_var) {
    predictions <- predict(model, data)
    residuals <- data[[y_var]] - predictions
    r_squared <- 1 - sum(residuals^2) / sum((data[[y_var]] - mean(data[[y_var]]))^2)  # Manually calculate R²
    rmse <- sqrt(mean(residuals^2))
    mae <- mean(abs(residuals))
    range_y <- max(data[[y_var]]) - min(data[[y_var]])
    rmse_percentage <- (rmse / range_y) * 100
    
    return(list(r_squared = r_squared, rmse = rmse, mae = mae, rmse_percentage = rmse_percentage))
  }
  
  # Fit cubic and LOESS models and calculate metrics for each Delta
  lm_delta_t1 <- lm(mean_delta_t1 ~ as.numeric(condition), data = data_25C)
  loess_delta_t1 <- loess(mean_delta_t1 ~ as.numeric(condition), data = data_25C, span = 0.75)
  metrics_delta_t1_lm <- calculate_metrics(lm_delta_t1, data_25C, "mean_delta_t1")
  metrics_delta_t1_loess <- calculate_metrics(loess_delta_t1, data_25C, "mean_delta_t1")
  
  lm_delta_t2 <- lm(mean_delta_t2 ~ as.numeric(condition), data = data_25C)
  loess_delta_t2 <- loess(mean_delta_t2 ~ as.numeric(condition), data = data_25C, span = 0.75)
  metrics_delta_t2_lm <- calculate_metrics(lm_delta_t2, data_25C, "mean_delta_t2")
  metrics_delta_t2_loess <- calculate_metrics(loess_delta_t2, data_25C, "mean_delta_t2")
  
  lm_delta_t3 <- lm(mean_delta_t3 ~ as.numeric(condition), data = data_25C)
  loess_delta_t3 <- loess(mean_delta_t3 ~ as.numeric(condition), data = data_25C, span = 0.75)
  metrics_delta_t3_lm <- calculate_metrics(lm_delta_t3, data_25C, "mean_delta_t3")
  metrics_delta_t3_loess <- calculate_metrics(loess_delta_t3, data_25C, "mean_delta_t3")
  
  lm_toe <- lm(mean_toe ~ as.numeric(condition), data = data_25C)
  loess_toe <- loess(mean_toe ~ as.numeric(condition), data = data_25C, span = 0.75)
  metrics_toe_lm <- calculate_metrics(lm_toe, data_25C, "mean_toe")
  metrics_toe_loess <- calculate_metrics(loess_toe, data_25C, "mean_toe")
  
  # Function to create plots with LOESS and linear model
  create_combined_plot <- function(data, y_var, y_label, lm_model, loess_model, metrics_lm, metrics_loess, color) {
    min_value <- min(data[[y_var]], na.rm = TRUE)
    max_value <- max(data[[y_var]], na.rm = TRUE)
    annotation_x <- max(as.numeric(data$condition)) - 1  # Adjust annotation position
    
    values <- if (y_var == "mean_delta_t1") {
      list(
        max_value = max_value - (max_value - min_value) * 0.1,  # Default for mean_delta_t1
        legend = c(0.2, 0.8)  # Legend position for mean_delta_t1
      )
    } else {
      list(
        max_value = max_value - (max_value - min_value) * 0.05,  # Move up slightly for other conditions
        legend = c(0.2, 0.25)  # Legend position for other conditions
      )
    }
    
    # Access the calculated values
    max_value <- values$max_value
    legend_position <- values$legend
    
    
    # Table for R² metrics
    table_data <- data.frame(
      Model = c("Linear", "LOESS"),
      R2 = c(round(metrics_lm$r_squared, 3), round(metrics_loess$r_squared, 3))
    )
    
    table_grob <- tableGrob(
      table_data,
      rows = NULL,
      theme = ttheme_minimal(
        core = list(bg_params = list(col = "black", lwd = 1),
                    fg_params = list(fontface = "bold", fontsize = 12)),
        colhead = list(bg_params = list(col = "black", lwd = 1),
                       fg_params = list(fontface = "bold", fontsize = 12))
      )
    )
    ggplot(data, aes(x = as.numeric(condition), y = !!sym(y_var))) +
      # Points for "25C"
      geom_point(aes(color = "25C"), size = 4, show.legend = TRUE) +
      
      # Linear model (solid line)
      # Linear model (solid line)
      geom_smooth(method = "lm", formula = y ~ x, se = FALSE,
                  aes(linetype = "Linear"), color = color, size = 1) +
      
      # LOESS model (dashed line)
      geom_line(aes(y = predict(loess_model, data), linetype = "LOESS"),
                color = color, size = 1.8) +
      
      # Manual legends and scales
      labs(
        x = "Methanol (%)",
        y = y_label,
        color = "Condition",  # Legends for points
        linetype = "Model"    # Legends for lines
      ) +
      scale_linetype_manual(values = c("Linear" = "solid", "LOESS" = "dashed")) +
      scale_color_manual(values = c("25C" = "#B8860B")) +
      
      # Fix the legend guides
      guides(
        color = guide_legend(override.aes = list(linetype = "blank", shape = 16, size = 4)),  # Ensure points are circles
        linetype = guide_legend(override.aes = list(color = color, size = c(0.8, 1.5)))              # Ensure lines are lines
      ) +
      
      # Adjust the theme and legend
      theme_minimal() +
      theme(
        axis.text.x = element_text(size = 20, family = "Times New Roman", face = "bold"),
        axis.text.y = element_text(size = 20, family = "Times New Roman", face = "bold"),
        axis.title = element_text(size = 20, family = "Times New Roman", face = "bold"),
        legend.text = element_text(size = 15, family = "Times New Roman", face = "bold"),
        legend.title =  element_text(size = 15, family = "Times New Roman", face = "bold"),
        legend.position = legend_position, 
        legend.direction = "vertical",
        legend.spacing.y = unit(0.002, 'cm'),
        #legend.key.size = unit(0.8, 'lines'),
        panel.background = element_blank(),
        plot.background = element_blank(),
        panel.grid.major = element_blank(),
        panel.grid.minor = element_blank(),
        panel.border = element_rect(color = "black", fill = NA)
      )+
      annotation_custom(
        table_grob,
        xmin = max(as.numeric(data$condition)) - 3,  # Adjust to position in top-right corner
        xmax = max(as.numeric(data$condition))-1.5,      # Align to the right edge
        ymin = max_value - (max_value - min_value) * 0.1,  # Slightly below the top
        ymax = max_value                              # Align to the top edge
      ) 
    
    
  }
  
  # Combined plots for each metric
  p1 <- create_combined_plot(data_25C, "mean_delta_t1", expression(bold(Delta~t[1]~"(s)")), lm_delta_t1, loess_delta_t1, metrics_delta_t1_lm, metrics_delta_t1_loess, "navyblue")
  p2 <- create_combined_plot(data_25C, "mean_delta_t2", expression(bold(Delta~t[2]~"(s)")), lm_delta_t2, loess_delta_t2, metrics_delta_t2_lm, metrics_delta_t2_loess, "navyblue")
  p3 <- create_combined_plot(data_25C, "mean_delta_t3", expression(bold(Delta~t[3]~"(s)")), lm_delta_t3, loess_delta_t3, metrics_delta_t3_lm, metrics_delta_t3_loess, "navyblue")
  p4 <- create_combined_plot(data_25C, "mean_toe", "TOE (s)", lm_toe, loess_toe, metrics_toe_lm, metrics_toe_loess, "navyblue")
  
  plot_grid <- arrangeGrob(p1, p2, p3, p4, ncol = 4)
  ggsave("R2M.png", plot = plot_grid, width = 16, height = 6)
}


combined_results$condition <- factor(combined_results$condition, levels = condition_order)


plot_cubic_models_25C(combined_results)
############rmse_cap#############
###################

all_results <- bind_rows(
  result_25C %>% mutate(Temperature = "25C")
)


# Now convert the 'Condition' column to a factor with the specified levels
all_results$condition <- factor(all_results$condition, levels = condition_order)
all_results_summarized <- all_results %>%
  group_by(condition, Temperature) %>%
  summarize(
    mean_cap = mean(mean_cap),
    sd_cap = mean(sd_cap),
    .groups = 'drop'  # Ensures we drop the grouping after summarizing
  )


# Fit separate models for 25C and 40C using cubic polynomial fit for mean_delta__cap
temp_models_cap <- all_results_summarized %>%
  group_by(Temperature) %>%
  nest() %>%
  mutate(model = map(data, ~ lm(mean_cap ~ as.numeric(condition), data = .x)))  # Linear model

# Calculate accuracy metrics for mean_delta__cap
temp_models_cap <- temp_models_cap %>%
  mutate(
    predictions = map(model, ~ augment(.x)),  # Get predictions
    r_squared = map_dbl(model, ~ glance(.x)$r.squared),  # R-squared
    range_y = map_dbl(data, ~ max(.x$mean_cap) - min(.x$mean_cap)),  # Range of the observed values
    rmse = map_dbl(model, ~ sqrt(mean(residuals(.x)^2))),  # Root Mean Squared Error
    mae = map_dbl(model, ~ mean(abs(residuals(.x))))  # Mean Absolute Error
  ) %>%
  mutate(
    rmse_percentage = (rmse / range_y) * 100,  # RMSE as percentage of the range
    mae_percentage = (mae / range_y) * 100  # MAE as percentage of the range
  )

# Print accuracy metrics for each Temperature for mean_cap
temp_models_cap %>%
  select(Temperature, r_squared, rmse, mae, rmse_percentage, mae_percentage)
```

```
## # A tibble: 1 × 6
## # Groups:   Temperature [1]
##   Temperature r_squared  rmse   mae rmse_percentage mae_percentage
##   <chr>           <dbl> <dbl> <dbl>           <dbl>          <dbl>
## 1 25C             0.374  4.58  3.65            21.3           16.9
```

```
min_value_cap <- min(all_results_summarized$mean_cap, na.rm = TRUE)
max_value_cap <- max(all_results_summarized$mean_cap, na.rm = TRUE)

max_value_cap = max_value_cap +5

custom_colors <- c("25C" = "navyblue")

p <- ggplot(all_results_summarized, aes(x = condition, y = mean_cap, group = Temperature)) +
  # Points with a fixed color (#B8860B) and a legend
  geom_point(aes(color = "25C"), size = 4, show.legend = TRUE) +
  
  # Linear fit with solid navy blue line
  geom_smooth(aes(linetype = "Linear"), method = "lm", se = FALSE, size = 1, color = "navyblue") +
  
  # LOESS fit with dashed navy blue line
  geom_smooth(aes(linetype = "LOESS"), method = "loess", se = FALSE, size = 1.8, color = "navyblue") +
  
  labs(
    x = "Methanol (%)",
    y = expression(bold(Mean~Delta~Cap~(fF))),
    color = "Condition",  # Title for points legend
    linetype = "Model"    # Title for lines legend
  ) +
  
  # Adjust the legend aesthetics
  guides(
    color = guide_legend(
      override.aes = list(
        shape = 16,       # Circle shape for points
        size = 4,         # Size of points
        color = "#B8860B" # Fixed color for points
      )
    ),
    linetype = guide_legend(
      override.aes = list(
        color = "navyblue",    # Navy blue legend lines
        size = c(0.8, 1.5)     # Thin solid line and thicker dashed line
      )
    )
  ) +
  
  # Apply custom scales for linetypes
  scale_linetype_manual(values = c("Linear" = "solid", "LOESS" = "dashed")) +
  
  # Apply custom scales for the points legend
  scale_color_manual(values = c("25C" = "#B8860B")) +
  
  # Customize x-axis labels
  scale_x_discrete(
    limits = condition_order,
    labels = c("0", "", "20", "", "40", "", "60", "", "80", "", "100")  # Show labels every 20
  ) +
  
  # Apply theme modifications
  theme_minimal() +
  theme(
    axis.text.x = element_text(size = 20, family = "Times New Roman", face = "bold"),
    axis.text.y = element_text(size = 20, family = "Times New Roman", face = "bold"),
    axis.title = element_text(size = 20, family = "Times New Roman", face = "bold"),
    legend.text = element_text(size = 15, family = "Times New Roman", face = "bold"),
    legend.title = element_text(size = 15, family = "Times New Roman", face = "bold"),
    legend.position = c(0.75, 0.8), 
    panel.background = element_blank(),
    plot.background = element_blank(),
    panel.grid.major = element_blank(),
    panel.grid.minor = element_blank(),
    panel.border = element_rect(color = "black", fill = NA)
  )

# Fit LOESS model
loess_model <- loess(mean_cap ~ as.numeric(condition), data = all_results_summarized, span = 0.75)

# Calculate RMSE for LOESS model
loess_predictions <- predict(loess_model, newdata = all_results_summarized)
loess_residuals <- all_results_summarized$mean_cap - loess_predictions
loess_rmse <- sqrt(mean(loess_residuals^2))
loess_range <- max(all_results_summarized$mean_cap) - min(all_results_summarized$mean_cap)
loess_rmse_percentage <- (loess_rmse / loess_range) * 100

# Extract RMSE for linear model
lm_rmse_percentage <- temp_models_cap$rmse_percentage[temp_models_cap$Temperature == "25C"]

# Create RMSE comparison table
rmse_comparison <- data.frame(
  Model = c("Linear", "LOESS"),
  RMSE = c(paste0(round(lm_rmse_percentage, 3), "%"), paste0(round(loess_rmse_percentage, 3), "%"))
)
#colnames(rmse_comparison) <- c("Model", "RMSE (%)")


# Create table grob
table_grob <- tableGrob(
  rmse_comparison,
  rows = NULL,
  theme = ttheme_minimal(
    core = list(bg_params = list(col = "black", lwd = 1),
                fg_params = list(fontface = "bold", fontsize = 12)),
    colhead = list(bg_params = list(col = "black", lwd = 1),
                   fg_params = list(fontface = "bold", fontsize = 12))
  )
)

# Annotate table in the plot
p <- p +
  annotation_custom(
    grob = table_grob,
    xmin =3,  # Position table near the right edge
    xmax = 4,  # Slightly left of the rightmost edge
    ymin = 148,  # Slightly below the top
    ymax =150                                       # Align to the top edge
  )

ggsave("RMSE_cap_M.png", plot = p, width =4, height = 6)

#___________________M_E____________________
# Function to Process Data for a Given Temperature
process_data <- function(data, temperature) {
  
  # Replace "-run" with "_run" in the Condition column to ensure uniformity
  data <- data %>%
    mutate(Condition = str_replace(Condition, "-run", "_run"))
  
  unique_conditions <- unique(data$Condition)
  
  split_conditions <- data.frame(
    condition = sapply(strsplit(unique_conditions, "_"), `[`, 1),
    run_name = sapply(strsplit(unique_conditions, "_"), `[`, 2)
  )
  
  
  results <- split_conditions %>%
    mutate(timeline_points = map2(condition, run_name, ~get_timeline_points(data, .x, .y)))
  
  final_results <- results %>%
    rowwise() %>%
    mutate(
      before_flat = list(timeline_points$before_flat),
      flat = list(timeline_points$flat),
      after_flat = list(timeline_points$after_flat),
      delta_t1 = timeline_points$delta_t1,
      delta_t2 = timeline_points$delta_t2,
      delta_t3 = timeline_points$delta_t3,
      TOE = timeline_points$TOE
    ) %>%
    select(-timeline_points) %>%
    ungroup()
  
  final_results <- final_results %>%
    mutate(
      before_flat = sapply(before_flat, function(x) toString(x$Time)),
      flat = sapply(flat, function(x) toString(x$Time)),
      after_flat = sapply(after_flat, function(x) toString(x$Time))
    )
  
  # Save the final results as an Excel file
  write_xlsx(final_results, paste0("timeline_results__Methanol_Ethanol", temperature, ".xlsx"))
  mean_sd_combinations_table <- final_results %>%
    group_by(condition) %>%
    do(calculate_mean_sd_combinations(.)) %>%
    ungroup()
  
  # Find the combination with the smallest combined SD for delta_t1, delta_t2, delta_t3, and TOE
  best_combinations_table <- mean_sd_combinations_table %>%
    group_by(condition) %>%
    mutate(total_sd = sd_delta_t1 + sd_delta_t2 + sd_delta_t3 + sd_toe) %>%
    slice_min(total_sd) %>%
    ungroup()
  
  # Apply the function to calculate mean Cap for each row of best_combinations_table
  best_combinations_table <- best_combinations_table %>%
    rowwise() %>%
    mutate(
      mean_cap = calculate_mean_cap(data, as.character(condition), as.character(combination))$mean_cap,
      sd_cap = calculate_mean_cap(data, as.character(condition), as.character(combination))$sd_cap
    ) %>%
    ungroup()
  
  # Define the correct order of conditions
  condition_order <- c(
    "0% Methanol", "10% Methanol", "20% Methanol", "30% Methanol",
    "40% Methanol", "50% Methanol", "60% Methanol", "70% Methanol",
    "80% Methanol", "90% Methanol", "Pure Methanol"
  )
  
  # Convert the condition column to a factor with the specified levels
  best_combinations_table$condition <- factor(best_combinations_table$condition, levels = condition_order)
  
  # Arrange the data frame based on the factor levels
  best_combinations_table <- best_combinations_table %>%
    arrange(condition)
  
  # Save the tables to Excel
  write_xlsx(list(
    Mean_SD_Combinations = mean_sd_combinations_table,
    Best_Combinations = best_combinations_table
  ), paste0("mean_sd_combinations_and_selected_runs_Methanol_Ethanol", temperature, ".xlsx"))
  
  return(best_combinations_table)
}
# Process for each temperature
data_25C <- read.xlsx("reshape_Methanol-Ethanol.xlsx", sheet = "25C", colNames = TRUE)
result_25C <- process_data(data_25C, "25C")


##############R2##################
combined_results <- bind_rows(
  result_25C %>% mutate(temperature = "25C")
)

all_results_summarized <- combined_results %>%
  group_by(condition, temperature) %>%
  summarize(
    mean_cap = mean(mean_cap),
    sd_cap = mean(sd_cap),
    .groups = 'drop'  # Ensures we drop the grouping after summarizing
  )


min_value_cap <- min(all_results_summarized$mean_cap, na.rm = TRUE)
max_value_cap <- max(all_results_summarized$mean_cap, na.rm = TRUE)


plot_cubic_models_25C <- function(data) {
  # Filter data for 25C temperature
  data_25C <- data %>% filter(temperature == "25C")
  
  # Function to calculate accuracy metrics (R², RMSE, etc.)
  calculate_metrics <- function(model, data, y_var) {
    predictions <- predict(model, data)
    residuals <- data[[y_var]] - predictions
    r_squared <- 1 - sum(residuals^2) / sum((data[[y_var]] - mean(data[[y_var]]))^2)  # Manually calculate R²
    rmse <- sqrt(mean(residuals^2))
    mae <- mean(abs(residuals))
    range_y <- max(data[[y_var]]) - min(data[[y_var]])
    rmse_percentage <- (rmse / range_y) * 100
    
    return(list(r_squared = r_squared, rmse = rmse, mae = mae, rmse_percentage = rmse_percentage))
  }
  
  # Fit cubic and LOESS models and calculate metrics for each Delta
  lm_delta_t1 <- lm(mean_delta_t1 ~ as.numeric(condition), data = data_25C)
  loess_delta_t1 <- loess(mean_delta_t1 ~ as.numeric(condition), data = data_25C, span = 0.75)
  metrics_delta_t1_lm <- calculate_metrics(lm_delta_t1, data_25C, "mean_delta_t1")
  metrics_delta_t1_loess <- calculate_metrics(loess_delta_t1, data_25C, "mean_delta_t1")
  
  lm_delta_t2 <- lm(mean_delta_t2 ~ as.numeric(condition), data = data_25C)
  loess_delta_t2 <- loess(mean_delta_t2 ~ as.numeric(condition), data = data_25C, span = 0.75)
  metrics_delta_t2_lm <- calculate_metrics(lm_delta_t2, data_25C, "mean_delta_t2")
  metrics_delta_t2_loess <- calculate_metrics(loess_delta_t2, data_25C, "mean_delta_t2")
  
  lm_delta_t3 <- lm(mean_delta_t3 ~ as.numeric(condition), data = data_25C)
  loess_delta_t3 <- loess(mean_delta_t3 ~ as.numeric(condition), data = data_25C, span = 0.75)
  metrics_delta_t3_lm <- calculate_metrics(lm_delta_t3, data_25C, "mean_delta_t3")
  metrics_delta_t3_loess <- calculate_metrics(loess_delta_t3, data_25C, "mean_delta_t3")
  
  lm_toe <- lm(mean_toe ~ as.numeric(condition), data = data_25C)
  loess_toe <- loess(mean_toe ~ as.numeric(condition), data = data_25C, span = 0.75)
  metrics_toe_lm <- calculate_metrics(lm_toe, data_25C, "mean_toe")
  metrics_toe_loess <- calculate_metrics(loess_toe, data_25C, "mean_toe")
  
  # Function to create plots with LOESS and linear model
  create_combined_plot <- function(data, y_var, y_label, lm_model, loess_model, metrics_lm, metrics_loess, color) {
    min_value <- min(data[[y_var]], na.rm = TRUE)
    max_value <- max(data[[y_var]], na.rm = TRUE)
    annotation_x <- max(as.numeric(data$condition)) - 1  # Adjust annotation position
    
    values <- if (y_var == "mean_delta_t1") {
      list(
        max_value = max_value - (max_value - min_value) * 0.1,  # Default for mean_delta_t1
        legend = c(0.2, 0.8)  # Legend position for mean_delta_t1
      )
    } else {
      list(
        max_value = max_value - (max_value - min_value) * 0.05,  # Move up slightly for other conditions
        legend = c(0.8, 0.6)  # Legend position for other conditions
      )
    }
    
    # Access the calculated values
    max_value <- values$max_value
    legend_position <- values$legend
    
    
    # Table for R² metrics
    table_data <- data.frame(
      Model = c("Linear", "LOESS"),
      R2 = c(round(metrics_lm$r_squared, 3), round(metrics_loess$r_squared, 3))
    )
    
    table_grob <- tableGrob(
      table_data,
      rows = NULL,
      theme = ttheme_minimal(
        core = list(bg_params = list(col = "black", lwd = 1),
                    fg_params = list(fontface = "bold", fontsize = 12)),
        colhead = list(bg_params = list(col = "black", lwd = 1),
                       fg_params = list(fontface = "bold", fontsize = 12))
      )
    )
    ggplot(data, aes(x = as.numeric(condition), y = !!sym(y_var))) +
      # Points for "25C"
      geom_point(aes(color = "25C"), size = 4, show.legend = TRUE) +
      
      # Linear model (solid line)
      # Linear model (solid line)
      geom_smooth(method = "lm", formula = y ~ x, se = FALSE,
                  aes(linetype = "Linear"), color = color, size = 1) +
      
      # LOESS model (dashed line)
      geom_line(aes(y = predict(loess_model, data), linetype = "LOESS"),
                color = color, size = 1.8) +
      
      # Manual legends and scales
      labs(
        x = "Methanol (%)",
        y = y_label,
        color = "Condition",  # Legends for points
        linetype = "Model"    # Legends for lines
      ) +
      scale_linetype_manual(values = c("Linear" = "solid", "LOESS" = "dashed")) +
      scale_color_manual(values = c("25C" = "#B8860B")) +
      
      # Fix the legend guides
      guides(
        color = guide_legend(override.aes = list(linetype = "blank", shape = 16, size = 4)),  # Ensure points are circles
        linetype = guide_legend(override.aes = list(color = color, size = c(0.8, 1.5)))              # Ensure lines are lines
      ) +
      
      # Adjust the theme and legend
      theme_minimal() +
      theme(
        axis.text.x = element_text(size = 20, family = "Times New Roman", face = "bold"),
        axis.text.y = element_text(size = 20, family = "Times New Roman", face = "bold"),
        axis.title = element_text(size = 20, family = "Times New Roman", face = "bold"),
        legend.text = element_text(size = 15, family = "Times New Roman", face = "bold"),
        legend.title =  element_text(size = 15, family = "Times New Roman", face = "bold"),
        legend.position = legend_position, 
        legend.direction = "vertical",
        legend.spacing.y = unit(0.002, 'cm'),
        #legend.key.size = unit(0.8, 'lines'),
        panel.background = element_blank(),
        plot.background = element_blank(),
        panel.grid.major = element_blank(),
        panel.grid.minor = element_blank(),
        panel.border = element_rect(color = "black", fill = NA)
      )+
      annotation_custom(
        table_grob,
        xmin = max(as.numeric(data$condition)) - 3,  # Adjust to position in top-right corner
        xmax = max(as.numeric(data$condition))-1.5,      # Align to the right edge
        ymin = max_value - (max_value - min_value) * 0.1,  # Slightly below the top
        ymax = max_value                              # Align to the top edge
      ) 
    
    
  }
  
  # Combined plots for each metric
  p1 <- create_combined_plot(data_25C, "mean_delta_t1", expression(bold(Delta~t[1]~"(s)")), lm_delta_t1, loess_delta_t1, metrics_delta_t1_lm, metrics_delta_t1_loess, "navyblue")
  p2 <- create_combined_plot(data_25C, "mean_delta_t2", expression(bold(Delta~t[2]~"(s)")), lm_delta_t2, loess_delta_t2, metrics_delta_t2_lm, metrics_delta_t2_loess, "navyblue")
  p3 <- create_combined_plot(data_25C, "mean_delta_t3", expression(bold(Delta~t[3]~"(s)")), lm_delta_t3, loess_delta_t3, metrics_delta_t3_lm, metrics_delta_t3_loess, "navyblue")
  p4 <- create_combined_plot(data_25C, "mean_toe", "TOE (s)", lm_toe, loess_toe, metrics_toe_lm, metrics_toe_loess, "navyblue")
  
  plot_grid <- arrangeGrob(p1, p2, p3, p4, ncol = 4)
  ggsave("R2EM.png", plot = plot_grid, width = 16, height = 6)
}

condition_order <- c(
  "0% Methanol", "10% Methanol", "20% Methanol", "30% Methanol",
  "40% Methanol", "50% Methanol", "60% Methanol", "70% Methanol",
  "80% Methanol", "90% Methanol", "Pure Methanol"
)


combined_results$condition <- factor(combined_results$condition, levels = condition_order)


plot_cubic_models_25C(combined_results)

#######################RMSE_CAP###################
condition_order <- c(
  "0% Methanol", "10% Methanol", "20% Methanol", "30% Methanol",
  "40% Methanol", "50% Methanol", "60% Methanol", "70% Methanol",
  "80% Methanol", "90% Methanol", "Pure Methanol"
)

all_results <- bind_rows(
  result_25C %>% mutate(Temperature = "25C")
)


# Now convert the 'Condition' column to a factor with the specified levels
all_results$condition <- factor(all_results$condition, levels = condition_order)
all_results_summarized <- all_results %>%
  group_by(condition, Temperature) %>%
  summarize(
    mean_cap = mean(mean_cap),
    sd_cap = mean(sd_cap),
    .groups = 'drop'  # Ensures we drop the grouping after summarizing
  )


# Fit separate models for 25C and 40C using cubic polynomial fit for mean_delta__cap
temp_models_cap <- all_results_summarized %>%
  group_by(Temperature) %>%
  nest() %>%
  mutate(model = map(data, ~ lm(mean_cap ~ as.numeric(condition), data = .x)))  # Linear model

# Calculate accuracy metrics for mean_delta__cap
temp_models_cap <- temp_models_cap %>%
  mutate(
    predictions = map(model, ~ augment(.x)),  # Get predictions
    r_squared = map_dbl(model, ~ glance(.x)$r.squared),  # R-squared
    range_y = map_dbl(data, ~ max(.x$mean_cap) - min(.x$mean_cap)),  # Range of the observed values
    rmse = map_dbl(model, ~ sqrt(mean(residuals(.x)^2))),  # Root Mean Squared Error
    mae = map_dbl(model, ~ mean(abs(residuals(.x))))  # Mean Absolute Error
  ) %>%
  mutate(
    rmse_percentage = (rmse / range_y) * 100,  # RMSE as percentage of the range
    mae_percentage = (mae / range_y) * 100  # MAE as percentage of the range
  )

# Print accuracy metrics for each Temperature for mean_cap
temp_models_cap %>%
  select(Temperature, r_squared, rmse, mae, rmse_percentage, mae_percentage)
```

```
## # A tibble: 1 × 6
## # Groups:   Temperature [1]
##   Temperature r_squared  rmse   mae rmse_percentage mae_percentage
##   <chr>           <dbl> <dbl> <dbl>           <dbl>          <dbl>
## 1 25C             0.429  1.76  1.38            18.6           14.6
```

```
min_value_cap <- min(all_results_summarized$mean_cap, na.rm = TRUE)
max_value_cap <- max(all_results_summarized$mean_cap, na.rm = TRUE)

max_value_cap = max_value_cap +5

custom_colors <- c("25C" = "navyblue")

# Add LOESS model and compare RMSE with a table
p <- ggplot(all_results_summarized, aes(x = condition, y = mean_cap, group = Temperature)) +
  # Points with a fixed color (#B8860B) and a legend
  geom_point(aes(color = "25C"), size = 4, show.legend = TRUE) +
  
  # Linear fit with solid navy blue line
  geom_smooth(aes(linetype = "Linear"), method = "lm", se = FALSE, size = 1, color = "navyblue") +
  
  # LOESS fit with dashed navy blue line
  geom_smooth(aes(linetype = "LOESS"), method = "loess", se = FALSE, size = 1.8, color = "navyblue") +
  
  labs(
    x = "Methanol (%)",
    y = expression(bold(Mean~Delta~Cap~(fF))),
    color = "Condition",  # Title for points legend
    linetype = "Model"    # Title for lines legend
  ) +
  
  # Adjust the legend aesthetics
  guides(
    color = guide_legend(
      override.aes = list(
        shape = 16,       # Circle shape for points
        size = 4,         # Size of points
        color = "#B8860B" # Fixed color for points
      )
    ),
    linetype = guide_legend(
      override.aes = list(
        color = "navyblue",    # Navy blue legend lines
        size = c(0.8, 1.5)     # Thin solid line and thicker dashed line
      )
    )
  ) +
  
  # Apply custom scales for linetypes
  scale_linetype_manual(values = c("Linear" = "solid", "LOESS" = "dashed")) +
  
  # Apply custom scales for the points legend
  scale_color_manual(values = c("25C" = "#B8860B")) +
  
  # Customize x-axis labels
  scale_x_discrete(
    limits = condition_order,
    labels = c("0", "", "20", "", "40", "", "60", "", "80", "", "100")  # Show labels every 20
  ) +
  
  # Apply theme modifications
  theme_minimal() +
  theme(
    axis.text.x = element_text(size = 20, family = "Times New Roman", face = "bold"),
    axis.text.y = element_text(size = 20, family = "Times New Roman", face = "bold"),
    axis.title = element_text(size = 20, family = "Times New Roman", face = "bold"),
    legend.text = element_text(size = 15, family = "Times New Roman", face = "bold"),
    legend.title = element_text(size = 15, family = "Times New Roman", face = "bold"),
    legend.position = c(0.75, 0.8), 
    panel.background = element_blank(),
    plot.background = element_blank(),
    panel.grid.major = element_blank(),
    panel.grid.minor = element_blank(),
    panel.border = element_rect(color = "black", fill = NA)
  )

# Fit LOESS model
loess_model <- loess(mean_cap ~ as.numeric(condition), data = all_results_summarized, span = 0.75)

# Calculate RMSE for LOESS model
loess_predictions <- predict(loess_model, newdata = all_results_summarized)
loess_residuals <- all_results_summarized$mean_cap - loess_predictions
loess_rmse <- sqrt(mean(loess_residuals^2))
loess_range <- max(all_results_summarized$mean_cap) - min(all_results_summarized$mean_cap)
loess_rmse_percentage <- (loess_rmse / loess_range) * 100

# Extract RMSE for linear model
lm_rmse_percentage <- temp_models_cap$rmse_percentage[temp_models_cap$Temperature == "25C"]

# Create RMSE comparison table
rmse_comparison <- data.frame(
  Model = c("Linear", "LOESS"),
  RMSE = c(paste0(round(lm_rmse_percentage, 3), "%"), paste0(round(loess_rmse_percentage, 3), "%"))
)

#colnames(rmse_comparison) <- c("Model", "RMSE (%)")


table_grob <- tableGrob(
  rmse_comparison,
  rows = NULL,
  theme = ttheme_minimal(
    core = list(bg_params = list(col = "black", lwd = 1),
                fg_params = list(fontface = "bold", fontsize = 12)),
    colhead = list(bg_params = list(col = "black", lwd = 1),
                   fg_params = list(fontface = "bold", fontsize = 12))
  )
)


# Annotate table in the plot
p <- p +
  annotation_custom(
    grob = table_grob,
    xmin =3.5,  # Position table near the right edge
    xmax = 4,  # Slightly left of the rightmost edge
    ymin = 143,  # Slightly below the top
    ymax =145                                      # Align to the top edge
  )


# Save the plot
ggsave("RMSE_cap_EM.png", plot = p, width = 4, height = 6)


#_______________________-arrange
#___________________________________
library(magick)

# Load and resize the images
R2E <- image_read("R2E.png") %>% image_background("white") 
RMSE_cap_E <- image_read("RMSE_cap_E.png") %>% image_background("white") 

R2M <- image_read("R2M.png") %>% image_background("white") 
RMSE_cap_M <- image_read("RMSE_cap_M.png") %>% image_background("white")

R2EM <- image_read("R2EM.png") %>% image_background("white")
RMSE_cap_EM <- image_read("RMSE_cap_EM.png") %>% image_background("white")

# Create horizontal spacer
horizontal_spacer <- image_blank(width = 100, height = image_info(R2E)$height, color = "white")  # 50px wide blank space

# Arrange the first row with horizontal spacers: R2E, spacer, RMSE_cap_E, spacer, RMSE_t2_E
row1 <- image_append(c(R2E, horizontal_spacer, RMSE_cap_E))

# Arrange the second row with horizontal spacers: R2M, spacer, RMSE_cap_M, spacer, RMSE_t2_M
row2 <- image_append(c(R2M, horizontal_spacer, RMSE_cap_M))

# Arrange the third row with horizontal spacers: R2EM, spacer, RMSE_cap_EM, spacer, RMSE_t2_EM
row3 <- image_append(c(R2EM, horizontal_spacer, RMSE_cap_EM))

# Create vertical spacer for alignment
vertical_spacer <- image_blank(width = image_info(row1)$width, height = 200, color = "white")  # 200px height blank space

# Stack the rows vertically with spacers between them
final_image <- image_append(c(row1, vertical_spacer, row2, vertical_spacer, row3), stack = TRUE)

# Save the final combined image
image_write(final_image, "combined_plot2.png")
knitr::include_graphics("combined_plot2.png")
```
