# Supplementary material for: Complementary metal-oxide-semiconductor (CMOS) time of evaporation measurement system for binary chemical monitoring
Source: Sci Rep. 2026 Jan 23;16:5542. doi: 10.1038/s41598-026-35322-x (PMC12887047; doi:10.1038/s41598-026-35322-x)
Supplement: Supplementary file 3 — Supplementary Material 3 [file 41598_2026_35322_MOESM3_ESM.html]

Complementary Metal-Oxide-Semiconductor (CMOS) Time of Evaporation Measurement System for Binary Chemical Monitoring


# Complementary Metal-Oxide-Semiconductor (CMOS) Time of Evaporation Measurement System for Binary Chemical Monitoring

#### **Ebrahim Ghafar-Zadeh¹\***, **Saghi Forouhi²\***, Hamed Osouli Tabrizi¹, Abbas Panahi¹, Yasaman Tahernezhad¹, Azadeh Amrollahi Biyouki¹ ¹ Biologically Inspired Sensors and Actuators Laboratory (BioSA Lab), Department of Electrical Engineering and Computer Science, Lassonde School of Engineering, York University, Toronto, ON, M3J 1P3, Canada ² Division of Electronics and Computer Engineering (ELDA), Department of Electrical Engineering (ISY), Linköping University, 581 83 Linköping, Sweden

# 1. Ethanol\_water

## 1.1 Result

```
#__________________________
# cap-time plot
#_________________________
rm(list=ls())

#_______________________
# Libraries
#_______________________
library(nortest)
library(writexl)
library(tidyverse)
library(openxlsx)
library(dplyr)
library(Matrix)
library(lme4)
library(ggplot2)
library(car)
library(extrafont)
library(stringr)
library(gridExtra)
library(combinat)
library(tidyr)
library(cowplot)
library(ggpubr)
library(rlang)
library(broom)
library(purrr)
library(ggpmisc)  # For adding equation and R² to plot
# Import fonts
# font_import(prompt = FALSE)
loadfonts(device = "win")

#_______________________
# Function Definitions
#_______________________
get_sharpness_time <- function(data) {
  # Loop through the data until the cap value increases significantly
  for (i in 2:nrow(data)) {
    # Check if there's a notable increase in the Cap value after a steady range
    if (data$Cap[i] > 160) {
      return(data$Time[i])
    }
  }
  
  # Return NA if no sharpness time is found
  return(NA)
}


# Function to extract timeline points and calculate delta times
get_timeline_points <- function(data, condition, run_name) {
  # Filter data for the specified condition and run
  # Group and calculate cumulative time
  data <- data %>%
    group_by(Condition) %>%
    mutate(Cumulative_Time = cumsum(Time)) %>%
    ungroup() %>%
    mutate(Time = Cumulative_Time) %>%
    select(-Cumulative_Time)
  
  # Replace "-run" with "_run" in the Condition column to ensure uniformity
  data <- data %>%
    mutate(Condition = str_replace(Condition, "-run", "_run"))
  
  filtered_data <- data %>% filter(Condition == paste(condition, run_name, sep = "_"))
  
  # Get the sharpness time (steepest capacitance transition)
  sharpness_time <- get_sharpness_time(filtered_data)
  
  # Filter data based on the sharpness time
  data_before_sharpness <- filtered_data %>%
    filter(Time < sharpness_time)
  
  
  # Filter data based on the sharpness time
  data_after_sharpness <- filtered_data %>%
    filter(Time >= sharpness_time)
  
  # Define timeline points based on the sharpness
  max_cap_value <- max(data_after_sharpness$Cap, na.rm = TRUE)
  lower_bound <- max_cap_value - 5
  upper_bound <- max_cap_value + 5
  
  cap_range_data <- data_after_sharpness %>%
    filter(Cap >= lower_bound & Cap <= upper_bound)
  
  first_flat_time <- cap_range_data$Time[1]
  last_flat_time <- cap_range_data$Time[nrow(cap_range_data)]
  
  data_after_last_time <- data_after_sharpness %>%
    filter(Time > last_flat_time)
  
  delta_t1 <- max(data_before_sharpness$Time) - min(data_before_sharpness$Time)
  delta_t2 <- max(cap_range_data$Time) - min(cap_range_data$Time)
  delta_t3 <- max(data_after_last_time$Time) - min(data_after_last_time$Time)
  
  TOE <- max(filtered_data$Time)-min(filtered_data$Time)
  
  return(list(
    before_flat = data_before_sharpness,
    flat = cap_range_data,
    after_flat = data_after_last_time,
    delta_t1 = delta_t1,
    delta_t2 = delta_t2,
    delta_t3 = delta_t3,
    TOE = TOE
  ))
}

# Function to calculate Mean and SD of delta_t1, delta_t3, and TOE for each combination of three runs
calculate_mean_sd_combinations <- function(data) {
  condition <- unique(data$condition)
  
  # Generate all possible combinations of three runs
  run_combinations <- combn(data$run_name, 3, simplify = FALSE)
  
  # Calculate Mean and SD of delta_t1, delta_t3, and TOE for each combination
  mean_delta_t1 <- sapply(run_combinations, function(runs) {
    selected_delta_t1 <- data %>% filter(run_name %in% runs) %>% pull(delta_t1)
    mean(selected_delta_t1)
  })
  
  sd_delta_t1 <- sapply(run_combinations, function(runs) {
    selected_delta_t1 <- data %>% filter(run_name %in% runs) %>% pull(delta_t1)
    sd(selected_delta_t1)
  })
  
  mean_delta_t2 <- sapply(run_combinations, function(runs) {
    selected_delta_t2 <- data %>% filter(run_name %in% runs) %>% pull(delta_t2)
    mean(selected_delta_t2)
  })
  
  sd_delta_t2 <- sapply(run_combinations, function(runs) {
    selected_delta_t2 <- data %>% filter(run_name %in% runs) %>% pull(delta_t2)
    sd(selected_delta_t2)
  })
  
  mean_delta_t3 <- sapply(run_combinations, function(runs) {
    selected_delta_t3 <- data %>% filter(run_name %in% runs) %>% pull(delta_t3)
    mean(selected_delta_t3)
  })
  
  sd_delta_t3 <- sapply(run_combinations, function(runs) {
    selected_delta_t3 <- data %>% filter(run_name %in% runs) %>% pull(delta_t3)
    sd(selected_delta_t3)
  })
  
  mean_toe <- sapply(run_combinations, function(runs) {
    selected_toes <- data %>% filter(run_name %in% runs) %>% pull(TOE)
    mean(selected_toes)
  })
  
  sd_toe <- sapply(run_combinations, function(runs) {
    selected_toes <- data %>% filter(run_name %in% runs) %>% pull(TOE)
    sd(selected_toes)
  })
  
  # Create a data frame with the combinations, their Mean and SD for delta_t1, delta_t3, and TOE
  mean_sd_table <- data.frame(
    condition = condition,
    combination = sapply(run_combinations, paste, collapse = ", "),
    mean_delta_t1 = mean_delta_t1,
    sd_delta_t1 = sd_delta_t1,
    mean_delta_t2 = mean_delta_t2,
    sd_delta_t2 = sd_delta_t2,
    mean_delta_t3 = mean_delta_t3,
    sd_delta_t3 = sd_delta_t3,
    mean_toe = mean_toe,
    sd_toe = sd_toe
  )
  
  # Return the Mean and SD table
  return(mean_sd_table)
}

# Function to calculate mean Cap for the selected runs
calculate_mean_cap <- function(data, condition, combination) {
  # Extract the runs from the combination column
  selected_runs <- str_split(combination, ", ") %>% unlist()
  
  # Filter the data for the specific condition and selected runs
  filtered_data <- data %>%
    filter(str_detect(Condition, paste0(condition, "_(", paste0(selected_runs, collapse = "|"), ")")))
  
  # Calculate the mean Cap
  mean_cap <- mean(filtered_data$Cap, na.rm = TRUE)
  sd_cap <- sd(filtered_data$Cap, na.rm = TRUE)
  return(list(mean_cap = mean_cap, sd_cap = sd_cap))
}

# Function to Process Data for a Given Temperature
process_data <- function(data, temperature) {
  
  # Replace "-run" with "_run" in the Condition column to ensure uniformity
  data <- data %>%
    mutate(Condition = str_replace(Condition, "-run", "_run"))
  
  unique_conditions <- unique(data$Condition)
  
  split_conditions <- data.frame(
    condition = sapply(strsplit(unique_conditions, "_"), `[`, 1),
    run_name = sapply(strsplit(unique_conditions, "_"), `[`, 2)
  )
  
  
  results <- split_conditions %>%
    mutate(timeline_points = map2(condition, run_name, ~get_timeline_points(data, .x, .y)))
  
  final_results <- results %>%
    rowwise() %>%
    mutate(
      before_flat = list(timeline_points$before_flat),
      flat = list(timeline_points$flat),
      after_flat = list(timeline_points$after_flat),
      delta_t1 = timeline_points$delta_t1,
      delta_t2 = timeline_points$delta_t2,
      delta_t3 = timeline_points$delta_t3,
      TOE = timeline_points$TOE
    ) %>%
    select(-timeline_points) %>%
    ungroup()
  
  final_results <- final_results %>%
    mutate(
      before_flat = sapply(before_flat, function(x) toString(x$Time)),
      flat = sapply(flat, function(x) toString(x$Time)),
      after_flat = sapply(after_flat, function(x) toString(x$Time))
    )
  
  # Save the final results as an Excel file
  write_xlsx(final_results, paste0("timeline_results__ethanol_water", temperature, ".xlsx"))
  mean_sd_combinations_table <- final_results %>%
    group_by(condition) %>%
    do(calculate_mean_sd_combinations(.)) %>%
    ungroup()
  
  # Find the combination with the smallest combined SD for delta_t1, delta_t2, delta_t3, and TOE
  best_combinations_table <- mean_sd_combinations_table %>%
    group_by(condition) %>%
    mutate(total_sd = sd_delta_t1 + sd_delta_t2 + sd_delta_t3 + sd_toe) %>%
    slice_min(total_sd) %>%
    ungroup()
  
  # Apply the function to calculate mean Cap for each row of best_combinations_table
  best_combinations_table <- best_combinations_table %>%
    rowwise() %>%
    mutate(
      mean_cap = calculate_mean_cap(data, as.character(condition), as.character(combination))$mean_cap,
      sd_cap = calculate_mean_cap(data, as.character(condition), as.character(combination))$sd_cap
    ) %>%
    ungroup()
  
  # Define the correct order of conditions
  condition_order <- c(
    "Pure Water", "10% Ethanol", "20% Ethanol", "30% Ethanol",
    "40% Ethanol", "50% Ethanol", "60% Ethanol", "70% Ethanol",
    "80% Ethanol", "90% Ethanol", "Pure Ethanol"
  )
  
  # Convert the condition column to a factor with the specified levels
  best_combinations_table$condition <- factor(best_combinations_table$condition, levels = condition_order)
  
  # Arrange the data frame based on the factor levels
  best_combinations_table <- best_combinations_table %>%
    arrange(condition)
  
  # Save the tables to Excel
  write_xlsx(list(
    Mean_SD_Combinations = mean_sd_combinations_table,
    Best_Combinations = best_combinations_table
  ), paste0("mean_sd_combinations_and_selected_runs_ethanol_water", temperature, ".xlsx"))
  
  return(best_combinations_table)
}
# Process for each temperature
data_25C <- read.xlsx("reshape_Water-Ethanol.xlsx", sheet = "25C", colNames = TRUE)
result_25C <- process_data(data_25C, "25C")
summary(result_25C)
```

```
##        condition combination        mean_delta_t1    sd_delta_t1    
##  Pure Water :1   Length:11          Min.   :10.46   Min.   : 1.126  
##  10% Ethanol:1   Class :character   1st Qu.:11.85   1st Qu.: 3.032  
##  20% Ethanol:1   Mode  :character   Median :13.74   Median : 4.469  
##  30% Ethanol:1                      Mean   :13.97   Mean   : 5.432  
##  40% Ethanol:1                      3rd Qu.:15.89   3rd Qu.: 7.186  
##  50% Ethanol:1                      Max.   :18.63   Max.   :14.389  
##  (Other)    :5                                                      
##  mean_delta_t2     sd_delta_t2     mean_delta_t3    sd_delta_t3    
##  Min.   : 65.06   Min.   : 5.274   Min.   : 85.3   Min.   : 6.213  
##  1st Qu.:148.84   1st Qu.: 8.501   1st Qu.:117.7   1st Qu.:10.065  
##  Median :269.43   Median :12.494   Median :183.1   Median :14.399  
##  Mean   :248.88   Mean   :12.917   Mean   :171.1   Mean   :19.608  
##  3rd Qu.:336.82   3rd Qu.:15.372   3rd Qu.:217.1   3rd Qu.:30.600  
##  Max.   :425.61   Max.   :23.344   Max.   :247.1   Max.   :33.648  
##                                                                    
##     mean_toe         sd_toe          total_sd        mean_cap    
##  Min.   :174.9   Min.   : 1.769   Min.   :22.73   Min.   :146.5  
##  1st Qu.:287.7   1st Qu.: 6.860   1st Qu.:33.92   1st Qu.:153.9  
##  Median :498.0   Median :15.040   Median :57.81   Median :155.6  
##  Mean   :442.8   Mean   :17.197   Mean   :55.15   Mean   :156.3  
##  3rd Qu.:537.2   3rd Qu.:24.955   3rd Qu.:71.02   3rd Qu.:160.6  
##  Max.   :694.7   Max.   :41.615   Max.   :88.26   Max.   :162.0  
##                                                                  
##      sd_cap     
##  Min.   :19.23  
##  1st Qu.:20.10  
##  Median :20.30  
##  Mean   :20.55  
##  3rd Qu.:20.98  
##  Max.   :22.29  
##
```

```
data_40C <- read.xlsx("reshape_Water-Ethanol.xlsx", sheet = "40C", colNames = TRUE)
result_40C <- process_data(data_40C, "40C")
summary(result_40C)
```

```
##        condition combination        mean_delta_t1     sd_delta_t1    
##  Pure Water :1   Length:11          Min.   : 5.522   Min.   : 1.340  
##  10% Ethanol:1   Class :character   1st Qu.: 6.678   1st Qu.: 3.402  
##  20% Ethanol:1   Mode  :character   Median : 9.746   Median : 7.726  
##  30% Ethanol:1                      Mean   :11.670   Mean   : 7.681  
##  40% Ethanol:1                      3rd Qu.:12.799   3rd Qu.:10.840  
##  50% Ethanol:1                      Max.   :27.232   Max.   :18.006  
##  (Other)    :5                                                       
##  mean_delta_t2     sd_delta_t2     mean_delta_t3     sd_delta_t3    
##  Min.   : 25.43   Min.   : 2.231   Min.   : 29.01   Min.   : 1.637  
##  1st Qu.: 39.19   1st Qu.: 3.974   1st Qu.: 46.32   1st Qu.: 6.877  
##  Median :102.89   Median : 4.647   Median :100.90   Median :16.312  
##  Mean   :122.27   Mean   : 8.606   Mean   : 87.80   Mean   :18.053  
##  3rd Qu.:189.38   3rd Qu.: 6.467   3rd Qu.:121.91   3rd Qu.:23.215  
##  Max.   :256.31   Max.   :27.800   Max.   :149.86   Max.   :59.977  
##                                                                     
##     mean_toe          sd_toe          total_sd         mean_cap    
##  Min.   : 74.69   Min.   : 4.077   Min.   : 13.06   Min.   :144.3  
##  1st Qu.: 99.39   1st Qu.:10.707   1st Qu.: 25.07   1st Qu.:151.6  
##  Median :222.98   Median :24.326   Median : 58.32   Median :157.3  
##  Mean   :228.60   Mean   :22.488   Mean   : 56.83   Mean   :154.9  
##  3rd Qu.:330.77   3rd Qu.:29.580   3rd Qu.: 64.12   3rd Qu.:158.7  
##  Max.   :431.78   Max.   :50.859   Max.   :135.76   Max.   :160.0  
##                                                                    
##      sd_cap     
##  Min.   :20.11  
##  1st Qu.:21.39  
##  Median :21.61  
##  Mean   :21.87  
##  3rd Qu.:22.62  
##  Max.   :23.25  
##
```

```
data_50C <- read.xlsx("reshape_Water-Ethanol.xlsx", sheet = "50C", colNames = TRUE)
result_50C <- process_data(data_50C, "50C")
summary(result_50C)
```

```
##        condition combination        mean_delta_t1     sd_delta_t1    
##  Pure Water :1   Length:11          Min.   : 7.876   Min.   : 3.253  
##  10% Ethanol:1   Class :character   1st Qu.:11.065   1st Qu.: 3.992  
##  20% Ethanol:1   Mode  :character   Median :15.628   Median : 5.690  
##  30% Ethanol:1                      Mean   :15.123   Mean   : 6.913  
##  40% Ethanol:1                      3rd Qu.:18.870   3rd Qu.: 9.750  
##  50% Ethanol:1                      Max.   :22.424   Max.   :11.257  
##  (Other)    :5                                                       
##  mean_delta_t2     sd_delta_t2     mean_delta_t3    sd_delta_t3    
##  Min.   : 10.24   Min.   : 0.776   Min.   :15.82   Min.   : 1.672  
##  1st Qu.: 21.27   1st Qu.: 2.329   1st Qu.:28.83   1st Qu.: 9.855  
##  Median : 46.87   Median : 5.035   Median :53.47   Median :12.369  
##  Mean   : 56.47   Mean   : 4.956   Mean   :50.76   Mean   :16.980  
##  3rd Qu.: 88.50   3rd Qu.: 6.314   3rd Qu.:64.14   3rd Qu.:24.426  
##  Max.   :124.78   Max.   :10.806   Max.   :88.00   Max.   :37.735  
##                                                                    
##     mean_toe          sd_toe          total_sd         mean_cap    
##  Min.   : 39.17   Min.   : 2.366   Min.   : 14.78   Min.   :143.4  
##  1st Qu.: 67.02   1st Qu.: 9.739   1st Qu.: 30.90   1st Qu.:145.8  
##  Median :123.88   Median :14.973   Median : 39.76   Median :151.1  
##  Mean   :128.53   Mean   :21.001   Mean   : 49.85   Mean   :149.7  
##  3rd Qu.:201.45   3rd Qu.:30.650   3rd Qu.: 64.67   3rd Qu.:151.8  
##  Max.   :205.73   Max.   :47.271   Max.   :101.47   Max.   :159.2  
##                                                                    
##      sd_cap     
##  Min.   :20.85  
##  1st Qu.:22.65  
##  Median :22.99  
##  Mean   :22.66  
##  3rd Qu.:23.16  
##  Max.   :23.36  
##
```

```
data_60C <- read.xlsx("reshape_Water-Ethanol.xlsx", sheet = "60C", colNames = TRUE)
result_60C <- process_data(data_60C, "60C")
summary(result_60C)
```

```
##        condition combination        mean_delta_t1     sd_delta_t1   
##  Pure Water :1   Length:11          Min.   : 9.562   Min.   :1.805  
##  10% Ethanol:1   Class :character   1st Qu.:13.350   1st Qu.:2.457  
##  20% Ethanol:1   Mode  :character   Median :15.720   Median :3.970  
##  30% Ethanol:1                      Mean   :15.673   Mean   :4.532  
##  40% Ethanol:1                      3rd Qu.:17.375   3rd Qu.:6.659  
##  50% Ethanol:1                      Max.   :23.044   Max.   :8.442  
##  (Other)    :5                                                      
##  mean_delta_t2     sd_delta_t2      mean_delta_t3     sd_delta_t3     
##  Min.   : 7.142   Min.   : 0.9839   Min.   : 11.34   Min.   :  0.760  
##  1st Qu.:11.797   1st Qu.: 2.2123   1st Qu.: 19.91   1st Qu.:  3.005  
##  Median :26.204   Median : 3.2000   Median : 26.90   Median :  6.536  
##  Mean   :31.365   Mean   : 3.8892   Mean   : 32.76   Mean   : 18.218  
##  3rd Qu.:47.431   3rd Qu.: 4.5194   3rd Qu.: 32.70   3rd Qu.:  9.200  
##  Max.   :69.803   Max.   :10.9044   Max.   :102.67   Max.   :126.086  
##                                                                       
##     mean_toe          sd_toe           total_sd         mean_cap    
##  Min.   : 43.49   Min.   :  5.212   Min.   : 13.32   Min.   :136.5  
##  1st Qu.: 53.08   1st Qu.:  7.306   1st Qu.: 17.31   1st Qu.:141.3  
##  Median : 67.93   Median :  9.720   Median : 23.96   Median :145.5  
##  Mean   : 85.25   Mean   : 20.819   Mean   : 47.46   Mean   :146.1  
##  3rd Qu.:115.83   3rd Qu.: 16.257   3rd Qu.: 33.79   3rd Qu.:150.8  
##  Max.   :175.22   Max.   :118.690   Max.   :254.12   Max.   :156.1  
##                                                                     
##      sd_cap     
##  Min.   :17.87  
##  1st Qu.:21.59  
##  Median :22.68  
##  Mean   :22.22  
##  3rd Qu.:23.37  
##  Max.   :23.65  
##
```

## 1.2 FigureS1,S2,S3,S4

```
#________________________________
#line plot for for TOE, delta_t1, delta_t2, and delta_t3
#__________________________________
plot_results <- function(data, y_var, sd_var, y_label, output_filename) {
  p <- ggplot(data, aes(x = condition, y = !!sym(y_var), group = temperature, color = temperature)) +
    geom_line(size = 1.2) +
    geom_point(size = 3) +
    geom_errorbar(aes(ymin = !!sym(y_var) - !!sym(sd_var),
                      ymax = !!sym(y_var) + !!sym(sd_var)),
                  width = 0.2, size = 1) +
    labs(x = "Ethanol (%)", y = y_label, color = "Temperature") +
    theme_minimal() +
    theme(
      axis.text.x = element_text(size = 12, family = "Times New Roman",face="bold"),
      axis.text.y= element_text(size = 12, family = "Times New Roman",face="bold"),
      axis.title = element_text(size = 12, family = "Times New Roman",face="bold"),
      legend.text = element_text(size = 12, family = "Times New Roman",face="bold"),
      legend.title = element_text(size = 12, family = "Times New Roman",face="bold"),
      legend.position = c(0.8, 0.8), 
      legend.direction = "vertical",
      legend.spacing.y = unit(0.002, 'cm'),
      legend.key.size = unit(0.8, 'lines'),
      panel.background = element_blank(),
      plot.background = element_blank(),
      panel.grid.major = element_blank(),
      panel.grid.minor = element_blank(),
      panel.border = element_rect(color = "black", fill = NA)
    ) +
    scale_x_discrete(labels = c("0", "10", "20", "30", "40", "50", "60", "70", "80", "90", "100")) +
    scale_color_manual(values = c("25C" = "navyblue", "40C" = "darkgreen", "50C" = "#B8860B", "60C" = "darkred"))
  print(p)
  # Save the plot
  ggsave(output_filename, plot = p, width = 10, height = 6)
}

# Combine all results into one data frame
combined_results <- bind_rows(
  result_25C %>% mutate(temperature = "25C"),
  result_40C %>% mutate(temperature = "40C"),
  result_50C %>% mutate(temperature = "50C"),
  result_60C %>% mutate(temperature = "60C")
)

# Convert condition to a factor with the correct order
condition_order <- c(
  "Pure Water", "10% Ethanol", "20% Ethanol", "30% Ethanol",
  "40% Ethanol", "50% Ethanol", "60% Ethanol", "70% Ethanol",
  "80% Ethanol", "90% Ethanol", "Pure Ethanol"
)

combined_results$condition <- factor(combined_results$condition, levels = condition_order)

# Plot TOE
plot_results(combined_results, "mean_toe", "sd_toe", "TOE (s)", "TOE_conditions_temperatures.png")
```

```
# Plot delta_t1
plot_results(combined_results, "mean_delta_t1", "sd_delta_t1", expression(bold(Delta~t[1]~"(s)")), "delta_t1_conditions_temperatures.png")
```

```
# Plot delta_t2
plot_results(combined_results, "mean_delta_t2", "sd_delta_t2", expression(bold(Delta~t[2]~"(s)")), "delta_t2_conditions_temperatures.png")
```

```
# Plot delta_t3
plot_results(combined_results, "mean_delta_t3", "sd_delta_t3", expression(bold(Delta~t[3]~"(s)")), "delta_t3_conditions_temperatures.png")
```

## 1.3 Table1.1

```
data_25C <- read.xlsx("reshape_Water-Ethanol.xlsx", sheet = "25C", colNames = TRUE)
data_40C <- read.xlsx("reshape_Water-Ethanol.xlsx", sheet = "40C", colNames = TRUE)
data_50C <- read.xlsx("reshape_Water-Ethanol.xlsx", sheet = "50C", colNames = TRUE)
data_60C <- read.xlsx("reshape_Water-Ethanol.xlsx", sheet = "60C", colNames = TRUE)


df_25C <- read.xlsx("timeline_results__ethanol_water25C.xlsx", colNames = TRUE)
df_40C <- read.xlsx("timeline_results__ethanol_water40C.xlsx", colNames = TRUE)
df2_40C <- read.xlsx("mean_sd_combinations_and_selected_runs_ethanol_water40C.xlsx", sheet = "Best_Combinations", colNames = TRUE)
df2_25C <- read.xlsx("mean_sd_combinations_and_selected_runs_ethanol_water25C.xlsx", sheet = "Best_Combinations", colNames = TRUE)
df_50C <- read.xlsx("timeline_results__ethanol_water50C.xlsx", colNames = TRUE)
df2_50C <- read.xlsx("mean_sd_combinations_and_selected_runs_ethanol_water50C.xlsx", sheet = "Best_Combinations", colNames = TRUE)

df_60C <- read.xlsx("timeline_results__ethanol_water60C.xlsx", colNames = TRUE)
df2_60C <- read.xlsx("mean_sd_combinations_and_selected_runs_ethanol_water60C.xlsx", sheet = "Best_Combinations", colNames = TRUE)


#________________________
#Dynamic range
#_______________________
process_dynemic <- function(data, temp_label, df, df2) {
  
  data <- data %>%
    group_by(Condition) %>%
    mutate(Cumulative_Time = cumsum(Time)) %>%
    ungroup() %>%
    mutate(Time = Cumulative_Time) %>%
    select(-Cumulative_Time) %>%
    mutate(Condition = str_replace(Condition, "-run", "_run"))
  
  # Assuming 'df' is already loaded and structured properly
  df$condition <- factor(df$condition)
  
  # Preparing to selected data
  selected_data <- data.frame()
  
  # Process each condition from df2
  unique_conditions <- unique(df2$condition)
  
  # Iterate over each unique condition from df2 which matches conditions specified in df
  for (condition_to_match in unique(df2$condition)) {
    # Subset df2 for specific condition
    df2_subset <- df2 %>% filter(str_detect(condition, condition_to_match))
    selected_runs <- unlist(strsplit(as.character(df2_subset$combination[1]), ", "))
    run = selected_runs[1]  # Ensuring the run exists in our data columns
    
    # Filter the main data for the selected run and condition
    filtered_data <- data %>%
      filter(Condition == paste(condition_to_match, run, sep = "_"))
    
    # Get the flat time range from df which matches the current condition and run
    flat_times <- df %>% 
      filter(condition == condition_to_match & run_name == run) %>% 
      pull(flat) %>% 
      strsplit(split = ",") %>%
      unlist() %>%
      as.numeric()
    
    # Assuming the existence of a function to get the appropriate sharpness time
    sharpness_time <- get_sharpness_time(filtered_data) # Define or adjust this function based on our dataset
    
    # Filtering to include only times within the flat time range
    if (length(flat_times) > 0) {
      data_run <- filtered_data %>%
        filter(Time >= min(flat_times) & Time <= max(flat_times)) %>%
        mutate(Time_aligned = pmax(Time - sharpness_time, 0),
               Cap_aligned = Cap)
      
      selected_data <- bind_rows(selected_data, data_run %>% select(Time_aligned, Cap_aligned, Condition))
    }
  }
  
  # Compute dynamic range for Capacitance (Cap)
  cap_dynamic <- ((max(selected_data$Cap_aligned) - min(selected_data$Cap_aligned))/mean(selected_data$Cap_aligned))*100
  print(paste("Dynamic range for Capacitance in temp (%)",temp_label, ":", cap_dynamic))
  
  # Compute dynamic range for Time
  time_dynamic <- ((max(selected_data$Time_aligned) - min(selected_data$Time_aligned))/mean(selected_data$Time_aligned))*100
  print(paste("Dynamic range for Time in temp",temp_label, ":",time_dynamic))
}

process_dynemic(data_25C, "25C", df_25C, df2_25C)
```

```
## [1] "Dynamic range for Capacitance in temp (%) 25C : 4.98344967484264"
## [1] "Dynamic range for Time in temp 25C : 325.701062592723"
```

```
process_dynemic(data_40C, "40C", df_40C, df2_40C)
```

```
## [1] "Dynamic range for Capacitance in temp (%) 40C : 3.2837603924602"
## [1] "Dynamic range for Time in temp 40C : 342.483900655696"
```

```
process_dynemic(data_50C, "50C", df_50C, df2_50C)
```

```
## [1] "Dynamic range for Capacitance in temp (%) 50C : 3.7244063398951"
## [1] "Dynamic range for Time in temp 50C : 316.271044641888"
```

```
process_dynemic(data_60C, "60C", df_60C, df2_60C)
```

```
## [1] "Dynamic range for Capacitance in temp (%) 60C : 3.27854916030513"
## [1] "Dynamic range for Time in temp 60C : 379.169561823391"
```

## 1.4 Table1.2

```
# Load necessary libraries
# Load the data
df2_40C <- read.xlsx("mean_sd_combinations_and_selected_runs_ethanol_water40C.xlsx", sheet = "Best_Combinations")
df2_25C <- read.xlsx("mean_sd_combinations_and_selected_runs_ethanol_water25C.xlsx", sheet = "Best_Combinations")
df2_50C <- read.xlsx("mean_sd_combinations_and_selected_runs_ethanol_water50C.xlsx", sheet = "Best_Combinations")
df2_60C <- read.xlsx("mean_sd_combinations_and_selected_runs_ethanol_water60C.xlsx", sheet = "Best_Combinations")

# Extract the 'mean_delta_t2' column
delta_t2_25C <- df2_25C$mean_delta_t2
delta_t2_40C <- df2_40C$mean_delta_t2
delta_t2_50C <- df2_50C$mean_delta_t2
delta_t2_60C <- df2_60C$mean_delta_t2

# Normality test using Shapiro-Wilk test
shapiro_test_25C <- shapiro.test(delta_t2_25C)
shapiro_test_40C <- shapiro.test(delta_t2_40C)
shapiro_test_50C <- shapiro.test(delta_t2_50C)
shapiro_test_60C <- shapiro.test(delta_t2_60C)

# Display the results of the normality tests
print(shapiro_test_25C)
```

```
## 
##  Shapiro-Wilk normality test
## 
## data:  delta_t2_25C
## W = 0.94491, p-value = 0.5798
```

```
print(shapiro_test_40C)
```

```
## 
##  Shapiro-Wilk normality test
## 
## data:  delta_t2_40C
## W = 0.89189, p-value = 0.1468
```

```
print(shapiro_test_50C)
```

```
## 
##  Shapiro-Wilk normality test
## 
## data:  delta_t2_50C
## W = 0.91224, p-value = 0.2592
```

```
print(shapiro_test_60C)
```

```
## 
##  Shapiro-Wilk normality test
## 
## data:  delta_t2_60C
## W = 0.90547, p-value = 0.2153
```

```
# Equality of variances test using Levene's test
levene_test <- leveneTest(c(delta_t2_25C, delta_t2_40C, delta_t2_50C, delta_t2_60C), 
                          group = rep(c("25C", "40C", "50C", "60C"), times = c(length(delta_t2_25C), length(delta_t2_40C), length(delta_t2_50C), length(delta_t2_60C))))

# Display the result of the Levene's test
print(levene_test)
```

```
## Levene's Test for Homogeneity of Variance (center = median)
##       Df F value    Pr(>F)    
## group  3  9.2238 9.208e-05 ***
##       40                      
## ---
## Signif. codes:  0 '***' 0.001 '**' 0.01 '*' 0.05 '.' 0.1 ' ' 1
```

## 1.5 FigureS5

```
#_________________________________
#barplot for cap
#_________________________
condition_order <- c(
  "Pure Water", "10% Ethanol", "20% Ethanol", "30% Ethanol",
  "40% Ethanol", "50% Ethanol", "60% Ethanol", "70% Ethanol",
  "80% Ethanol", "90% Ethanol", "Pure Ethanol"
)

all_results <- bind_rows(
  result_25C %>% mutate(Temperature = "25C"),
  result_40C %>% mutate(Temperature = "40C"),
  result_50C %>% mutate(Temperature = "50C"),
  result_60C %>% mutate(Temperature = "60C")
)


# Now convert the 'Condition' column to a factor with the specified levels
all_results$condition <- factor(all_results$condition, levels = condition_order)

# If previous steps confirm correct naming and values, proceed to plot or further analysis

ggplot(all_results, aes(x = condition, y = mean_cap, fill = Temperature)) +
  geom_bar(stat = "identity", position = position_dodge(width = 0.9) , width=0.9) +
  geom_errorbar(aes(ymin = mean_cap - sd_cap, ymax = mean_cap + sd_cap),
                position = position_dodge(width = 0.9), width = 0.25) +  # Adjust width for clarity
  scale_fill_manual(values =  c("25C" = "navyblue", "40C" = "darkgreen", "50C" = "#B8860B", "60C" = "darkred")) +
  labs(
       x = "Ethanol(%)",
       y = expression(bold( Mean~Delta~Cap)),
       fill = "Temperature") +
  theme(
    plot.title = element_text(size = 12, family = "Times New Roman"),
    axis.text.x = element_text(size = 12 , family = "Times New Roman"),
    axis.text.y= element_text(size = 12, family = "Times New Roman",face="bold"),
    axis.title = element_text(size = 12, family = "Times New Roman",face="bold"),
    legend.text = element_text(size = 12, family = "Times New Roman",face="bold"),
    legend.title = element_text(size = 12, family = "Times New Roman",face="bold"),
    legend.key.height = unit(1.5, "lines"),
    legend.position = "right",
    panel.background = element_blank(),
    plot.background = element_blank(),
    panel.grid.major = element_blank(),
    panel.grid.minor = element_blank(),
    panel.border = element_rect(color = "black", fill = NA)
  )+
  scale_x_discrete(labels = c("0", "10", "20", "30", "40", "50", "60", "70", "80", "90", "100"))
```

# 2. Methanol\_water

## 2.1 Result

```
#__________________________
# cap-time plot
#_________________________
rm(list=ls())

#_______________________
# Libraries
#_______________________
library(nortest)
library(writexl)
library(tidyverse)
library(openxlsx)
library(dplyr)
library(Matrix)
library(lme4)
library(ggplot2)
library(car)
library(extrafont)
library(stringr)
library(gridExtra)
library(combinat)
library(tidyr)
library(cowplot)
library(ggpubr)
library(rlang)
library(broom)
library(purrr)
library(ggpmisc)  # For adding equation and R² to plot
# Import fonts
# font_import(prompt = FALSE)
loadfonts(device = "win")

#_______________________
# Function Definitions
#_______________________
get_sharpness_time <- function(data) {
  # Loop through the data until the cap value increases significantly
  for (i in 2:nrow(data)) {
    # Check if there's a notable increase in the Cap value after a steady range
    if (data$Cap[i] > 160) {
      return(data$Time[i])
    }
  }
  
  # Return NA if no sharpness time is found
  return(NA)
}


# Function to extract timeline points and calculate delta times
get_timeline_points <- function(data, condition, run_name) {
  # Filter data for the specified condition and run
  # Group and calculate cumulative time
  data <- data %>%
    group_by(Condition) %>%
    mutate(Cumulative_Time = cumsum(Time)) %>%
    ungroup() %>%
    mutate(Time = Cumulative_Time) %>%
    select(-Cumulative_Time)
  
  # Replace "-run" with "_run" in the Condition column to ensure uniformity
  data <- data %>%
    mutate(Condition = str_replace(Condition, "-run", "_run"))
  
  filtered_data <- data %>% filter(Condition == paste(condition, run_name, sep = "_"))
  
  # Get the sharpness time (steepest capacitance transition)
  sharpness_time <- get_sharpness_time(filtered_data)
  
  # Filter data based on the sharpness time
  data_before_sharpness <- filtered_data %>%
    filter(Time < sharpness_time)
  
  
  # Filter data based on the sharpness time
  data_after_sharpness <- filtered_data %>%
    filter(Time >= sharpness_time)
  
  # Define timeline points based on the sharpness
  max_cap_value <- max(data_after_sharpness$Cap, na.rm = TRUE)
  lower_bound <- max_cap_value - 5
  upper_bound <- max_cap_value + 5
  
  cap_range_data <- data_after_sharpness %>%
    filter(Cap >= lower_bound & Cap <= upper_bound)
  
  first_flat_time <- cap_range_data$Time[1]
  last_flat_time <- cap_range_data$Time[nrow(cap_range_data)]
  
  data_after_last_time <- data_after_sharpness %>%
    filter(Time > last_flat_time)
  
  delta_t1 <- max(data_before_sharpness$Time) - min(data_before_sharpness$Time)
  delta_t2 <- max(cap_range_data$Time) - min(cap_range_data$Time)
  delta_t3 <- max(data_after_last_time$Time) - min(data_after_last_time$Time)
  
  TOE <- max(filtered_data$Time)-min(filtered_data$Time)
  
  return(list(
    before_flat = data_before_sharpness,
    flat = cap_range_data,
    after_flat = data_after_last_time,
    delta_t1 = delta_t1,
    delta_t2 = delta_t2,
    delta_t3 = delta_t3,
    TOE = TOE
  ))
}

# Function to calculate Mean and SD of delta_t1, delta_t3, and TOE for each combination of three runs
calculate_mean_sd_combinations <- function(data) {
  condition <- unique(data$condition)
  
  # Generate all possible combinations of three runs
  run_combinations <- combn(data$run_name, 3, simplify = FALSE)
  
  # Calculate Mean and SD of delta_t1, delta_t3, and TOE for each combination
  mean_delta_t1 <- sapply(run_combinations, function(runs) {
    selected_delta_t1 <- data %>% filter(run_name %in% runs) %>% pull(delta_t1)
    mean(selected_delta_t1)
  })
  
  sd_delta_t1 <- sapply(run_combinations, function(runs) {
    selected_delta_t1 <- data %>% filter(run_name %in% runs) %>% pull(delta_t1)
    sd(selected_delta_t1)
  })
  
  mean_delta_t2 <- sapply(run_combinations, function(runs) {
    selected_delta_t2 <- data %>% filter(run_name %in% runs) %>% pull(delta_t2)
    mean(selected_delta_t2)
  })
  
  sd_delta_t2 <- sapply(run_combinations, function(runs) {
    selected_delta_t2 <- data %>% filter(run_name %in% runs) %>% pull(delta_t2)
    sd(selected_delta_t2)
  })
  
  mean_delta_t3 <- sapply(run_combinations, function(runs) {
    selected_delta_t3 <- data %>% filter(run_name %in% runs) %>% pull(delta_t3)
    mean(selected_delta_t3)
  })
  
  sd_delta_t3 <- sapply(run_combinations, function(runs) {
    selected_delta_t3 <- data %>% filter(run_name %in% runs) %>% pull(delta_t3)
    sd(selected_delta_t3)
  })
  
  mean_toe <- sapply(run_combinations, function(runs) {
    selected_toes <- data %>% filter(run_name %in% runs) %>% pull(TOE)
    mean(selected_toes)
  })
  
  sd_toe <- sapply(run_combinations, function(runs) {
    selected_toes <- data %>% filter(run_name %in% runs) %>% pull(TOE)
    sd(selected_toes)
  })
  
  # Create a data frame with the combinations, their Mean and SD for delta_t1, delta_t3, and TOE
  mean_sd_table <- data.frame(
    condition = condition,
    combination = sapply(run_combinations, paste, collapse = ", "),
    mean_delta_t1 = mean_delta_t1,
    sd_delta_t1 = sd_delta_t1,
    mean_delta_t2 = mean_delta_t2,
    sd_delta_t2 = sd_delta_t2,
    mean_delta_t3 = mean_delta_t3,
    sd_delta_t3 = sd_delta_t3,
    mean_toe = mean_toe,
    sd_toe = sd_toe
  )
  
  # Return the Mean and SD table
  return(mean_sd_table)
}

# Function to calculate mean Cap for the selected runs
calculate_mean_cap <- function(data, condition, combination) {
  # Extract the runs from the combination column
  selected_runs <- str_split(combination, ", ") %>% unlist()
  
  # Filter the data for the specific condition and selected runs
  filtered_data <- data %>%
    filter(str_detect(Condition, paste0(condition, "_(", paste0(selected_runs, collapse = "|"), ")")))
  
  # Calculate the mean Cap
  mean_cap <- mean(filtered_data$Cap, na.rm = TRUE)
  sd_cap <- sd(filtered_data$Cap, na.rm = TRUE)
  return(list(mean_cap = mean_cap, sd_cap = sd_cap))
}

# Function to Process Data for a Given Temperature
process_data <- function(data, temperature) {
  
  # Replace "-run" with "_run" in the Condition column to ensure uniformity
  data <- data %>%
    mutate(Condition = str_replace(Condition, "-run", "_run"))
  
  unique_conditions <- unique(data$Condition)
  
  split_conditions <- data.frame(
    condition = sapply(strsplit(unique_conditions, "_"), `[`, 1),
    run_name = sapply(strsplit(unique_conditions, "_"), `[`, 2)
  )
  
  
  results <- split_conditions %>%
    mutate(timeline_points = map2(condition, run_name, ~get_timeline_points(data, .x, .y)))
  
  final_results <- results %>%
    rowwise() %>%
    mutate(
      before_flat = list(timeline_points$before_flat),
      flat = list(timeline_points$flat),
      after_flat = list(timeline_points$after_flat),
      delta_t1 = timeline_points$delta_t1,
      delta_t2 = timeline_points$delta_t2,
      delta_t3 = timeline_points$delta_t3,
      TOE = timeline_points$TOE
    ) %>%
    select(-timeline_points) %>%
    ungroup()
  
  final_results <- final_results %>%
    mutate(
      before_flat = sapply(before_flat, function(x) toString(x$Time)),
      flat = sapply(flat, function(x) toString(x$Time)),
      after_flat = sapply(after_flat, function(x) toString(x$Time))
    )
  
  # Save the final results as an Excel file
  write_xlsx(final_results, paste0("timeline_results__Methanol_water", temperature, ".xlsx"))
  mean_sd_combinations_table <- final_results %>%
    group_by(condition) %>%
    do(calculate_mean_sd_combinations(.)) %>%
    ungroup()
  
  # Find the combination with the smallest combined SD for delta_t1, delta_t2, delta_t3, and TOE
  best_combinations_table <- mean_sd_combinations_table %>%
    group_by(condition) %>%
    mutate(total_sd = sd_delta_t1 + sd_delta_t2 + sd_delta_t3 + sd_toe) %>%
    slice_min(total_sd) %>%
    ungroup()
  
  # Apply the function to calculate mean Cap for each row of best_combinations_table
  best_combinations_table <- best_combinations_table %>%
    rowwise() %>%
    mutate(
      mean_cap = calculate_mean_cap(data, as.character(condition), as.character(combination))$mean_cap,
      sd_cap = calculate_mean_cap(data, as.character(condition), as.character(combination))$sd_cap
    ) %>%
    ungroup()
  
  # Define the correct order of conditions
  condition_order <- c(
    "Pure Water", "10% Methanol", "20% Methanol", "30% Methanol",
    "40% Methanol", "50% Methanol", "60% Methanol", "70% Methanol",
    "80% Methanol", "90% Methanol", "Pure Methanol"
  )
  
  # Convert the condition column to a factor with the specified levels
  best_combinations_table$condition <- factor(best_combinations_table$condition, levels = condition_order)
  
  # Arrange the data frame based on the factor levels
  best_combinations_table <- best_combinations_table %>%
    arrange(condition)
  
  # Save the tables to Excel
  write_xlsx(list(
    Mean_SD_Combinations = mean_sd_combinations_table,
    Best_Combinations = best_combinations_table
  ), paste0("mean_sd_combinations_and_selected_runs_Methanol_water", temperature, ".xlsx"))
  
  return(best_combinations_table)
}
# Process for each temperature
data_25C <- read.xlsx("reshape_Water-Methanol.xlsx", sheet = "25C", colNames = TRUE)
result_25C <- process_data(data_25C, "25C")
summary(result_25C)
```

```
##         condition combination        mean_delta_t1    sd_delta_t1    
##  Pure Water  :1   Length:11          Min.   :10.80   Min.   :0.5732  
##  10% Methanol:1   Class :character   1st Qu.:12.79   1st Qu.:1.5013  
##  20% Methanol:1   Mode  :character   Median :13.75   Median :3.1254  
##  30% Methanol:1                      Mean   :14.08   Mean   :3.8095  
##  40% Methanol:1                      3rd Qu.:14.20   3rd Qu.:5.3619  
##  50% Methanol:1                      Max.   :20.16   Max.   :8.7867  
##  (Other)     :5                                                      
##  mean_delta_t2     sd_delta_t2     mean_delta_t3    sd_delta_t3    
##  Min.   : 14.98   Min.   : 1.306   Min.   : 35.8   Min.   : 2.638  
##  1st Qu.:166.90   1st Qu.:11.639   1st Qu.:156.4   1st Qu.:13.345  
##  Median :268.07   Median :22.821   Median :191.2   Median :17.852  
##  Mean   :286.31   Mean   :24.218   Mean   :177.7   Mean   :18.945  
##  3rd Qu.:446.80   3rd Qu.:33.641   3rd Qu.:221.4   3rd Qu.:25.297  
##  Max.   :476.31   Max.   :65.492   Max.   :236.9   Max.   :36.975  
##                                                                    
##     mean_toe          sd_toe          total_sd          mean_cap    
##  Min.   : 73.35   Min.   : 1.924   Min.   :  9.823   Min.   :146.9  
##  1st Qu.:328.66   1st Qu.:15.475   1st Qu.: 46.842   1st Qu.:159.5  
##  Median :479.63   Median :25.616   Median : 69.754   Median :162.8  
##  Mean   :488.68   Mean   :25.429   Mean   : 72.401   Mean   :161.0  
##  3rd Qu.:707.15   3rd Qu.:29.843   3rd Qu.: 77.449   3rd Qu.:164.4  
##  Max.   :737.33   Max.   :72.922   Max.   :151.153   Max.   :168.5  
##                                                                     
##      sd_cap     
##  Min.   :18.36  
##  1st Qu.:20.09  
##  Median :20.52  
##  Mean   :20.73  
##  3rd Qu.:21.71  
##  Max.   :22.33  
##
```

```
data_40C <- read.xlsx("reshape_Water-Methanol.xlsx", sheet = "40C", colNames = TRUE)
result_40C <- process_data(data_40C, "40C")
summary(result_40C)
```

```
##         condition combination        mean_delta_t1    sd_delta_t1    
##  Pure Water  :1   Length:11          Min.   :14.63   Min.   : 4.118  
##  10% Methanol:1   Class :character   1st Qu.:17.87   1st Qu.: 7.491  
##  20% Methanol:1   Mode  :character   Median :22.58   Median :12.248  
##  30% Methanol:1                      Mean   :22.62   Mean   :11.156  
##  40% Methanol:1                      3rd Qu.:25.96   3rd Qu.:14.318  
##  50% Methanol:1                      Max.   :34.26   Max.   :18.006  
##  (Other)     :5                                                      
##  mean_delta_t2     sd_delta_t2     mean_delta_t3    sd_delta_t3    
##  Min.   : 11.64   Min.   : 3.571   Min.   : 32.8   Min.   :  8.84  
##  1st Qu.: 70.93   1st Qu.: 7.000   1st Qu.: 72.9   1st Qu.: 16.86  
##  Median :150.43   Median :11.741   Median :124.3   Median : 59.39  
##  Mean   :128.95   Mean   :13.019   Mean   :118.1   Mean   : 58.49  
##  3rd Qu.:181.06   3rd Qu.:18.035   3rd Qu.:149.3   3rd Qu.: 78.69  
##  Max.   :256.31   Max.   :27.223   Max.   :212.9   Max.   :158.88  
##                                                                    
##     mean_toe          sd_toe          total_sd         mean_cap    
##  Min.   : 90.99   Min.   : 16.36   Min.   : 35.43   Min.   :137.3  
##  1st Qu.:167.43   1st Qu.: 25.22   1st Qu.: 66.11   1st Qu.:150.4  
##  Median :307.29   Median : 41.63   Median :126.03   Median :152.8  
##  Mean   :277.36   Mean   : 64.00   Mean   :146.66   Mean   :151.6  
##  3rd Qu.:376.53   3rd Qu.: 97.58   3rd Qu.:196.08   3rd Qu.:154.3  
##  Max.   :448.16   Max.   :178.99   Max.   :361.52   Max.   :157.4  
##                                                                    
##      sd_cap     
##  Min.   :18.37  
##  1st Qu.:20.60  
##  Median :21.14  
##  Mean   :20.88  
##  3rd Qu.:21.23  
##  Max.   :22.39  
##
```

```
data_50C <- read.xlsx("reshape_Water-Methanol.xlsx", sheet = "50C", colNames = TRUE)
result_50C <- process_data(data_50C, "50C")
summary(result_50C)
```

```
##         condition combination        mean_delta_t1    sd_delta_t1    
##  Pure Water  :1   Length:11          Min.   :14.10   Min.   : 3.537  
##  10% Methanol:1   Class :character   1st Qu.:22.46   1st Qu.: 6.675  
##  20% Methanol:1   Mode  :character   Median :24.88   Median : 8.703  
##  30% Methanol:1                      Mean   :24.71   Mean   :10.783  
##  40% Methanol:1                      3rd Qu.:26.90   3rd Qu.:12.457  
##  50% Methanol:1                      Max.   :34.69   Max.   :31.220  
##  (Other)     :5                                                      
##  mean_delta_t2      sd_delta_t2     mean_delta_t3     sd_delta_t3     
##  Min.   :  6.118   Min.   : 1.630   Min.   : 24.90   Min.   :  1.985  
##  1st Qu.: 30.371   1st Qu.: 2.696   1st Qu.: 35.71   1st Qu.: 10.027  
##  Median : 74.271   Median : 5.972   Median : 43.95   Median : 13.627  
##  Mean   : 69.207   Mean   : 8.285   Mean   : 72.89   Mean   : 56.978  
##  3rd Qu.:104.609   3rd Qu.:11.644   3rd Qu.: 65.24   3rd Qu.: 23.960  
##  Max.   :127.662   Max.   :22.702   Max.   :238.36   Max.   :318.008  
##                                                                       
##     mean_toe          sd_toe           total_sd         mean_cap    
##  Min.   : 58.31   Min.   :  9.692   Min.   : 29.36   Min.   :135.3  
##  1st Qu.: 97.98   1st Qu.: 13.434   1st Qu.: 36.47   1st Qu.:142.4  
##  Median :161.46   Median : 24.994   Median : 59.21   Median :149.1  
##  Mean   :174.12   Mean   : 66.707   Mean   :142.75   Mean   :147.3  
##  3rd Qu.:207.72   3rd Qu.: 40.224   3rd Qu.: 91.32   3rd Qu.:152.0  
##  Max.   :358.23   Max.   :322.572   Max.   :650.71   Max.   :159.2  
##                                                                     
##      sd_cap     
##  Min.   :16.57  
##  1st Qu.:20.15  
##  Median :20.57  
##  Mean   :20.27  
##  3rd Qu.:20.94  
##  Max.   :22.42  
##
```

```
data_60C <- read.xlsx("reshape_Water-Methanol.xlsx", sheet = "60C", colNames = TRUE)
result_60C <- process_data(data_60C, "60C")
summary(result_60C)
```

```
##         condition combination        mean_delta_t1    sd_delta_t1    
##  Pure Water  :1   Length:11          Min.   :10.41   Min.   : 1.476  
##  10% Methanol:1   Class :character   1st Qu.:13.95   1st Qu.: 3.126  
##  20% Methanol:1   Mode  :character   Median :18.65   Median : 9.552  
##  30% Methanol:1                      Mean   :17.84   Mean   : 9.582  
##  40% Methanol:1                      3rd Qu.:20.69   3rd Qu.:12.091  
##  50% Methanol:1                      Max.   :29.84   Max.   :21.933  
##  (Other)     :5                                                      
##  mean_delta_t2     sd_delta_t2      mean_delta_t3      sd_delta_t3      
##  Min.   : 5.171   Min.   : 0.3655   Min.   :  9.374   Min.   :  0.7324  
##  1st Qu.:28.390   1st Qu.: 2.9928   1st Qu.: 22.003   1st Qu.:  3.0563  
##  Median :53.392   Median : 4.9391   Median : 29.484   Median :  5.9628  
##  Mean   :46.131   Mean   : 6.0695   Mean   : 48.503   Mean   : 39.0324  
##  3rd Qu.:67.025   3rd Qu.: 7.5679   3rd Qu.: 38.251   3rd Qu.:  8.4860  
##  Max.   :80.895   Max.   :16.2595   Max.   :256.387   Max.   :366.2017  
##                                                                         
##     mean_toe          sd_toe           total_sd         mean_cap    
##  Min.   : 31.47   Min.   :  2.707   Min.   : 11.68   Min.   :140.5  
##  1st Qu.: 74.21   1st Qu.: 12.512   1st Qu.: 27.20   1st Qu.:146.1  
##  Median :116.17   Median : 15.559   Median : 33.38   Median :151.5  
##  Mean   :119.19   Mean   : 49.559   Mean   :104.24   Mean   :149.8  
##  3rd Qu.:131.51   3rd Qu.: 25.340   3rd Qu.: 59.42   3rd Qu.:153.7  
##  Max.   :354.82   Max.   :368.068   Max.   :749.28   Max.   :156.4  
##                                                                     
##      sd_cap     
##  Min.   :19.36  
##  1st Qu.:20.65  
##  Median :20.98  
##  Mean   :21.12  
##  3rd Qu.:21.61  
##  Max.   :23.46  
##
```

## 2.2 FigureS5,S6,S7,S8,S9

```
#________________________________
#line plot for for TOE, delta_t1, delta_t2, and delta_t3
#__________________________________
plot_results <- function(data, y_var, sd_var, y_label, output_filename) {
  p <- ggplot(data, aes(x = condition, y = !!sym(y_var), group = temperature, color = temperature)) +
    geom_line(size = 1.2) +
    geom_point(size = 3) +
    geom_errorbar(aes(ymin = !!sym(y_var) - !!sym(sd_var),
                      ymax = !!sym(y_var) + !!sym(sd_var)),
                  width = 0.2, size = 1) +
    labs(x = "Methanol (%)", y = y_label, color = "Temperature") +
    theme_minimal() +
    theme(
      axis.text.x = element_text(size = 12, family = "Times New Roman",face="bold"),
      axis.text.y= element_text(size = 12, family = "Times New Roman",face="bold"),
      axis.title = element_text(size = 12, family = "Times New Roman",face="bold"),
      legend.text = element_text(size = 12, family = "Times New Roman",face="bold"),
      legend.title = element_text(size = 12, family = "Times New Roman",face="bold"),
      legend.position = c(0.8, 0.8), 
      legend.direction = "vertical",
      legend.spacing.y = unit(0.002, 'cm'),
      legend.key.size = unit(0.8, 'lines'),
      panel.background = element_blank(),
      plot.background = element_blank(),
      panel.grid.major = element_blank(),
      panel.grid.minor = element_blank(),
      panel.border = element_rect(color = "black", fill = NA)
    ) +
    scale_x_discrete(labels = c("0", "10", "20", "30", "40", "50", "60", "70", "80", "90", "100")) +
    scale_color_manual(values = c("25C" = "navyblue", "40C" = "darkgreen", "50C" = "#B8860B", "60C" = "darkred"))
  print(p)
  # Save the plot
  ggsave(output_filename, plot = p, width = 10, height = 6)
}

# Combine all results into one data frame
combined_results <- bind_rows(
  result_25C %>% mutate(temperature = "25C"),
  result_40C %>% mutate(temperature = "40C"),
  result_50C %>% mutate(temperature = "50C"),
  result_60C %>% mutate(temperature = "60C")
)

# Convert condition to a factor with the correct order
condition_order <- c(
  "Pure Water", "10% Methanol", "20% Methanol", "30% Methanol",
  "40% Methanol", "50% Methanol", "60% Methanol", "70% Methanol",
  "80% Methanol", "90% Methanol", "Pure Methanol"
)

combined_results$condition <- factor(combined_results$condition, levels = condition_order)

# Plot TOE
plot_results(combined_results, "mean_toe", "sd_toe", "TOE (s)", "TOE_conditions_temperatures.png")
```

```
# Plot delta_t1
plot_results(combined_results, "mean_delta_t1", "sd_delta_t1", expression(bold(Delta~t[1]~"(s)")), "delta_t1_conditions_temperatures.png")
```

```
# Plot delta_t2
plot_results(combined_results, "mean_delta_t2", "sd_delta_t2", expression(bold(Delta~t[2]~"(s)")), "delta_t2_conditions_temperatures.png")
```

```
# Plot delta_t3
plot_results(combined_results, "mean_delta_t3", "sd_delta_t3", expression(bold(Delta~t[3]~"(s)")), "delta_t3_conditions_temperatures.png")
```

## 2.3 Table2.1

```
data_25C <- read.xlsx("reshape_Water-Methanol.xlsx", sheet = "25C", colNames = TRUE)
data_40C <- read.xlsx("reshape_Water-Methanol.xlsx", sheet = "40C", colNames = TRUE)
data_50C <- read.xlsx("reshape_Water-Methanol.xlsx", sheet = "50C", colNames = TRUE)
data_60C <- read.xlsx("reshape_Water-Methanol.xlsx", sheet = "60C", colNames = TRUE)


df_25C <- read.xlsx("timeline_results__Methanol_water25C.xlsx", colNames = TRUE)
df_40C <- read.xlsx("timeline_results__Methanol_water40C.xlsx", colNames = TRUE)
df2_40C <- read.xlsx("mean_sd_combinations_and_selected_runs_Methanol_water40C.xlsx", sheet = "Best_Combinations", colNames = TRUE)
df2_25C <- read.xlsx("mean_sd_combinations_and_selected_runs_Methanol_water25C.xlsx", sheet = "Best_Combinations", colNames = TRUE)
df_50C <- read.xlsx("timeline_results__Methanol_water50C.xlsx", colNames = TRUE)
df2_50C <- read.xlsx("mean_sd_combinations_and_selected_runs_Methanol_water50C.xlsx", sheet = "Best_Combinations", colNames = TRUE)

df_60C <- read.xlsx("timeline_results__Methanol_water60C.xlsx", colNames = TRUE)
df2_60C <- read.xlsx("mean_sd_combinations_and_selected_runs_Methanol_water60C.xlsx", sheet = "Best_Combinations", colNames = TRUE)

#________________________
#Dynamic range
#_______________________
process_dynemic <- function(data, temp_label, df, df2) {
  
  data <- data %>%
    group_by(Condition) %>%
    mutate(Cumulative_Time = cumsum(Time)) %>%
    ungroup() %>%
    mutate(Time = Cumulative_Time) %>%
    select(-Cumulative_Time) %>%
    mutate(Condition = str_replace(Condition, "-run", "_run"))
  
  # Assuming 'df' is already loaded and structured properly
  df$condition <- factor(df$condition)
  
  # Preparing to selected data
  selected_data <- data.frame()
  
  # Process each condition from df2
  unique_conditions <- unique(df2$condition)
  
  # Iterate over each unique condition from df2 which matches conditions specified in df
  for (condition_to_match in unique(df2$condition)) {
    # Subset df2 for specific condition
    df2_subset <- df2 %>% filter(str_detect(condition, condition_to_match))
    selected_runs <- unlist(strsplit(as.character(df2_subset$combination[1]), ", "))
    run = selected_runs[1]  # Ensuring the run exists in our data columns
    
    # Filter the main data for the selected run and condition
    filtered_data <- data %>%
      filter(Condition == paste(condition_to_match, run, sep = "_"))
    
    # Get the flat time range from df which matches the current condition and run
    flat_times <- df %>% 
      filter(condition == condition_to_match & run_name == run) %>% 
      pull(flat) %>% 
      strsplit(split = ",") %>%
      unlist() %>%
      as.numeric()
    
    # Assuming the existence of a function to get the appropriate sharpness time
    sharpness_time <- get_sharpness_time(filtered_data) # Define or adjust this function based on our dataset
    
    # Filtering to include only times within the flat time range
    if (length(flat_times) > 0) {
      data_run <- filtered_data %>%
        filter(Time >= min(flat_times) & Time <= max(flat_times)) %>%
        mutate(Time_aligned = pmax(Time - sharpness_time, 0),
               Cap_aligned = Cap)
      
      selected_data <- bind_rows(selected_data, data_run %>% select(Time_aligned, Cap_aligned, Condition))
    }
  }
  
  # Compute dynamic range for Capacitance (Cap)
  cap_dynamic <- ((max(selected_data$Cap_aligned) - min(selected_data$Cap_aligned))/mean(selected_data$Cap_aligned))*100
  print(paste("Dynamic range for Capacitance in temp (%)",temp_label, ":", cap_dynamic))
  
  # Compute dynamic range for Time
  time_dynamic <- ((max(selected_data$Time_aligned) - min(selected_data$Time_aligned))/mean(selected_data$Time_aligned))*100
  print(paste("Dynamic range for Time in temp",temp_label, ":",time_dynamic))
}

process_dynemic(data_25C, "25C", df_25C, df2_25C)
```

```
## [1] "Dynamic range for Capacitance in temp (%) 25C : 4.64330641435545"
## [1] "Dynamic range for Time in temp 25C : 306.013826737946"
```

```
process_dynemic(data_40C, "40C", df_40C, df2_40C)
```

```
## [1] "Dynamic range for Capacitance in temp (%) 40C : 5.35819266849556"
## [1] "Dynamic range for Time in temp 40C : 335.206750382034"
```

```
process_dynemic(data_50C, "50C", df_50C, df2_50C)
```

```
## [1] "Dynamic range for Capacitance in temp (%) 50C : 4.82747729321347"
## [1] "Dynamic range for Time in temp 50C : 309.763407263064"
```

```
process_dynemic(data_60C, "60C", df_60C, df2_60C)
```

```
## [1] "Dynamic range for Capacitance in temp (%) 60C : 4.42999186235866"
## [1] "Dynamic range for Time in temp 60C : 294.717625600186"
```

## 2.4 Table2.2

```
# Load necessary libraries
# Load the data
df2_40C <- read.xlsx("mean_sd_combinations_and_selected_runs_Methanol_water40C.xlsx", sheet = "Best_Combinations")
df2_25C <- read.xlsx("mean_sd_combinations_and_selected_runs_Methanol_water25C.xlsx", sheet = "Best_Combinations")
df2_50C <- read.xlsx("mean_sd_combinations_and_selected_runs_Methanol_water50C.xlsx", sheet = "Best_Combinations")
df2_60C <- read.xlsx("mean_sd_combinations_and_selected_runs_Methanol_water60C.xlsx", sheet = "Best_Combinations")

# Extract the 'mean_delta_t2' column
delta_t2_25C <- df2_25C$mean_delta_t2
delta_t2_40C <- df2_40C$mean_delta_t2
delta_t2_50C <- df2_50C$mean_delta_t2
delta_t2_60C <- df2_60C$mean_delta_t2

# Normality test using Shapiro-Wilk test
shapiro_test_25C <- shapiro.test(delta_t2_25C)
shapiro_test_40C <- shapiro.test(delta_t2_40C)
shapiro_test_50C <- shapiro.test(delta_t2_50C)
shapiro_test_60C <- shapiro.test(delta_t2_60C)

# Display the results of the normality tests
print(shapiro_test_25C)
```

```
## 
##  Shapiro-Wilk normality test
## 
## data:  delta_t2_25C
## W = 0.91985, p-value = 0.3174
```

```
print(shapiro_test_40C)
```

```
## 
##  Shapiro-Wilk normality test
## 
## data:  delta_t2_40C
## W = 0.97058, p-value = 0.8923
```

```
print(shapiro_test_50C)
```

```
## 
##  Shapiro-Wilk normality test
## 
## data:  delta_t2_50C
## W = 0.92321, p-value = 0.3463
```

```
print(shapiro_test_60C)
```

```
## 
##  Shapiro-Wilk normality test
## 
## data:  delta_t2_60C
## W = 0.94217, p-value = 0.5463
```

```
# Equality of variances test using Levene's test
levene_test <- leveneTest(c(delta_t2_25C, delta_t2_40C, delta_t2_50C, delta_t2_60C), 
                          group = rep(c("25C", "40C", "50C", "60C"), times = c(length(delta_t2_25C), length(delta_t2_40C), length(delta_t2_50C), length(delta_t2_60C))))

# Display the result of the Levene's test
print(levene_test)
```

```
## Levene's Test for Homogeneity of Variance (center = median)
##       Df F value   Pr(>F)    
## group  3  11.998 9.67e-06 ***
##       40                     
## ---
## Signif. codes:  0 '***' 0.001 '**' 0.01 '*' 0.05 '.' 0.1 ' ' 1
```

## 2.5 FigureS10

```
#_________________________________
#barplot for cap
#_________________________
condition_order <- c(
  "Pure Water", "10% Methanol", "20% Methanol", "30% Methanol",
  "40% Methanol", "50% Methanol", "60% Methanol", "70% Methanol",
  "80% Methanol", "90% Methanol", "Pure Methanol"
)

all_results <- bind_rows(
  result_25C %>% mutate(Temperature = "25C"),
  result_40C %>% mutate(Temperature = "40C"),
  result_50C %>% mutate(Temperature = "50C"),
  result_60C %>% mutate(Temperature = "60C")
)


# Now convert the 'Condition' column to a factor with the specified levels
all_results$condition <- factor(all_results$condition, levels = condition_order)

# If previous steps confirm correct naming and values, proceed to plot or further analysis

ggplot(all_results, aes(x = condition, y = mean_cap, fill = Temperature)) +
  geom_bar(stat = "identity", position = position_dodge(width = 0.9) , width=0.9) +
  geom_errorbar(aes(ymin = mean_cap - sd_cap, ymax = mean_cap + sd_cap),
                position = position_dodge(width = 0.9), width = 0.25) +  # Adjust width for clarity
  scale_fill_manual(values =  c("25C" = "navyblue", "40C" = "darkgreen", "50C" = "#B8860B", "60C" = "darkred")) +
  labs(
       x = "Methanol(%)",
       y = expression(bold( Mean~Delta~Cap)),
       fill = "Temperature") +
  theme(
    plot.title = element_text(size = 12, family = "Times New Roman"),
    axis.text.x = element_text(size = 12 , family = "Times New Roman"),
    axis.text.y= element_text(size = 12, family = "Times New Roman",face="bold"),
    axis.title = element_text(size = 12, family = "Times New Roman",face="bold"),
    legend.text = element_text(size = 12, family = "Times New Roman",face="bold"),
    legend.title = element_text(size = 12, family = "Times New Roman",face="bold"),
    legend.key.height = unit(1.5, "lines"),
    legend.position = "right",
    panel.background = element_blank(),
    plot.background = element_blank(),
    panel.grid.major = element_blank(),
    panel.grid.minor = element_blank(),
    panel.border = element_rect(color = "black", fill = NA)
  )+
  scale_x_discrete(labels = c("0", "10", "20", "30", "40", "50", "60", "70", "80", "90", "100"))
```

# 3. Methanol\_Ethanol

## 3.1 Table3.1

```
# Load necessary libraries
# Load the data
df2_25C <- read.xlsx("mean_sd_combinations_and_selected_runs_methanol_ethanol25C.xlsx", sheet = "Best_Combinations", colNames = TRUE)


# Extract the 'mean_delta_t2' column
delta_t2_25C <- df2_25C$mean_delta_t2


# Normality test using Shapiro-Wilk test
shapiro_test_25C <- shapiro.test(delta_t2_25C)


# Display the results of the normality tests
print(shapiro_test_25C)
```

```
## 
##  Shapiro-Wilk normality test
## 
## data:  delta_t2_25C
## W = 0.89797, p-value = 0.1746
```

## 3.5 FigureS11

```
combined_results <- bind_rows(
  result_25C %>% mutate(temperature = "25C"),
)

condition_order <- c(
  "0% Methanol", "10% Methanol", "20% Methanol", "30% Methanol",
  "40% Methanol", "50% Methanol", "60% Methanol", "70% Methanol",
  "80% Methanol", "90% Methanol", "Pure Methanol"
)

# Replace 'Pure Water' with '0' and remove 'Methanol' and '%' from other labels
clean_condition_labels <-  gsub(" Methanol|%", "", condition_order)

# Create the plot with summarized data and customizations
combined_results <- combined_results %>%
  mutate(Condition_Num = as.numeric(factor(condition, levels = condition_order)))

# Step 1: Summarize the data to calculate the overall mean and SD for each temperature
temperature_summary <- combined_results %>%
  group_by(temperature) %>%
  summarize(
    overall_mean_toe = mean(mean_delta_t2),
    overall_sd_toe = sd(mean_delta_t2),
    .groups = 'drop'
  )

# Step 2: Create the plot with the summarized data
p <- ggplot(combined_results, aes(x = temperature, y = mean_delta_t2)) +
  geom_boxplot(outlier.shape = NA) +
  geom_errorbar(data = temperature_summary, 
                aes(x = temperature, ymin = overall_mean_toe - overall_sd_toe, ymax = overall_mean_toe + overall_sd_toe), 
                width = 0.2, linewidth = 1, color = "black", inherit.aes = FALSE) +  # Single error bar for each temperature
  geom_jitter(aes(color = factor(Condition_Num)), size = 4, width = 0.1) +
  
  # Use dark colors for each condition
  scale_color_manual(
    values = c("navyblue", "brown", "#B8860B", "darkgreen", "darkred", "#8B4513", "#4682B4", "#556B2F", "#8B008B", "#2E8B57", "#483D8B"),
    breaks = seq_along(condition_order),
    labels = clean_condition_labels  # Updated labels with '0' for 'Pure Water' and cleaned others
  ) +
  
  # Update axis labels and legend title
  labs(
    x = "Temperature",
    y = expression(bold(Delta~t[2]~"(s)")),
    color = "Methanol (%)"
  ) +
  
  # Customize the theme
  theme_minimal() +
  theme(
    axis.text= element_text(size = 12, family = "Times New Roman",face="bold"),
    axis.title = element_text(size = 12, family = "Times New Roman",face="bold"),
    legend.text = element_text(size = 12, family = "Times New Roman",face="bold"),
    legend.title = element_text(size = 12, family = "Times New Roman",face="bold"),
    legend.position = c(0.9, 0.5), 
    legend.direction = "vertical",
    panel.background = element_blank(),
    plot.background = element_blank(),
    panel.grid.major = element_blank(),
    panel.grid.minor = element_blank(),
    panel.border = element_rect(color = "black", fill = NA)
  )

p
```

## 3.6 FigureS12

```
#_________________________________
#barplot for cap
#_________________________
condition_order <- c(
  "0% Methanol", "10% Methanol", "20% Methanol", "30% Methanol",
  "40% Methanol", "50% Methanol", "60% Methanol", "70% Methanol",
  "80% Methanol", "90% Methanol", "Pure Methanol"
)

all_results <- bind_rows(
  result_25C %>% mutate(Temperature = "25C")
)


# Now convert the 'Condition' column to a factor with the specified levels
all_results$condition <- factor(all_results$condition, levels = condition_order)

# If previous steps confirm correct naming and values, proceed to plot or further analysis

ggplot(all_results, aes(x = condition, y = mean_cap, fill = Temperature)) +
  geom_bar(stat = "identity", position = position_dodge(width = 0.9) , width=0.9) +
  geom_errorbar(aes(ymin = mean_cap - sd_cap, ymax = mean_cap + sd_cap),
                position = position_dodge(width = 0.9), width = 0.25) +  # Adjust width for clarity
  scale_fill_manual(values =  c("25C" = "navyblue")) +
  labs(
       x = "Methanol(%)",
       y = expression(bold( Mean~Delta~Cap)),
       fill = "Temperature") +
  theme(
    plot.title = element_text(size = 12, family = "Times New Roman"),
    axis.text.x = element_text(size = 12 , family = "Times New Roman"),
    axis.text.y= element_text(size = 12, family = "Times New Roman",face="bold"),
    axis.title = element_text(size = 12, family = "Times New Roman",face="bold"),
    legend.text = element_text(size = 12, family = "Times New Roman",face="bold"),
    legend.title = element_text(size = 12, family = "Times New Roman",face="bold"),
    legend.key.height = unit(1.5, "lines"),
    legend.position = "right",
    panel.background = element_blank(),
    plot.background = element_blank(),
    panel.grid.major = element_blank(),
    panel.grid.minor = element_blank(),
    panel.border = element_rect(color = "black", fill = NA)
  )+
  scale_x_discrete(labels = c("0", "10", "20", "30", "40", "50", "60", "70", "80", "90", "100"))
```

# 4. All combinations

## 4.1 FigureS13

```
rm(list=ls())
#_______________________
library(nortest)
library(writexl)
library(tidyverse)
library(openxlsx)
library(dplyr)
library(Matrix)
library(lme4)
library(ggplot2)
library(car)
library(extrafont)
library(stringr)
library(gridExtra)
library(combinat)
library(tidyr)
library(cowplot)
library(ggpubr)
library(rlang)
library(broom)
library(purrr)

# Load data
ethanol_water <- read.xlsx("mean_sd_combinations_and_selected_runs_ethanol_water25C.xlsx", sheet = "Best_Combinations", colNames = TRUE)
methanol_water <- read.xlsx("mean_sd_combinations_and_selected_runs_methanol_water25C.xlsx", sheet = "Best_Combinations", colNames = TRUE)
methanol_ethanol <- read.xlsx("mean_sd_combinations_and_selected_runs_methanol_ethanol25C.xlsx", sheet = "Best_Combinations", colNames = TRUE)

ethanol_water_points_25 <- read.xlsx("timeline_results__ethanol_water25C.xlsx", colNames = TRUE)
ethanol_water_points_25$mixture_type <- "Ethanol-Water"


methanol_water_points_25 <- read.xlsx("timeline_results__Methanol_water25C.xlsx", colNames = TRUE)
methanol_water_points_25$mixture_type <- "Methanol-Water"


methanol_ethanol_points_25 <- read.xlsx("timeline_results__Methanol_Ethanol25C.xlsx", colNames = TRUE)
methanol_ethanol_points_25$mixture_type <- "Methanol-Ethanol"


# Create a new column to distinguish datasets and unify into one dataset
ethanol_water$mixture_type <- "Ethanol-Water"
methanol_water$mixture_type <- "Methanol-Water"
methanol_ethanol$mixture_type <- "Methanol-Ethanol"

# Combine the three datasets for Delta t2
combined_data_delta_t2 <- bind_rows(
  ethanol_water %>% select(condition,combination, mean_delta_t2, sd_delta_t2, mixture_type),
  methanol_water %>% select(condition,combination, mean_delta_t2, sd_delta_t2, mixture_type),
  methanol_ethanol %>% select(condition,combination, mean_delta_t2, sd_delta_t2, mixture_type)
)

# Reorder mixture_type levels
combined_data_delta_t2$mixture_type <- factor(combined_data_delta_t2$mixture_type, levels = c("Methanol-Water", "Ethanol-Water", "Methanol-Ethanol"))

# Define a function to map condition names to numeric alcohol percentages
map_condition_to_percentage <- function(condition) {
  case_when(
    condition %in% c("Pure Water", "0% Methanol") ~ 0,
    grepl("10%", condition) ~ 10,
    grepl("20%", condition) ~ 20,
    grepl("30%", condition) ~ 30,
    grepl("40%", condition) ~ 40,
    grepl("50%", condition) ~ 50,
    grepl("60%", condition) ~ 60,
    grepl("70%", condition) ~ 70,
    grepl("80%", condition) ~ 80,
    grepl("90%", condition) ~ 90,
    condition %in% c("Pure Ethanol", "Pure Methanol") ~ 100,
    TRUE ~ NA_real_  # Handle unexpected cases
  )
}

# Apply the function to the condition column
combined_data_delta_t2$condition <- map_condition_to_percentage(combined_data_delta_t2$condition)

# Combine all points data into one dataframe for selected runs
selected_runs_points <- bind_rows(
  ethanol_water_points_25,
  methanol_water_points_25,
  methanol_ethanol_points_25
)

selected_runs_points$condition <- as.numeric(map_condition_to_percentage(selected_runs_points$condition))

expanded <- combined_data_delta_t2 %>%
  separate_rows(combination, sep = ",\\s*") %>%
  rename(run_name = combination) %>%
  mutate(run_name = str_trim(run_name)) %>%
  select(condition, run_name, mixture_type)


selected_combination_runs <- selected_runs_points %>%
  inner_join(expanded, by = c("condition", "run_name", "mixture_type"))


# Function to calculate metrics
calculate_metrics <- function(model, data) {
  predictions <- augment(model)
  r_squared <- glance(model)$r.squared
  rmse <- sqrt(mean(residuals(model)^2))
  mae <- mean(abs(residuals(model)))
  range_y <- max(data) - min(data)
  rmse_percentage <- (rmse / range_y) * 100
  mae_percentage <- (mae / range_y) * 100
  
  return(list(r_squared = r_squared, rmse = rmse, mae = mae, rmse_percentage = rmse_percentage))
}

# Fit linear models for each `mixture_type`
models <- combined_data_delta_t2 %>%
  group_by(mixture_type) %>%
  do(model = lm(mean_delta_t2 ~ condition, data = .))

# Calculate metrics for each model
metrics <- models %>%
  rowwise() %>%
  mutate(metrics = list(calculate_metrics(model, combined_data_delta_t2$mean_delta_t2))) %>%
  unnest_wider(metrics)

generate_combined_boxplot <- function(data_combined, metrics) {
  
  # Ensure the order of `mixture_type` is correct (Ethanol-Water, Methanol-Water, Methanol-Ethanol)
  data_combined <- data_combined %>%
    mutate(mixture_type = factor(mixture_type, levels = c("Ethanol-Water", "Methanol-Water", "Methanol-Ethanol")))
  
  # Create the table with Type Mixtures, R², and RMSE
  table_data <- data.frame(
    `Type Mixtures` = c("Ethanol-Water", "Methanol-Water", "Methanol-Ethanol"),
    `R2` = round(metrics$r_squared, 3),
    `RMSE` = round(metrics$rmse, 2)
  )
  
  # Generate the table grob
  table_grob <- tableGrob(table_data, rows = NULL, theme = ttheme_minimal(
    core = list(
      fg_params = list(fontface = "bold", fontsize = 12, col = "black"),
      bg_params = list(fill = NA, col = "black", lwd = 0.5)  # Transparent background and black borders
    ),
    colhead = list(
      fg_params = list(fontface = "bold", fontsize = 12, col = "black"),
      bg_params = list(fill = NA, col = "black", lwd = 0.5)  # Transparent background and black borders for header
    ),
    padding = unit(c(5, 5), "mm")
  ))
  
  # Add custom font family to table
  table_grob$grobs <- lapply(table_grob$grobs, function(grob) {
    if ("grid.text" %in% class(grob)) {
      grob$gp <- gpar(fontfamily = "Times New Roman")  # Set Times New Roman font
    }
    return(grob)
  })
  
  # Create the plot with corrected aesthetics
  p <- ggplot(data_combined, aes(x = condition, y = mean_delta_t2, color = mixture_type)) +
    geom_point(size = 5) +  # Main points to represent data
    geom_smooth(aes(color = mixture_type), method = "lm", se = FALSE, size = 1, linetype = "solid") +  # Solid lines for trend
    geom_point(data = selected_combination_runs, aes(x = condition, y = delta_t2, color = mixture_type), size = 4, alpha = 0.6) +  # Smaller points from selected runs
    labs(
      x = "(%)",
      y = expression(bold(Delta~t[2]~"(s)")),
      color = "Mixture Type"
    ) +
    scale_color_manual(
      values = c("Ethanol-Water" = "navyblue", 
                 "Methanol-Water" = "#B8860B", 
                 "Methanol-Ethanol" = "#A52A2A"),
      labels = c("E-W(%E)", "M-W(%M)", "M-E(%M)")
    ) +
    theme_minimal() +
    theme(
      axis.text.x = element_text(size = 12, family = "Times New Roman", face = "bold"),
      axis.text.y = element_text(size = 12, family = "Times New Roman", face = "bold"),
      axis.title.x = element_text(size = 12, family = "Times New Roman"),
      axis.title.y = element_text(size = 12, family = "Times New Roman"),
      legend.text = element_text(size = 12, family = "Times New Roman", face = "bold"),
      legend.title = element_text(size = 12, family = "Times New Roman", face = "bold"),
      legend.position = c(0.85, 0.55), 
      legend.direction = "vertical",
      panel.background = element_blank(),
      plot.background = element_blank(),
      panel.grid.major = element_blank(),
      panel.grid.minor = element_blank(),
      panel.border = element_rect(color = "black", fill = NA)
    ) +
    scale_x_continuous(breaks = seq(0, 100, by = 10))  # Numeric x-axis with breaks every 10%
  
  # Add the table inside the plot using annotation_custom
  p <- p + annotation_custom(
    grob = table_grob,
    xmin = 60,  # Adjust these values based on where we want the table inside the plot
    xmax = 100,
    ymin = 400,
    ymax = 500
  )
  
  return(p)
}

# Generate the boxplot with the table inside the plot
combined_plot <- generate_combined_boxplot(combined_data_delta_t2, metrics)
ggsave("boxplot_with_table_inside.png", plot = combined_plot, width = 9  , height = 6)

#####################left-side##################
#__________________________
# cap-time plot
#_________________________
rm(list=ls())

#_______________________
# Libraries
#_______________________
library(nortest)
library(writexl)
library(tidyverse)
library(openxlsx)
library(dplyr)
library(Matrix)
library(lme4)
library(ggplot2)
library(car)
library(extrafont)
library(stringr)
library(gridExtra)
library(combinat)
library(tidyr)
library(cowplot)
library(ggpubr)
library(rlang)
library(broom)
library(purrr)
library(ggpmisc)  # For adding equation and R² to plot
# Import fonts
# font_import(prompt = FALSE)
loadfonts(device = "win")

#_______________________
# Function Definitions
#_______________________
get_sharpness_time <- function(data) {
  # Loop through the data until the cap value increases significantly
  for (i in 2:nrow(data)) {
    # Check if there's a notable increase in the Cap value after a steady range
    if (data$Cap[i] > 160) {
      return(data$Time[i])
    }
  }
  
  # Return NA if no sharpness time is found
  return(NA)
}


# Function to extract timeline points and calculate delta times
get_timeline_points <- function(data, condition, run_name) {
  # Filter data for the specified condition and run
  # Group and calculate cumulative time
  data <- data %>%
    group_by(Condition) %>%
    mutate(Cumulative_Time = cumsum(Time)) %>%
    ungroup() %>%
    mutate(Time = Cumulative_Time) %>%
    select(-Cumulative_Time)
  
  # Replace "-run" with "_run" in the Condition column to ensure uniformity
  data <- data %>%
    mutate(Condition = str_replace(Condition, "-run", "_run"))
  
  filtered_data <- data %>% filter(Condition == paste(condition, run_name, sep = "_"))
  
  # Get the sharpness time (steepest capacitance transition)
  sharpness_time <- get_sharpness_time(filtered_data)
  
  # Filter data based on the sharpness time
  data_before_sharpness <- filtered_data %>%
    filter(Time < sharpness_time)
  
  
  # Filter data based on the sharpness time
  data_after_sharpness <- filtered_data %>%
    filter(Time >= sharpness_time)
  
  # Define timeline points based on the sharpness
  max_cap_value <- max(data_after_sharpness$Cap, na.rm = TRUE)
  lower_bound <- max_cap_value - 5
  upper_bound <- max_cap_value + 5
  
  cap_range_data <- data_after_sharpness %>%
    filter(Cap >= lower_bound & Cap <= upper_bound)
  
  first_flat_time <- cap_range_data$Time[1]
  last_flat_time <- cap_range_data$Time[nrow(cap_range_data)]
  
  data_after_last_time <- data_after_sharpness %>%
    filter(Time > last_flat_time)
  
  delta_t1 <- max(data_before_sharpness$Time) - min(data_before_sharpness$Time)
  delta_t2 <- max(cap_range_data$Time) - min(cap_range_data$Time)
  delta_t3 <- max(data_after_last_time$Time) - min(data_after_last_time$Time)
  
  TOE <- max(filtered_data$Time)-min(filtered_data$Time)
  
  return(list(
    before_flat = data_before_sharpness,
    flat = cap_range_data,
    after_flat = data_after_last_time,
    delta_t1 = delta_t1,
    delta_t2 = delta_t2,
    delta_t3 = delta_t3,
    TOE = TOE
  ))
}

# Function to calculate Mean and SD of delta_t1, delta_t3, and TOE for each combination of three runs
calculate_mean_sd_combinations <- function(data) {
  condition <- unique(data$condition)
  
  # Generate all possible combinations of three runs
  run_combinations <- combn(data$run_name, 3, simplify = FALSE)
  
  # Calculate Mean and SD of delta_t1, delta_t3, and TOE for each combination
  mean_delta_t1 <- sapply(run_combinations, function(runs) {
    selected_delta_t1 <- data %>% filter(run_name %in% runs) %>% pull(delta_t1)
    mean(selected_delta_t1)
  })
  
  sd_delta_t1 <- sapply(run_combinations, function(runs) {
    selected_delta_t1 <- data %>% filter(run_name %in% runs) %>% pull(delta_t1)
    sd(selected_delta_t1)
  })
  
  mean_delta_t2 <- sapply(run_combinations, function(runs) {
    selected_delta_t2 <- data %>% filter(run_name %in% runs) %>% pull(delta_t2)
    mean(selected_delta_t2)
  })
  
  sd_delta_t2 <- sapply(run_combinations, function(runs) {
    selected_delta_t2 <- data %>% filter(run_name %in% runs) %>% pull(delta_t2)
    sd(selected_delta_t2)
  })
  
  mean_delta_t3 <- sapply(run_combinations, function(runs) {
    selected_delta_t3 <- data %>% filter(run_name %in% runs) %>% pull(delta_t3)
    mean(selected_delta_t3)
  })
  
  sd_delta_t3 <- sapply(run_combinations, function(runs) {
    selected_delta_t3 <- data %>% filter(run_name %in% runs) %>% pull(delta_t3)
    sd(selected_delta_t3)
  })
  
  mean_toe <- sapply(run_combinations, function(runs) {
    selected_toes <- data %>% filter(run_name %in% runs) %>% pull(TOE)
    mean(selected_toes)
  })
  
  sd_toe <- sapply(run_combinations, function(runs) {
    selected_toes <- data %>% filter(run_name %in% runs) %>% pull(TOE)
    sd(selected_toes)
  })
  
  # Create a data frame with the combinations, their Mean and SD for delta_t1, delta_t3, and TOE
  mean_sd_table <- data.frame(
    condition = condition,
    combination = sapply(run_combinations, paste, collapse = ", "),
    mean_delta_t1 = mean_delta_t1,
    sd_delta_t1 = sd_delta_t1,
    mean_delta_t2 = mean_delta_t2,
    sd_delta_t2 = sd_delta_t2,
    mean_delta_t3 = mean_delta_t3,
    sd_delta_t3 = sd_delta_t3,
    mean_toe = mean_toe,
    sd_toe = sd_toe
  )
  
  # Return the Mean and SD table
  return(mean_sd_table)
}

# Function to calculate mean Cap for the selected runs
calculate_mean_cap <- function(data, condition, combination) {
  # Extract the runs from the combination column
  selected_runs <- str_split(combination, ", ") %>% unlist()
  
  # Filter the data for the specific condition and selected runs
  filtered_data <- data %>%
    filter(str_detect(Condition, paste0(condition, "_(", paste0(selected_runs, collapse = "|"), ")")))
  
  # Calculate the mean Cap
  mean_cap <- mean(filtered_data$Cap, na.rm = TRUE)
  sd_cap <- sd(filtered_data$Cap, na.rm = TRUE)
  return(list(mean_cap = mean_cap, sd_cap = sd_cap))
}

# Function to Process Data for a Given Temperature
process_data_E <- function(data, temperature) {
  
  # Replace "-run" with "_run" in the Condition column to ensure uniformity
  data <- data %>%
    mutate(Condition = str_replace(Condition, "-run", "_run"))
  
  unique_conditions <- unique(data$Condition)
  
  split_conditions <- data.frame(
    condition = sapply(strsplit(unique_conditions, "_"), `[`, 1),
    run_name = sapply(strsplit(unique_conditions, "_"), `[`, 2)
  )
  
  
  results <- split_conditions %>%
    mutate(timeline_points = map2(condition, run_name, ~get_timeline_points(data, .x, .y)))
  
  final_results <- results %>%
    rowwise() %>%
    mutate(
      before_flat = list(timeline_points$before_flat),
      flat = list(timeline_points$flat),
      after_flat = list(timeline_points$after_flat),
      delta_t1 = timeline_points$delta_t1,
      delta_t2 = timeline_points$delta_t2,
      delta_t3 = timeline_points$delta_t3,
      TOE = timeline_points$TOE
    ) %>%
    select(-timeline_points) %>%
    ungroup()
  
  final_results <- final_results %>%
    mutate(
      before_flat = sapply(before_flat, function(x) toString(x$Time)),
      flat = sapply(flat, function(x) toString(x$Time)),
      after_flat = sapply(after_flat, function(x) toString(x$Time))
    )
  
  # Save the final results as an Excel file
  write_xlsx(final_results, paste0("timeline_results__ethanol_water", temperature, ".xlsx"))
  mean_sd_combinations_table <- final_results %>%
    group_by(condition) %>%
    do(calculate_mean_sd_combinations(.)) %>%
    ungroup()
  
  # Find the combination with the smallest combined SD for delta_t1, delta_t2, delta_t3, and TOE
  best_combinations_table <- mean_sd_combinations_table %>%
    group_by(condition) %>%
    mutate(total_sd = sd_delta_t1 + sd_delta_t2 + sd_delta_t3 + sd_toe) %>%
    slice_min(total_sd) %>%
    ungroup()
  
  # Apply the function to calculate mean Cap for each row of best_combinations_table
  best_combinations_table <- best_combinations_table %>%
    rowwise() %>%
    mutate(
      mean_cap = calculate_mean_cap(data, as.character(condition), as.character(combination))$mean_cap,
      sd_cap = calculate_mean_cap(data, as.character(condition), as.character(combination))$sd_cap
    ) %>%
    ungroup()
  
  # Define the correct order of conditions
  condition_order <- c(
    "Pure Water", "10% Ethanol", "20% Ethanol", "30% Ethanol",
    "40% Ethanol", "50% Ethanol", "60% Ethanol", "70% Ethanol",
    "80% Ethanol", "90% Ethanol", "Pure Ethanol"
  )
  
  # Convert the condition column to a factor with the specified levels
  best_combinations_table$condition <- factor(best_combinations_table$condition, levels = condition_order)
  
  # Arrange the data frame based on the factor levels
  best_combinations_table <- best_combinations_table %>%
    arrange(condition)
  
  # Save the tables to Excel
  write_xlsx(list(
    Mean_SD_Combinations = mean_sd_combinations_table,
    Best_Combinations = best_combinations_table
  ), paste0("mean_sd_combinations_and_selected_runs_ethanol_water", temperature, ".xlsx"))
  
  return(best_combinations_table)
}

# Function to Process Data for a Given Temperature
process_data_M <- function(data, temperature) {
  
  # Replace "-run" with "_run" in the Condition column to ensure uniformity
  data <- data %>%
    mutate(Condition = str_replace(Condition, "-run", "_run"))
  
  unique_conditions <- unique(data$Condition)
  
  split_conditions <- data.frame(
    condition = sapply(strsplit(unique_conditions, "_"), `[`, 1),
    run_name = sapply(strsplit(unique_conditions, "_"), `[`, 2)
  )
  
  
  results <- split_conditions %>%
    mutate(timeline_points = map2(condition, run_name, ~get_timeline_points(data, .x, .y)))
  
  final_results <- results %>%
    rowwise() %>%
    mutate(
      before_flat = list(timeline_points$before_flat),
      flat = list(timeline_points$flat),
      after_flat = list(timeline_points$after_flat),
      delta_t1 = timeline_points$delta_t1,
      delta_t2 = timeline_points$delta_t2,
      delta_t3 = timeline_points$delta_t3,
      TOE = timeline_points$TOE
    ) %>%
    select(-timeline_points) %>%
    ungroup()
  
  final_results <- final_results %>%
    mutate(
      before_flat = sapply(before_flat, function(x) toString(x$Time)),
      flat = sapply(flat, function(x) toString(x$Time)),
      after_flat = sapply(after_flat, function(x) toString(x$Time))
    )
  
  # Save the final results as an Excel file
  write_xlsx(final_results, paste0("timeline_results__Methanol_water", temperature, ".xlsx"))
  mean_sd_combinations_table <- final_results %>%
    group_by(condition) %>%
    do(calculate_mean_sd_combinations(.)) %>%
    ungroup()
  
  # Find the combination with the smallest combined SD for delta_t1, delta_t2, delta_t3, and TOE
  best_combinations_table <- mean_sd_combinations_table %>%
    group_by(condition) %>%
    mutate(total_sd = sd_delta_t1 + sd_delta_t2 + sd_delta_t3 + sd_toe) %>%
    slice_min(total_sd) %>%
    ungroup()
  
  # Apply the function to calculate mean Cap for each row of best_combinations_table
  best_combinations_table <- best_combinations_table %>%
    rowwise() %>%
    mutate(
      mean_cap = calculate_mean_cap(data, as.character(condition), as.character(combination))$mean_cap,
      sd_cap = calculate_mean_cap(data, as.character(condition), as.character(combination))$sd_cap
    ) %>%
    ungroup()
  
  # Define the correct order of conditions
  condition_order <- c(
    "Pure Water", "10% Methanol", "20% Methanol", "30% Methanol",
    "40% Methanol", "50% Methanol", "60% Methanol", "70% Methanol",
    "80% Methanol", "90% Methanol", "Pure Methanol"
  )
  
  # Convert the condition column to a factor with the specified levels
  best_combinations_table$condition <- factor(best_combinations_table$condition, levels = condition_order)
  
  # Arrange the data frame based on the factor levels
  best_combinations_table <- best_combinations_table %>%
    arrange(condition)
  
  # Save the tables to Excel
  write_xlsx(list(
    Mean_SD_Combinations = mean_sd_combinations_table,
    Best_Combinations = best_combinations_table
  ), paste0("mean_sd_combinations_and_selected_runs_Methanol_water", temperature, ".xlsx"))
  
  return(best_combinations_table)
}


process_data_EM <- function(data, temperature) {
  
  # Replace "-run" with "_run" in the Condition column to ensure uniformity
  data <- data %>%
    mutate(Condition = str_replace(Condition, "-run", "_run"))
  
  unique_conditions <- unique(data$Condition)
  
  split_conditions <- data.frame(
    condition = sapply(strsplit(unique_conditions, "_"), `[`, 1),
    run_name = sapply(strsplit(unique_conditions, "_"), `[`, 2)
  )
  
  
  results <- split_conditions %>%
    mutate(timeline_points = map2(condition, run_name, ~get_timeline_points(data, .x, .y)))
  
  final_results <- results %>%
    rowwise() %>%
    mutate(
      before_flat = list(timeline_points$before_flat),
      flat = list(timeline_points$flat),
      after_flat = list(timeline_points$after_flat),
      delta_t1 = timeline_points$delta_t1,
      delta_t2 = timeline_points$delta_t2,
      delta_t3 = timeline_points$delta_t3,
      TOE = timeline_points$TOE
    ) %>%
    select(-timeline_points) %>%
    ungroup()
  
  final_results <- final_results %>%
    mutate(
      before_flat = sapply(before_flat, function(x) toString(x$Time)),
      flat = sapply(flat, function(x) toString(x$Time)),
      after_flat = sapply(after_flat, function(x) toString(x$Time))
    )
  
  # Save the final results as an Excel file
  write_xlsx(final_results, paste0("timeline_results__Methanol_Ethanol", temperature, ".xlsx"))
  mean_sd_combinations_table <- final_results %>%
    group_by(condition) %>%
    do(calculate_mean_sd_combinations(.)) %>%
    ungroup()
  
  # Find the combination with the smallest combined SD for delta_t1, delta_t2, delta_t3, and TOE
  best_combinations_table <- mean_sd_combinations_table %>%
    group_by(condition) %>%
    mutate(total_sd = sd_delta_t1 + sd_delta_t2 + sd_delta_t3 + sd_toe) %>%
    slice_min(total_sd) %>%
    ungroup()
  
  # Apply the function to calculate mean Cap for each row of best_combinations_table
  best_combinations_table <- best_combinations_table %>%
    rowwise() %>%
    mutate(
      mean_cap = calculate_mean_cap(data, as.character(condition), as.character(combination))$mean_cap,
      sd_cap = calculate_mean_cap(data, as.character(condition), as.character(combination))$sd_cap
    ) %>%
    ungroup()
  
  # Define the correct order of conditions
  condition_order <- c(
    "0% Methanol", "10% Methanol", "20% Methanol", "30% Methanol",
    "40% Methanol", "50% Methanol", "60% Methanol", "70% Methanol",
    "80% Methanol", "90% Methanol", "Pure Methanol"
  )
  
  # Convert the condition column to a factor with the specified levels
  best_combinations_table$condition <- factor(best_combinations_table$condition, levels = condition_order)
  
  # Arrange the data frame based on the factor levels
  best_combinations_table <- best_combinations_table %>%
    arrange(condition)
  
  # Save the tables to Excel
  write_xlsx(list(
    Mean_SD_Combinations = mean_sd_combinations_table,
    Best_Combinations = best_combinations_table
  ), paste0("mean_sd_combinations_and_selected_runs_Methanol_Ethanol", temperature, ".xlsx"))
  
  return(best_combinations_table)
}

############Etanol
# Process for each temperature
data_25C <- read.xlsx("reshape_Water-Ethanol.xlsx", sheet = "25C", colNames = TRUE)
result_25C_ethanol <- process_data_E(data_25C, "25C")

data_40C <- read.xlsx("reshape_Water-Ethanol.xlsx", sheet = "40C", colNames = TRUE)
result_40C_ethanol <- process_data_E(data_40C, "40C")


data_50C <- read.xlsx("reshape_Water-Ethanol.xlsx", sheet = "50C", colNames = TRUE)
result_50C_ethanol <- process_data_E(data_50C, "50C")


data_60C <- read.xlsx("reshape_Water-Ethanol.xlsx", sheet = "60C", colNames = TRUE)
result_60C_ethanol <- process_data_E(data_60C, "60C")

#############Metanol
# Process for each temperature
data_25C <- read.xlsx("reshape_Water-Methanol.xlsx", sheet = "25C", colNames = TRUE)
result_25C_methanol <- process_data_M(data_25C, "25C")

data_40C <- read.xlsx("reshape_Water-Methanol.xlsx", sheet = "40C", colNames = TRUE)
result_40C_methanol <- process_data_M(data_40C, "40C")


data_50C <- read.xlsx("reshape_Water-Methanol.xlsx", sheet = "50C", colNames = TRUE)
result_50C_methanol <- process_data_M(data_50C, "50C")


data_60C <- read.xlsx("reshape_Water-Methanol.xlsx", sheet = "60C", colNames = TRUE)
result_60C_methanol <- process_data_M(data_60C, "60C")
###############Methanol_ethanol
data_25C <- read.xlsx("reshape_Methanol-Ethanol.xlsx", sheet = "25C", colNames = TRUE)
result_25C_ethanol_methanol  <- process_data_EM(data_25C, "25C")

# Function to convert condition labels to numeric values
convert_conditions_to_numeric <- function(df, chemical_type) {
  df <- df %>%
    mutate(condition_numeric = case_when(
      condition == "Pure Water" & chemical_type == "Ethanol" ~ 0,
      condition == "Pure Ethanol" & chemical_type == "Ethanol" ~ 100,
      condition == "0% Methanol" & chemical_type == "Methanol" ~ 0,
      condition == "Pure Water" & chemical_type == "Methanol" ~ 0,
      condition == "Pure Methanol" & chemical_type == "Methanol" ~ 100,
      str_detect(condition, "% Ethanol") & chemical_type == "Ethanol" ~ as.numeric(str_replace(condition, "% Ethanol", "")),
      str_detect(condition, "% Methanol") & chemical_type == "Methanol-Ethanol" ~ as.numeric(str_replace(condition, "% Methanol", "")),
      str_detect(condition, "% Methanol") & chemical_type == "Methanol" ~ as.numeric(str_replace(condition, "% Methanol", "")),
      TRUE ~ NA_real_
    ))
  
  return(df)
}


# Assuming the individual datasets for ethanol, methanol, and methanol-ethanol are result_25C, result_40C, etc.

# Convert the condition columns for each result set
result_25C_ethanol <- convert_conditions_to_numeric(result_25C_ethanol, "Ethanol")
result_40C_ethanol <- convert_conditions_to_numeric(result_40C_ethanol, "Ethanol")
result_50C_ethanol <- convert_conditions_to_numeric(result_50C_ethanol, "Ethanol")
result_60C_ethanol <- convert_conditions_to_numeric(result_60C_ethanol, "Ethanol")

result_25C_methanol <- convert_conditions_to_numeric(result_25C_methanol, "Methanol")
result_40C_methanol <- convert_conditions_to_numeric(result_40C_methanol, "Methanol")
result_50C_methanol <- convert_conditions_to_numeric(result_50C_methanol, "Methanol")
result_60C_methanol<- convert_conditions_to_numeric(result_60C_methanol, "Methanol")


result_25C_methanol_ethanol<- convert_conditions_to_numeric(result_25C_ethanol_methanol, "Methanol")

# Combine the datasets with the numeric condition values for plotting
combined_data <- bind_rows(
  result_25C_ethanol %>% mutate(temperature = "25C", typemix = "E"),
  result_40C_ethanol %>% mutate(temperature = "40C", typemix = "E"),
  result_50C_ethanol %>% mutate(temperature = "50C", typemix = "E"),
  result_60C_ethanol %>% mutate(temperature = "60C", typemix = "E"),
  
  result_25C_methanol %>% mutate(temperature = "25C", typemix = "M"),
  result_40C_methanol %>% mutate(temperature = "40C", typemix = "M"),
  result_50C_methanol %>% mutate(temperature = "50C", typemix = "M"),
  result_60C_methanol %>% mutate(temperature = "60C", typemix = "M"),
  
  result_25C_methanol_ethanol %>% mutate(temperature = "25C", typemix = "EM")
)

# Function to generate the boxplot with jitter points and correct label order
generate_combined_boxplot <- function(data_combined) {
  
  # Ensure the order of `typemix` is correct (E, M, then EM)
  data_combined <- data_combined %>%
    mutate(typemix = factor(typemix, levels = c("E", "M", "EM")))
  
  p <- ggplot(data_combined, aes(x = temperature, y = mean_delta_t2, fill = typemix)) +
    geom_boxplot(outlier.shape = NA, position = position_dodge(width = 0.8)) +
    
    # Add jitter points with a gradient color based on concentration
    geom_jitter(aes(color = condition_numeric), size = 4, position = position_jitterdodge(dodge.width = 0.8)) +
    
    # Set up the gradient color from light gray (low concentration) to black (high concentration)
    scale_color_gradientn(colors = c("lightgray", "black"), limits = c(0, 100), name = "Concentration") + 
    
    # Customize the fill colors for the boxplots and update the legend labels
    scale_fill_manual(
      values = c("E" = "navyblue", "M" = "#B8860B", "EM" = "darkred"),
      labels = c("E" = "E-W(%E)", "M" = "M-W(%M)", "EM" = "M-E(%M)"),
      guide = guide_legend(override.aes = list(shape = NA))
    ) +
    
    # Customize the x and y axis labels
    labs(
      x = "Temperature (°C)",
      y = expression(bold("Mean"~Delta~t[2]~(s))),
      fill = "Mixer Type"  # Label for the mix type legend
    ) +
    
    # Customize the theme
    theme_minimal() +
    theme(
      axis.text = element_text(size = 12, family = "Times New Roman", face = "bold"),
      axis.title = element_text(size = 12, family = "Times New Roman", face = "bold"),
      legend.text = element_text(size = 12, family = "Times New Roman", face = "bold"),
      legend.title = element_text(size = 12, family = "Times New Roman", face = "bold"),
      legend.position = c(0.85, 0.65), 
      legend.direction = "vertical",
      panel.background = element_blank(),
      plot.background = element_blank(),
      panel.grid.major = element_blank(),
      panel.grid.minor = element_blank(),
      panel.border = element_rect(color = "black", fill = NA),
    )
  return(p)
}

# Generate the boxplot with the updated annotation order
combined_plot <- generate_combined_boxplot(combined_data)
ggsave("boxplotall.png", plot = combined_plot, width = 8, height = 6)

#######__________arrange
# Define temp_label for 25°C
library(magick)


  left_image <- image_read("boxplotall.png") %>% 
    image_background("white") %>% 
    image_resize("800x600")
  
  right_image <- image_read("boxplot_with_table_inside.png") %>% 
    image_background("white") %>% 
    image_resize("900x600")
  

  # Combine the left and right images side by side
  final_combined_image <- image_append(c(left_image, right_image))
  
  # Save the combined image
  image_write(final_combined_image,"combined_image_boxline.png")

  knitr::include_graphics("combined_image_boxline.png")
```

## 4.2 FigureS14

```
# ============================================================
# Multi-panel Model Comparison (Linear, LOESS, NN, GAM, SVR)
# 3x5 Combined Layout with Larger Font and Titles
# ============================================================

rm(list = ls())
library(tidyverse)
library(openxlsx)
library(nnet)
library(mgcv)
library(e1071)
library(Metrics)
library(ggpubr)
library(cowplot)
library(writexl)

#-----------------------------------------
# Helper: compute R²
#-----------------------------------------
compute_r2 <- function(y, yhat) {
  1 - sum((y - yhat)^2) / sum((y - mean(y))^2)
}

#-----------------------------------------
# Function: Fit and plot all models
#-----------------------------------------
fit_plot_models <- function(df, yvar, system_name, color_point = "#D19000") {
  # Clean concentration
  df <- df %>%
    mutate(
      conc = as.numeric(str_extract(condition, "\\d+")),
      conc = case_when(
        is.na(conc) & grepl("Pure Water", condition, ignore.case = TRUE) ~ 0,
        is.na(conc) & grepl("Pure Ethanol", condition, ignore.case = TRUE) ~ 100,
        is.na(conc) & grepl("Pure Methanol", condition, ignore.case = TRUE) ~ 100,
        TRUE ~ conc
      )
    ) %>%
    arrange(conc)
  
  x <- df$conc
  y <- df[[yvar]]
  
  # === Linear ===
  lm_model <- lm(y ~ x)
  pred_lm <- predict(lm_model)
  r2_lm <- compute_r2(y, pred_lm)
  rmse_lm <- rmse(y, pred_lm)
  
  # === LOESS ===
  loess_model <- loess(y ~ x, span = 0.75)
  pred_loess <- predict(loess_model)
  r2_loess <- compute_r2(y, pred_loess)
  rmse_loess <- rmse(y, pred_loess)
  
  # === Neural Network ===
  set.seed(123)
  nn_model <- nnet(y ~ x, size = 3, linout = TRUE, decay = 0.001, maxit = 500, trace = FALSE)
  pred_nn <- predict(nn_model)
  r2_nn <- compute_r2(y, pred_nn)
  rmse_nn <- rmse(y, pred_nn)
  
  # === GAM ===
  gam_model <- gam(y ~ s(x, k = 5))
  pred_gam <- predict(gam_model)
  r2_gam <- compute_r2(y, pred_gam)
  rmse_gam <- rmse(y, pred_gam)
  
  # === SVR ===
  svr_model <- svm(y ~ x, kernel = "radial")
  pred_svr <- predict(svr_model)
  r2_svr <- compute_r2(y, pred_svr)
  rmse_svr <- rmse(y, pred_svr)
  
  # === Data for plotting ===
  df_plot <- tibble(
    conc = x,
    observed = y,
    linear = pred_lm,
    loess = pred_loess,
    nn = pred_nn,
    gam = pred_gam,
    svr = pred_svr
  )
  
  # === Plot ===
  p <- ggplot(df_plot, aes(x = conc)) +
    geom_point(aes(y = observed), color = color_point, size = 3) +
    geom_line(aes(y = linear), color = "#003366", linetype = "dashed", size = 1) +  # navy blue dashed
    geom_line(aes(y = loess), color = "#D19000", size = 1.2) +                      # golden brown
    geom_line(aes(y = nn), color = "darkred", size = 1.1) +                         # dark red
    geom_line(aes(y = gam), color = "darkgreen", size = 1.1) +                      # dark green
    geom_line(aes(y = svr), color = "black", size = 1.1) +                          # black
    theme_classic(base_size = 18) +
    labs(x = "Methanol (%)", y = yvar) +
    annotate(
      "text", x = Inf, y = Inf,
      label = paste0("Linear: R²=", sprintf("%.3f", r2_lm),
                     "\nLOESS: R²=", sprintf("%.3f", r2_loess),
                     "\nNN: R²=", sprintf("%.3f", r2_nn),
                     "\nGAM: R²=", sprintf("%.3f", r2_gam),
                     "\nSVR: R²=", sprintf("%.3f", r2_svr)),
      hjust = 1.1, vjust = 1.1, size = 5.5, color = "black"
    ) +
    theme(
      axis.text = element_text(size = 20, face = "bold", color = "black"),
      axis.title = element_text(size = 20, face = "bold", color = "black"),
      plot.margin = margin(15, 20, 15, 20)
    )
  
  # Return both plot and numeric summary
  list(
    plot = p,
    metrics = tibble(
      system = system_name,
      metric = yvar,
      R2_linear = r2_lm, RMSE_linear = rmse_lm,
      R2_loess = r2_loess, RMSE_loess = rmse_loess,
      R2_nn = r2_nn, RMSE_nn = rmse_nn,
      R2_gam = r2_gam, RMSE_gam = rmse_gam,
      R2_svr = r2_svr, RMSE_svr = rmse_svr
    )
  )
}

#-----------------------------------------
# Process each solvent system
#-----------------------------------------
process_system <- function(file_path, system_name) {
  cat("\n=== Processing:", system_name, "===\n")
  df <- read.xlsx(file_path, sheet = "Best_Combinations")
  metrics <- c("mean_delta_t1", "mean_delta_t2", "mean_delta_t3", "mean_toe", "mean_cap")
  
  plot_list <- list()
  all_metrics <- list()
  
  for (m in metrics) {
    res <- fit_plot_models(df, m, system_name)
    plot_list[[m]] <- res$plot
    all_metrics[[m]] <- res$metrics
  }
  
  summary_tbl <- bind_rows(all_metrics)
  return(list(summary = summary_tbl, plots = plot_list))
}

#-----------------------------------------
# Run for all systems
#-----------------------------------------
ethanol <- process_system("mean_sd_combinations_and_selected_runs_ethanol_water25C.xlsx", "Ethanol–Water")
```

```
## 
## === Processing: Ethanol–Water ===
```

```
methanol <- process_system("mean_sd_combinations_and_selected_runs_Methanol_water25C.xlsx", "Methanol–Water")
```

```
## 
## === Processing: Methanol–Water ===
```

```
mixed <- process_system("mean_sd_combinations_and_selected_runs_methanol_ethanol25C.xlsx", "Methanol–Ethanol")
```

```
## 
## === Processing: Methanol–Ethanol ===
```

```
#-----------------------------------------
# Combine plots with titles (3x5 grid)
#-----------------------------------------
combined_plot <- plot_grid(
  ggdraw() + draw_label("Ethanol–Water", fontface = "bold", size = 22, y = 0.5),
  plot_grid(plotlist = ethanol$plots, ncol = 5),
  ggdraw() + draw_label("Methanol–Water", fontface = "bold", size = 22, y = 0.5),
  plot_grid(plotlist = methanol$plots, ncol = 5),
  ggdraw() + draw_label("Methanol–Ethanol", fontface = "bold", size = 22, y = 0.5),
  plot_grid(plotlist = mixed$plots, ncol = 5),
  ncol = 1,
  rel_heights = c(0.15, 1, 0.15, 1, 0.15, 1)
)

#ggsave("ModelComparison_AllSystems.png",
      # combined_plot, width = 22, height = 18, dpi = 300)


  knitr::include_graphics("ModelComparison_AllSystems.png")
```
